# Supplementary material for: Cage-confined photocatalysis for wide-scope unusually selective [2 + 2] cycloaddition through visible-light triplet sensitization
Source: Nat Commun. 2020 Sep 16;11:4675. doi: 10.1038/s41467-020-18487-5 (PMC7494878; doi:10.1038/s41467-020-18487-5)
Supplement: Supplementary file 1 — Supplementary information [file 41467_2020_18487_MOESM1_ESM.pdf]

Supplementary Information

Cage-confined photocatalysis for wide-scope unusually selective [2+2] cycloaddition through visible-light triplet sensitization

Jing-Si Wang, et al

Supplementary Methods

Synthesis and single-crystal structural determination of MOC-16 and guests⊂MOC-16

MOC-16 containing BF_4^- and PF_6^- anions was prepared following our previously reported procedure[1] with stepwise assembly from metalloligand $RuL_3(PF_6)_2$ ((L = 2-(pyridin-3-yl)-1H-imidazo [4,5-f][1,10]-phenanthroline) and $Pd(BF_4)_2$. Single-crystals of MOC-16 suitable for X-ray diffraction were obtained by slow vapor diffusion of diethyl ether into a 200 μL CH_3CN solution of MOC-16 for four weeks. The cages encapsulating hexane or heptane guest molecules in their rhombic portals are obtained by immersing MOC-16 crystals in hexane or heptane solution for several hours.

Single-crystals of MOC-16 and guests⊂MOC-16 were carefully picked, coated with paratone oil and attached to a glass fiber, which was inserted in a stainless steel stick. Crystals were quickly mounted to the Agilent Gemini S Ultra CCD Diffractometer with the Enhance X-ray Source of Cu radiation (λ = 1.54184 Å) using the ω-scan technique. All of the structures were solved by direct methods and refined by full-matrix least squares against F^2 using the SHELXL programs.[2] Hydrogen atoms were placed in geometrically calculated positions and included in the refinement process using riding model with isotropic thermal parameters: Uiso(H) = 1.2 Ueq(-CH). All the electrons of disordered solvent molecules which cannot be determined are removed by SQUEEZE routine of PLATON program.[3] Crystal and refinement parameters are listed in Supplementary Table 1, and selected bond lengths (Å) and angles (°) in Supplementary Table 2-4.

Single-crystal structures of products 1 and 70.

Diffraction data were collected on an Agilent SuperNova X-Ray diffractometer using micro-focus X-ray sources (Cu-$K\alpha$, λ =1.54184 Å). Crystal and refinement parameters are listed in Supplementary Table 5, and selected bond lengths (Å) and angles (°) in Supplementary Table 6,7.

General procedure for preparation of *α,β*-unsaturated ketones.

Synthesis of chalcone derivatives

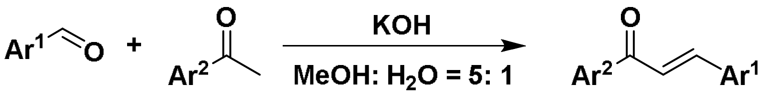

In a 100 mL flask, a solution of aldehyde (15 mmol) in 50 mL methanol at 0 °C was added aqueous KOH solution (40 mmol in 10 mL H_2O) slowly and stirred for 10 min, then ketone was added in portions at 0 ºC. The reaction was monitored by TLC. After complete consumption of the starting materials, MeOH was removed under reduced pressure. The residue was extracted with EtOAc (3 x 15 mL). The combined organic phase was washed with brine, dried over Na_2SO_4, and concentrated *in vacuo*. The residue was purified by flash chromatography on silica gel to give the corresponding product.

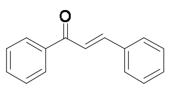
S1

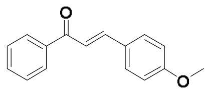
S2

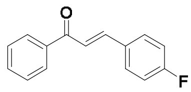
S3

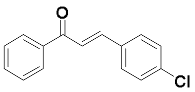
S4

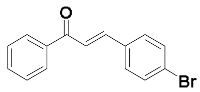
S5

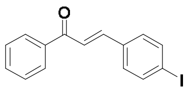
S6

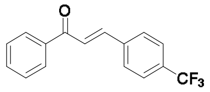
S7

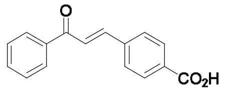
S8

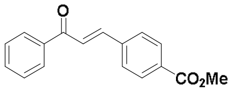
S9

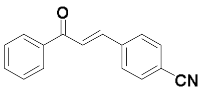
S10

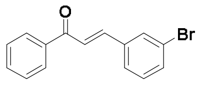
S11

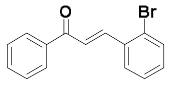
S12

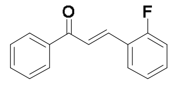
S13

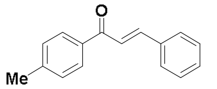
S14

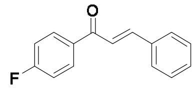
S15

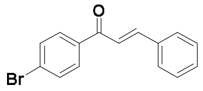
S16

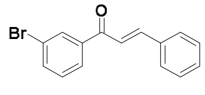
S17

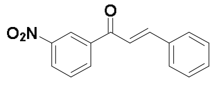
S18

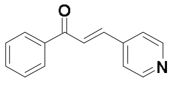
S19

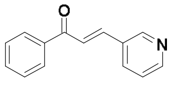
S20

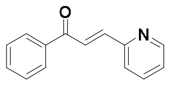
S21

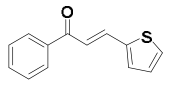
S22

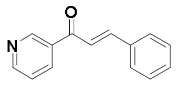
S23

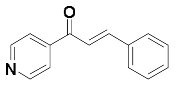
S24

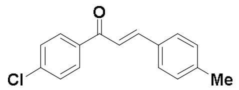
S25

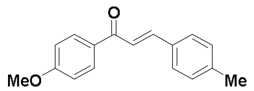
S26

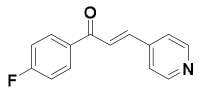
S27

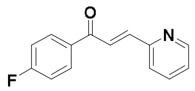
S28

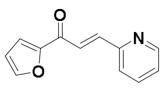
S29

Synthesis of cinnamic ester derivatives

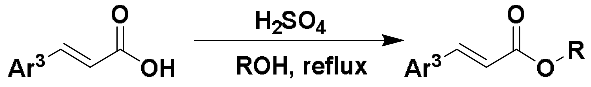

In a 100mL flask, α,β-unsaturated carboxylic acid (6 mmol) was dissolved in alcohol (20 mL), and H_2SO_4 (9 mmol) was added in portions. The reaction was stirred at reflux for 16 h then cooled to room temperature. The mixture was poured into H_2O (40 mL) and extracted with EtOAc (3 x 15 mL). The combined organic phase was washed with saturated aqueous $NaHCO_3$, dried over Na_2SO_4 and concentrated *in vacuo*. The residue was purified by flash chromatography to give the corresponding ester.

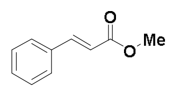
S30

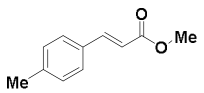
S31

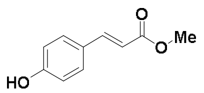
S32

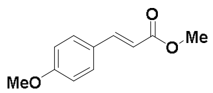
S33

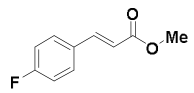
S34

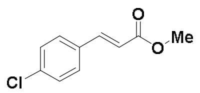
S35

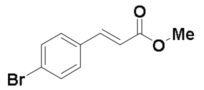
S36

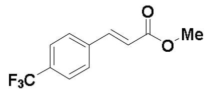
S37

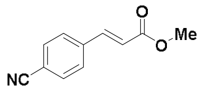
S38

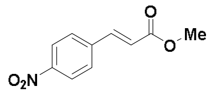
S39

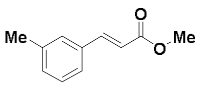
S40

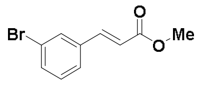
S41

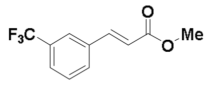
S42

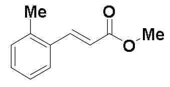
S43

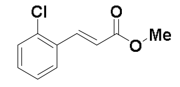
S44

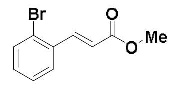
S45

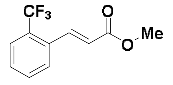
S46

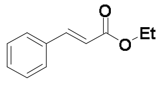
S47

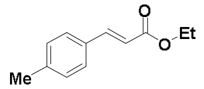
S48

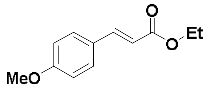
S49

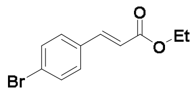
S50

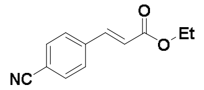
S51

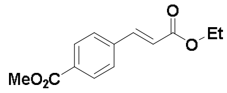
S52

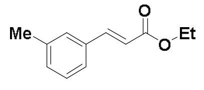
S53

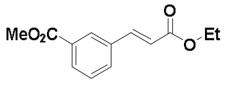
S54

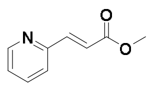
S55

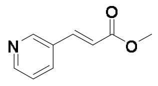
S56

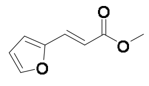
S57

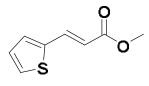
S58

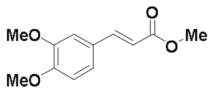
S59

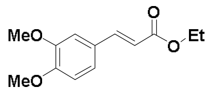
S60

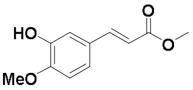
S61

Synthesis of enone derivatives

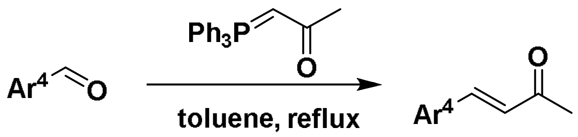

To a 100 mL flask equipped with a stir bar and a condenser was added carboxaldehyde (0.8 mmol), 1-(triphenylphosphoranylidene)-2-propanone (1.12 mmol), and toluene (20 ml). The reaction mixture was refluxed for 10 h. After completion, the reaction mixture was concentrated under reduced pressure. The crude mixture was purified by flash chromatography on silica gel to give the corresponding product.

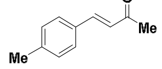
S62

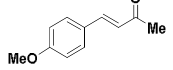
S63

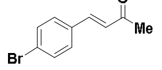
664

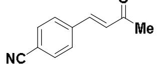
S65

The characteristic data of **S1, S2, S3, S4, S7, S10, S13, S14, S25, S26** was consistent with ref. 4; **S5, S15, S16, S17** was consistent with ref. 6; **S18** was consistent with ref. 7; **S8** was consistent with ref. 8; **S9** was consistent with the ref. 9. **S6** was consistent with ref. 10; **S11, S12, S22** was consistent with ref. 11; **S19** was consistent with ref. 12; **S29** was consistent with ref. 13; **S28** was consistent with ref. 14; **S20, S21, S23, S24** was consistent with ref. 15; **S31, S49, S50, S58, S60** was consistent with ref. 16; **S33, S34, S41, S42** was consistent with ref. 17; **S35, S36, S44, S45** was consistent with ref. 18; **S30, S48, S51, S52, S53** was consistent with ref. 5; **S37** was consistent with ref. 19; **S32, S55, S57** was consistent with ref. 20; **S38, S39, S43, S56, S59** was consistent with ref. 21; **S40**, was consistent with ref. 22; **S46**, was consistent with ref. 23; **S54** was consistent with ref. 24; **S61** was consistent with ref. 25; **S62, S63** was consistent with ref. 26; **S64** was consistent with ref. 27; **S65** was consistent with ref. 28.

S27

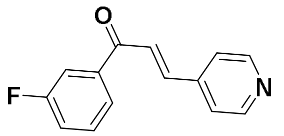

This compound was prepared according to the synthesis of chalcone derivetives. Yield 20% (white solid).^{1}H NMR (400 MHz, $CDCl_3$) δ 8.70 (d, J = 6.0 Hz, 2H), 8.23-7.97 (m, 2H), 7.75-7.58 (m, 2H),7.54-7.44 (m, 2H), 7.28-7.16(m, 2H). ^{13}C NMR (101 MHz, $CDCl_3$) δ 188.19, 166.07 (d, J = 255.7 Hz), 150.47, 142.46, 141.73,133.97 (d, J = 2.9 Hz), 131.42 (d, J = 9.3 Hz), 125.97, 122.27, 116.16 (d, J = 21.9 Hz). ^{19}F NMR (377 MHz, $CDCl_3$) δ -104.67 (s, 1F). HRMS (ESI) Calcd. for $C_{14}H_{10}FNO$ $[M+H]^+$: 228.0819. Found: 228.0817.

The dimerization of *α,β*-unsaturated carbonyl compounds

The photocatalytic [2+2] cycloaddition reactions were carried out with blue LED (450 nm, Supplementary Fig. 3) at room temperature in the following five types of procedures:

General Procedure A for dimerization of *α,β*-unsaturated carbonyl compounds

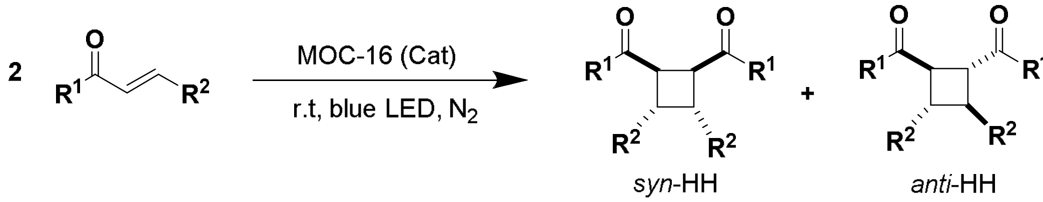

A 25 mL Schlenk flask equipped with a magnetic stir bar was charged with substrate (0.10 mmol), MOC-16 (0.03 mol%) and DMSO (0.75 mL). The solution was degassed by N_2 for 10 min. Then degassed H_2O (2.25 mL) was added. The mixture was irradiated with 24 W LEDs. Upon completion of the reaction (1 to 24 h), the residue was extracted with EtOAc (3 x 15mL). The combined organic phase was washed with brine, dried over Na_2SO_4, and concentrated in *vacuo*. The residue was dissolved in $CDCl_3$ for ^{1}H NMR analysis to test the d.r. value and then collected and purified by flash chromatography on silica gel (eluting with hexane/EtOAc = 100:1) to afford the desired product.

General Procedure B for dimerization of *α,β*-unsaturated carbonyl compounds

A 25 mL Schlenk flask equipped with a magnetic stir bar was charged with substrate (0.10 mmol), MOC-16 (0.08 mol%) and DMSO (0.75 mL). The solution was degassed by N_2 for 10 min. Then degassed H_2O (2.25 mL) was added. The mixture was irradiated with 24 W LEDs. Upon completion of the reaction (1 to 24 h), the residue was extracted with EtOAc (3 x 15 mL). The combined organic phase was washed with brine, dried over Na_2SO_4, and concentrated in *vacuo*. The residue was dissolved in $CDCl_3$ for ^1H NMR analysis to test the d.r. value and then collected and purified by flash chromatography on silica gel (eluting with hexane/EtOAc = 100:1) to afford the desired product.

General Procedure C for dimerization of *α,β*-unsaturated carbonyl compounds

A 25 mL Schlenk flask equipped with a magnetic stir bar was charged with substrate (0.10 mmol) and MOC-16 (0.08 mol%). After evacuation and refill with dry N_2 for three times, degassed acetone (2 mL) and H_2O (1 mL) was added. The mixture was irradiated with 24 W blue LEDs. Upon completion of the reaction (1 to 24 h), the mixture was extracted with EtOAc (3 x 15 mL). The combined organic phase was washed with brine, dried over Na_2SO_4, and concentrated in *vacuo*. The residue was dissolved in $CDCl_3$ for ^1H NMR analysis to test the d.r. value and then collected and purified by flash chromatography on silica gel (eluting with hexane/EtOAc = 100:1) to afford the desired product.

General Procedure D for the cross dimerization of α,β-unsaturated carbonyl compounds

A 25 mL Schlenk flask equipped with a magnetic stir bar was charged with chalcone (0.20 mmol), cinnamate (0.10 mmol), MOC-16 (0.12 mol%) and DMSO (0.75 mL). The solution was degassed by N_2 for 10 min. Then degassed H_2O (2.25 mL) was added. The mixture was irradiated with 24 W LEDs. Upon completion of the reaction (10 h), the residue was extracted with EtOAc (3 x 15 mL). The combined organic phase was washed with brine, dried over Na_2SO_4, and concentrated in *vacuo*. The residue was dissolved in $CDCl_3$ for ^1H NMR analysis to test the d.r. value and then collected and purified by flash chromatography on silica gel (eluting with hexane/EtOAc = 100:1) to afford the desired product.

General Procedure E for the cross dimerization of *α,β*-unsaturated carbonyl compounds

A 25 mL Schlenk flask equipped with a magnetic stir bar was charged with chalcone (0.10 mmol), benzylideneacetone (0.20 mmol), MOC-16 (0.08 mol%) and DMSO (0.75 mL). The solution was degassed by N_2 for 10 min. Then degassed H_2O (2.25 mL) was added. The mixture was irradiated with 24 W LEDs. Upon completion of the reaction (10 h), the residue was extracted with EtOAc (3 x 15 mL). The combined organic phase was washed with brine, dried over Na_2SO_4, and concentrated *in vacuo*. The residue was purified by flash chromatography on silica gel (eluting with hexane/EtOAc = 100:1) to afford the desired product.

Kinetic study using chalcone as the substrate

General procedure A was followed using MOC-16 (0.03 mol%) or RuL_3 (0.24 mol%) as photocatalysts. The reaction mixture was monitored at indicated time intervals, and the conversions of chalcone were determined by ^1H NMR using mesitylene as internal standard.

Kinetic study using ethyl 4-bromocinnamate as substrate

General procedure B was followed using MOC-16 (0.08 mol%) or RuL_3 (0.64 mol%) as photocatalysts. The reaction mixture was monitored at indicated time intervals, and the conversions of chalcone were determined by ^1H NMR using mesitylene as internal standard.

Synthesis of ethyl (*E*)-2-methyl-3-phenylacrylate

Ethyl (*E*)-2-methyl-3-phenylacrylate was synthesized following the general procedure of synthesis of cinnamic ester derivatives. The characteristic data of ethyl (*E*)-2-methyl-3-phenylacrylate was consistent with ref. 29. A 25 mL Schlenk flask equipped with a magnetic stir bar was charged with ethyl (*E*)-2-methyl-3-phenylacrylate (0.10 mmol), MOC-16 (0.12 mol%) and DMSO (0.75 mL). The solution was degassed by N_2 for 10 min. Then degassed H_2O (2.25 mL) was added. The mixture was irradiated with 24 W blue LEDs for 15 h. Then the reaction mixture was extracted with EtOAc. The combined organic phase was washed with brine, dried over Na_2SO_4, then concentrated in *vacuo* and the residue was purified by flash chromatography on silica gel to get the mixed olefin isomers and send for ^1H NMR test. After the test, the *Z*-isomer was separated *via* chromatography on silica gel and characterized. The characteristic data of ethyl (*Z*)-2-methyl-3-phenylacrylate was consistent with ref. 30. ^1H NMR (400 MHz, $CDCl_3$) δ 7.32 – 7.03 (m, 5H), 6.64 (s, 1H), 4.04 (q, J = 7.2 Hz, 2H), 2.02 (s, 3H), 1.03 (t, J = 7.2 Hz, 3H). ^{13}C NMR (101 MHz, $CDCl_3$) δ 169.84, 136.60, 134.43, 130.37, 128.24, 128.14, 127.67, 60.74, 21.56, 13.92.

Supplementary Discussion

Optimization and control experiments of chalcone photocycloaddition

Chalcone was chosen as the benchmark substrate to optimize the reactivity of [2+2] cycloaddition by applying MOC-16 as the photocatalyst (Supplementary Table 8). In aprotic organic solvents like acetone and CH_3CN, photodimerization is inefficient with *anti*-HH isomer as the major homocoupling product, showing similar diastereoselectivity as reported by using iridium-sensitizers.[4,5] Changing reaction media to hydrous organic solvents, *i.e.* acetone-H_2O or CH_3CN-H_2O, results in better conversion (70-75%), but *anti*-HH diastereomers are still predominant. Further screening of solvents shows exciting results when using hydrous DMSO-H_2O mixed solvent. Excellent yield with uncommonly reversed diastereomeric ratio (d.r.) of 1.5: 1 (*syn*-HH: *anti*-HH) is observed. This infusive result implies an important role of host-guest interactions, relying on hydrophobic effect in different solvents, for the efficiency and selectivity of [2+2] photocycloaddition，accounting for the reason to switch the diastereoselectivity of photoreactions between acetone-H_2O or CH_3CN-H_2O solutions (week host-guest interactions) and DMSO-H_2O media (hydrophobic effect enforced host-guest interactions). To investigate the influence of steric and electronic effect, substrates with different substitutes have been tested, applying RuL_3 as the catalyst for comparison (entries 21-27). From the results we can see that the diastereoselectivity and performance of the Ru-metalloligand photocatalyst are somewhat influenced by the substitutes and much lower than those of MOC-16 cage photocatalyst. No obvious regularity regarding the reaction selectivity is observed between substrates with electron-rich and deficient groups.

Triplet energy transfer mechanism study

E-Z isomerization of olefin

Triplet intermediates of olefins may show *trans-cis* (or *E-Z*) isomerization. To prove the triplet energy-transfer mechanism for olefin photosensitization, two substrates (chalcone and ethyl 4-bromocinnamate) have been tested and the reaction mixtures have been checked after running the reactions for different time (5, 10, 15, 30, 60 and 180 min, respectively). However, no *cis*-isomer was observed, indicating that the diradical intermediates are active enough for [2+2] cycloaddition under the reaction conditions. Alternatively, a cinnamate substrate with significantly steric hindrance (ethyl (*E*)-2-methyl-3-phenylacrylate) was studied, and the *Z*-isomer was obtained successfully in the absence of [2+2] cycloaddition product under the reaction conditions, resulting in mixed olefins with an *E/Z* ratio of 2.17: 1 (Supplementary Fig. 11).

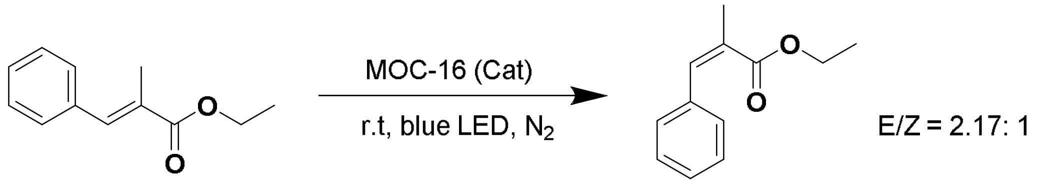

Absorption and emission spectra of MOC-16 and chalcone

UV-Vis absorption and emission study of MOC-16 and chalcone support the triplet energy transfer mechanism. Exciting MOC-16 with 450 nm light resulted in a broad emission band around 610 nm, which belongs to the ^{3}MLCT emission of MOC-16[31] and covers the triplet energy area of chalcone (*ca.* 590 nm)[4]. Emission quenching is clearly observed by exciting the mixed solvent of MOC-16 and chalcone (Supplementary Fig. 12).

Supplementary Figures

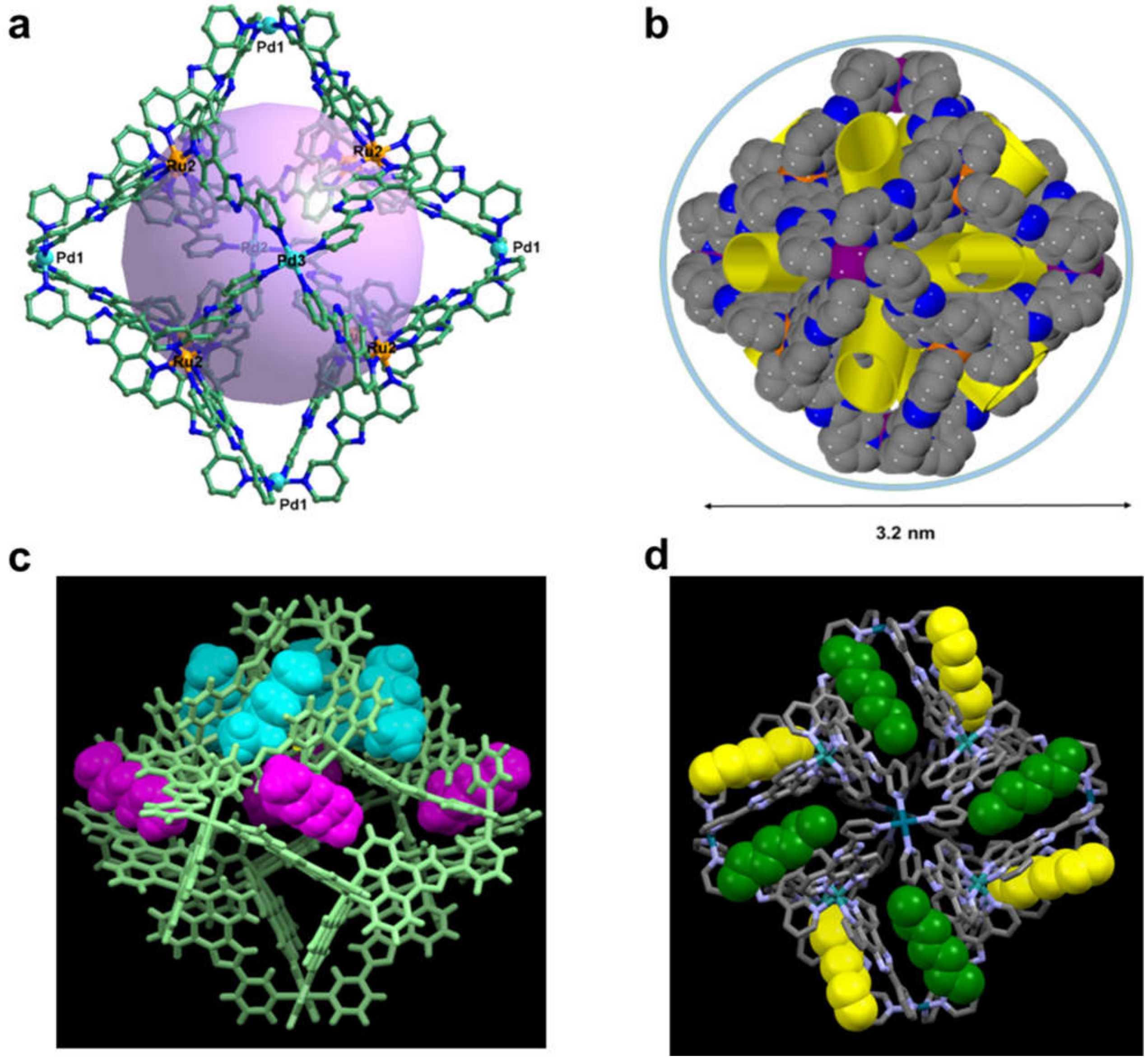

Supplementary Figure 1 | Single-crystal structures of Cages. a, Nanosized MOC-16 showing inner cavity with a purple ball. **b**, Space-fill mode of MOC16 showing the rhombic portals and a diameter about 3.2 nm of its outer sphere. **c,d**, Encapsulation of eight guest molecules of hexane **c**) or heptane **d**) by one cage in it portals, viewed from the top or side directions. Hexane or heptane molecules are shown in space-filling mode.

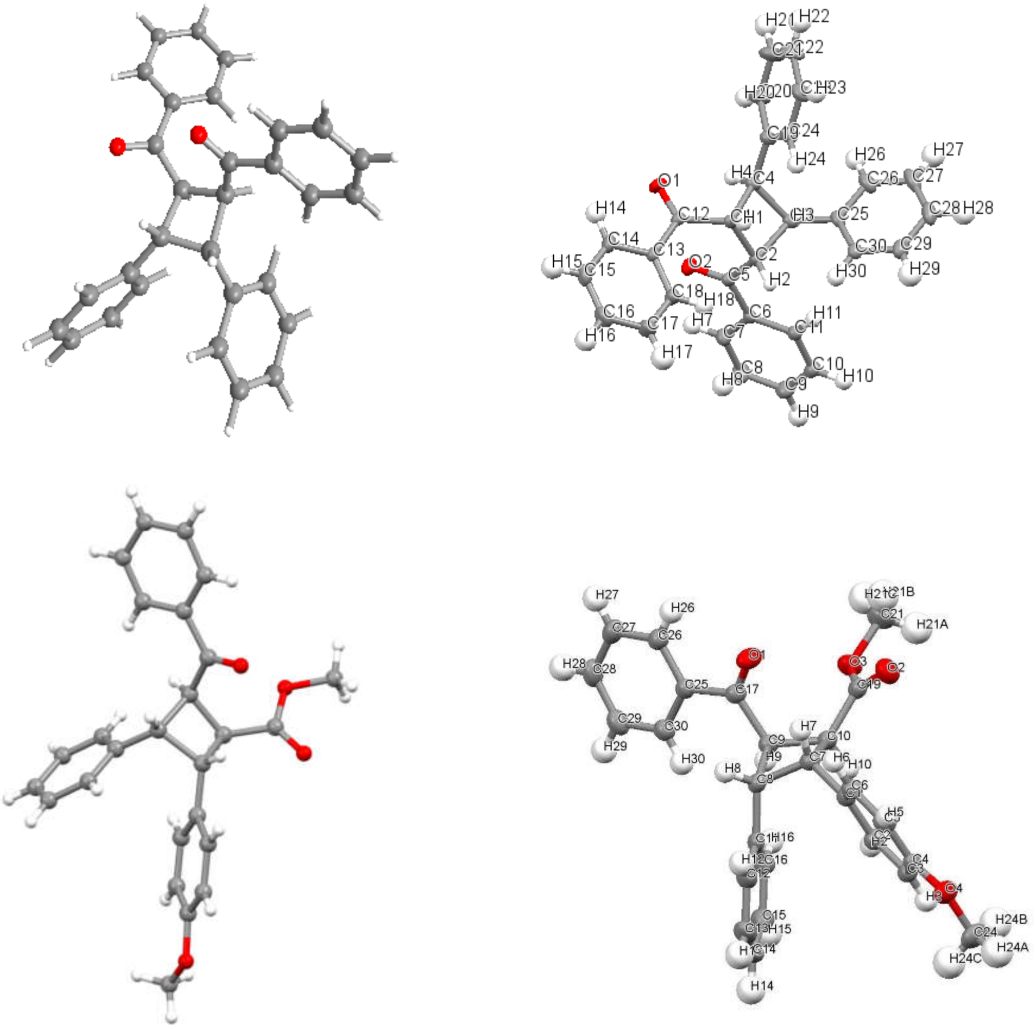

Supplementary Figure 2 | Crystal structures of photoproducts **1** (upper) and **70** (lower).

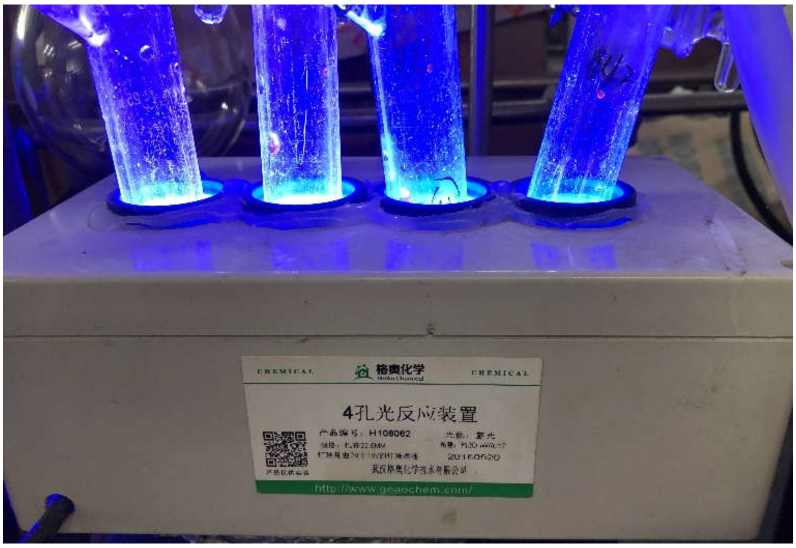

Supplementary Figure 3 | 24 W blue LED light source used for the [2+2] cycloaddition reactions.

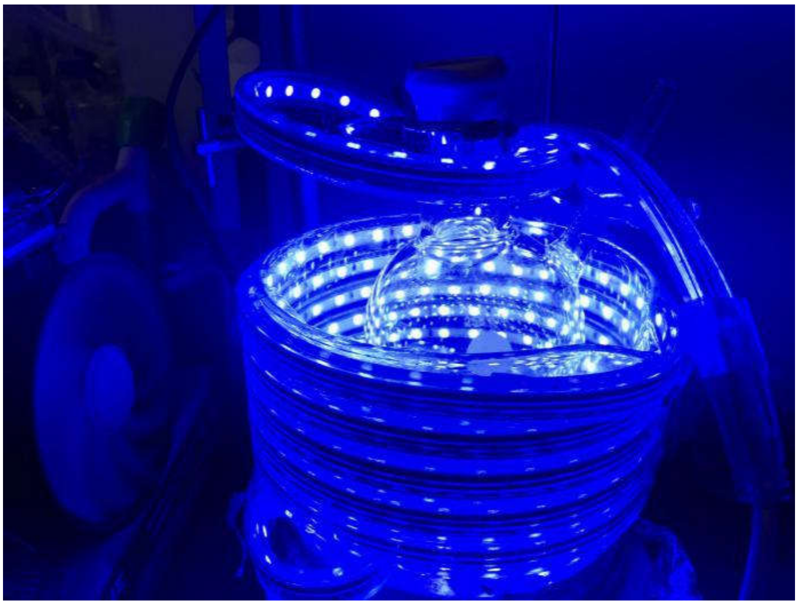

Supplementary Figure 4 | 40 W blue LED light strip used for gram-scale photocycloaddition.

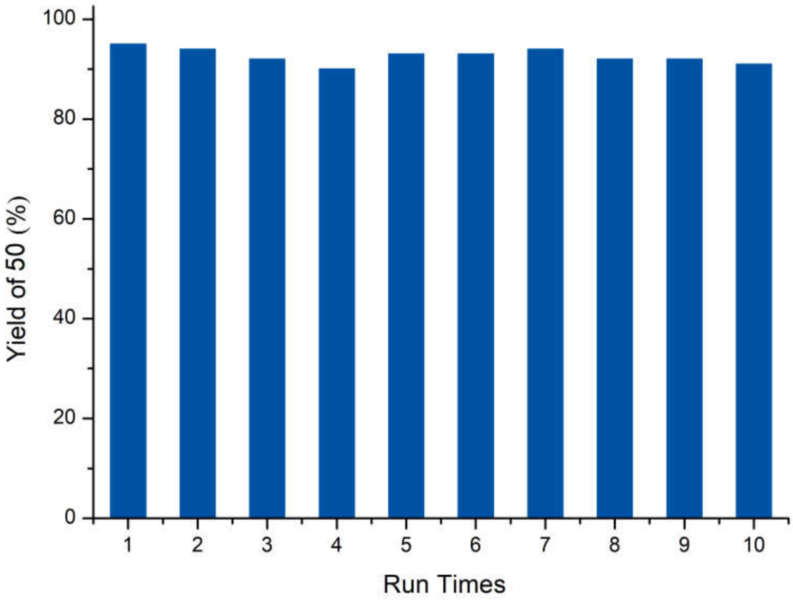

Supplementary Figure 5 | Yields of product **50** for recycling experiments.

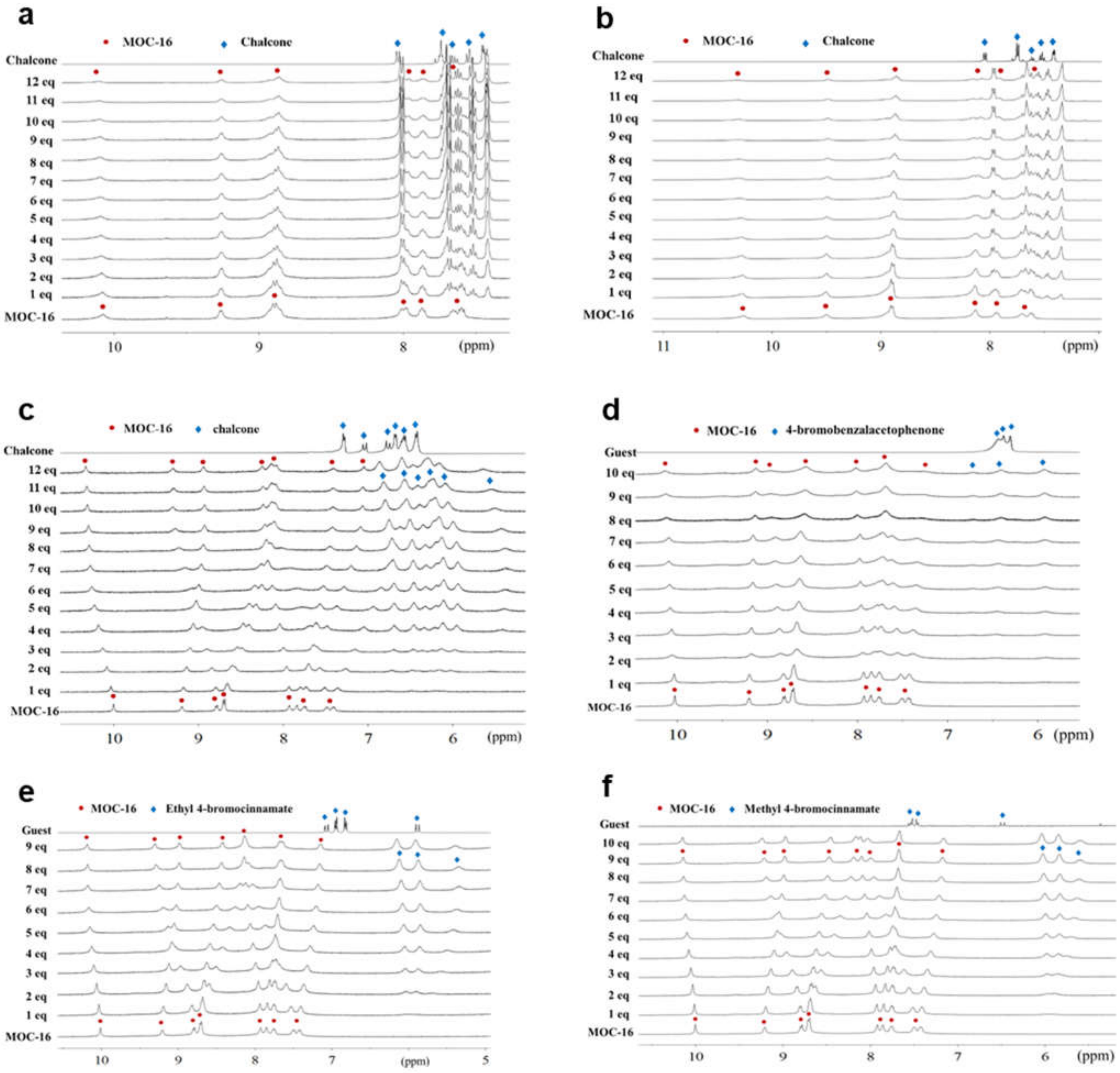

Supplementary Figure 6 | ^{1}H NMR titrations (400 MHz, 298 K) for guest capture. a, Addition of 1 to 12 eq aliquots of chalcone into an MOC-16 solution in acetonitrile-d_3-D_2O (2: 1, v: v). **b,** Addition of 1 to 12 eq aliquots of chalcone into an MOC-16 solution in acetone-d_3-D_2O (2: 1, v: v). **c,** Addition of 1 to 12 eq aliquots of chalcone into an MOC-16 solution in DMSO-d_6-D_2O (1: 3, v: v). **d,** Addition of 1 to 10 eq aliquots of ethyl 4-bromobenzalacetophenone into an MOC-16 solution in DMSO-d_6-D_2O (1: 3, v: v). **e,** Addition of 1 to 9 eq aliquots of 4-bromocinnamate into an MOC-16 solution in DMSO-d_6-D_2O (1: 3, v: v). **f,** Addition of 1 to 10 eq aliquots of methyl 4-bromocinnamate into an MOC-16 solution in DMSO-d_6-D_2O (1: 3, v: v). The ^{1}H NMR were measured in similar conditions, in case of not well soluble, the portion of organic solvent was slightly increased.

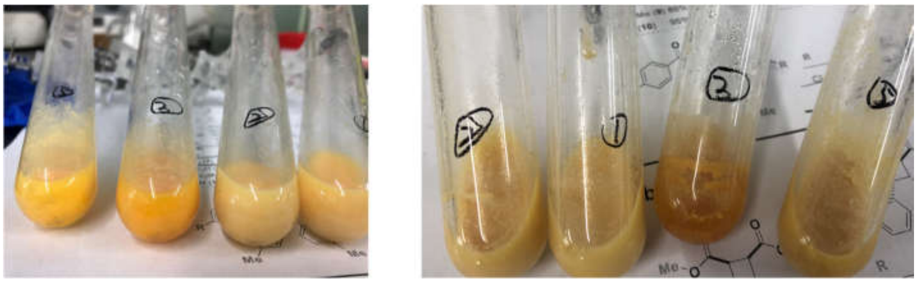

Supplementary Figure 7 | Photos of exemplified reaction mixtures. left, before reaction; **right,** after reaction.

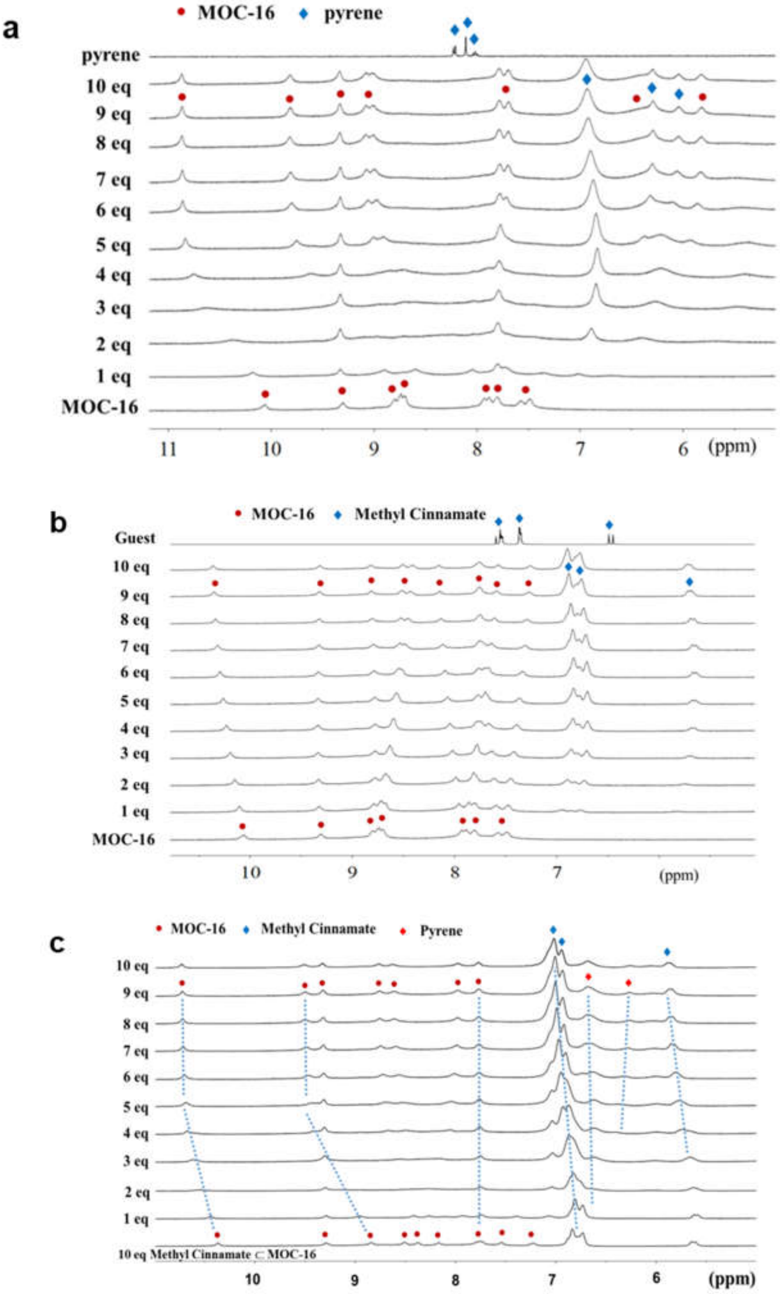

Supplementary Figure 8 | ^{1}H NMR titrations (400 MHz, 298 K) for guest competition. a, Addition of 1 to 10 eq aliquots of pyrene into an MOC-16 solution in DMSO-d_6-D_2O (1: 1, v: v). **b,** Addition of 1 to 10 eq

aliquots of methyl cinnamate in an MOC-16 solution in DMSO-d_6-D_2O (1: 1, v: v). **c,** Addition of 1 to 10 eq aliquots of pyrene into an MOC-16 solution containing 10 eq methyl cinnamate in DMSO-d_6-D_2O (1: 1, v: v).

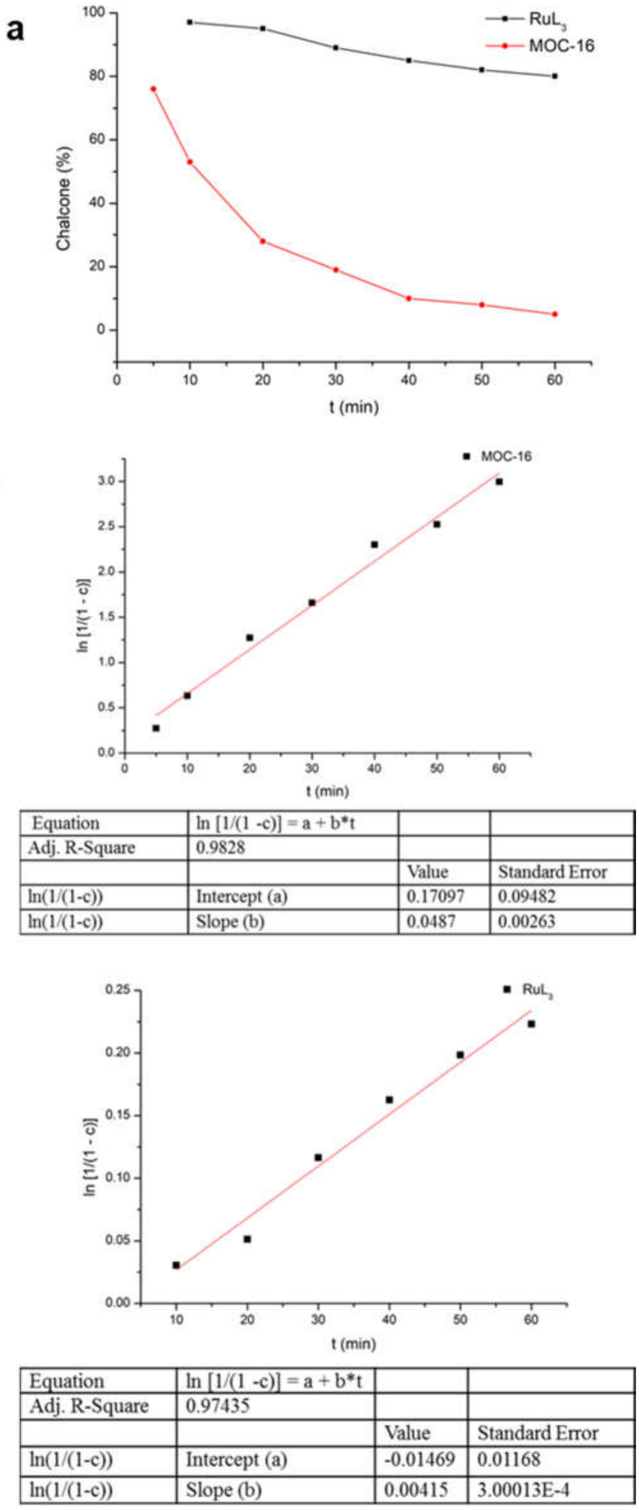

Equation	ln [1/(1 -c)] = a + b*t		
Adj. R-Square	0.9828		
		Value	Standard Error
ln(1/(1-c))	Intercept (a)	0.17097	0.09482
ln(1/(1-c))	Slope (b)	0.0487	0.00263

Equation	ln [1/(1 -c)] = a + b*t		
Adj. R-Square	0.97435		
		Value	Standard Error
ln(1/(1-c))	Intercept (a)	-0.01469	0.01168
ln(1/(1-c))	Slope (b)	0.00415	3.00013E-4

Supplementary Figure 9 | Kinetic study for chalcone photoreactions using MOC-16 and RuL_3 as the catalyst. a, Conversions of chalcone in 1 h. **b,** Reaction order simulation showing apparent first-order rate of k^1(MOC-16) = 0.0487 min^{-1}. **c,** Reaction order simulation showing apparent first-order rate of k^1(RuL_3) = 0.00415 min^{-1}.

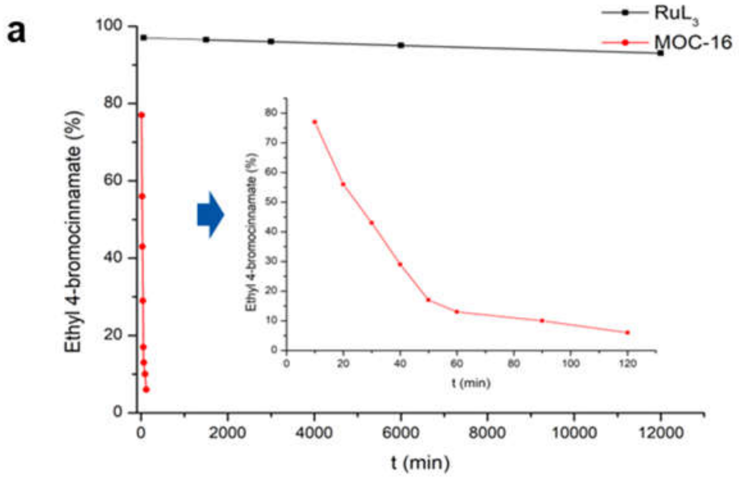

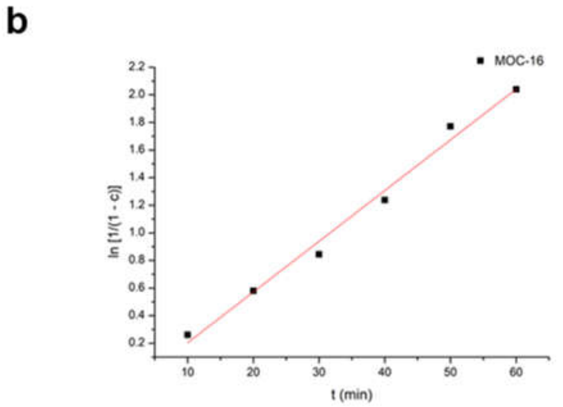

Equation	ln [1/(1 -c)] = a + b*t		
Adj. R-Square	0.98604		
		Value	Standard Error
ln(1/(1-c))	Intercept (a)	-0.16393	0.07605
ln(1/(1-c))	Slope (b)	0.03676	0.00195

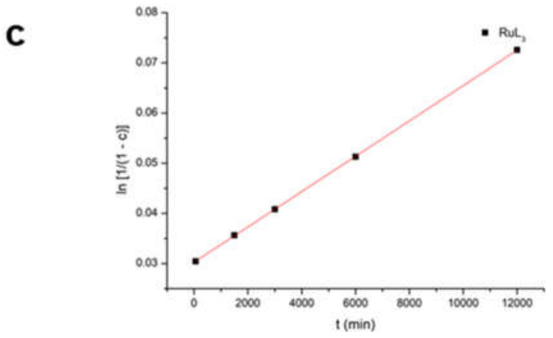

Equation	ln [1/(1 -c)] = a + b*t		
Adj. R-Square	0.99998		
		Value	Standard Error
ln(a/(a-x2))	Intercept (a)	0.03026	5.22003E-5
ln(a/(a-x2))	Slope (b)	3.5221E-6	8.44021E-9

Supplementary Figure 10 | Kinetic study for ethyl 4-bromocinnamate photoreactions using MOC-16 and RuL_3 as the catalyst. a, Conversions of ethyl 4-bromocinnamate in certain times. **b,** Reaction order simulation showing apparent first-order rate of k^2(MOC-16) = 0.03676 min^{-1}. **c,** Reaction order simulation showing apparent first-order rate of $k^2(RuL_3)$ = 3.5221×10^{-6} min^{-1}.

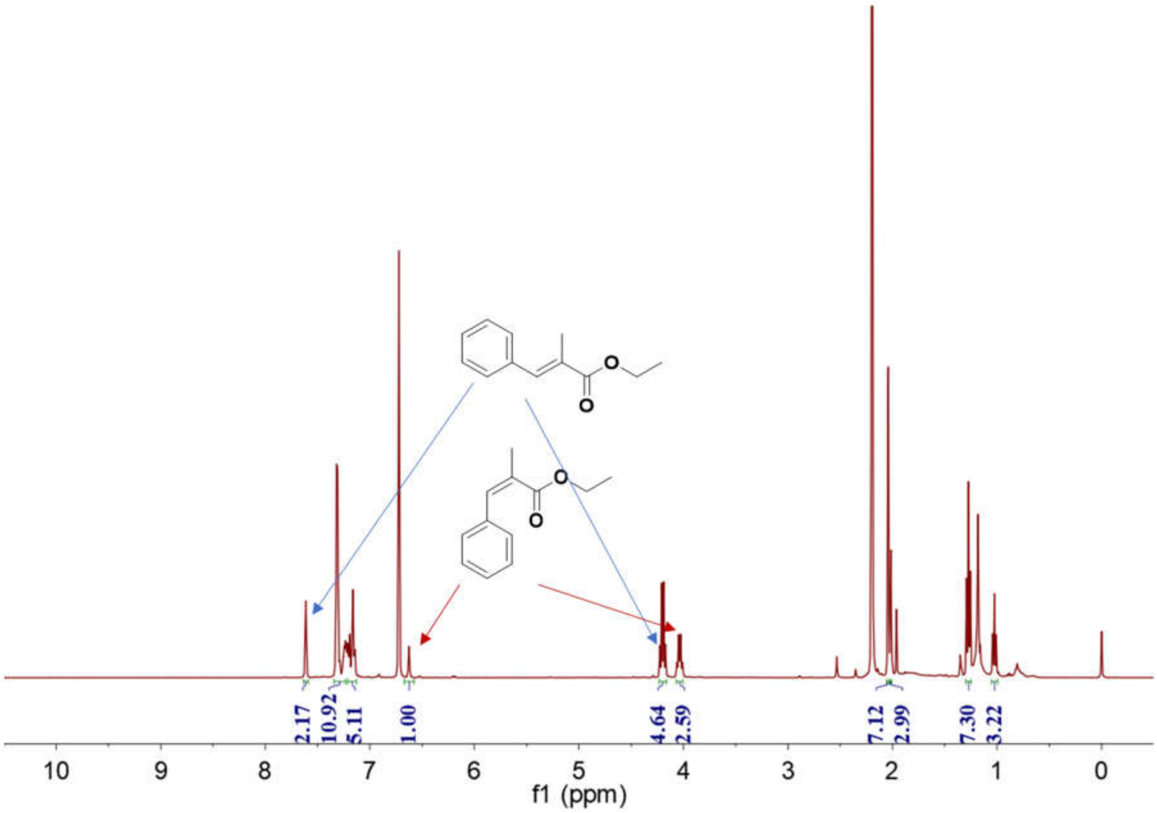

Supplementary Figure 11 | *E*-*Z* isomerization of ethyl (*E*)-2-methyl-3-phenylacrylate.

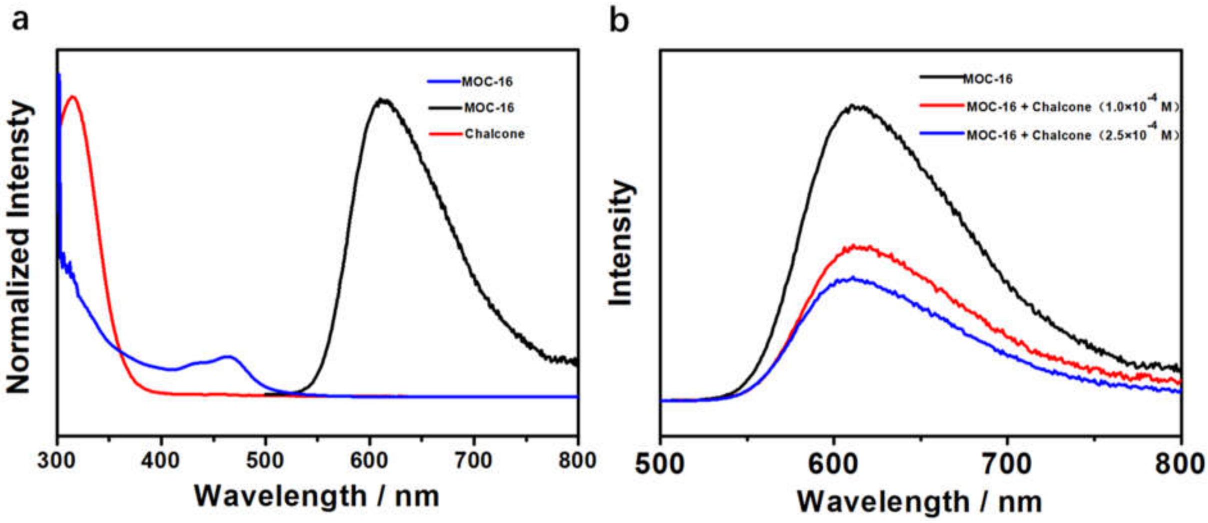

Supplementary Figure 12 | Absorption and emission spectra of MOC-16 and chalcone. a, The emission spectrum of MOC-16 (black line), and the absorption spectra of MOC-16 (blue line) and chalcone (red line); **b**, The emission quenching of MOC-16 by chalcone at 298 K. For **a**, **b**, excitation wavelength is 450 nm, the solvent is DMSO: H_2O (1: 3), MOC-16 = 1×10^{-5} M. For **a** [chalcone] = 1.0×10^{-4} M

Supplementary Tables

Supplementary Table 1 | Crystal data and structure refinement for cages.

Identification code	**MOC-16**	**Hex@MOC-16**	**Hep@MOC-16**
Empirical Formula	$C_{464}H_{328}B_{12}F_{84}N_{128}O_4P_6Pd_6Ru_8$	$C_{480}H_{376}B_{12}F_{84}N_{120}P_6Pd_6Ru_8$	$C_{488}H_{392}B_{12}F_{84}N_{120}P_6Pd_6Ru_8$
Formula weight	11119.03	11183.49	11295.69
T/K	150.01(10)	150.01(10)	150.01(10)
Crystal system	Tetragonal	Tetragonal	Tetragonal
Space group	*P*4/n	*P*4/n	*P*4/n
a/Å	30.6641(2)	30.5388(4)	30.4558(3)
b/Å	30.6641(2)	30.5388(4)	30.4558(3)
c/Å	34.4718(5)	34.7560(15)	34.8341(8)
α/°	90	90	90
β/°	90	90	90
γ/°	90	90	90
Volume/$Å^3$	32413.3(6)	32414.1(17)	32310.5(10)
Z	2	2	2
ρ_{calc}, g/cm^3	1.139	1.146	1.161
μ/mm^{-1}	3.567	3.560	3.576
Goodness-of-fit	1.027	0.964	1.035
R_1 [I≥2σ (I)]	0.0742	0.0922	0.0931
wR_2 (all data)	0.2446	0.3083	0.3044
CCDC No.	1982352	1982353	1982354
Data/restraints/ parameters	27161/234/1636	28201/251/1543	28174/281/1616

Supplementary Table 2 | Selected bond lengths (Å) and angles (°) for **MOC-16**.

Pd(3)-N(25)	2.017(5)	Pd(1)-N(30)^1	2.045(7)
Pd(3)-N(25)^1	2.017(5)	Pd(1)-N(20B)	2.093(14)
Pd(3)-N(25)^2	2.017(5)	Ru(1)-N(1)	2.054(7)
Pd(3)-N(25)^3	2.017(5)	Ru(1)-N(12)	2.057(6)
Ru(2)-N(21)	2.060(5)	Ru(1)-N(7)	2.067(7)
Ru(2)-N(16)	2.059(5)	Ru(1)-N(2)	2.080(6)
Ru(2)-N(26)	2.063(5)	Ru(1)-N(11)	2.079(7)
Ru(2)-N(22)	2.064(5)	Ru(1)-N(6)	2.082(6)
Ru(2)-N(17)	2.065(5)	Pd(2)-N(10)	2.086(9)
Ru(2)-N(27)	2.070(5)	Pd(2)-N(10)^2	2.086(9)
Pd(1)-N(20A)	1.984(15)	Pd(2)-N(10)^1	2.086(9)
Pd(1)-N(5)	2.030(5)	Pd(2)-N(10)^3	2.086(9)
Pd(1)-N(15)^2	2.040(8)		

N(25)-Pd(3)-N(25)[1]	89.996(3)	N(15)[2]-Pd(1)-N(30)[1]	173.6(2)
N(25)-Pd(3)-N(25)[2]	89.995(4)	N(5)-Pd(1)-N(20B)	172.5(8)
N(25)[1]-Pd(3)-N(25)[2]	179.0(3)	N(15)[2]-Pd(1)-N(20B)	85.2(8)
N(25)-Pd(3)-N(25)[3]	179.0(3)	N(30)[1]-Pd(1)-N(20B)	96.4(8)
N(25)[1]-Pd(3)-N(25)[3]	89.995(4)	N(1)-Ru(1)-N(12)	90.7(3)
N(25)[2]-Pd(3)-N(25)[3]	89.995(3)	N(1)-Ru(1)-N(7)	171.5(2)
N(21)-Ru(2)-N(16)	93.5(2)	N(12)-Ru(1)-N(7)	96.1(2)
N(21)-Ru(2)-N(26)	171.09(19)	N(1)-Ru(1)-N(2)	80.1(2)
N(16)-Ru(2)-N(26)	94.0(2)	N(12)-Ru(1)-N(2)	97.2(2)
N(21)-Ru(2)-N(22)	79.68(18)	N(7)-Ru(1)-N(2)	94.1(3)
N(16)-Ru(2)-N(22)	171.64(19)	N(1)-Ru(1)-N(11)	94.3(3)
N(26)-Ru(2)-N(22)	93.2(2)	N(12)-Ru(1)-N(11)	79.7(2)
N(21)-Ru(2)-N(17)	89.60(19)	N(7)-Ru(1)-N(11)	91.8(3)
N(16)-Ru(2)-N(17)	79.37(18)	N(2)-Ru(1)-N(11)	173.6(3)
N(26)-Ru(2)-N(17)	96.4(2)	N(1)-Ru(1)-N(6)	93.6(3)
N(22)-Ru(2)-N(17)	95.59(19)	N(12)-Ru(1)-N(6)	172.4(2)
N(21)-Ru(2)-N(27)	94.37(19)	N(7)-Ru(1)-N(6)	80.1(2)
N(16)-Ru(2)-N(27)	96.07(18)	N(2)-Ru(1)-N(6)	89.7(3)
N(26)-Ru(2)-N(27)	80.1(2)	N(11)-Ru(1)-N(6)	93.7(3)
N(22)-Ru(2)-N(27)	89.36(18)	N(10)-Pd(2)-N(10)[2]	89.64(3)
N(17)-Ru(2)-N(27)	174.15(19)	N(10)-Pd(2)-N(10)[1]	89.65(3)
N(20A)-Pd(1)-N(5)	172.0(7)	N(10)[2]-Pd(2)-N(10)[1]	171.0(4)
N(20A)-Pd(1)-N(15)[2]	95.1(7)	N(10)-Pd(2)-N(10)[3]	171.0(4)
N(5)-Pd(1)-N(15)[2]	89.5(2)	N(10)[2]-Pd(2)-N(10)[3]	89.65(3)
N(20A)-Pd(1)-N(30)[1]	86.4(7)	N(10)[1]-Pd(2)-N(10)[3]	89.65(3)
N(5)-Pd(1)-N(30)[1]	88.4(2)		

[1]+Y, 1/2-X, +Z; [2]1/2-Y, +X, +Z; [3]1/2-X, 1/2-Y, +Z

Supplementary Table 3 | Selected bond lengths (Å) and angles (°) for **8×Hex⊂MOC-16**.

Pd(1)-N(20)	1.971(14)	Pd(3)-N(30)	2.008(8)
Pd(1)-N(5)	2.004(10)	Ru(2)-N(21)	2.056(7)
Pd(1)-N(25)[1]	2.021(8)	Ru(2)-N(27)	2.065(9)
Pd(1)-N(10)[2]	2.028(7)	Ru(2)-N(17)	2.066(9)
Pd(3)-N(30)[1]	2.008(8)	Ru(2)-N(26)	2.078(7)
Pd(3)-N(30)[2]	2.008(8)	Ru(2)-N(22)	2.079(7)
Pd(3)-N(30)[3]	2.008(8)	Ru(2)-N(16)	2.094(8)
N(20)-Pd(1)-N(5)	174.7(3)	N(27)-Ru(2)-N(17)	171.5(3)
N(20)-Pd(1)-N(25)[1]	92.2(4)	N(21)-Ru(2)-N(26)	171.7(3)
N(5)-Pd(1)-N(25)[1]	89.3(4)	N(27)-Ru(2)-N(26)	79.6(3)
N(20)-Pd(1)-N(10)[2]	87.5(4)	N(17)-Ru(2)-N(26)	93.5(3)
N(5)-Pd(1)-N(10)[2]	90.4(3)	N(21)-Ru(2)-N(22)	79.1(3)
N(25)[1]-Pd(1)-N(10)[2]	173.6(3)	N(27)-Ru(2)-N(22)	89.7(3)

$N(30)^1$-Pd(3)-$N(30)^2$	179.1(5)	N(17)-Ru(2)-N(22)	95.9(3)
$N(30)^1$-Pd(3)-$N(30)^3$	89.997(5)	N(26)-Ru(2)-N(22)	96.0(3)
$N(30)^2$-Pd(3)-$N(30)^3$	89.997(5)	N(21)-Ru(2)-N(16)	95.6(3)
$N(30)^1$-Pd(3)-N(30)	90.000(5)	N(27)-Ru(2)-N(16)	94.8(3)
$N(30)^2$-Pd(3)-N(30)	89.993(5)	N(17)-Ru(2)-N(16)	80.2(3)
$N(30)^3$-Pd(3)-N(30)	179.1(5)	N(26)-Ru(2)-N(16)	89.7(3)
N(21)-Ru(2)-N(27)	93.6(3)	N(22)-Ru(2)-N(16)	173.3(3)
N(21)-Ru(2)-N(17)	93.8(4)		

^{1}+Y, -1/2-X, +Z; ^{2}-1/2-Y, +X, +Z; ^{3}-1/2-X, -1/2-Y, +Z

Supplementary Table 4 | Selected bond lengths (Å) and angles (°) for **8×Hep⊂MOC-16**.

Pd(1)-N(20B)	1.89(4)	Ru(2)-N(21)	2.060(8)
Pd(1)-N(30)	2.011(11)	Ru(2)-N(22)	2.067(7)
Pd(1)-$N(15)^1$	2.022(10)	Ru(2)-N(17)	2.080(7)
Pd(1)-N(5)	2.040(7)	Ru(2)-N(27)	2.089(7)
Pd(1)-N(20A)	2.110(12)	Ru(1)-N(11)	2.042(8)
Pd(3)-N(25)	2.047(7)	Ru(1)-N(2)	2.047(8)
Pd(3)-$N(25)^2$	2.047(7)	Ru(1)-N(7)	2.061(8)
Pd(3)-$N(25)^3$	2.047(7)	Ru(1)-N(12)	2.066(9)
Pd(3)-$N(25)^1$	2.047(7)	Ru(1)-N(1)	2.074(7)
Ru(2)-N(26)	2.044(9)	Ru(1)-N(6)	2.092(9)
Ru(2)-N(16)	2.058(8)		
N(20B)-Pd(1)-N(30)	72.0(13)	N(21)-Ru(2)-N(17)	89.4(3)
N(20B)-Pd(1)-$N(15)^1$	109.7(13)	N(22)-Ru(2)-N(17)	96.1(3)
N(30)-Pd(1)-$N(15)^1$	174.1(3)	N(26)-Ru(2)-N(27)	80.5(3)
N(20B)-Pd(1)-N(5)	160.5(13)	N(16)-Ru(2)-N(27)	95.8(3)
N(30)-Pd(1)-N(5)	88.6(4)	N(21)-Ru(2)-N(27)	94.5(3)
$N(15)^1$-Pd(1)-N(5)	89.4(3)	N(22)-Ru(2)-N(27)	89.5(3)
N(30)-Pd(1)-N(20A)	94.8(5)	N(17)-Ru(2)-N(27)	173.7(3)
$N(15)^1$-Pd(1)-N(20A)	86.4(5)	N(11)-Ru(1)-N(2)	172.1(3)
N(5)-Pd(1)-N(20A)	171.9(4)	N(11)-Ru(1)-N(7)	93.9(3)
N(25)-Pd(3)-$N(25)^2$	179.7(4)	N(2)-Ru(1)-N(7)	93.3(3)
N(25)-Pd(3)-$N(25)^3$	89.998(3)	N(11)-Ru(1)-N(12)	79.7(3)
$N(25)^2$-Pd(3)-$N(25)^3$	90.000(2)	N(2)-Ru(1)-N(12)	96.4(3)
N(25)-Pd(3)-$N(25)^1$	90.001(2)	N(7)-Ru(1)-N(12)	95.6(3)
$N(25)^2$-Pd(3)-$N(25)^1$	90.000(3)	N(11)-Ru(1)-N(1)	93.1(3)
$N(25)^3$-Pd(3)-$N(25)^1$	179.7(4)	N(2)-Ru(1)-N(1)	80.1(3)
N(26)-Ru(2)-N(16)	93.6(4)	N(7)-Ru(1)-N(1)	170.7(3)
N(26)-Ru(2)-N(21)	171.4(3)	N(12)-Ru(1)-N(1)	91.8(3)
N(16)-Ru(2)-N(21)	93.9(3)	N(11)-Ru(1)-N(6)	92.7(4)
N(26)-Ru(2)-N(22)	93.6(3)	N(2)-Ru(1)-N(6)	91.9(3)
N(16)-Ru(2)-N(22)	171.7(3)	N(7)-Ru(1)-N(6)	79.4(3)

N(21)-Ru(2)-N(22)	79.3(3)	N(12)-Ru(1)-N(6)	170.5(3)
N(26)-Ru(2)-N(17)	96.3(3)	N(1)-Ru(1)-N(6)	94.2(3)
N(16)-Ru(2)-N(17)	78.9(3)		

[1]1/2-Y, +X, +Z; [2]1/2-X, 1/2-Y, +Z; [3]+Y, 1/2-X, +Z

Supplementary Table 5 | Crystal data and structure refinement for photoproducts

Identification code	**1**	**70**
Empirical Formula	$C_{30}H_{24}O_2$	$C_{26}H_{24}O_4$
Formula weight	416.49	400.45
T/K	200.01(10)	180.00(10)
Crystal system	Monoclinic	Orthorhombic
Space group	$P2_1/c$	*P*bca
a/Å	9.4392(2)	18.8066(3)
b/Å	22.7068(4)	9.15020(10)
c/Å	11.5061(3)	24.1066(4)
α/°	90	90
β/°	110.736(2)	90
γ/°	90	90
Volume/Å^3	2306.40(9)	4148.36(11)
Z	4	8
ρ_{calc}, g/cm^3	1.199	1.282
μ/mm^{-1}	0.576	0.688
Goodness-of-fit	1.040	1.034
R_1 [I≥2σ (I)]	0.0388	0.0357
wR_2 (all data)	0.1037	0.0927
CCDC No.	1982355	1982356
Data/restraints/parameters	4033/0/385	4044/0/274

Supplementary Table 6 | Selected bond lengths (Å) and angles (°) for **1**.

O(2)-C(5)	1.2164(15)	C(25)-C(26)	1.402(2)
C(2)-C(5)	1.5019(17)	C(10)-C(9)	1.380(2)
C(2)-C(1)	1.5506(17)	C(7)-C(8)	1.377(2)
C(2)-C(3)	1.5678(16)	C(24)-C(23)	1.389(2)
C(6)-C(7)	1.3895(18)	C(24)-C(19)	1.3925(19)
C(6)-C(11)	1.3916(18)	C(9)-C(8)	1.380(2)
C(6)-C(5)	1.4949(17)	C(30)-C(29)	1.397(2)
C(12)-O(1)	1.2197(14)	C(19)-C(20)	1.390(2)
C(12)-C(13)	1.4889(17)	C(18)-C(17)	1.384(2)
C(12)-C(1)	1.5143(17)	C(14)-C(15)	1.374(2)
C(1)-C(4)	1.5386(16)	C(23)-C(22)	1.377(3)
C(13)-C(18)	1.3821(18)	C(20)-C(21)	1.388(2)

C(13)-C(14)	1.3933(18)	C(26)-C(27)	1.383(2)
C(4)-C(19)	1.5014(17)	C(17)-C(16)	1.376(2)
C(4)-C(3)	1.5779(18)	C(27)-C(28)	1.369(3)
C(11)-C(10)	1.388(2)	C(15)-C(16)	1.370(3)
C(3)-C(25)	1.5072(18)	C(22)-C(21)	1.376(3)
C(25)-C(30)	1.379(2)	C(28)-C(29)	1.378(3)
C(5)-C(2)-C(1)	115.47(10)	C(6)-C(5)-C(2)	118.29(10)
C(5)-C(2)-C(3)	111.01(10)	C(10)-C(11)-C(6)	120.09(13)
C(1)-C(2)-C(3)	88.78(9)	C(25)-C(3)-C(2)	117.70(10)
C(7)-C(6)-C(11)	119.29(12)	C(25)-C(3)-C(4)	117.71(10)
C(7)-C(6)-C(5)	118.11(11)	C(2)-C(3)-C(4)	87.57(9)
C(11)-C(6)-C(5)	122.55(11)	C(30)-C(25)-C(26)	118.30(13)
O(1)-C(12)-C(13)	119.90(11)	C(30)-C(25)-C(3)	124.01(12)
O(1)-C(12)-C(1)	119.30(11)	C(26)-C(25)-C(3)	117.65(13)
C(13)-C(12)-C(1)	120.55(10)	C(9)-C(10)-C(11)	119.81(14)
C(12)-C(1)-C(4)	116.30(10)	C(8)-C(7)-C(6)	120.37(14)
C(12)-C(1)-C(2)	123.46(10)	C(23)-C(24)-C(19)	120.88(14)
C(4)-C(1)-C(2)	89.60(9)	C(8)-C(9)-C(10)	120.32(14)
C(18)-C(13)-C(14)	118.71(13)	C(25)-C(30)-C(29)	120.75(16)
C(18)-C(13)-C(12)	122.83(11)	C(20)-C(19)-C(24)	117.98(13)
C(14)-C(13)-C(12)	118.43(12)	C(20)-C(19)-C(4)	119.79(12)
C(19)-C(4)-C(1)	120.56(10)	C(24)-C(19)-C(4)	122.23(12)
C(19)-C(4)-C(3)	122.20(11)	C(13)-C(18)-C(17)	120.39(14)
C(1)-C(4)-C(3)	88.84(9)	C(15)-C(14)-C(13)	120.43(15)
O(2)-C(5)-C(6)	120.47(11)	C(22)-C(23)-C(24)	120.12(16)
O(2)-C(5)-C(2)	121.24(11)	C(21)-C(20)-C(19)	121.03(16)

Supplementary Table 7 | Selected bond lengths (Å) and angles (°) for **70**

O(3)-C(19)	1.3379(15)	O(2)-C(19)	1.2034(16)
O(3)-C(21)	1.4477(16)	C(1)-C(2)	1.3904(17)
O(4)-C(4)	1.3729(14)	C(1)-C(7)	1.5017(16)
O(4)-C(24)	1.4242(18)	C(17)-C(9)	1.5095(16)
O(1)-C(17)	1.2178(15)	C(7)-H(7)	0.9800
C(19)-O(3)-C(21)	116.40(11)	C(9)-C(8)-C(7)	87.80(9)
C(4)-O(4)-C(24)	117.17(11)	C(12)-C(11)-C(8)	117.55(12)
C(2)-C(1)-C(7)	123.07(11)	O(1)-C(17)-C(9)	121.24(11)
C(6)-C(1)-C(7)	119.53(11)	C(10)-C(9)-C(8)	88.06(8)
C(7)-C(10)-C(9)	90.47(9)	C(10)-C(7)-C(8)	87.51(9)
O(2)-C(19)-O(3)	124.38(12)	C(17)-C(9)-H(9)	113.9
O(2)-C(19)-C(10)	124.77(11)	O(4)-C(4)-C(3)	124.83(11)

Supplementary Table 8 | Reactivity of MOC-16 and RuL_3 under different conditions[a]

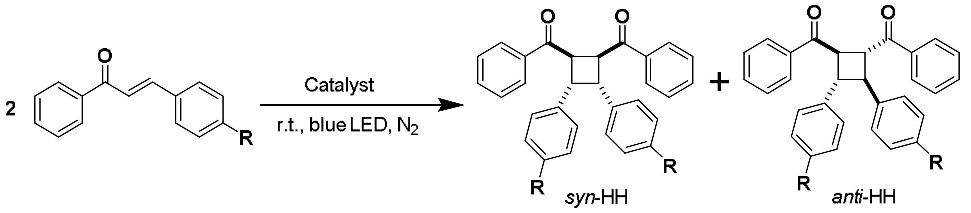

Entry	R	Solvent	t (h)	Catalyst (mol%)	Yield (d.r.)
1	H	Acetone (3 mL)	12	MOC-16 (2)	43% (1:6)
2	H	CH_3CN (3 mL)	12	MOC-16 (2)	35% (1:3)
3	H	Acetone-H_2O (2:1, 3 mL)	12	MOC-16 (2)	77% (1:6)
4	H	Acetone: H_2O (2: 1, 3 mL)	12	MOC-16 (0.08)	75% (1:6)
5	H	Acetone: H_2O (2: 1, 3 mL)	12	RuL_3 (0.64)	62% (1:9)
6	H	CH_3CN-H_2O (2:1, 3 mL)	12	MOC-16 (2)	75% (1:5)
7	H	CH_3CN: H_2O (2: 1, 3 mL)	12	MOC-16 (0.08)	70% (1: 5)
8	H	CH_3CN: H_2O (2: 1, 3 mL)	12	RuL_3 (0.64)	67% (1: 5)
9	H	DMSO-H_2O (1:4, 3 mL)	12	MOC-16 (0.5)	89% (1.4:1)
10	H	DMSO-H_2O (1:3, 3 mL)	12	MOC-16 (0.5)	95% (1.5:1)
11	H	DMSO-H_2O (1:2, 3 mL)	12	MOC-16 (0.5)	85% (1.2:1)
12	H	DMSO-H_2O (1:3, 3 mL)	3	MOC-16 (0.25)	93% (1.5:1)
13	H	DMSO-H_2O (1:3, 3 mL)	3	MOC-16 (0.03)	92% (1.5:1)
14	H	DMSO-H_2O (1:3, 3 mL)	3	MOC-16 (0.01)	57% (1:2)
15	H	DMSO-H_2O (1:3, 3 mL)	1	MOC-16 (0.03)	93%, 89%[b] (1.5:1)
16	H	DMSO: H_2O (1: 3, 3 mL)	1	RuL_3 (0.24)	23% (1: 3)
17	H	DMSO-H_2O (1:3, 3 mL)	1	-	N.D.
18[c]	H	DMSO-H_2O (1:3, 3 mL)	3	MOC-16 (0.03)	N.D.
19[d]	H	DMSO-H_2O (1:3, 3 mL)	3	MOC-16 (0.03)	14% (1:2)
20[e]	H	DMSO-H_2O (1:3, 3 mL)	3	MOC-16 (0.03)	4% (1:1)
21	OMe	DMSO: H_2O (1: 3, 3 mL)	10	RuL_3 (0.64)	70% (1.5: 1)
22	COOMe	DMSO: H_2O (1: 3, 3 mL)	10	RuL_3 (0.64)	20% (6: 1)
23	COOH	DMSO: H_2O (1: 3, 3 mL)	10	RuL_3 (0.64)	17% (1: 2)
24	I	DMSO: H_2O (1: 3, 3 mL)	10	RuL_3 (0.64)	N.D.
25	I	Acetone: H_2O (2: 1, 3 mL)	24	RuL3 (0.64)	18% (1.25: 1)
26	CF3	DMSO: H_2O (1: 3, 3 mL)	10	RuL3 (0.64)	trace
27	CF_3	Acetone: H_2O (2: 1, 3 mL)	24	RuL_3 (0.64)	12% (1: 1)

[a]Reaction conditions: chalcone (0.1 mmol), MOC-16 and solvent were stirred at room temperature (r.t.) under N_2 with irradiation by 24 W blue light-emitting diode (LED). Yields are determined by ^{1}H NMR using mesitylene as the internal standard. d.r. values were obtained by ^{1}H NMR analyses of reaction mixtures. N.D., not detected. [b]Isolated yield. [c]Without LED. [d]NEt_3 (0.02 mmol) was added. [e]1 eq TEMPO was added.

Supplementary Table 9 | Yields of the recycling experiments

Run	Yield (%)
1	95
2	94
3	92
4	90
5	93
6	93
7	94
8	92
9	92
10	91

Supplementary Table 10 | Yields and accumulative TONs of ten continuous photoreactions.

Run	Time (h)	Yield (%)	TON
1	2	96	96
2	4	85	170
3	6	84	252
4	8	87	348
5	10	81	405
6	12	85	510
7	14	90	630
8	16	84	672
9	18	90	810
10	20	91	910

Supplementary Table 11 | Kinetic data for chalcone photoreaction using MOC-16 as catalyst

Time (min)	$\ln(1/(1-c_1))$	$1/(1-c_1)$	$1-c_1$	c_1
5	0.27444	0.01316	0.76	0.24
10	0.63488	0.01887	0.53	0.47
20	1.27297	0.03571	0.28	0.72
30	1.66073	0.05263	0.19	0.81
40	2.30259	0.1	0.10	0.90
50	2.52573	0.125	0.08	0.92
60	2.99573	0.2	0.05	0.95

Supplementary Table 12｜Kinetic data for chalcone photoreaction using RuL_3 as catalyst

Time(min)	$\ln(1/(1-c_2))$	$1/(1-c_2)$	$1-c_2$	c_2
10	0.03046	1.030928	0.97	0.03
20	0.05129	1.052632	0.95	0.05
30	0.11653	1.123596	0.89	0.11
40	0.16252	1.176471	0.85	0.15
50	0.19845	1.219512	0.82	0.18
60	0.22314	1.25	0.80	0.20

Supplementary Table 13｜Kinetic data for ethyl 4-bromocinnamate using MOC-16 as catalyst

Time(min)	$\ln(1/(1-c_3))$	$1/(1-c_3)$	$1-c_3$	c_3
10	0.261365	1.298701	0.77	0.23
20	0.579818	1.785714	0.56	0.44
30	0.84397	2.325581	0.43	0.57
40	1.237874	3.448276	0.29	0.71
50	1.771957	5.882353	0.17	0.83
60	2.040221	7.692308	0.13	0.87
90	2.302585	10	0.10	0.90
120	2.813411	16.66667	0.06	0.94

Supplementary Table 14｜Kinetic data for ethyl 4-bromocinnamate using RuL_3 as catalyst

Time(min)	$\ln(1/(1-c_4))$	$1/(1-c_4)$	$1-c_4$	c_4
60	0.030459	1.030928	0.97	0.03
1500	0.035627	1.036269	0.965	0.035
3000	0.040822	1.041667	0.96	0.04
6000	0.051293	1.052632	0.95	0.05
12000	0.072571	1.075269	0.93	0.07

Supplementary Notes

Supplementary Note 1. Synthesis and characterization data for all products

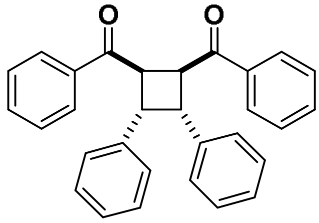

The compound **1** was prepared according to the general procedure A. Reaction time: 1 h. Isolated yield: 60% (White solid); d.r.: 1.5: 1 (*syn*-HH: *anti*-HH); total yield together with the *anti*-head-to-head isomer: 89%. ^{1}H NMR (400 MHz, $CDCl_3$) δ 7.82 (d, *J* = 7.6 Hz, 4H), 7.50 - 7.44 (m, 2H), 7.39 - 7.32 (m, 4H), 7.18 - 7.06 (m, 6H), 7.02 (d, *J* = 7.4 Hz, 4H), 4.81 - 4.74 (m, 2H), 4.48 - 4.39 (m, 2H). ^{13}C NMR (101 MHz, $CDCl_3$) δ 198.00, 139.20, 135.94, 133.11, 128.78, 128.30, 128.20, 128.14, 126.58, 49.27, 44.91. HRMS (ESI) Calcd. for $C_{30}H_{25}O_2$ $[M+H]^+$: 417.18491. Found: 417.18523.

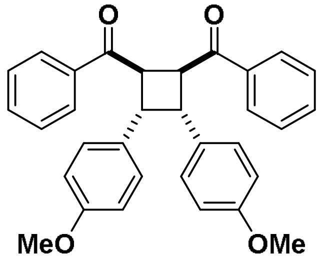

The compound **2** was prepared according to the general procedure A. Reaction time: 3 h. Isolated yield: 80% (White solid); d.r.: 12: 1 (*syn*-HH: *anti*-HH); total yield together with the *anti*-head-to-head isomer: 86%. ^{1}H NMR (400 MHz, $CDCl_3$) δ 7.81 (d, *J* = 7.8 Hz, 4H), 7.51 - 7.44 (m, 2H), 7.38 - 7.31 (m, 4H), 6.95 (d, *J* = 8.2 Hz, 4H), 6.72 (d, *J* = 8.2 Hz, 4H), 4.72 - 4.65 (m, 2H), 4.39-4.31 (m, 2H), 3.74 (s, 6H). ^{13}C NMR (101 MHz, $CDCl_3$) δ 198.16, 158.22, 136.00, 133.04, 131.42, 129.21, 128.75, 128.19, 113.76, 55.31, 49.67, 44.36. HRMS (APCI) Calcd. for $C_{32}H_{29}O_4$ $[M+H]^+$: 477.20604. Found: 477.20522.

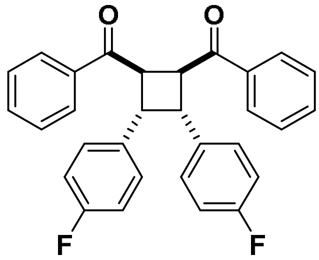

The compound **3** was prepared according to the general procedure B. Reaction time: 10 h. Isolated yield: 84% (White solid); d.r.: 12: 1 (*syn*-HH: *anti*-HH); total yield together with the *anti*-head-to-head isomer: 91%. ^{1}H NMR (400 MHz, $CDCl_3$) δ 7.80 (d, *J* = 7.8 Hz, 4H), 7.52 - 7.43 (m, 2H), 7.42 - 7.32 (m, 4H), 7.02 - 6.92 (m, 4H), 6.92 - 6.80 (m, 4H), 4.77 - 4.66 (m, 2H), 4.46 - 4.34 (m, 2H). ^{13}C NMR (101 MHz, $CDCl_3$) δ 197.69, 161.66 (d, *J* = 245.5 Hz), 135.76, 134.73 (d, *J* = 3.1 Hz), 133.26, 129.55 (d, *J* = 8.0 Hz), 128.83, 128.14, 115.34 (d, *J* = 21.3 Hz), 49.27, 44.18. ^{19}F NMR (377 MHz, $CDCl_3$) δ -116.45 (s, 2F). HRMS (APCI) Calcd. for $C_{30}H_{23}O_2F_2$ $[M+H]^+$: 453.16606. Found: 453.16683.

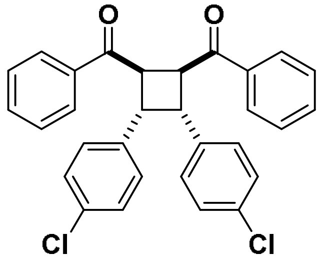

The compound **4** was prepared according to the general procedure C. Reaction time: 24 h. Isolated yield: 53% (White solid); no *anti*-head-to-head isomer was observed. ^{1}H NMR (400 MHz, Chloroform-*d*) δ 7.78 (d, *J* = 7.8 Hz, 4H), 7.51 - 7.45 (m, 2H), 7.41 - 7.33 (m, 4H), 7.16 (d, *J* = 8.0 Hz, 4H), 6.95 (d, *J* = 8.0 Hz, 4H), 4.72 - 4.67 (m, 2H), 4.44 - 4.36 (m, 2H). ^{13}C NMR (101 MHz, Chloroform-*d*) δ 197.51, 137.50, 135.67, 133.32, 132.64, 129.37, 128.85, 128.68, 128.12, 49.21, 44.18. HRMS (APCI) Calcd. for $C_{30}H_{23}O_2Cl_6$ $[M+H]^+$: 485.10696. Found: 485.10758.

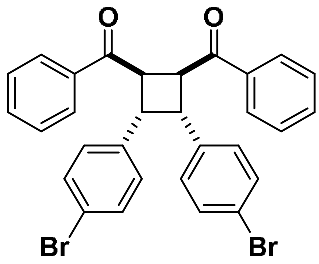

The compound **5** was prepared according to the general procedure B. Reaction time: 24 h. Isolated yield: 92% (White solid); no *anti*-head-to-head isomer was observed. ^{1}H NMR (400 MHz, $CDCl_3$) δ 7.78 (d, J = 7.8 Hz, 4H), 7.53 - 7.45 (m, 2H), 7.40 - 7.27 (m, 8H), 6.89 (d, J = 8.0 Hz, 4H), 4.72 - 4.66 (m, 2H), 4.43 - 4.34 (m, 2H). ^{13}C NMR (101 MHz, $CDCl_3$) δ 197.48, 138.02, 135.64, 133.33, 131.64, 129.73, 128.85, 128.12, 120.78, 49.19, 44.15. HRMS (APCI) Calcd. for $C_{30}H_{23}O_2Br_2$ $[M+H]^+$: 573.00593. Found: 573.00698.

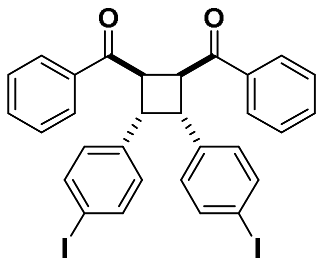

The compound **6** was prepared according to the general procedure C. Reaction time: 24 h. Isolated yield: Yield 92% (White solid); no *anti*-head-to-head isomer was observed. ^{1}H NMR (400 MHz, $CDCl_3$) δ 7.77 (d, J = 7.6 Hz, 4H), 7.62 - 7.44 (m, 6H), 7.43 - 7.31 (m, 4H), 6.77 (d, J = 8.0 Hz, 4H), 4.74 - 4.60 (m, 2H), 4.43 - 4.28 (m, 2H). ^{13}C NMR (101 MHz, $CDCl_3$) δ 197.46, 138.72, 137.59, 135.64, 133.32, 130.02, 128.85, 128.12, 92.31, 49.20, 44.21. HRMS (APCI) Calcd. for $C_{30}H_{23}O_2I_2$ $[M+H]^+$: 668.97819. Found: 668.97874.

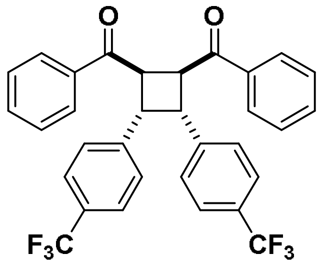

The compound **7** was prepared according to the general procedure C. Reaction time: 10 h. Isolated yield: 63% (White solid); d.r.: 13: 1 (*syn*-HH: *anti*-HH); total yield together with the *anti*-head-to-head isomer: 68%. ^{1}H NMR (400 MHz, $CDCl_3$) δ 7.79 (d, J = 7.6 Hz, 4H), 7.54 - 7.33 (m, 10H), 7.13 (d, J = 7.8 Hz, 4H), 4.82 - 4.75 (m, 2H), 4.58 - 4.51 (m, 2H). ^{13}C NMR (101 MHz, $CDCl_3$) δ 197.27, 142.98, 135.56, 133.48, 129.21 (q, J = 32.4 Hz), 128.92, 128.31, 128.14, 125.53, 122.77, 49.04, 44.48. ^{19}F NMR (377 MHz, $CDCl_3$) δ -63.06 (s, 6F). HRMS (APCI) Calcd. for $C_{32}H_{23}O_2F_6$ $[M+H]^+$: 553.15968. Found: 553.15899.

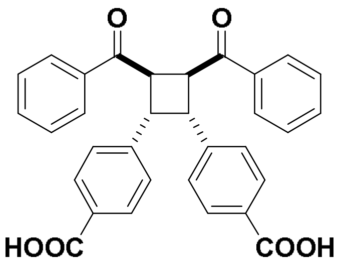

The compound **8** was prepared according to the general procedure B. Reaction time: 10 h. Isolated yield: Yield 83% (White solid); no *anti*-head-to-head isomer was observed. ^{1}H NMR (400 MHz, DMSO-d_6) δ 7.93 - 7.76 (m, 4H), 7.70 (d, J = 8.2 Hz, 4H), 7.58 - 7.50 (m, 2H), 7.47 - 7.38 (m, 4H), 7.32 (d, J = 8.2 Hz, 4H), 5.25 - 5.16 (m, 2H), 4.42 - 4.34 (m, 2H). ^{13}C NMR (101 MHz, DMSO-d_6) δ 198.06, 167.17, 144.19, 135.32, 133.22, 128.89, 128.75, 128.71, 128.38, 127.97, 47.10, 44.42. HRMS (APCI) Calcd. for $C_{32}H_{23}O_6$ [M-H]$^+$: 503.15001. Found: 503.15022.

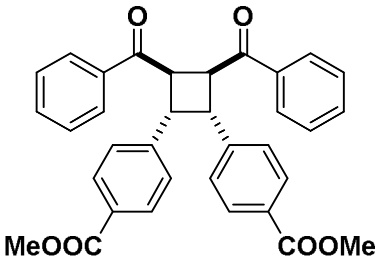

The compound **9** was prepared according to the general procedure B. Reaction time: 10 h. Isolated yield: Yield 85% (White solid); no *anti*-head-to-head isomer was observed. ^{1}H NMR (400 MHz, $CDCl_3$) δ 7.87 - 7.75 (m, 8H), 7.54 - 7.47 (m, 2H), 7.41 - 7.33 (m, 4H), 7.12 - 7.04 (m, 4H), 4.83 - 4.77 (m, 2H), 4.57 - 4.50 (m, 2H), 3.87 (s, 6H). ^{13}C NMR (101 MHz, $CDCl_3$) δ 197.38, 166.89, 144.25, 135.65, 133.37, 129.79, 128.87, 128.73, 128.15, 128.01, 52.19, 48.91, 44.80. HRMS (APCI) Calcd. for $C_{34}H_{29}O_6$ [M+H]$^+$: 533.19587. Found: 533.19658.

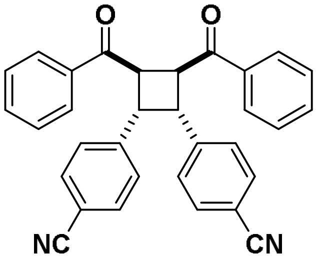

The compound **10** was prepared according to the general procedure B. Reaction time: 10 h. Isolated yield:

Yield 95% (White solid); no *anti*-head-to-head isomer was observed. ^{1}H NMR (400 MHz, $CDCl_3$) δ 7.80 - 7.72 (m, 4H), 7.54 - 7.48 (m, 6H), 7.40 - 7.34 (m, 4H), 7.18 - 7.08 (m, 4H), 4.79 - 4.75 (m, 2H), 4.59 - 4.53 (m, 2H). ^{13}C NMR (101 MHz, $CDCl_3$) δ 196.84, 144.21, 135.38, 133.65, 132.45, 128.98, 128.66, 128.10, 118.54, 111.12, 48.66, 44.67. HRMS (APCI) Calcd. for $C_{32}H_{23}O_2N_2$ $[M+H]^+$: 467.17540. Found: 467.17532.

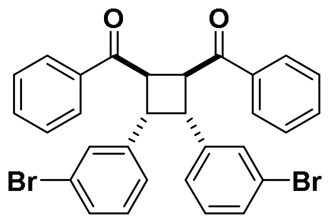

The compound **11** was prepared according to the general procedure B. Reaction time: 24 h. Isolated yield: 68% (White solid); d.r.: 4.8: 1 (*syn*-HH: *anti*-HH); total yield together with the *anti*-head-to-head isomer 82%. ^{1}H NMR (400 MHz, $CDCl_3$) δ 7.86 (d, J = 7.8 Hz, 4H), 7.62 - 7.52 (m, 2H), 7.48 - 7.40 (m, 4H), 7.34 (d, J = 8.0 Hz, 4H), 7.16 - 7.08 (m, 2H), 7.00 (d, J = 7.6 Hz, 2H), 4.82 - 4.76 (m, 2H), 4.51 - 4.41 (m, 2H). ^{13}C NMR (101 MHz, $CDCl_3$) δ 197.36, 141.12, 135.67, 133.35, 131.02, 130.04, 129.98, 128.87, 128.16, 126.80, 122.68, 48.73, 44.48. HRMS (APCI) Calcd. for $C_{30}H_{23}O_2Br_2$ $[M+H]^+$: 573.00593. Found: 573.00694.

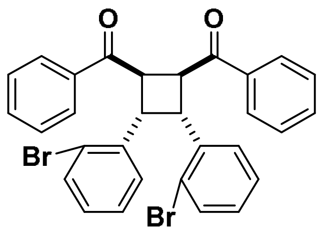

The compound **12** was prepared according to the general procedure B. Reaction time: 24 h. Isolated yield: 53% (White solid); no *anti*-head-to-head isomer was observed. ^{1}H NMR (400 MHz, $CDCl_3$) δ 7.79 (d, J = 7.6 Hz, 4H), 7.56 - 7.30 (m, 8H), 7.24 - 7.11 (m, 4H) , 7.07 - 6.94 (m, 2H), 5.05 - 4.91 (m, 2H), 4.79 - 4.71 (m, 2H). ^{13}C NMR (101 MHz, $CDCl_3$) δ 197.40, 138.37, 135.81, 133.27, 133.20, 128.77, 128.38, 128.21, 128.17, 127.06, 125.85, 48.94, 44.29. HRMS (APCI) Calcd. for $C_{30}H_{23}O_2Br_2$ $[M+H]^+$: 573.00593. Found: 573.00671.

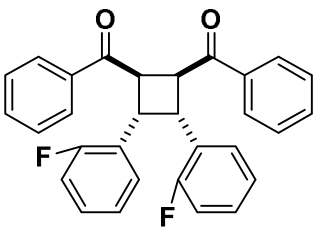

The compound **13** was prepared according to the general procedure A. Reaction time: 10 h. Isolated yield: 74% (White solid); d.r.: 4: 1 (*syn*-HH: *anti*-HH); total yield together with the *anti*-head-to-head isomer: 93%. ^{1}H NMR (400 MHz, $CDCl_3$) δ 7.81 (d, J = 7.6 Hz, 4H), 7.53 - 7.43 (m, 2H), 7.40 - 7.30 (m, 4H), 7.20 - 7.05(m, 4H), 7.02 - 6.95 (m, 2H), 6.90 - 6.77 (m, 2H), 5.00 - 4.90 (m, 2H), 4.70 - 4.60 (m, 2H). ^{13}C NMR (101 MHz, $CDCl_3$) δ 197.78, 160.97 (d, J = 246.1 Hz), 135.82, 133.15, 129.12, 128.78, 128.61 (d, J = 8.4 Hz), 128.13, 126.20 (d, J = 15.0 Hz), 123.88, 115.30 (d, J = 22.5 Hz), 47.90, 39.63. ^{19}F NMR (377 MHz, $CDCl_3$) δ -115.39 (m, 2H). HRMS (APCI) Calcd. for $C_{30}H_{23}O_2F_2$ $[M+H]^+$: 453.16606. Found: 453.16679.

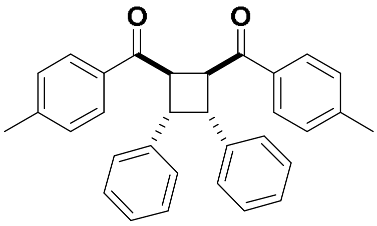

The compound **14** was prepared according to the general procedure A. Reaction time: 3 h. Isolated yield: 50% (White solid); d.r.: 1.5: 1 (*syn*-HH: *anti*-HH); total yield together with the *anti*-head-to-head isomer: 83%. ^{1}H NMR (400 MHz, $CDCl_3$) δ 7.72 (d, J = 7.8 Hz, 4H), 7.20 - 7.12 (m, 8H), 7.12 - 7.05 (m, 2H), 7.02 (d, J = 7.2 Hz, 4H), 4.80 - 4.70 (m, 2H), 4.50 - 4.40 (m, 2H), 2.35 (s, 6H).^{13}C NMR (101 MHz, $CDCl_3$) δ 197.62, 143.79, 139.41, 133.50, 129.42, 128.31, 128.24, 128.15, 126.47, 49.25, 44.91, 21.76. HRMS (APCI) Calcd. for $C_{32}H_{29}O_2$ $[M+H]^+$: 445.21621. Found: 445.21702.

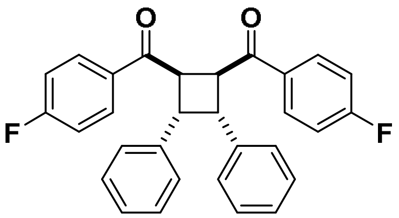

The compound **15** was prepared according to the general procedure A. Reaction time: 3 h. Isolated yield: 67.5% (White solid); d.r.: 3: 1 (*syn*-HH: *anti*-HH); total yield together with the *anti*-head-to-head isomer: 90%.

^{1}H NMR (400 MHz, $CDCl_3$) δ 7.84 - 7.79 (m, 4H), 7.19 - 7.08 (m, 6H), 7.07 - 6.97 (m, 8H), 4.75 - 4.69 (m, 2H) , 4.46 - 4.39 (m, 2H). ^{13}C NMR (101 MHz, $CDCl_3$) δ 196.49, 165.76 (d, *J* = 254.8 Hz), 138.90, 132.32 (d, *J* = 2.8 Hz), 130.77 (d, *J* = 9.2 Hz), 128.37, 128.08, 126.73, 115.95 (d, *J* = 21.9 Hz), 49.11, 44.93. ^{19}F NMR (377 MHz, $CDCl_3$) δ -105.55 (s, 2F). HRMS (APCI) Calcd. for $C_{30}H_{23}O_2F_2$ $[M+H]^+$: 453.16606. Found: 453.16674.

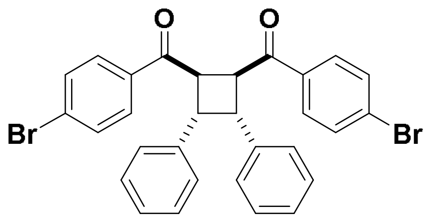

The compound **16** was prepared according to the general procedure B. Reaction time: 24 h. Isolated yield: 63% (White solid); d.r.: 2.5: 1 (*syn*-HH: *anti*-HH); total yield together with the *anti*-head-to-head isomer: 89%. ^{1}H NMR (400 MHz, $CDCl_3$) δ 7.68 - 7.62 (m, 4H), 7.54 - 7.48 (m, 4H), 7.18 - 7.09 (m, 6H), 7.02 - 6.98 (m, 4H), 4.73 - 4.66 (m, 2H), 4.43 - 4.38 (m, 2H). ^{13}C NMR (101 MHz, $CDCl_3$) δ 197.04, 138.77, 134.59, 132.16, 129.68, 128.40, 128.07, 126.79, 49.14, 44.92. HRMS (APCI) Calcd. for $C_{30}H_{21}O_2Br_2$ $[M-H]^+$: 570.99138. Found: 570.99206.

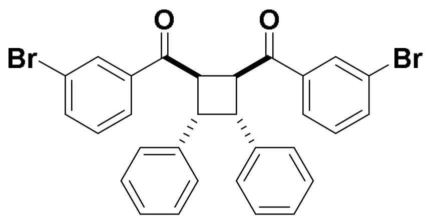

The compound **17** was prepared according to the general procedure B. Reaction time: 24 h. Isolated yield: 45% (White solid); d.r.: 2.7: 1 (*syn*-HH: *anti*-HH); total yield together with the *anti*-head-to-head isomer: 62%. ^{1}H NMR (400 MHz, $CDCl_3$) δ 7.95 - 7.90 (m, 2H), 7.73 - 7.68 (m, 2H), 7.65 - 7.59 (m, 2H), 7.29 - 7.22 (m, 2H), 7.20 - 7.10 (m, 6H), 7.03 - 6.98 (m, 4H), 4.74 - 4.69 (m, 2H), 4.46 - 4.42 (m, 2H). ^{13}C NMR (101 MHz, $CDCl_3$) δ 196.75, 138.75, 137.63, 136.11, 131.31, 130.39, 128.41, 128.10, 126.80, 126.68, 123.24, 49.26, 44.74. HRMS (APCI) Calcd. for $C_{30}H_{21}O_2Br_2$ $[M-H]^+$: 570.99138. Found: 570.99200.

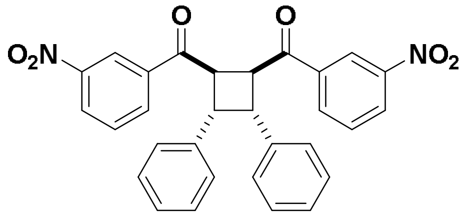

The compound **18** was prepared according to the general procedure B. Reaction time: 24 h. Isolated yield: 41% (White solid); no *anti*-head-to-head isomer was observed. ^{1}H NMR (400 MHz, $CDCl_3$) δ 8.52 - 8.42 (m, 2H), 8.31 - 8.21 (m, 2H), 8.05 - 7.95 (m, 2H), 7.55 - 7.45 (m, 2H), 7.25 - 7.20 (m, 4H), 7.16 - 7.08 (m, 4H), 7.07 - 7.01 (m, 2H), 5.13 - 5.03 (m, 2H), 4.83 - 4.75 (m, 2H). ^{13}C NMR (101 MHz, $CDCl_3$) δ 196.59, 148.14, 138.20, 137.71, 133.70, 129.65, 128.75, 128.16, 127.72, 127.28, 123.26, 51.40, 42.34. HRMS (APCI) Calcd. for $C_{30}H_{21}O_6N_2$ $[M+H]^+$: 505.14051. Found: 505.14083.

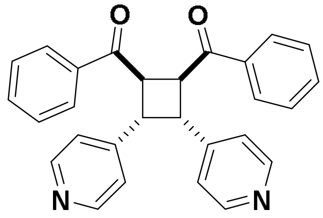

The compound **19** was prepared according to the general procedure B. Reaction time: 10 h. Isolated yield: 92% (White solid); no *anti*-head-to-head isomer was observed. ^{1}H NMR (400 MHz, $CDCl_3$) δ 8.43 (d, J = 4.8 Hz, 4H), 7.77 (d, J = 7.8 Hz, 4H), 7.56-7.47 (m, 2H), 7.42-7.33 (m, 4H), 6.95 (d, J = 4.8Hz, 4H), 4.82 - 4.74 (m, 2H), 4.50 - 4.43 (m, 2H). ^{13}C NMR (101 MHz, $CDCl_3$) δ 196.84, 150.11, 147.59, 135.37, 133.61, 128.96, 128.10, 123.03, 48.33, 43.69. HRMS (APCI) Calcd. for $C_{28}H_{23}O_2N_2$ $[M+H]^+$: 419.17540. Found: 419.17492.

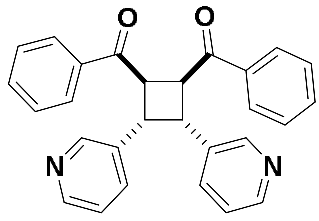

The compound **20** was prepared according to the general procedure B. Reaction time: 10 h. Isolated yield: 65% (Colorless oil); d.r.: 6: 1 (*syn*-HH: *anti*-HH); total yield together with the *anti*-head-to-head isomer: 75%. ^{1}H NMR (400 MHz, $CDCl_3$) δ 8.45 - 8.30 (m, 4H), 7.78 (d, J = 7.4 Hz, 4H), 7.55 - 7.48 (m, 2H), 7.45 - 7.27 (m, 6H), 7.13 - 7. 08 (m, 2H), 4.87 - 4.77 (m, 2H), 4.55 - 4.49 (m, 2H). ^{13}C NMR (101 MHz, $CDCl_3$) δ 197.08, 149.43, 148.49, 135.54, 134.11, 133.52, 128.95, 128.12, 123.47, 48.54, 42.36. HRMS (APCI) Calcd. for

$C_{28}H_{23}O_2N_2$ $[M+H]^+$: 419.17540. Found: 419.17507.

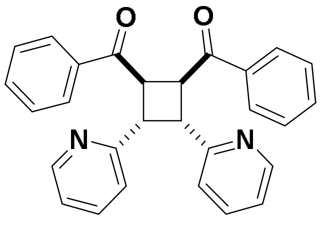

The compound **21** was prepared according to the general procedure B. Reaction time: 10 h. Isolated yield: 96% (White solid); no *anti*-head-to-head isomer was observed. ^{1}H NMR (400 MHz, $CDCl_3$) δ 8.41 (d, J = 4.0 Hz, 2H), 7.88 (d, J = 7.6 Hz, 4H), 7.49 - 7.28 (m, 8H), 7.05 - 6.80 (m, 4H), 5.50 - 5.40 (m, 2H), 4.60 - 4.46 (m, 2H). ^{13}C NMR (101 MHz, $CDCl_3$) δ 198.98, 158.09, 148.98, 136.10, 135.77, 132.90, 128.62, 128.29, 123.75, 121.38, 46.55, 45.99. HRMS (APCI) Calcd. for $C_{28}H_{23}O_2N_2$ $[M+H]^+$: 419.17540. Found: 419.17482.

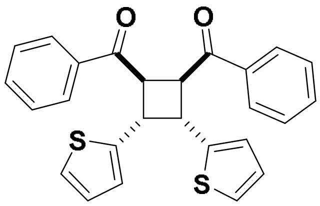

The compound **22** was prepared according to the general procedure B. Reaction time: 10 h. Isolated yield: 53% (Pale yellow oil); d.r.: 1.5: 1 (*syn*-HH: *anti*-HH); total yield together with the *anti*-head-to-head isomer: 88%. ^{1}H NMR (400 MHz, $CDCl_3$) δ 7.81 (d, J = 7.8 Hz, 4H), 7.54 - 7.44 (m, 2H), 7.40 - 7.30 (m, 4H), 7.15 (d, J = 4.8 Hz, 2H), 6.96 - 6.80 (m, 4H), 4.79 - 4.70 (m, 2H), 4.69 - 4.61 (m, 2H). ^{13}C NMR (101 MHz, $CDCl_3$) δ 197.25, 142.39, 135.84, 133.25, 128.79, 128.20, 126.92, 125.88, 124.80, 51.31, 41.48. HRMS (APCI) Calcd. for $C_{26}H_{21}O_2S_2$ $[M+H]^+$: 429.09775. Found: 429.09759.

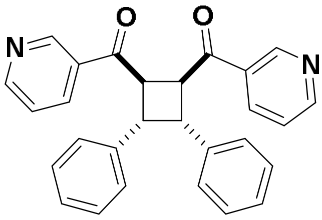

The compound **23** was prepared according to the general procedure B. Reaction time: 10 h. Isolated yield: 50% (Pale yellow oil); d.r.: 5: 1 (*syn*-HH: *anti*-HH); total yield together with the *anti*-head-to-head isomer:

60%. ^{1}H NMR (400 MHz, $CDCl_3$) δ 9.06 - 8.95 (m, 2H), 8.75 - 8.65 (m, 2H), 8.13 (d, J = 7.8 Hz, 2H), 7.40 - 7.32 (m, 2H), 7.23 - 7.05 (m, 6H), 7.04 - 6.95 (m, 4H), 4.82 - 4.74 (m, 2H), 4.49 - 4.40 (m, 2H). ^{13}C NMR (101 MHz, $CDCl_3$) δ 196.93, 153.71, 149.54, 138.21, 135.62, 131.14, 128.49, 128.02, 126.97, 124.00, 49.10, 44.92. HRMS (APCI) Calcd. for $C_{28}H_{23}O_2N_2$ $[M+H]^+$: 419.17540. Found: 419.17484.

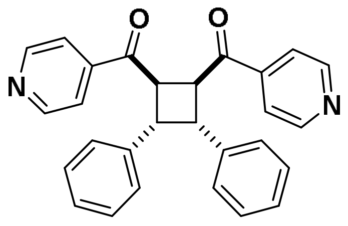

The compound **24** was prepared according to the general procedure B. Reaction time: 10 h. Isolated yield: 60% (Pale yellow oil); d.r.: 4: 1 (*syn*-HH: *anti*-HH); total yield together with the *anti*-head-to-head isomer: 75%. ^{1}H NMR (400 MHz, $CDCl_3$) δ 8.78 - 8.66 (m, 4H), 7.64 - 7.54 (m, 4H), 7.21 - 7.10 (m, 6H), 7.03 - 6.95 (m, 4H), 4.77 - 4.70 (m, 2H), 4.43 - 4.37 (m, 2H). ^{13}C NMR (101 MHz, $CDCl_3$) δ 197.59, 151.14, 141.55, 138.00, 128.51, 127.99, 127.07, 121.09, 49.11, 44.92. HRMS (APCI) Calcd. for $C_{28}H_{23}O_2N_2$ $[M+H]^+$: 419.17540. Found: 419.17584.

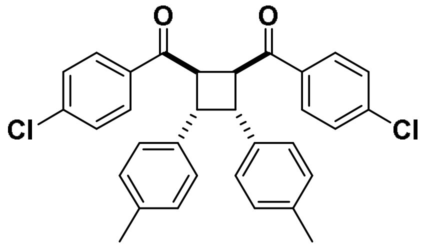

The compound **25** was prepared according to the general procedure C. Reaction time: 10 h. Isolated yield: 58.5% (White solid); d.r.: 3: 1 (*syn*-HH: *anti*-HH); total yield together with the *anti*-head-to-head isomer: 78%. ^{1}H NMR (400 MHz, Chloroform-*d*) δ 7.73 (d, J = 8.4 Hz, 4H), 7.34 (d, J = 8.2 Hz, 4H), 6.98 (d, J = 7.8 Hz, 4H), 6.89 (d, J = 7.8 Hz, 4H), 4.69 - 4.61 (m, 2H), 4.37 - 4.30 (m, 2H), 2.25(s, 6H). ^{13}C NMR (101 MHz, $CDCl_3$) δ 197.00, 139.54, 136.23, 135.88, 134.24, 129.58, 129.12, 127.99, 49.52, 44.63, 21.18. HRMS (APCI) Calcd. for $C_{32}H_{27}O_2Cl_2$ $[M+H]^+$: 513.13826. Found: 513.13782.

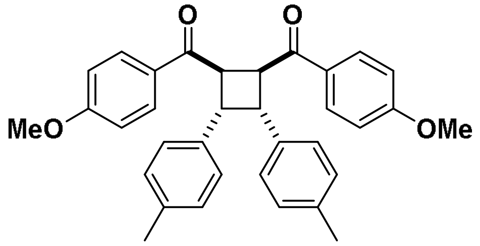

The compound **26** was prepared according to the general procedure C. Reaction time: 10 h. Isolated yield: 68% (White solid); d.r.: 3: 1 (*syn*-HH: *anti*-HH); total yield together with the *anti*-head-to-head isomer: 91%. ^{1}H NMR (400 MHz, Chloroform-*d*) δ ^{1}H NMR (400 MHz, CDCl3) δ 7.79 (d, *J* = 8.2 Hz, 4H), 7.70 - 6.88 (m, 8H), 6.83 (d, *J* = 8.2 Hz, 4H), 4.66 - 4.64 (m, 2H), 4.37 - 4.36 (m, 2H), 3.81 (s, 6H), 2.25 (s, 6H). ^{13}C NMR (101 MHz, Chloroform-*d*) δ 196.72, 163.33, 136.60, 135.81, 130.39, 129.21, 128.95, 128.05, 113.87, 55.54, 49.44, 44.61, 21.18. HRMS (APCI) Calcd. for $C_{34}H_{33}O_4$ [M+H]$^+$: 505.23734. Found: 505.23689.

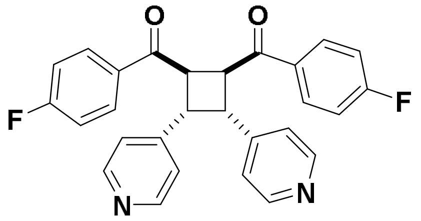

The compound **27** was prepared according to the general procedure B. Reaction time: 10 h. Isolated yield: 70% (White solid); d.r.: 8: 1 (*syn*-HH: *anti*-HH); total yield together with the *anti*-head-to-head isomer: 79%. ^{1}H NMR (400 MHz, $CDCl_3$) δ 8.45 - 8.38 (m, 4H), 7.82 - 7.72 (m, 4H), 7.15 - 6.99 (m, 4H), 6.95 - 6.90 (m, 4H), 4.76 - 4.66 (m, 2H), 4.46 - 4.40 (m, 2H).^{13}C NMR (101 MHz, $CDCl_3$) δ 195.25, 165.99 (d, *J* = 256.1 Hz), 150.11, 147.36,131.78 (d, *J* = 2.9 Hz), 130.71 (d, *J* = 9.3 Hz), 122.95, 116.20 (d, *J* = 22.0 Hz), 48.14, 43.67. ^{19}F NMR (377 MHz, $CDCl_3$) δ -104.13 (s, 2F). HRMS (APCI) Calcd. for $C_{28}H_{21}O_2N_2F_2$ [M+H]$^+$: 455.15656. Found: 455.15723.

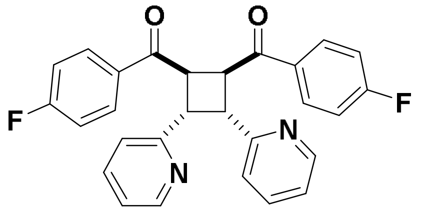

The compound **28** was prepared according to the general procedure B. Reaction time: 10 h. Isolated yield: 97% (White solid); no *anti*-head-to-head isomer was observed. ^{1}H NMR (400 MHz, $CDCl_3$) δ 8.41 (d, *J* = 4.0

Hz, 2H), 7.95 - 7.83 (m, 4H), 7.44 - 7.34 (m, 2H), 7.08 - 6.94 (m, 6H), 6.89 (d, J = 7.8 Hz, 2H), 5.47 - 5.36 (m, 2H), 4.62 - 4.46 (m, 2H). ^{13}C NMR (101 MHz, $CDCl_3$) δ 197.50, 165.68 (d, J = 254.4 Hz), 157.74, 149.02, 135.87, 132.55 (d, J = 2.8 Hz), 130.89 (d, J = 9.3 Hz), 123.81, 121.53, 115.78 (d, J = 21.9 Hz), 46.23, 45.92. ^{19}F NMR (377 MHz, $CDCl_3$) δ -106.02 (s, 2F). HRMS (APCI) Calcd. for $C_{28}H_{21}O_2N_2F_2$ $[M+H]^+$: 455.15656. Found: 455.15602.

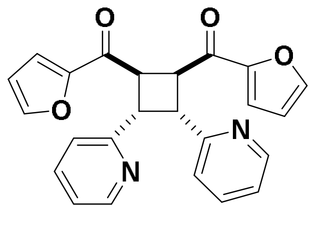

The compound **29** was prepared according to the general procedure B. Reaction time: 10 h. Isolated yield: 60% (Yellow solid); no *anti*-head-to-head isomer was observed. ^{1}H NMR (400 MHz, $CDCl_3$) δ 8.44 - 8.32 (m, 2H), 7.45 - 7.41 (m, 2H), 7.38 - 7.30 (m, 2H), 7.20 - 7.10 (m, 2H), 7.02 - 6.84 (m, 4H), 6.48 - 6.34 (m, 2H), 5.30 - 5.20 (m, 2H), 4.66 - 4.56 (m, 2H). ^{13}C NMR (101 MHz, $CDCl_3$) δ 187.88, 158.47, 152.45, 148.93, 146.13, 135.69, 123.61, 121.26, 117.05, 112.24, 46.01, 45.48. HRMS (APCI) Calcd. for $C_{24}H_{19}O_4N_2$ $[M+H]^+$: 399.13393. Found: 399.13341.

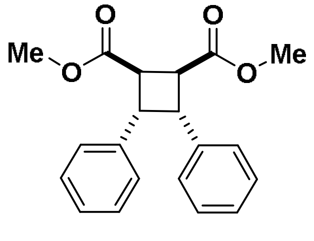

The compound **30** was prepared according to the general procedure A. Reaction time: 5 h. Isolated yield: 53% (Colorless oil); d.r.: 2.5: 1 (*syn*-HH: *anti*-HH); total yield together with the *anti*-head-to-head isomer: 75%. The compound **30** was reported (ref.: Pagire, S. K.; Hossain, A.; Traub, L.; Kerres, S.; Reiser, O. Photosensitised regioselective [2+2]-cycloaddition of cinnamates and related alkenes. *Chem. Commun.* **2017**, *53*, 12072-12075). ^{1}H NMR (400 MHz, $CDCl_3$) δ 7.16 - 6.99 (m, 6H), 6.99 - 6.85 (m, 4H), 4.45 - 4.35 (m, 2H), 3.88 - 3.79 (m, 2H), 3.75 (s, 6H). ^{13}C NMR (101 MHz, $CDCl_3$) δ 173.09, 138.61, 128.15, 127.91, 126.52, 52.32, 45.08, 43.40.

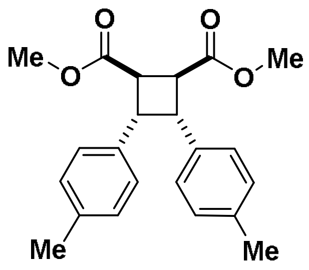

The compound **31** was prepared according to the general procedure A. Reaction time: 10 h. Isolated yield: 86% (Colorless oil); no *anti*-head-to-head isomer was observed. ^{1}H NMR (400 MHz, $CDCl_3$) δ 6.92 (d, J = 7.8 Hz, 4H), 6.84 - 6.79 (m, 4H), 4.36 - 4.30 (m, 2H), 3.82 - 3.77(m, 2H), 3.74 (s, 6H), 2.21 (s, 6H). ^{13}C NMR (101 MHz, $CDCl_3$) δ 173.19, 135.93, 135.73, 128.86, 127.81, 52.22, 44.72, 43.71, 21.09. HRMS (APCI) Calcd. for $C_{22}H_{25}O_4$ $[M+H]^+$: 353.17474. Found: 353.17492.

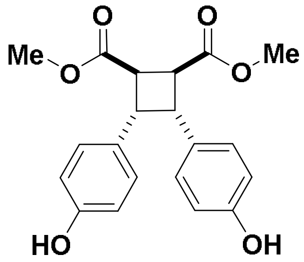

The compound **32** was prepared according to the general procedure B. Reaction time: 10 h. Isolated yield: 58.5% (Colorless oil); d.r.: 1.8: 1 (*syn*-HH: *anti*-HH); total yield together with the *anti*-head-to-head isomer: 91%. ^{1}H NMR (400 MHz, $CDCl_3$) δ 6.77 - 6.73 (m, 4H), 6.61 - 6.54 (m, 4H), 5.24 (bs, 2H), 4.28 - 4.22 (m, 2H), 3.77 - 3.71 (m, 8H). ^{13}C NMR (101 MHz, $CDCl_3$) δ 173.37, 154.24, 130.78, 129.16, 115.14, 52.35, 44.51, 43.63. HRMS (APCI) Calcd. for $C_{22}H_{21}O_6$ $[M+H]^+$: 357.13326. Found: 357.13330.

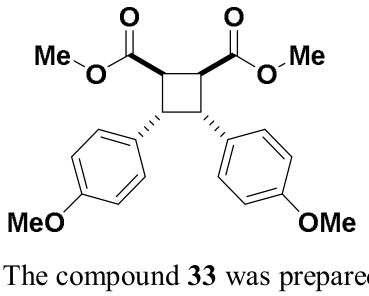

The compound **33** was prepared according to the general procedure B. Reaction time: 3 h. Isolated yield: 91% (Pale yellow oil); no *anti*-head-to-head isomer was observed. ^{1}H NMR (400 MHz, $CDCl_3$) δ 6.86 - 6.80 (m, 4H), 6.69 - 6.64 (m, 4H), 4.32 - 4.26 (m, 2H), 3.78 - 3.75(m, 2H), 3.74 (s, 6H), 3.71 (s, 6H). ^{13}C NMR (101 MHz, $CDCl_3$) δ 173.16, 158.15, 130.84, 128.95, 113.59, 55.24, 52.22, 44.47, 43.71. HRMS (APCI) Calcd. for

$C_{22}H_{25}O_6$ [M+H]$^+$: 385.16456. Found: 385.16483.

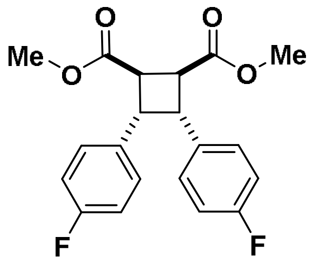

The compound **34** was prepared according to the general procedure B. Reaction time: 10 h. Isolated yield: 82% (White solid); no *anti*-head-to-head isomer was observed. ^{1}H NMR (400 MHz, $CDCl_3$) δ 6.93 - 6.57 (m, 8H), 4.38 - 4.31 (m, 2H), 3.79 - 3.75 (m, 2H), 3.75 (s, 6H). ^{13}C NMR (101 MHz, $CDCl_3$) δ 172.79, 161.61 (d, J = 245.4 Hz), 134.13 (d, J = 3.2 Hz), 129.34 (d, J = 8.0 Hz), 115.18 (d, J = 21.4 Hz), 52.39, 44.40, 43.48. ^{19}F NMR (377 MHz, $CDCl_3$) δ -116.05 (m, 2H). HRMS (APCI) Calcd. for $C_{20}H_{19}F_2O_4$ [M+H]$^+$: 361.12459. Found: 361.12491.

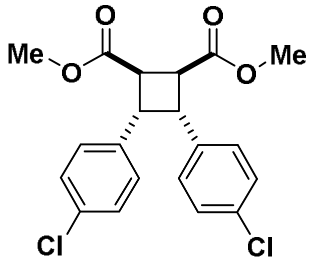

The compound **35** was prepared according to the general procedure B. Reaction time: 10 h. Isolated yield: 94% (White solid); no *anti*-head-to-head isomer was observed. ^{1}H NMR (400 MHz, $CDCl_3$) δ 7.11 (d, J = 7.6 Hz, 4H), 6.84 (d, J = 7.6 Hz, 4H), 4.41 - 4.31 (m, 2H), 3.82 - 3.68 (m, 8H). ^{13}C NMR (101 MHz, $CDCl_3$) δ 172.63, 136.85, 132.63, 129.17, 128.53, 52.42, 44.43, 43.42. HRMS (APCI) Calcd. for $C_{20}H_{19}Cl_2O_4$ [M+H]$^+$: 393.06549. Found: 393.06570.

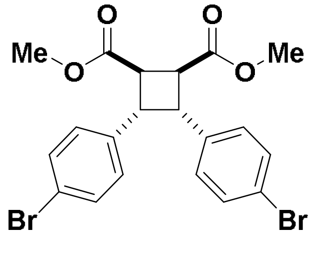

The compound **36** was prepared according to the general procedure B. Reaction time: 10 h. Isolated yield:

96% (White solid); no *anti*-head-to-head isomer was observed. ^{1}H NMR (400 MHz, $CDCl_3$) δ 7.30 - 7.20 (m, 4H), 6.81 - 6.73 (m, 4H), 4.36 - 4.30 (m, 2H), 3.78 - 3.70 (m, 8H). ^{13}C NMR (101 MHz, $CDCl_3$) δ 172.59, 137.36, 131.49, 129.53, 120.76, 52.44, 44.40, 43.39. HRMS (ESI) Calcd. for $C_{20}H_{18}Br_2O_4Na$ $[M+Na]^+$: 504.94436. Found: 504.94287.

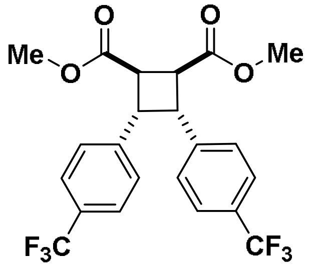

The compound **37** was prepared according to the general procedure B. Reaction time: 36 h. Isolated yield: 92% (White solid); no *anti*-head-to-head isomer was observed. ^{1}H NMR (400 MHz, $CDCl_3$) δ 7.38 (d, J = 8.2 Hz, 4H), 7.03 (d, J = 8.2 Hz, 4H), 4.55 - 4.45(m, 2H), 3.87 - 3.82 (m, 2H), 3.76 (s, 6H). ^{13}C NMR (101 MHz, $CDCl_3$) δ 172.40, 142.28, 129.18 (q, J = 32.5 Hz), 128.11, 125.35 (q, J = 3.8 Hz), 121.37 (q, J = 271.9 Hz), 52.51, 44.77, 43.31. ^{19}F NMR (377 MHz, $CDCl_3$) δ -63.12 (s, 6F). HRMS (APCI) Calcd. for $C_{22}H_{19}F_6O_4$ $[M+H]^+$: 461.11820. Found: 461.11854.

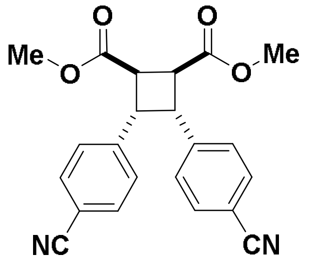

The compound **38** was prepared according to the general procedure B. Reaction time: 10 h. Isolated yield: 94% (White solid); no *anti*-head-to-head isomer was observed. ^{1}H NMR (400 MHz, $CDCl_3$) δ 7.43 (d, J = 8.0 Hz, 4H), 7.02 (d, J = 8.0 Hz, 4H), 4.52 - 4.45 (m, 2H), 3.84 - 3.78 (m, 2H), 3.77 (s, 6H). ^{13}C NMR (101 MHz, $CDCl_3$) δ 172.05, 143.43, 132.29, 128.46, 118.47, 111.06, 52.65, 44.99, 42.99. HRMS (APCI) Calcd. for $C_{22}H_{19}N_2O_4$ $[M+H]^+$: 375.13393. Found: 375.13431.

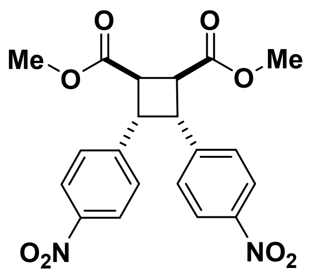

The compound **39** was prepared according to the general procedure B. Reaction time: 10 h. Isolated yield: 93% (White solid); no *anti*-head-to-head isomer was observed. ^{1}H NMR (400 MHz, $CDCl_3$) δ 8.10 - 7.95 (m, 4H), 7.15 - 7.06 (m, 4H), 4.62 - 4.54 (m, 2H), 3.91 - 3.85 (m, 2H), 3.79 (s, 6H). ^{13}C NMR (101 MHz, $CDCl_3$) δ 171.94, 146.92, 145.46, 128.55, 123.79, 52.69, 44.89, 43.18. HRMS (APCI) Calcd. for $C_{22}H_{19}N_2O_8$ $[M+H]^+$: 415.11359. Found: 415.11374.

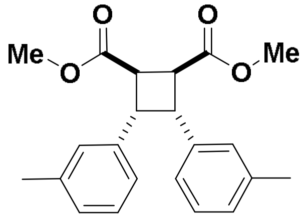

The compound **40** was prepared according to the general procedure A. Reaction time: 2 h. Isolated yield: 74% (Colorless oil); d.r.: 3.8: 1 (*syn*-HH: *anti*-HH); total yield together with the *anti*-head-to-head isomer: 94%. ^{1}H NMR (400 MHz, $CDCl_3$) δ 7.01 - 6.93 (m, 2H), 6.86 (d, *J* = 7.6 Hz, 2H), 6.79 - 6.50 (m, 4H), 4.38 - 4.30 (m, 2H), 3.86 - 3.80 (m, 2H), 3.75 (s, 6H), 2.18 (s, 6H). ^{13}C NMR (101 MHz, $CDCl_3$) δ 173.13, 138.54, 137.51, 128.80, 127.91, 127.18, 124.92, 52.24, 44.99, 43.43, 21.44. HRMS (APCI) Calcd. for $C_{22}H_{25}O_4$ $[M+H]^+$: 353.17474. Found: 353.17498.

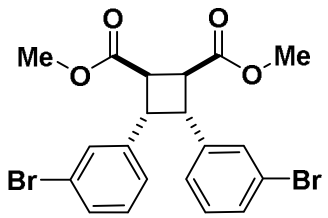

The compound **41** was prepared according to the general procedure B. Reaction time: 10 h. Isolated yield: 90% (White solid); no *anti*-head-to-head isomer was observed. ^{1}H NMR (400 MHz, $CDCl_3$) δ 7.22 (d, *J* = 7.8 Hz, 2H), 7.13 - 7.06 (m, 2H), 7.03 - 6.97 (m, 2H), 6.81 (d, *J* = 7.8 Hz, 2H), 4.37 - 4.30 (m, 2H), 3.80 - 3.77 (m, 2H), 3.76 (s, 6H). ^{13}C NMR (101 MHz, $CDCl_3$) δ 172.49, 140.49, 130.92, 129.95, 129.82, 126.52, 122.52,

52.46, 44.67, 43.10. HRMS (APCI) Calcd. for $C_{20}H_{19}Br_2O_4$ $[M+H]^+$: 480.96446. Found: 480.96494.

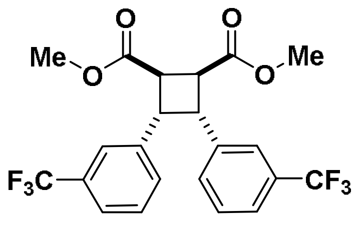

The compound **42** was prepared according to the general procedure B. Reaction time: 24 h. Isolated yield: 46% (White solid); d.r.: 4: 1 (*syn*-HH: *anti*-HH); total yield together with the *anti*-head-to-head isomer: 58%. ^{1}H NMR (400 MHz, $CDCl_3$) δ 7.32 (d, J = 7.8 Hz, 2H), 7.23 (d, J = 7.8 Hz, 2H), 7.11 - 7.06 (m, 4H), 4.52 - 4.47 (m, 2H), 3.93 - 3.86 (m, 2H), 3.78 (s, 6H). ^{13}C NMR (101 MHz, $CDCl_3$) δ 172.42, 138.96, 131.10, 130.73 (q, J = 32.3 Hz), 128.86, 124.57 (q, J = 3.9 Hz), 123.94 (q, J = 272.3 Hz), 123.67 (q, J = 3.9 Hz), 52.51, 44.85, 42.83. ^{19}F NMR (377 MHz, $CDCl_3$) δ -63.50 (s, 6H). HRMS (APCI) Calcd. for $C_{22}H_{19}O_4F_6$ $[M+H]^+$: 461.11820. Found: 461.11846.

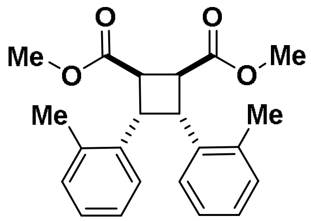

The compound **43** was prepared according to the general procedure A. Reaction time: 2 h. Isolated yield: 62% (White solid); d.r.: 2: 1 (*syn*-HH: *anti*-HH); total yield together with the *anti*-head-to-head isomer: 93%. ^{1}H NMR (400 MHz, $CDCl_3$) δ 7.10 - 6.89 (m, 8H), 4.62 - 4.55 (m, 2H), 3.84 - 3.75 (m, 2H), 3.75 (s, 6H), 2.12 (s, 6H). ^{13}C NMR (101 MHz, $CDCl_3$) δ 173.18, 137.02, 136.63, 130.28, 126.69, 126.29, 125.65, 52.29, 43.81, 41.51, 19.80. HRMS (APCI) Calcd. for $C_{22}H_{25}O_4$ $[M+H]^+$: 353.17474. Found: 353.17489.

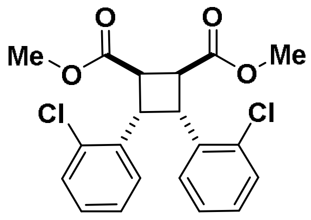

The compound **44** was prepared according to the general procedure B. Reaction time: 10 h. Isolated yield:

63% (White solid); d.r.: 2: 1 (*syn*-HH: *anti*-HH); total yield together with the *anti*-head-to-head isomer: 96%. ^{1}H NMR (400 MHz, $CDCl_3$) δ 7.23 - 7.14 (m, 2H), 7.10 - 6.97 (m, 6H), 4.96 - 4.84 (m, 2H), 3.85 - 3.77 (m, 2H), 3.76 (s, 6H). ^{13}C NMR (101 MHz, $CDCl_3$) δ 172.53, 136.22, 134.84, 129.68, 128.05, 127.70, 126.29, 52.39, 43.23, 41.98. HRMS (APCI) Calcd. for $C_{20}H_{19}Cl_2O_4$ $[M+H]^+$: 393.06549. Found: 393.06580.

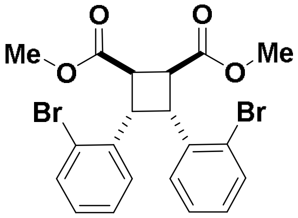

The compound **45** was prepared according to the general procedure B. Reaction time: 10 h. Isolated yield: 57% (White solid); d.r.: 1.7: 1 (*syn*-HH: *anti*-HH); total yield together with the *anti*-head-to-head isomer: 91%. ^{1}H NMR (400 MHz, $CDCl_3$) δ 7.43 - 7.38 (m, 2H), 7.14 - 7.05 (m, 2H), 7.03 - 6.94 (m, 4H), 4.93 - 4.86 (m, 2H), 3.89 - 3.67 (m, 8H). ^{13}C NMR (101 MHz, $CDCl_3$) δ 172.48, 137.92, 133.14, 128.34, 127.85, 126.95, 125.67, 52.41, 44.53, 43.45. HRMS (APCI) Calcd. for $C_{20}H_{19}Br_2O_4$ $[M+H]^+$: 480.96446. Found: 480.96476.

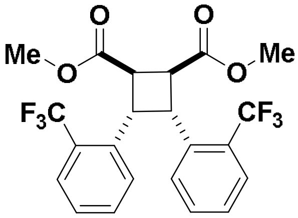

The compound **46** was prepared according to the general procedure B. Reaction time: 24 h. Isolated yield: 35% (White solid); d.r.: 3: 1 (*syn*-HH: *anti*-HH); total yield together with the *anti*-head-to-head isomer: 46%. ^{1}H NMR (400 MHz, $CDCl_3$) δ 7.60 - 7.50 (m, 2H), 7.48 - 7.32 (m, 2H), 7.29 - 7.21 (m, 4H), 4.94 - 4.87 (m, 2H), 3.90 - 3.80 (m, 2H), 3.75 (s, 6H). ^{13}C NMR (101 MHz, $CDCl_3$) δ 172.16, 137.47, 131.54, 129.29 (q, J = 30.1 Hz), 128.01, 127.07, 126.77 (q, J = 5.7 Hz), 124.08 (q, J = 274.3 Hz), 52.40, 44.43, 41.72. ^{19}F NMR (377 MHz, $CDCl_3$) δ -60.29 (s, 6F). HRMS (APCI) Calcd. for $C_{22}H_{19}O_4F_6$ $[M+H]^+$: 461.11820. Found: 461.11861.

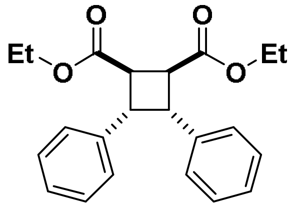

The compound **47** was prepared according to the general procedure B. Reaction time: 6 h. Isolated yield: 29% (Colorless oil); d.r.: 0.8: 1 (*syn*-HH: *anti*-HH); total yield together with the *anti*-head-to-head isomer: 66%. The compound **47** was reported (ref.: Pagire, S. K.; Hossain, A.; Traub, L.; Kerres, S.; Reiser, O. Photosensitised regioselective [2+2]-cycloaddition of cinnamates and related alkenes. *Chem. Commun.* **2017**, *53*, 12072-12075). ^{1}H NMR (400 MHz, $CDCl_3$) δ 7.16 - 6.98 (m, 6H), 6.97 - 6.85 (m, 4H), 4.50 - 4.28 (m, 2H), 4.21 (q, J = 7.2 Hz, 4H), 3.86 - 3.81 (m, 2H), 1.29 (t, J = 7.2 Hz, 6H). ^{13}C NMR (101 MHz, $CDCl_3$) δ 172.60, 138.86, 128.11, 127.95, 126.43, 61.12, 44.99, 43.60, 14.35.

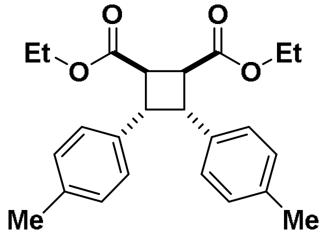

The compound **48** was prepared according to the general procedure B. Reaction time: 10 h. Isolated yield: 72% (Colorless oil); no *anti*-head-to-head isomer was observed. ^{1}H NMR (400 MHz, $CDCl_3$) δ 6.93 (d, J = 8.0 Hz, 4H), 6.86 - 6.80 (m, 4H), 4.48 - 4.28 (m, 2H), 4.19 (q, J = 7.2 Hz, 4H), 3.87 - 3.60 (m, 2H), 2.21 (s, 6H), 1.28 (t, J = 7.2Hz, 6H). ^{13}C NMR (101 MHz, $CDCl_3$) δ 172.67, 135.99, 135.82, 128.82, 127.85, 61.02, 44.63, 43.93, 21.10, 14.34. HRMS (ESI) Calcd. for $C_{24}H_{28}O_4Na$ $[M+Na]^+$: 403.18798. Found: 403.18698.

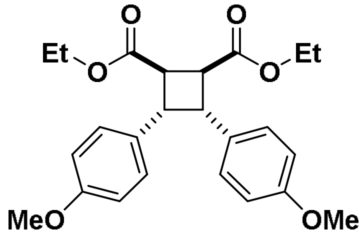

The compound **49** was prepared according to the general procedure B. Reaction time: 10 h. Isolated yield: 91% (Colorless oil); no *anti*-head-to-head isomer was observed. ^{1}H NMR (400 MHz, $CDCl_3$) δ 6.89 - 6.79 (m, 4H), 6.73 - 6.63 (m, 4H), 4.34 - 4.25 (m, 2H), 4.19 (q, J = 7.2 Hz, 4H), 3.77 - 3.71 (m, 2H), 3.70 (s, 6H), 1.27

(t, J = 7.2 Hz, 6H). ^{13}C NMR (101 MHz, $CDCl_3$) δ 172.66, 158.09, 131.10, 128.99, 113.55, 61.02, 55.24, 44.37, 43.91, 14.34. HRMS (ESI) Calcd. for $C_{24}H_{28}O_6Na$ $[M+Na]^+$: 435.17781. Found: 435.17694.

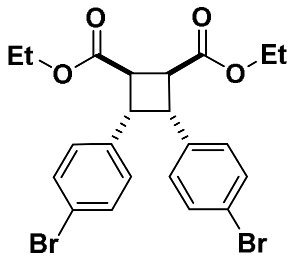

The compound **50** was prepared according to the general procedure B. Reaction time: 2 h. Isolated yield: 92% (White solid); no *anti*-head-to-head isomer was observed. ^{1}H NMR (400 MHz, $CDCl_3$) δ 7.18 (d, J = 7.0 Hz, 4H), 6.72 (d, J = 8.0 Hz, 4H)., 4.33 - 4.20 (m, 2H), 4.13 (q, J = 7.0 Hz, 4H), 3.71 - 3.60 (m, 2H), 1.20 (t, J = 7.2 Hz, 6H). ^{13}C NMR (101 MHz, $CDCl_3$) δ 172.08, 137.59, 131.43, 129.55, 120.65, 61.28, 44.33, 43.57, 14.31. HRMS (APCI) Calcd. for $C_{22}H_{23}Br_2O_4$ $[M+H]^+$: 508.99576. Found: 508.99557.

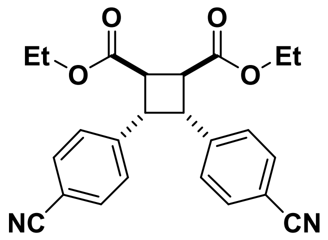

The compound **51** was prepared according to the general procedure B. Reaction time: 10 h. Isolated yield: 93% (Colorless oil); no *anti*-head-to-head isomer was observed. ^{1}H NMR (400 MHz, $CDCl_3$) δ 7.46 - 7.38 (m, 4H), 7.07 - 6.98 (m, 4H), 4.55 - 4.45 (m, 2H), 4.21 (q, J = 7.0 Hz, 4H), 3.85 - 3.75 (m, 2H), 1.28 (t, J = 7.0 Hz, 6H). ^{13}C NMR (101 MHz, $CDCl_3$) δ 171.56, 143.66, 132.25, 128.48, 118.50, 110.97, 61.60, 44.95, 43.17, 14.29. HRMS (ESI) Calcd. for $C_{24}H_{22}N_2O_4Na$ $[M+Na]^+$: 425.14718. Found: 425.14627.

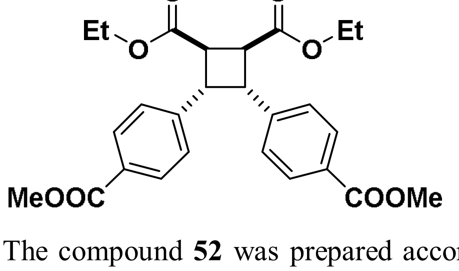

The compound **52** was prepared according to the general procedure B. Reaction time: 10 h. Isolated yield: 93% (Colorless oil); no *anti*-head-to-head isomer was observed. ^{1}H NMR (400 MHz, $CDCl_3$) δ 7.81 - 7.71 (m,

4H), 7.07 - 6.95 (m, 4H), 4.50 - 4.40 (m, 2H), 4.21 (q, J = 7.0 Hz, 4H), 3.91 - 3.75 (m, 8H), 1.28 (t, J = 7.0 Hz, 6H). ^{13}C NMR (101 MHz, CDCl3) δ 172.04, 166.84, 143.77, 129.59, 128.56, 127.81, 61.35, 52.13, 45.01, 43.35, 14.31. HRMS (ESI) Calcd. for $C_{26}H_{28}O_8Na$ $[M+Na]^+$: 491.16764. Found: 491.16681.

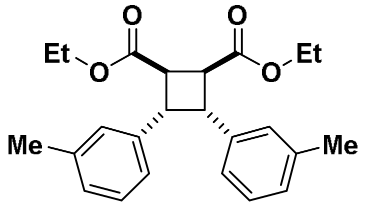

The compound **53** was prepared according to the general procedure B. Reaction time: 10 h. Isolated yield: 37% (Colorless oil); d.r.: 3: 1 (*syn*-HH: *anti*-HH); total yield together with the *anti*-head-to-head isomer: 50%. ^{1}H NMR (400 MHz, $CDCl_3$) δ 7.03 - 6.94 (m, 2H), 6.86 (d, J = 7.6 Hz, 2H), 6.79 - 6.64 (m, 4H), 4.38 - 4.30 (m, 2H), 4.21 (q, J = 7.2 Hz, 4H), 3.84 - 3.78 (m, 2H), 2.19 (s, 6H), 1.29 (t, J = 7.2 Hz, 6H). ^{13}C NMR (101 MHz, $CDCl_3$) δ 172.63, 138.79, 137.46, 128.84, 127.87, 127.09, 124.97, 61.03, 44.90, 43.63, 21.44, 14.34. HRMS (ESI) Calcd. for $C_{24}H_{28}O_4Na$ $[M+Na]^+$: 403.18798. Found: 403.18713.

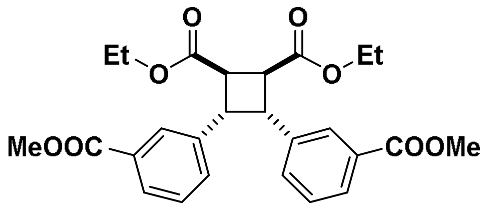

The compound **54** was prepared according to the general procedure B. Reaction time: 10 h. Isolated yield: 44% (Colorless oil); no *anti*-head-to-head isomer was observed. ^{1}H NMR (400 MHz, $CDCl_3$) δ 7.74 - 7.64 (m, 4H), 7.18 - 7.10 (m, 2H), 7.10 - 6.99 (m, 2H), 4.50 - 4.40 (m, 2H), 4.22 (q, J = 7.2 Hz, 4H), 3.90 - 3.86 (m, 2H), 3.85 (s, 6H), 1.29 (t, J = 7.2 Hz, 6H). ^{13}C NMR (101 MHz, $CDCl_3$) δ 172.17, 166.92, 138.82, 132.66, 130.09, 128.78, 128.32, 127.93, 61.28, 52.18, 44.80, 43.22, 14.32. HRMS (ESI) Calcd. for $C_{26}H_{28}O_8Na$ $[M+Na]^+$: 491.16764. Found: 491.16644.

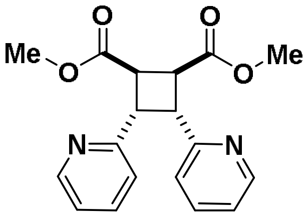

The compound **55** was prepared according to the general procedure B. Reaction time: 24 h. Isolated yield: 54% (White solid); no *anti*-head-to-head isomer was observed. ^{1}H NMR (400 MHz, $CDCl_3$) δ 8.35 - 8.27 (m, 2H), 7.38 - 7.30 (m, 2H), 6.94 - 6.85 (m, 4H), 4.52 - 4.44 (m, 2H), 4.40 - 4.34 (m, 2H), 3.75 (s, 6H). ^{13}C NMR (101 MHz, $CDCl_3$) δ 173.51, 158.23, 148.94, 135.69, 123.22, 121.27, 52.17, 46.28, 41.79. HRMS (APCI) Calcd. for $C_{18}H_{19}N_2O_4$ $[M+H]^+$: 327.13393. Found: 327.13423.

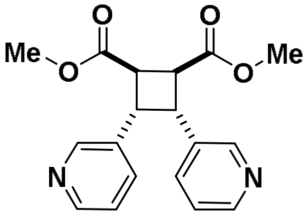

The compound **56** was prepared according to the general procedure B. Reaction time: 24 h. Isolated yield: 38% (Colorless oil); d.r.: 3.5: 1 (*syn*-HH: *anti*-HH); total yield together with the *anti*-head-to-head isomer: 50%. ^{1}H NMR (400 MHz, $CDCl_3$) δ 8.35 - 8.28 (m, 2H), 8.28 - 8.22 (m, 2H), 7.20 - 7.16 (m, 2H), 7.06 - 7.00 (m, 2H), 4.46 - 4.42 (m, 2H), 3.89 - 3.83 (m, 2H), 3.76 (s, 6H). ^{13}C NMR (101 MHz, $CDCl_3$) δ 172.24, 149.31, 148.38, 135.21, 133.50, 123.28, 52.55, 42.90, 42.65. HRMS (APCI) Calcd. for $C_{18}H_{19}N_2O_4$ $[M+H]^+$: 327.13393. Found: 327.13439.

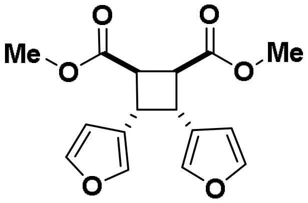

The compound **57** was prepared according to the general procedure B. Reaction time: 3 h. Isolated yield: 40% (Colorless oil); no *anti*-head-to-head isomer was observed. ^{1}H NMR (400 MHz, $CDCl_3$) δ 7.24 - 7.17 (m, 2H), 6.25 - 6.15 (m, 2H), 5.98 - 5.90 (m, 2H), 4.28 - 4.22 (m, 2H), 3.96 - 3.81 (m, 2H), 3.73 (s, 6H). ^{13}C NMR (101 MHz, $CDCl_3$) δ 172.44, 152.71, 141.90, 110.37, 107.13, 52.34, 43.24, 38.64. HRMS (APCI) Calcd. for $C_{16}H_{17}O_6$ $[M+H]^+$: 305.10196. Found: 305.10209.

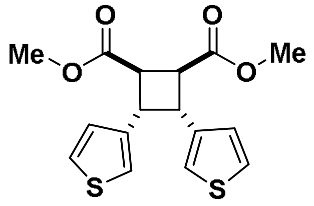

The compound **58** was prepared according to the general procedure B. Reaction time: 2 h. Isolated yield: 46% (Yellow oil); d.r.: 1.6: 1 (*syn*-HH: *anti*-HH); total yield together with the *anti*-head-to-head isomer: 74%. ^{1}H NMR (400 MHz, $CDCl_3$) δ 7.12 - 7.06 (m, 2H), 6.91 - 6.81(m, 2H), 6.75 - 6.68 (m, 2H), 4.55 - 4.51 (m, 2H), 3.86 - 3.78 (m, 2H), 3.74 (s, 6H).^{13}C NMR (101 MHz, $CDCl_3$) δ 172.24, 141.69, 126.73, 125.44, 124.77, 52.38, 45.80, 41.59. HRMS (APCI) Calcd. for $C_{16}H_{17}S_2O_4$ $[M+H]^+$: 337.05628. Found: 337.05657.

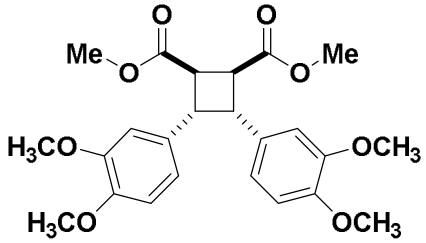

The compound **59** was prepared according to the general procedure B. Reaction time: 10 h. Isolated yield: 85% (Colorless oil); no *anti*-head-to-head isomer was observed. ^{1}H NMR (400 MHz, $CDCl_3$) δ 6.65 (d, J = 8.2 Hz, 2H), 6.58 - 6.52 (m, 2H), 6.34 - 6.30 (m, 2H), 4.35 - 4.20 (m, 2H), 3.78 - 3.74 (m, 8H), 3.73 (s, 6H), 3.61 (s, 6H). ^{13}C NMR (101 MHz, $CDCl_3$) δ 172.99, 148.59, 147.74, 131.23, 119.77, 111.72, 110.75, 55.86, 55.78, 52.23, 44.75, 43.59. HRMS (ESI) Calcd. for $C_{24}H_{28}O_8Na$ $[M+Na]^+$: 467.1676. Found: 467.1659.

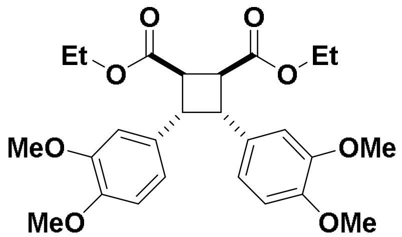

The compound **60** was prepared according to the general procedure B. Reaction time: 10 h. Isolated yield: 92% (write solid); no *anti*-head-to-head isomer was observed. ^{1}H NMR (400 MHz, $CDCl_3$) δ 6.65 (d, J = 8.2 Hz, 2H), 6.58 - 6.53 (m, 2H), 6.35 - 6.30 (m, 2H), 4.31 - 4.25(m, 2H), 4.18 (q, J = 7.2 Hz, 4H), 3.76 (s, 6H), 3.74 - 3.71 (m, 2H), 3.61 (s, 6H), 1.27 (t, J = 7.2 Hz, 6H). ^{13}C NMR (101 MHz, $CDCl_3$) δ 172.48, 148.58, 147.68, 131.51, 119.84, 111.77, 110.77, 61.02, 55.86, 55.78, 44.65, 43.79, 14.28. HRMS (ESI) Calcd. for

$C_{26}H_{32}O_8$ $[M+Na]^+$:495.1989. Found: 495.1982.

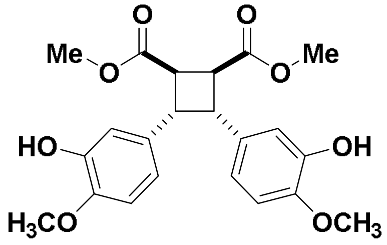

The compound **61** was prepared according to the general procedure B. Reaction time: 10 h. Isolated yield: 67% (Colorless oil); no *anti*-head-to-head isomer was observed. ^{1}H NMR (400 MHz, $CDCl_3$) δ 6.62 (d, J = 8.2 Hz, 2H), 6.56 - 6.48 (m, 2H), 6.48 - 6.42 (m, 2H), 5.43 (s, 2H), 4.26 - 4.22 (m, 2H), 3.78 (s, 6H), 3.75 - 3.70 (m, 8H). ^{13}C NMR (101 MHz, CDCl3) δ 173.14, 145.29, 145.17, 132.14, 119.61, 114.22, 110.38, 55.98, 52.25, 44.57, 43.70. HRMS (ESI) Calcd. for $C_{22}H_{23}O_8$ $[M-H]^+$: 415.1398. Found: 415.1405.

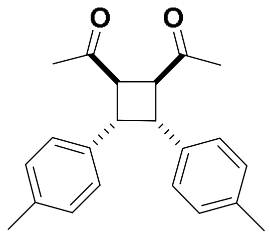

The compound **62** was prepared according to the general procedure B. Reaction time: 10 h. Isolated yield: 73% (write solid); d.r.: 5: 1 (*syn*-HH: *anti*-HH); total yield together with the *anti*-head-to-head isomer: 89%. ^{1}H NMR (400 MHz, $CDCl_3$) δ 6.94 (d, J = 8.0 Hz, 4H), 6.82 (d, J = 8.0 Hz, 4H), 4.14 - 4.08 (m, 2H), 3.89 - 3.82 (m, 2H), 2.22 (s, 6H), 2.17 (s, 6H). ^{13}C NMR (101 MHz, $CDCl_3$) δ 208.72, 136.20, 135.64, 128.98, 127.96, 51.35, 44.83, 29.39, 21.12. HRMS (ESI) Calcd. for $C_{22}H_{24}O_2$ $[M+Na]^+$: 343.1669. Found: 343.1663.

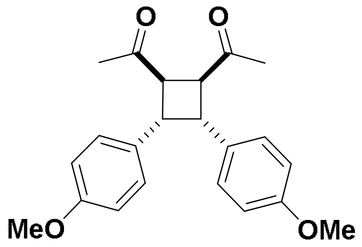

The compound **63** was prepared according to the general procedure B. Reaction time: 10 h. Isolated yield: 60% (write solid); d.r.: 8: 1 (*syn*-HH: *anti*-HH); total yield together with the *anti*-head-to-head isomer: 68%. ^{1}H NMR (400 MHz, $CDCl_3$) δ 6.88 - 6.80 (m, 4H), 6.70 - 6.64 (m, 4H), 4.10 - 4.05 (m, 2H), 3.84 - 3.80(m, 2H), 3.72 (s, 6H), 2.17 (s, 6H). ^{13}C NMR (101 MHz, $CDCl_3$) δ 208.68, 158.32, 130.73, 129.09, 113.71, 55.29,

51.37, 44.59, 29.40. HRMS (ESI) Calcd. for $C_{22}H_{24}O_4$ $[M+Na]^+$:375.1567. Found: 375.1562.

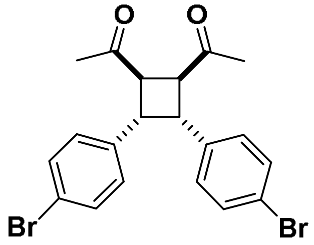

The compound **64** was prepared according to the general procedure B. Reaction time: 10 h. Isolated yield: 90% (write solid); no *anti*-head-to-head isomer was observed. ^{1}H NMR (400 MHz, $CDCl_3$) δ 7.24 - 7.17 (m, 4H), 6.72 (d, J = 8.2 Hz, 4H), 4.10 - 4.02 (m, 2H), 3.78 - 3.71 (m, 2H), 2.10 (s, 6H). ^{13}C NMR (101 MHz, $CDCl_3$) δ 207.64, 137.32, 131.61, 129.61, 120.95, 50.95, 44.32, 29.35. HRMS (ESI) Calcd. for $C_{20}H_{18}Br_2O_2$ $[M+Na]^+$: 470.9566. Found: 470.9557.

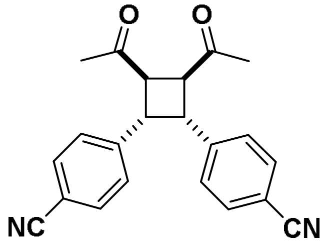

The compound **65** was prepared according to the general procedure B. Reaction time: 10 h. Isolated yield: 78% (write solid); no *anti*-head-to-head isomer was observed. ^{1}H NMR (400 MHz, $CDCl_3$) δ 7.48 - 7.41 (m, 4H), 7.05 - 7.00 (m, 4H), 4.31 - 4.26 (m, 2H), 3.91 - 3.87 (m, 2H), 2.20 (s, 6H). ^{13}C NMR (101 MHz, $CDCl_3$) δ 206.67, 143.44, 132.38, 128.53, 118.40, 111.24, 50.48, 44.69, 29.30. HRMS (ESI) Calcd. for $C_{22}H_{18}N_2O_2$ [M+Na]+: 365.1261. Found: 365.1254.

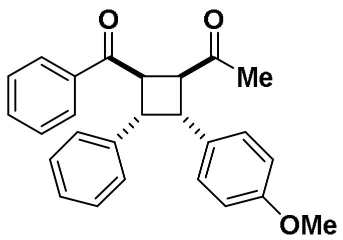

The compound **66** was prepared according to the general procedure E. Reaction time: 10 h. Isolated yield: 68% (write solid); d.r.: 20: 1 (*syn*-HH: *anti*-HH); total yield together with the *anti*-head-to-head isomer: 71%. ^{1}H NMR (400 MHz, $CDCl_3$) δ 7.91 - 7.82 (m, 2H), 7.58 - 7.52 (m, 1H), 7.47 - 7.39 (m, 2H), 7.22 - 7.10 (m,

3H), 7.02 - 6.98 (m, 2H), 6.87 - 6.81 (m, 2H), 6.71 - 6.62 (m, 2H), 4.72 (dd, *J* = 9.2, 5.0 Hz, 1H), 4.41 (t, *J* = 9.6 Hz, 1H), 4.07 (dd, *J* = 10.0, 5.0 Hz, 1H), 3.89 (t, *J* = 9.2 Hz, 1H), 3.70 (s, 3H), 2.14 (s, 3H). ^{13}C NMR (101 MHz, $CDCl_3$) δ 207.99, 198.89, 158.26, 138.60, 135.81, 133.42, 130.95, 128.94, 128.87, 128.44, 128.42, 128.27, 126.75, 113.61, 55.25, 51.28, 48.31, 45.50, 44.09, 28.56. HRMS (ESI) Calcd. for $C_{26}H_{24}O_3$ $[M+Na]^+$: 407.1617. Found: 407.1613.

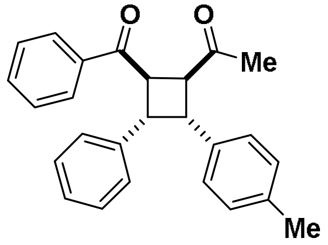

The compound **67** was prepared according to the general procedure E. Reaction time: 10 h. Isolated yield: 64% (write solid); d.r.: 17: 1 (*syn*-HH: *anti*-HH); total yield together with the *anti*-head-to-head isomer: 68%. ^{1}H NMR (400 MHz, $CDCl_3$) δ 7.87 (d, *J* = 7.4 Hz, 2H), 7.59 - 7.51 (m, 1H), 7.47 - 7.39 (m, 2H), 7.22 - 7.08 (m, 3H), 7.03 (d, *J* = 7.2 Hz, 2H), 6.93 (d, *J* = 7.8 Hz, 2H), 6.81 (d, *J* = 7.8 Hz, 2H), 4.71 (dd, *J* = 9.8, 5.2 Hz, 1H), 4.42 (t, *J* = 9.6 Hz, 1H), 4.10 (dd, *J* = 10.0, 5.0 Hz, 1H), 3.94 (t, *J* = 9.2 Hz, 1H), 2.22 (s, 3H), 2.14 (s, 3H). ^{13}C NMR (101 MHz, $CDCl_3$) δ 207.98, 198.88, 138.73, 136.10, 135.84, 135.78, 133.42, 128.90, 128.87, 128.44, 128.38, 128.28, 127.78, 126.72, 51.15, 48.50, 45.37, 44.40, 28.59, 21.11. HRMS (ESI) Calcd. for $C_{26}H_{24}O_2$ $[M+Na]^+$: 391.1669. Found: 391.1663.

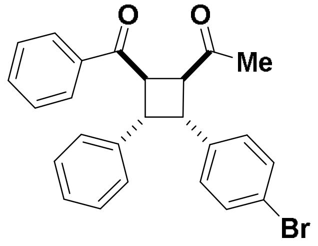

The compound **68** was prepared according to the general procedure E. Reaction time: 10 h. Isolated yield: 69% (write solid); d.r.: 20: 1 (*syn*-HH: *anti*-HH); total yield together with the *anti*-head-to-head isomer: 72%. ^{1}H NMR (400 MHz, $CDCl_3$) δ 7.86 - 7.79 (m, 2H), 7.61 - 7.49 (m, 1H), 7.45 - 7.39 (m, 2H), 7.25 - 7.12 (m, 5H), 7.07 - 6.98 (m, 2H), 6.77 (d, *J* = 8.2 Hz, 2H), 4.73 (dd, *J* = 9.4, 4.6 Hz, 1H), 4.47 (t, *J* = 9.8 Hz, 1H), 4.03 (dd, *J* = 10.0, 4.6 Hz, 1H), 3.88 (t, *J* = 9.6 Hz, 1H), 2.15 (s, 3H). ^{13}C NMR (101 MHz, $CDCl_3$) δ 207.39, 198.43, 138.20, 137.99, 135.47, 133.63, 131.24, 129.51, 128.94, 128.66, 128.52, 128.25, 127.12, 120.46,

50.57, 48.78, 45.54, 43.72, 28.47. HRMS (ESI) Calcd. for $C_{25}H_{21}BrO_2$ $[M+Na]^+$: 455.0617. Found: 455.0611.

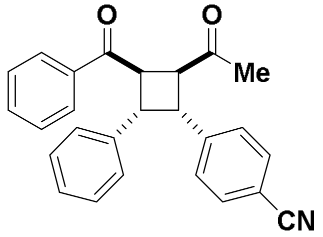

The compound **69** was prepared according to the general procedure E. Reaction time: 10 h. Isolated yield: 67% (write solid); d.r.: 20: 1 (*syn*-HH: *anti*-HH); total yield together with the *anti*-head-to-head isomer: 70%. ^{1}H NMR (400 MHz, $CDCl_3$) δ 7.84 (d, J = 7.8 Hz, 2H), 7.61 - 7.54 (m, 1H), 7.48 - 7.41 (m, 2H), 7.41 - 7.34 (m, 2H), 7.24 - 7.13 (m, 3H), 7.02 (d, J = 6.8 Hz, 2H), 6.97 (d, J = 8.0 Hz, 2H), 4.76 (dd, J = 9.4, 4.2 Hz, 1H), 4.57 (t, J = 10.0 Hz, 1H), 4.03 (dd, J = 10.2, 4.3 Hz, 1H), 3.92 (t, J = 9.6 Hz, 1H), 2.17 (s, 3H). ^{13}C NMR (101 MHz, $CDCl_3$) δ 206.80, 197.99, 144.70, 137.87, 135.15, 133.84, 131.91, 129.03, 128.84, 128.59, 128.46, 128.20, 127.45, 118.94, 110.29, 49.96, 49.18, 45.92, 43.76, 28.37. HRMS (ESI) Calcd. for $C_{26}H_{21}NO_2[M+Na]^+$: 402.1465. Found: 402.1457.

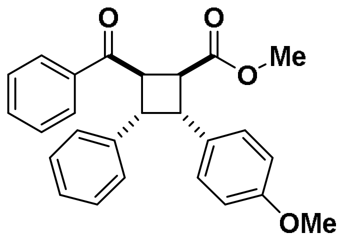

The compound **70** was prepared according to the general procedure D. Reaction time: 10 h. Isolated yield: 70% (write solid); d.r.: 17: 1 (*syn*-HH: *anti*-HH); total yield together with the *anti*-head-to-head isomer: 74%. ^{1}H NMR (400 MHz, $CDCl_3$) δ 7.92 - 7.81 (m, 2H), 7.59 - 7.52 (m, 1H), 7.47 - 7.40 (m, 2H), 7.21 - 7.08 (m, 3H), 7.03 (d, J = 7.4 Hz, 2H), 6.83 (d, J = 8.6 Hz, 2H), 6.65 (d, J = 8.6 Hz, 2H), 4.64 (dd, J = 9.8, 5.2 Hz, 1H), 4.45 (t, J = 9.6 Hz, 1H), 4.22 (dd, J = 10.2, 5.2 Hz, 1H), 3.96 (t, J = 9.6 Hz, 1H), 3.70 (s, 3H), 3.58 (s, 3H). ^{13}C NMR (101 MHz, $CDCl_3$) δ 198.11, 173.04, 158.11, 138.89, 135.71, 133.42, 130.96, 128.80, 128.48, 128.40, 128.31, 126.69, 113.52, 55.25, 51.93, 47.29, 45.23, 43.80, 43.66. HRMS (ESI) Calcd. for $C_{26}H_{24}O_4$ $[M+Na]^+$: 423.15668. Found: 423.15555.

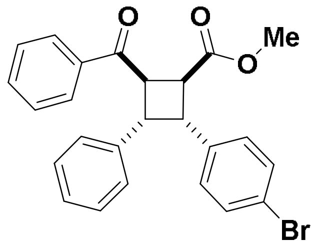

The compound **71** was prepared according to the general procedure D. Reaction time: 10 h. Isolated yield: 73% (write solid); d.r.: 15: 1 (*syn*-HH: *anti*-HH); total yield together with the *anti*-head-to-head isomer: 78%. ^{1}H NMR (400 MHz, $CDCl_3$) δ 7.90 - 7.84 (m, 2H), 7.59 - 7.53 (m, 1H), 7.47 - 7.40 (m, 2H), 7.25 - 7.11 (m, 5H), 7.06 - 7.00 (m, 2H), 6.78 (d, *J* = 8.4 Hz, 2H), 4.63 (dd, *J* = 9.8, 4.8 Hz, 1H), 4.49 (t, *J* = 9.8 Hz, 1H), 4.20 (dd, *J* = 10.2, 4.8 Hz, 1H), 3.97 (t, *J* = 9.6 Hz, 1H), 3.60 (s, 3H). ^{13}C NMR (101 MHz, $CDCl_3$) δ 197.90, 172.77, 138.45, 137.95, 135.57, 133.56, 131.18, 129.42, 128.86, 128.64, 128.51, 128.29, 127.06, 120.35, 52.01, 47.41, 45.22, 43.66, 43.21. HRMS (ESI) Calcd. for $C_{25}H_{21}BrO_3$ $[M+Na]^+$: 471.0566. Found: 471.0559.

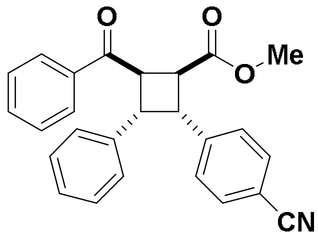

The compound **72** was prepared according to the general procedure D. Reaction time: 10 h. Isolated yield: 71% (write solid); d.r.: 5: 1 (*syn*-HH: *anti*-HH); total yield together with the *anti*-head-to-head isomer: 85%. ^{1}H NMR (400 MHz, $CDCl_3$) δ 7.87 (d, *J* = 8.0 Hz, 2H), 7.61 - 7.53 (m, 1H), 7.48 - 7.41 (m, 2H), 7.39 (d, *J* = 8.0 Hz, 2H)., 7.23 - 7.11 (m, 3H), 7.05 - 6.95 (m, 4H), 4.72 - 4.54 (m, 2H), 4.19 (dd, *J* = 10.2, 4.4 Hz, 1H), 4.03 (t, *J* = 9.6 Hz, 1H), 3.62 (s, 3H). ^{13}C NMR (101 MHz, $CDCl_3$) δ 197.70, 172.52, 144.55, 138.00, 135.40, 133.70, 131.88, 128.91, 128.78, 128.52, 128.35, 128.19, 127.34, 118.98, 110.18, 52.10, 47.40, 45.53, 44.05, 42.60. HRMS (ESI) Calcd. for $C_{26}H_{21}NO_3$ $[M+Na]^+$: 418.14136. Found: 418.14050.

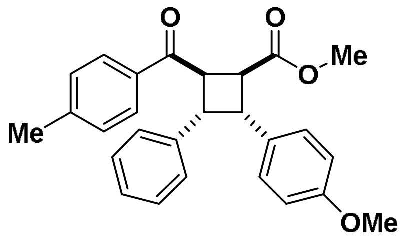

The compound **73** was prepared according to the general procedure D. Reaction time: 10 h. Isolated yield:

50% (write solid); d.r.: 9: 1 (*syn*-HH: *anti*-HH); total yield together with the *anti*-head-to-head isomer: 55%. ^{1}H NMR (400 MHz, $CDCl_3$) δ 7.78 (d, *J* = 8.2 Hz, 2H), 7.25 - 7.08 (m, 5H), 7.06 - 7.00 (m, 2H), 6.83 (d, *J* = 8.4 Hz, 2H), 6.68 - 6.62 (m, 2H), 4.62 (dd, J = 9.8, 5.2 Hz, 1H), 4.45 (t, J = 9.6 Hz, 1H), 4.21 (dd, J = 10.0, 5.2 Hz, 1H), 3.94 (t, J = 9.4 Hz, 1H), 3.70 (s, 3H), 3.59 (s, 3H), 2.39 (s, 3H). ^{13}C NMR (101 MHz, $CDCl_3$) δ 197.70, 173.09, 158.10, 144.25, 139.03, 133.24, 131.08, 129.49, 128.81, 128.61, 128.38, 128.33, 126.64, 113.53, 55.25, 51.90, 47.26, 45.31, 43.79, 43.64, 21.81. HRMS (ESI) Calcd. for $C_{27}H_{26}O_4$ $[M+Na]^+$: 437.1723. Found: 437.1718.

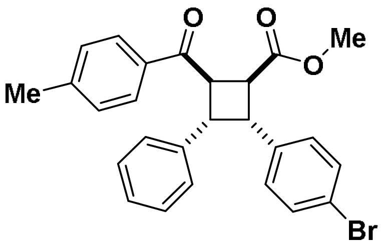

The compound **74** was prepared according to the general procedure D. Reaction time: 10 h. Isolated yield: 49% (write solid); d.r.: 9: 1 (*syn*-HH: *anti*-HH); total yield together with the *anti*-head-to-head isomer: 54%. ^{1}H NMR (400 MHz, $CDCl_3$) δ 7.77 (d, *J* = 8.2 Hz, 2H), 7.25 - 7.08 (m, 7H), 7.03 (d, *J* = 7.2 Hz, 2H), 6.78 (d, *J* = 8.2 Hz, 2H), 4.61 (dd, *J* = 9.8, 4.6 Hz, 1H), 4.48 (t, *J* = 9.8 Hz, 1H), 4.16 (dd, *J* = 10.0, 4.6 Hz, 1H), 3.95 (t, *J* = 9.6 Hz, 1H), 3.61 (s, 3H), 2.39 (s, 3H). ^{13}C NMR (101 MHz, $CDCl_3$) δ 197.49, 172.83, 144.45, 138.56, 138.03, 133.06, 131.16, 129.54, 129.42, 128.64, 128.61, 128.30, 127.01, 120.30, 52.00, 47.38, 45.31, 43.63, 43.15, 21.84. HRMS (ESI) Calcd. for $C_{26}H_{23}BrO_3$ $[M+Na]^+$: 485.0723. Found: 485.0715.

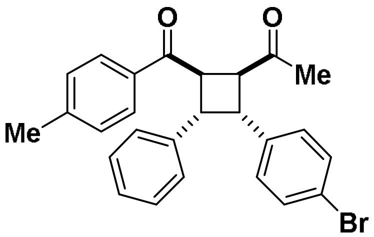

The compound **75** was prepared according to the general procedure E. Reaction time: 10 h. Isolated yield: 55% (write solid); d.r.: 8: 1 (*syn*-HH: *anti*-HH); total yield together with the *anti*-head-to-head isomer: 62%. ^{1}H NMR (400 MHz, $CDCl_3$) δ 7.74 (d, *J* = 8.2 Hz, 2H), 7.25 - 7.08 (m, 7H), 7.03 (d, *J* = 7.0 Hz, 2H), 6.76 (d, *J* = 8.2 Hz, 2H), 4.72 (dd, *J* = 9.4, 4.5 Hz, 1H), 4.45 (t, *J* = 9.8 Hz, 1H), 4.00 (dd, *J* = 10.0, 4.5 Hz, 1H), 3.86 (t, *J* = 9.6 Hz, 1H), 2.39 (s, 3H), 2.15 (s, 3H). ^{13}C NMR (101 MHz, $CDCl_3$) δ 207.46, 197.97, 144.57, 138.34,

138.10, 132.91, 131.21, 129.63, 129.51, 128.66, 128.64, 128.28, 127.08, 120.40, 50.45, 48.90, 45.66, 43.61, 28.49, 21.83. HRMS (ESI) Calcd. for $C_{26}H_{23}BrO_2$ $[M+Na]^+$: 469.0774. Found: 469.0767.

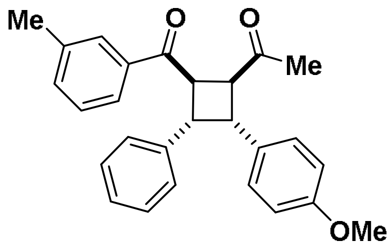

The compound **76** was prepared according to the general procedure E. Reaction time: 10 h. Isolated yield: 54% (write solid); d.r.: 14: 1 (*syn*-HH: *anti*-HH); total yield together with the *anti*-head-to-head isomer: 58%. ^1H NMR (400 MHz, $CDCl_3$) δ 7.69 - 7.63 (m, 1H), 7.55 (d, *J* = 7.6 Hz, 1H), 7.32 - 7.28 (m, 1H), 7.25 - 7.19(m, 1H), 7.17 - 7.03 (m, 3H), 6.94 (d, *J* = 7.0 Hz, 2H), 6.76 (d, *J* = 8.6 Hz, 2H), 6.65 - 6.56 (m, 2H), 4.65 (dd, *J* = 9.4, 5.2 Hz, 1H), 4.33 (t, *J* = 9.6 Hz, 1H), 4.02 (dd, *J* = 10.0, 5.0 Hz, 1H), 3.82 (t, *J* = 9.4 Hz, 1H), 3.66 (s, 3H), 2.30 (s, 3H), 2.09 (s, 3H). ^{13}C NMR (101 MHz, $CDCl_3$) δ 208.01, 199.14, 158.25, 138.77, 138.71, 135.84, 134.21, 131.08, 129.03, 128.95, 128.69, 128.39, 128.31, 126.71, 125.66, 113.62, 55.27, 51.34, 48.54, 45.55, 43.98, 28.66, 21.46. HRMS (ESI) Calcd. for $C_{27}H_{26}O_3$ $[M+Na]^+$: 421.1774. Found: 421.1766.

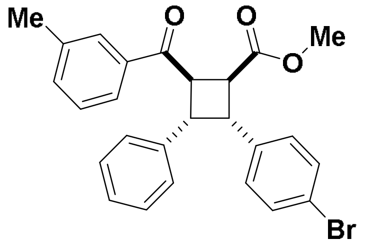

The compound **77** was prepared according to the general procedure D. Reaction time: 10 h. Isolated yield: 69% (write solid); d.r.: 12: 1 (*syn*-HH: *anti*-HH); total yield together with the *anti*-head-to-head isomer: 75%. ^1H NMR (400 MHz, $CDCl_3$) δ 7.72 - 7.66 (m, 1H), 7.63 (d, *J* = 7.6 Hz, 1H), 7.41 - 7.29 (m, 2H), 7.24 - 7.11 (m, 5H), 7.02 (d, *J* = 7.0 Hz, 2H), 6.78 (d, *J* = 8.4 Hz, 2H), 4.60 (dd, *J* = 9.8, 4.8 Hz, 1H), 4.48 (t, *J* = 9.8 Hz, 1H), 4.19 (dd, *J* = 10.2, 4.9 Hz, 1H), 3.96 (t, *J* = 9.6 Hz, 1H), 3.60 (s, 3H), 2.36 (s, 3H). ^{13}C NMR (101 MHz, $CDCl_3$) δ 198.12, 172.81, 138.69, 138.59, 138.05, 135.63, 134.31, 131.18, 129.44, 129.06, 128.69, 128.60, 128.33, 127.02, 125.73, 120.32, 52.00, 47.56, 45.28, 43.63, 43.28, 21.47. HRMS (ESI) Calcd. for $C_{26}H_{23}BrO_3$ $[M+Na]^+$: 485.0723. Found: 485.0714.

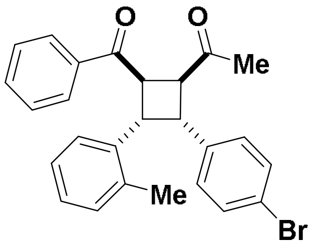

The compound **78** was prepared according to the general procedure E. Reaction time: 10 h. Isolated yield: 40% (write solid); no *anti*-head-to-head isomer was observed. ^{1}H NMR (400 MHz, $CDCl_3$) δ 7.88 (d, J = 7.8 Hz, 2H), 7.60 - 7.53 (m, 1H), 7.48 - 7.36 (m, 3H), 7.26 - 7.15 (m, 3H), 7.14 - 7.07 (m, 1H), 6.97 (d, J = 7.4 Hz, 1H), 6.77 (d, J = 8.4 Hz, 2H), 4.87 (dd, J = 9.4, 5.2 Hz, 1H), 4.39 (t, J = 9.4 Hz, 1H), 4.26 (dd, J = 9.8, 5.2 Hz, 1H), 3.76 (t, J = 9.0 Hz, 1H), 2.08 (s, 3H), 1.87 (s, 3H). ^{13}C NMR (101 MHz, $CDCl_3$) δ 207.22, 198.95, 138.13, 137.49, 135.76, 133.56, 131.15, 130.76, 129.85, 128.96, 128.36, 127.21, 126.22, 125.62, 120.89, 52.15, 46.71, 44.29, 42.23, 28.54, 19.89. HRMS (ESI) Calcd. for $C_{26}H_{23}BrO_2$ $[M+Na]^+$: 469.0774. Found: 469.0767.

Supplementary Note 2. ^{1}H and ^{13}C and ^{19}F NMR spectra of products

NMR spectra of product 1

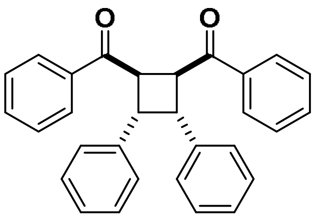

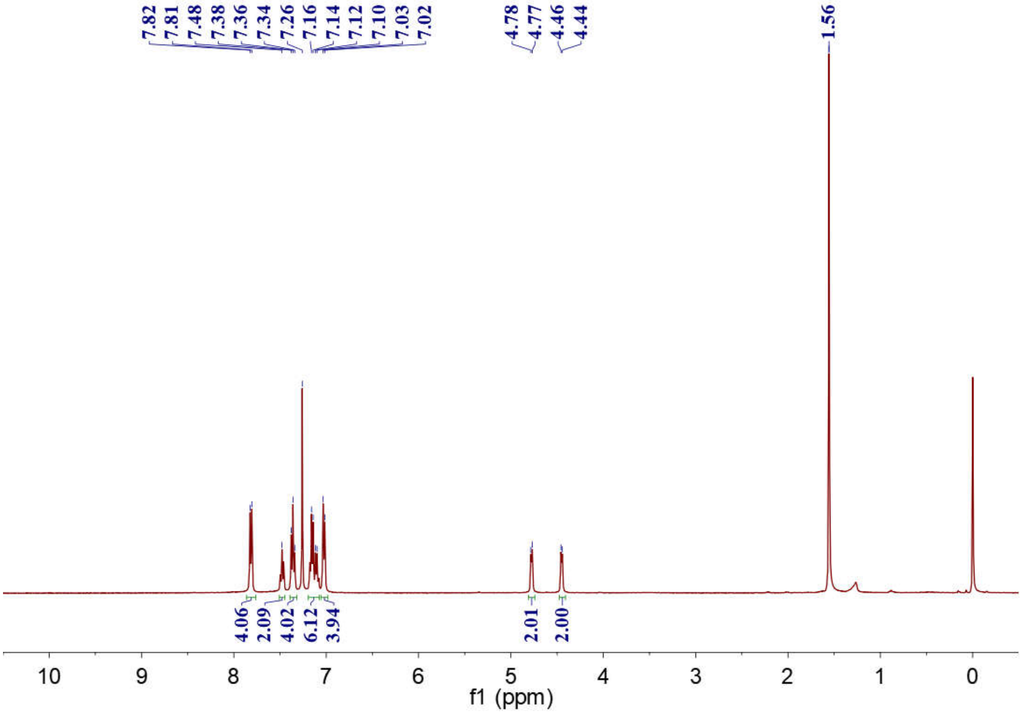

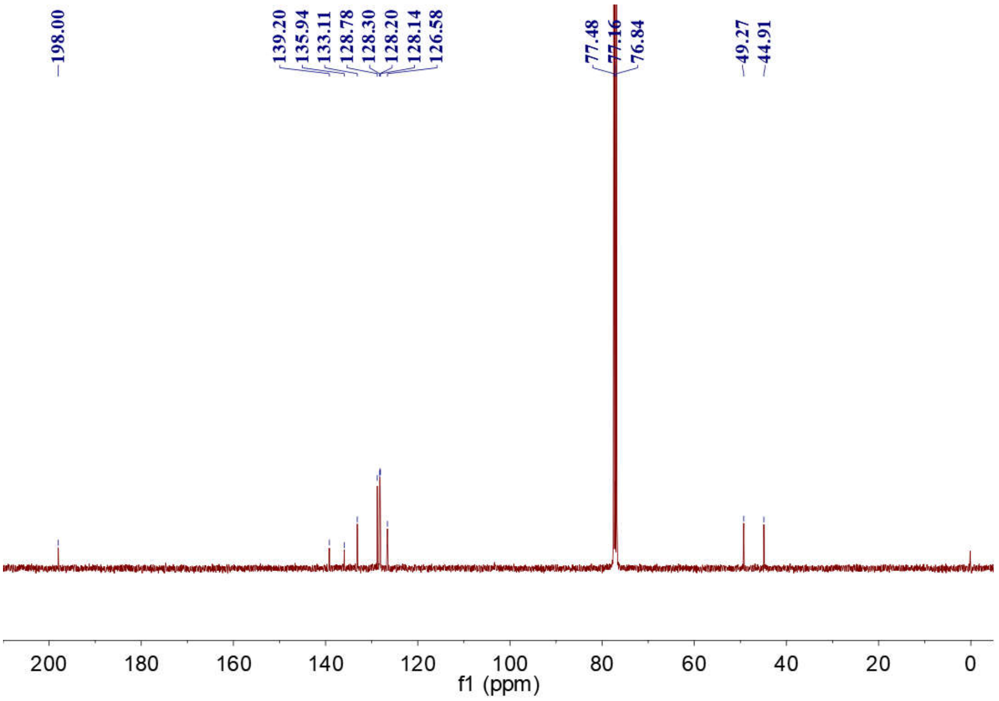

NMR spectra of product 2

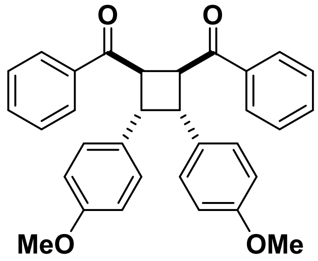

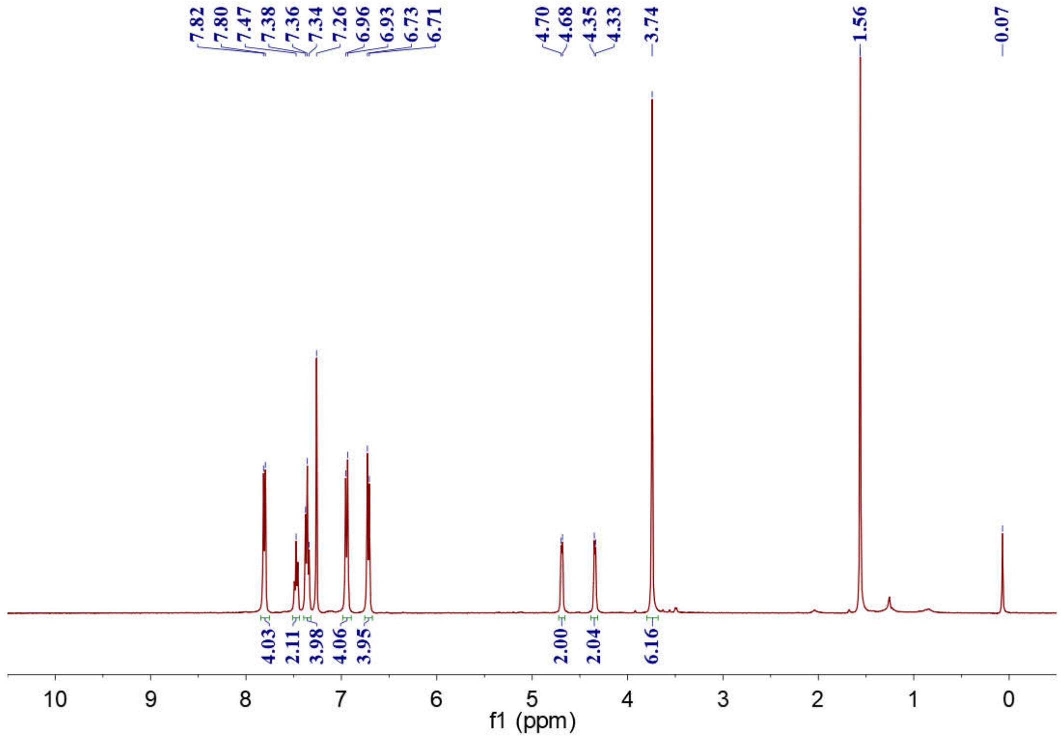

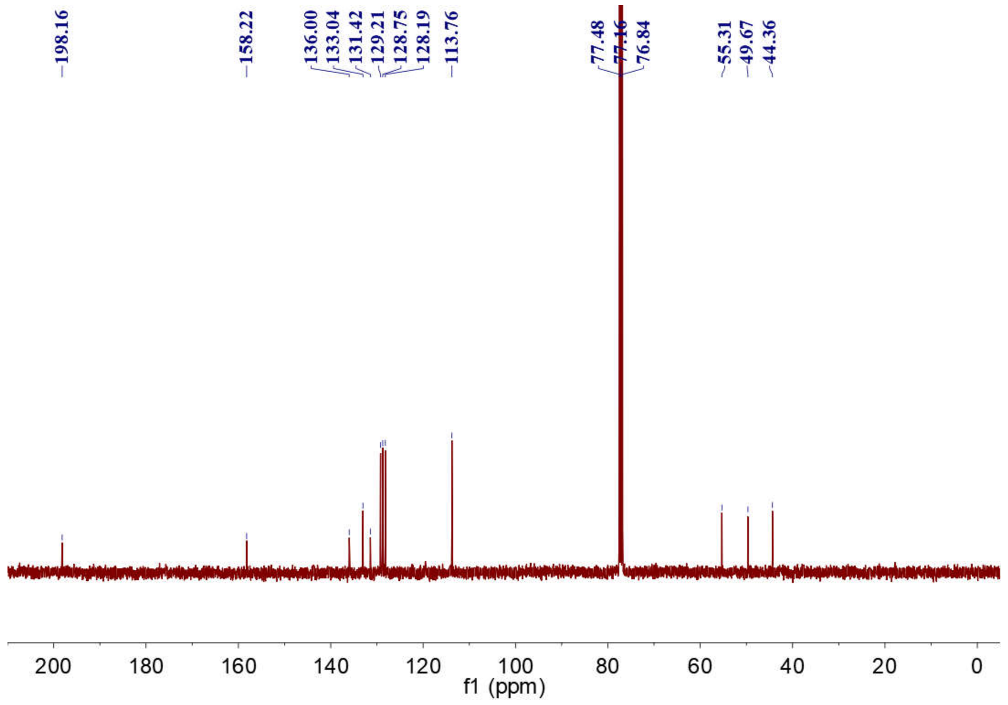

NMR spectra of product 3

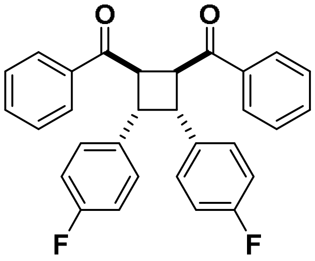

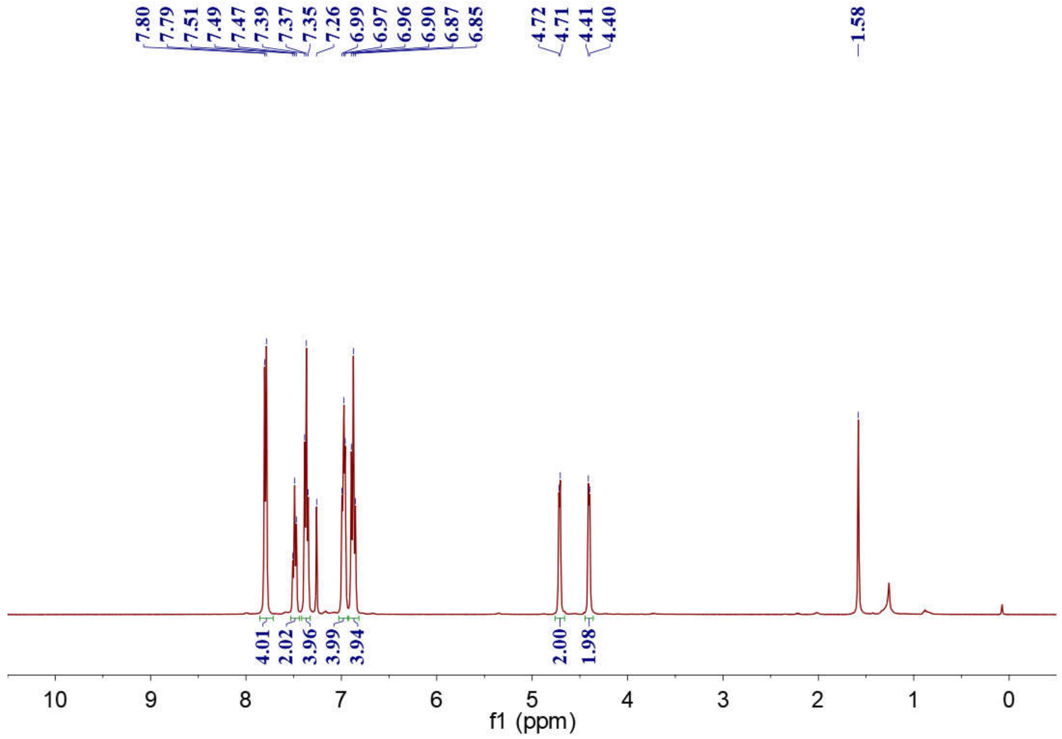

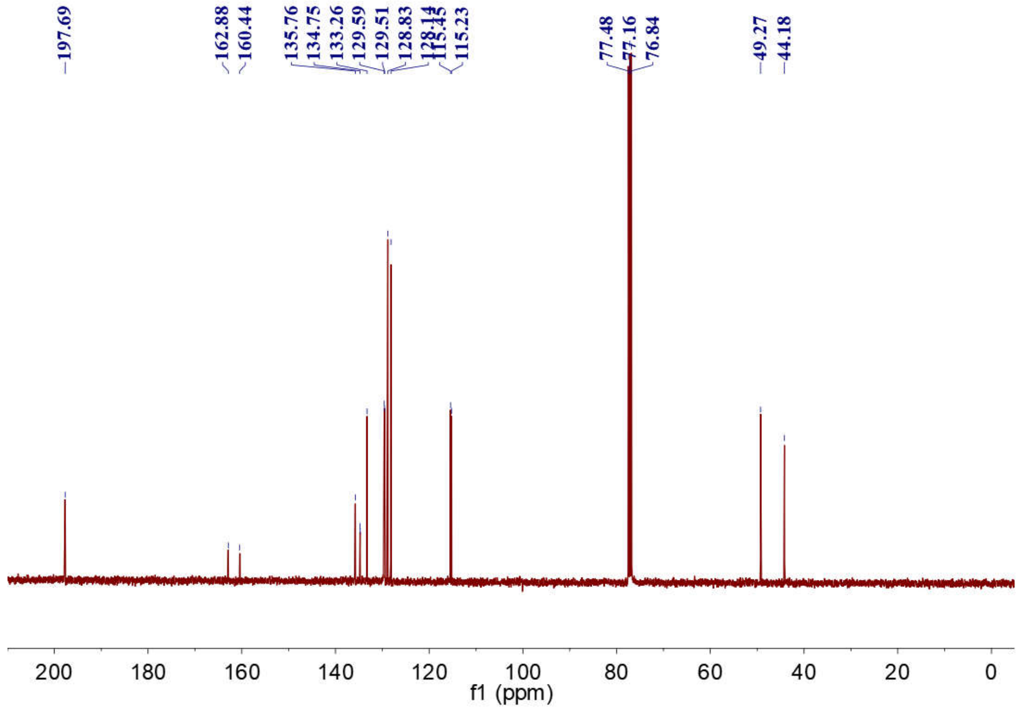

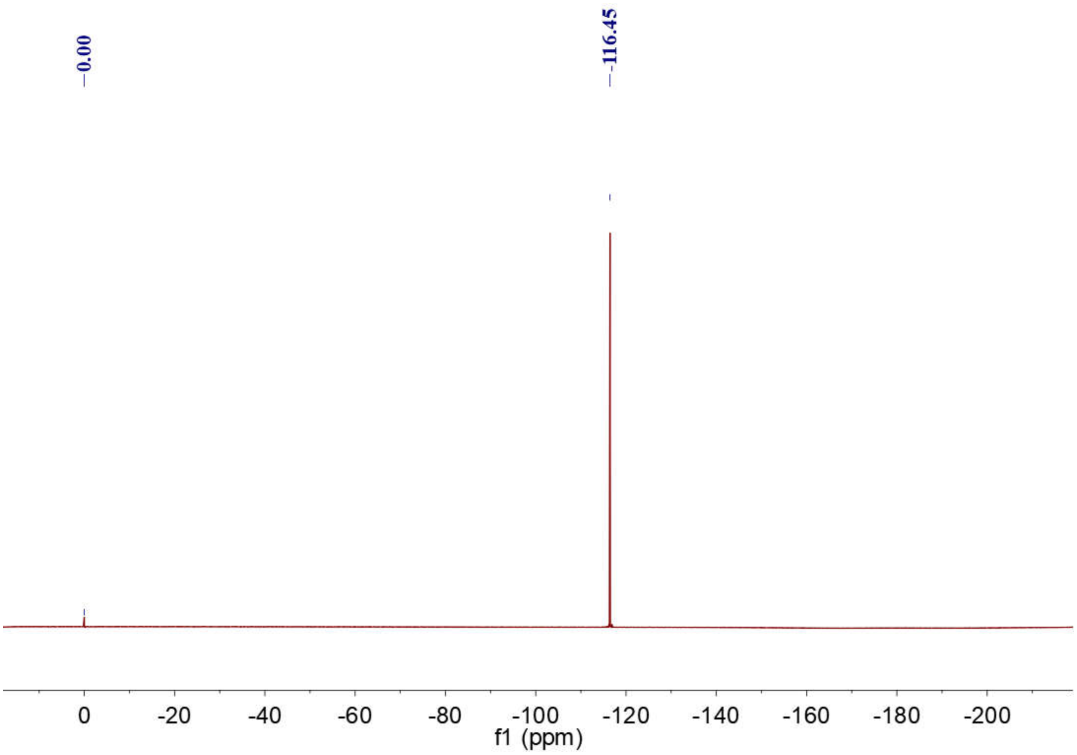

-0.00
-116.45
0
-20
-40
-60
-80
-100
-120
-140
-160
-180
-200
f1 (ppm)

NMR spectra of product 4

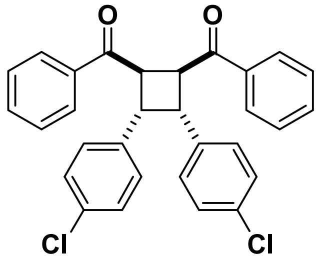

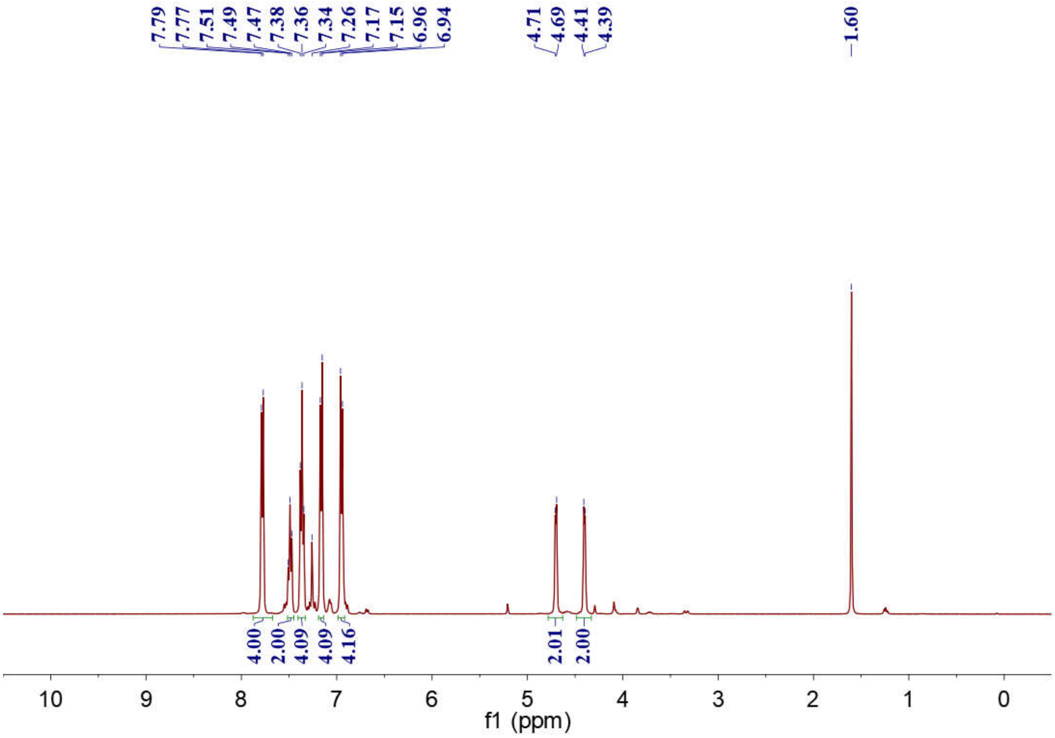

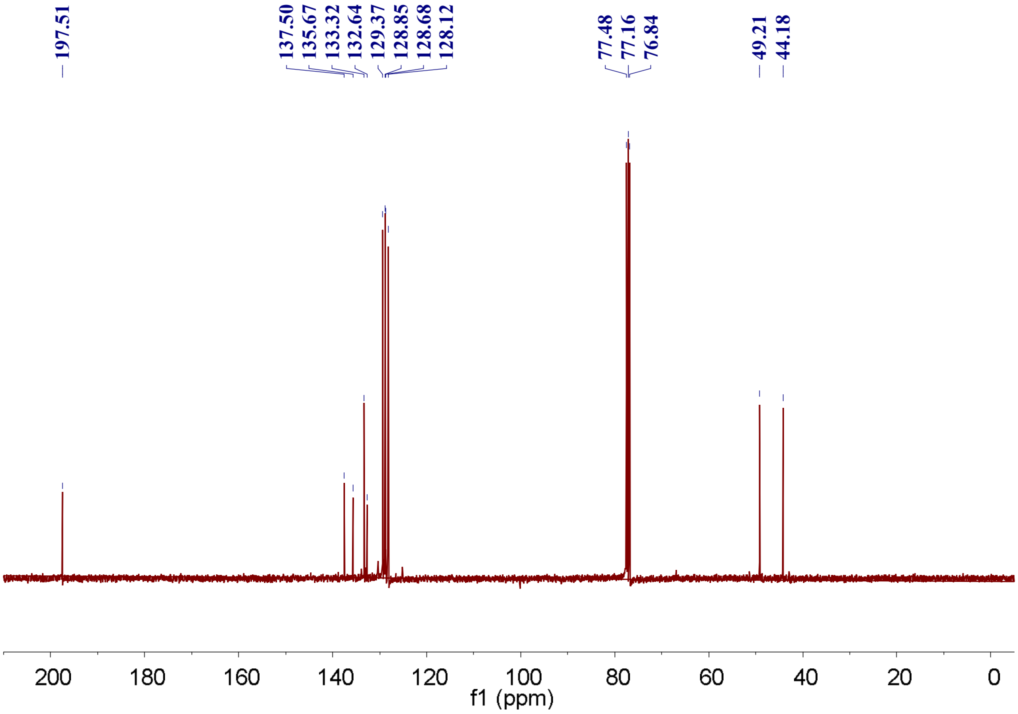

NMR spectra of product 5

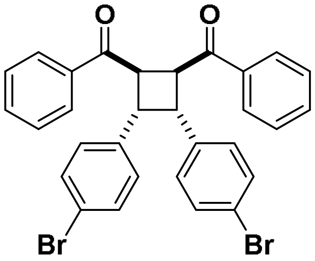

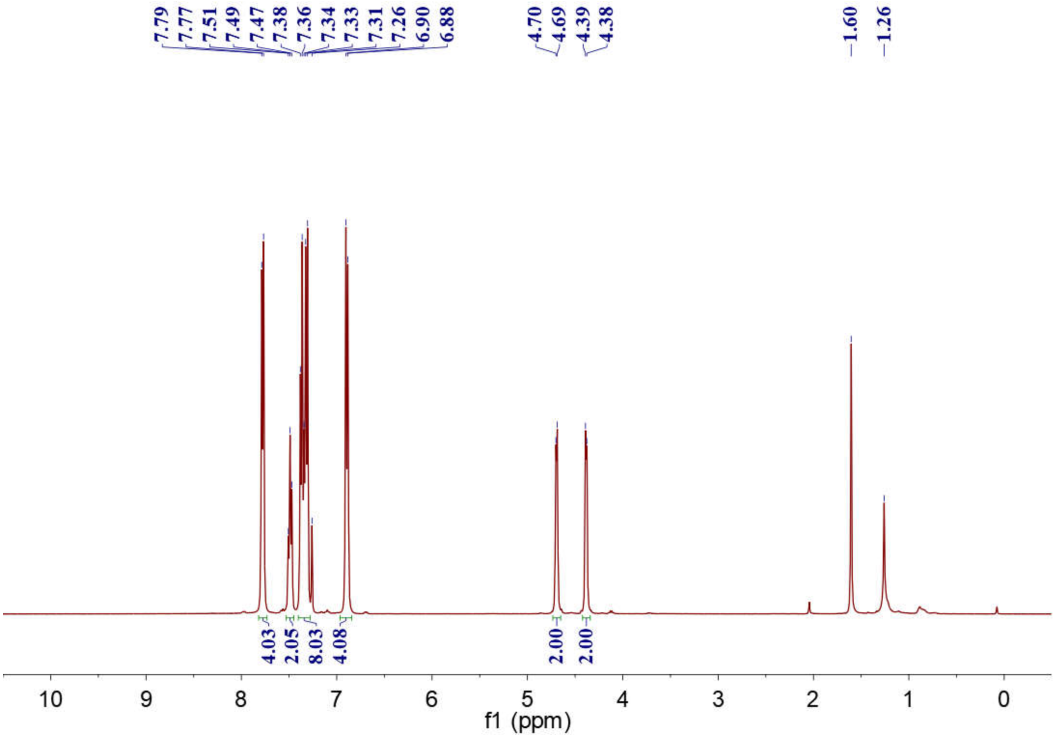

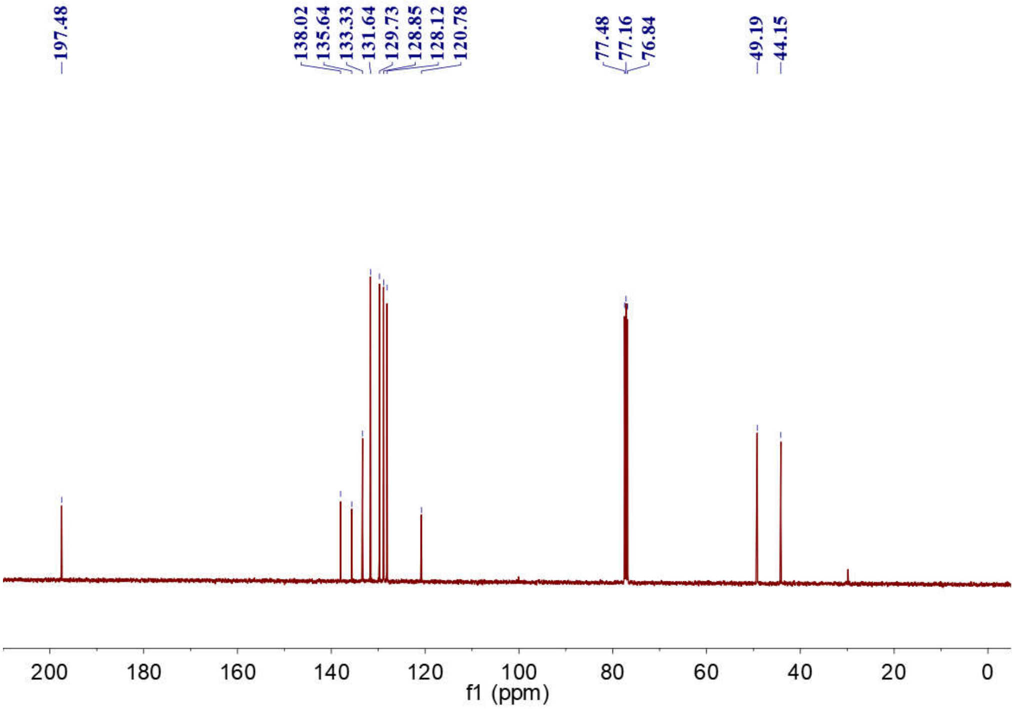

NMR spectra of product 6

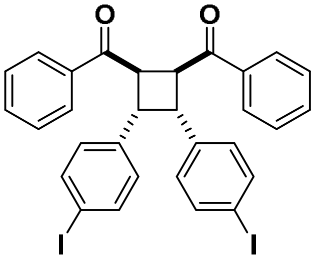

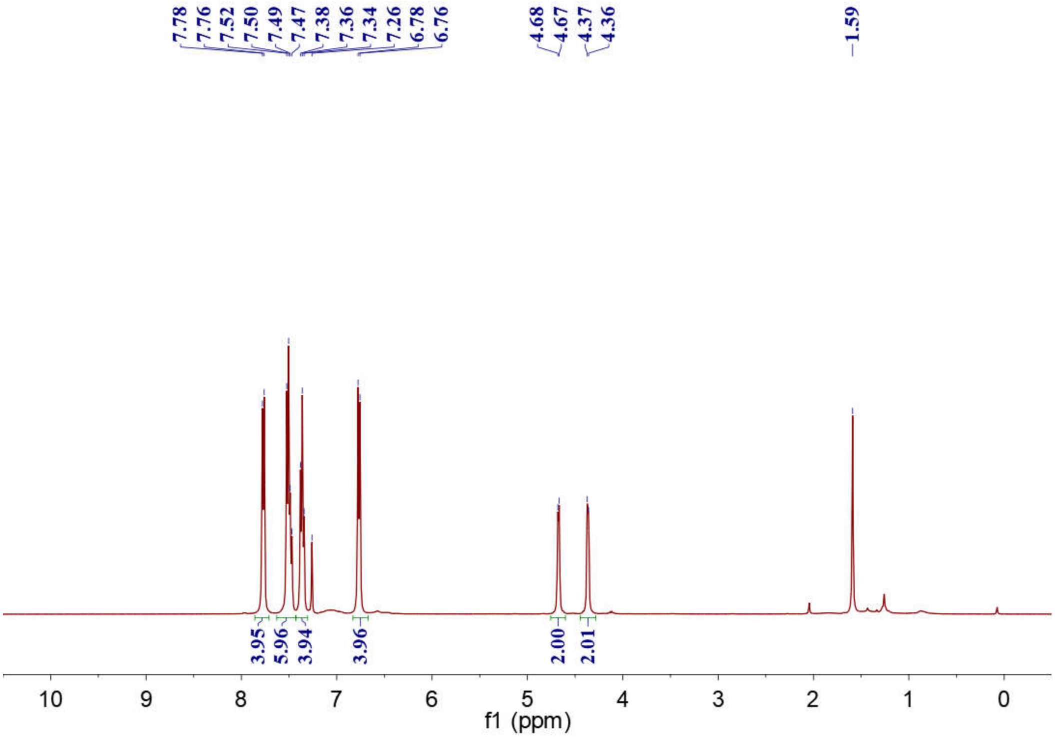

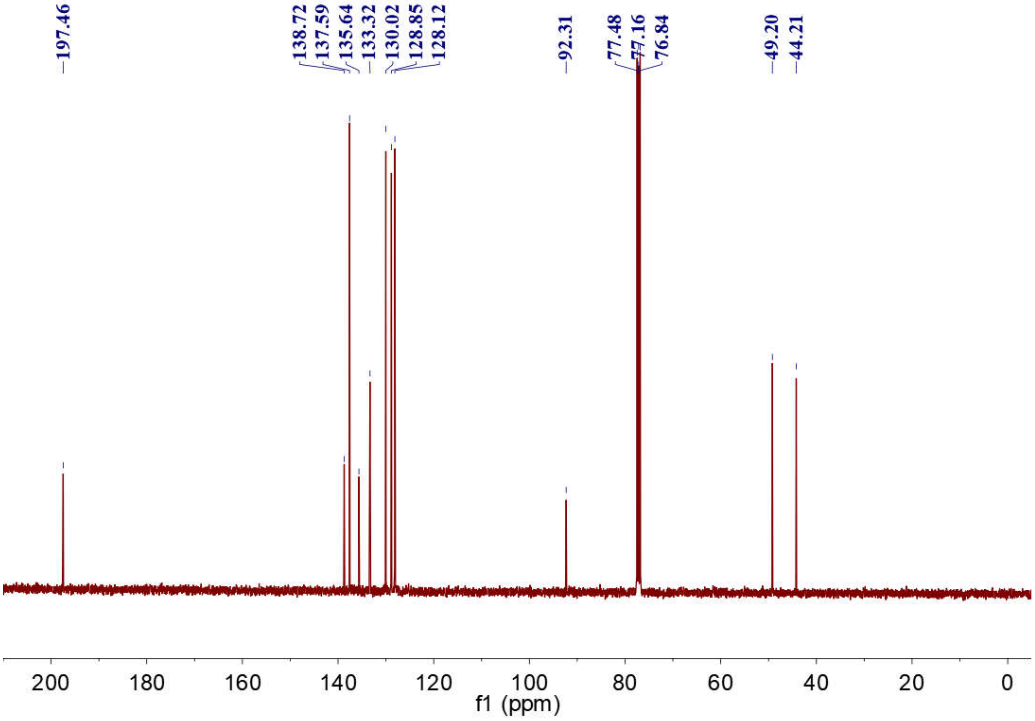

NMR spectra of product 7

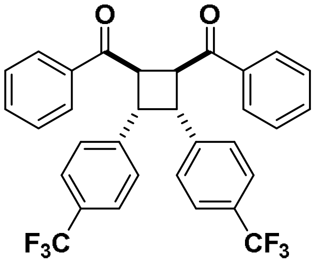

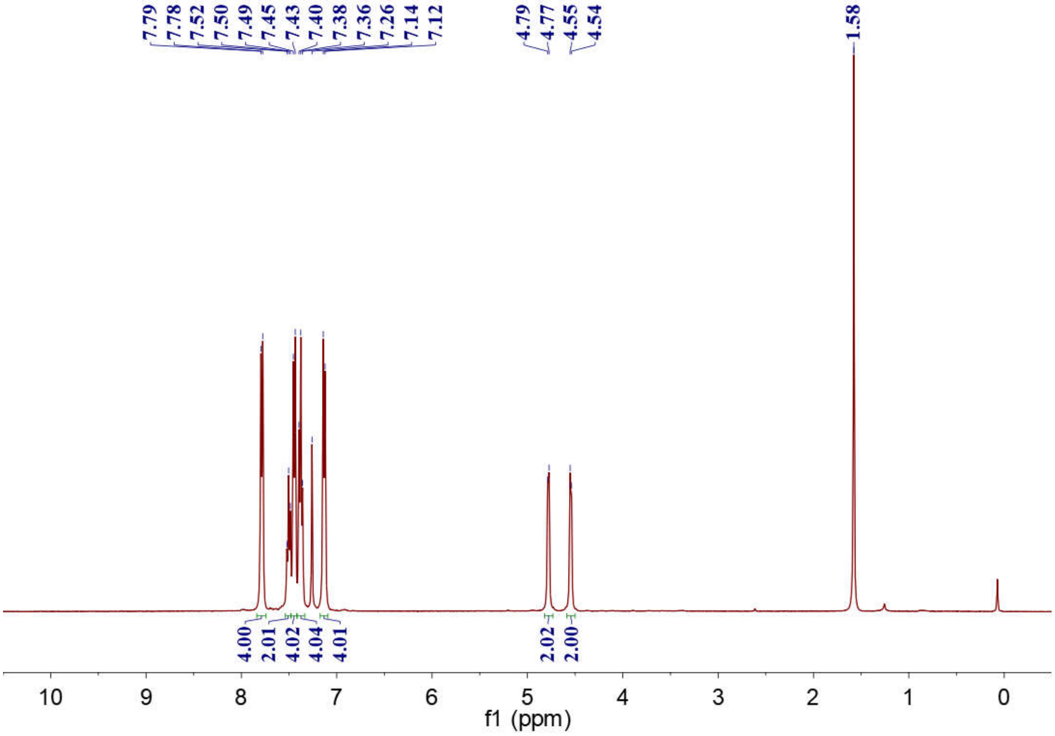

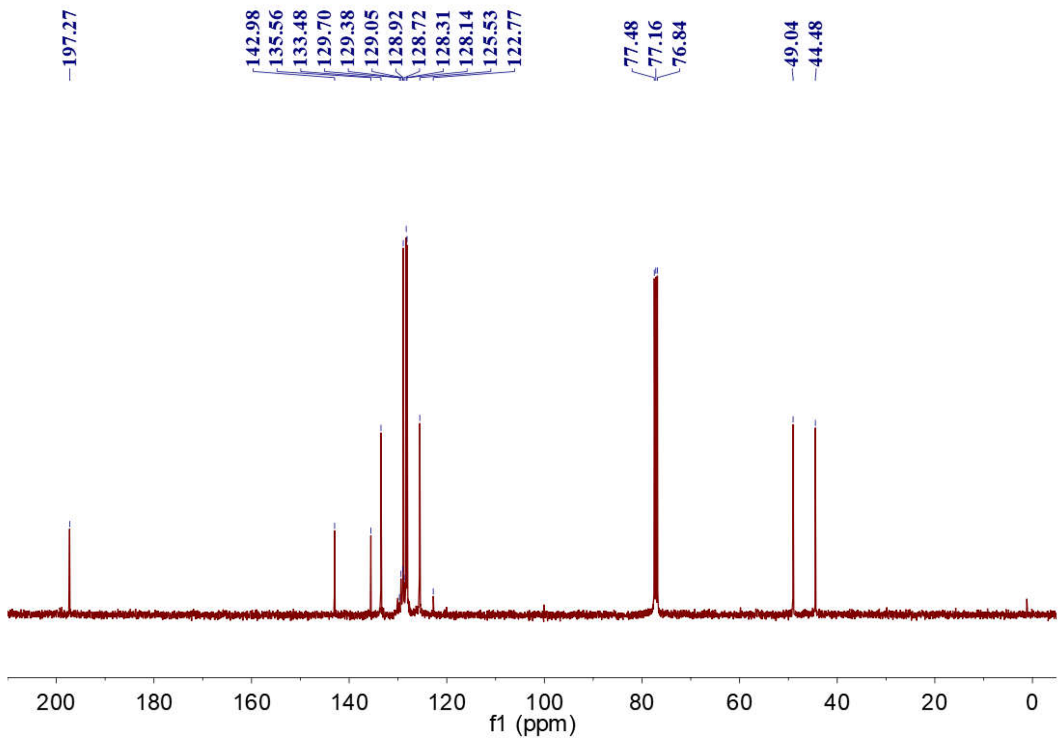
197.27
142.98
135.56
133.48
129.70
129.38
129.05
128.92
128.72
128.31
128.14
125.53
122.77
77.48
77.16
76.84
49.04
44.48
200 180 160 140 120 100 80 60 40 20 0
f1 (ppm)

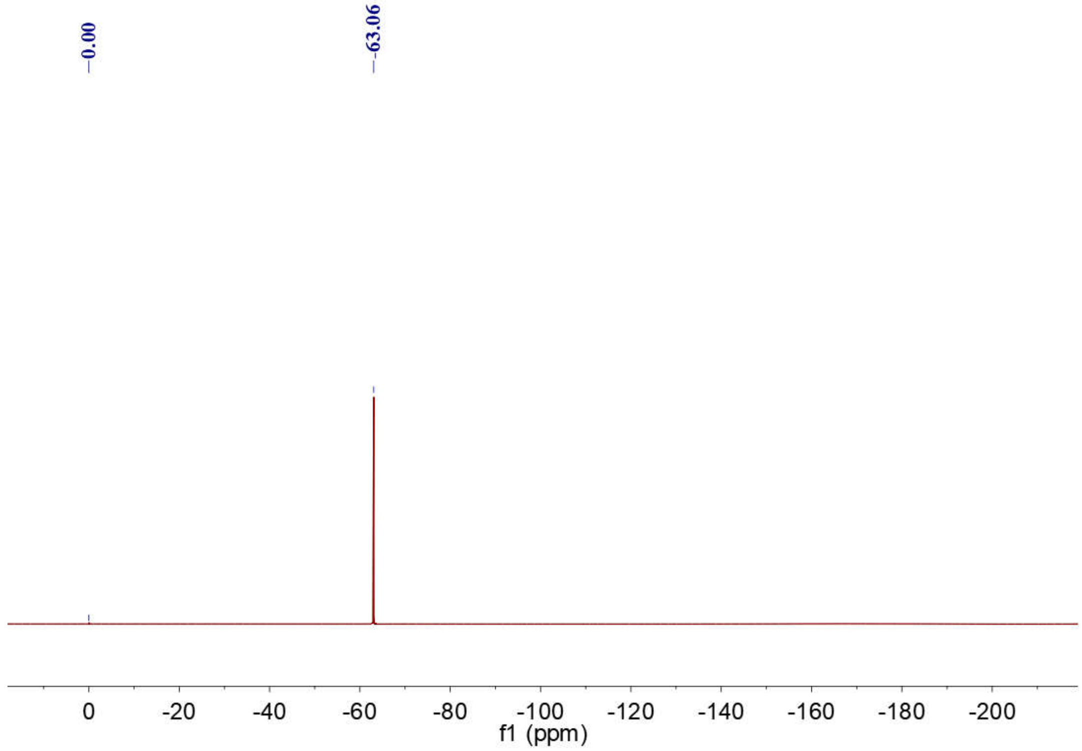
0.00
-63.06
0 -20 -40 -60 -80 -100 -120 -140 -160 -180 -200
f1 (ppm)

NMR spectra of product 8

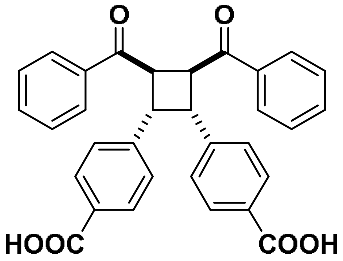

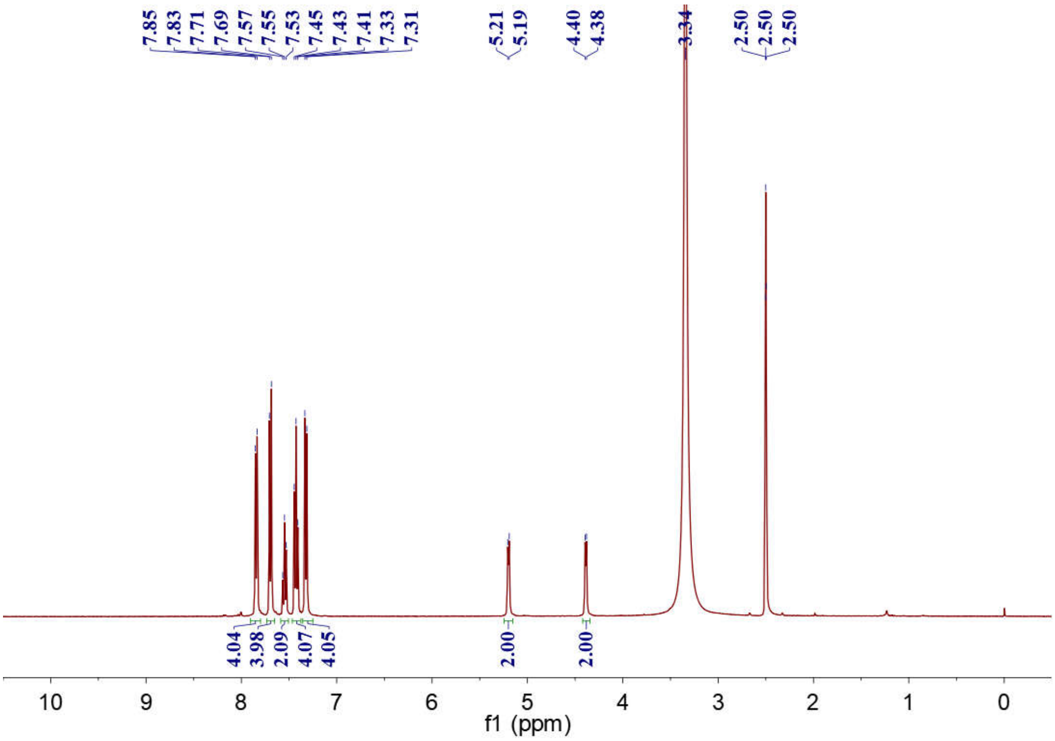

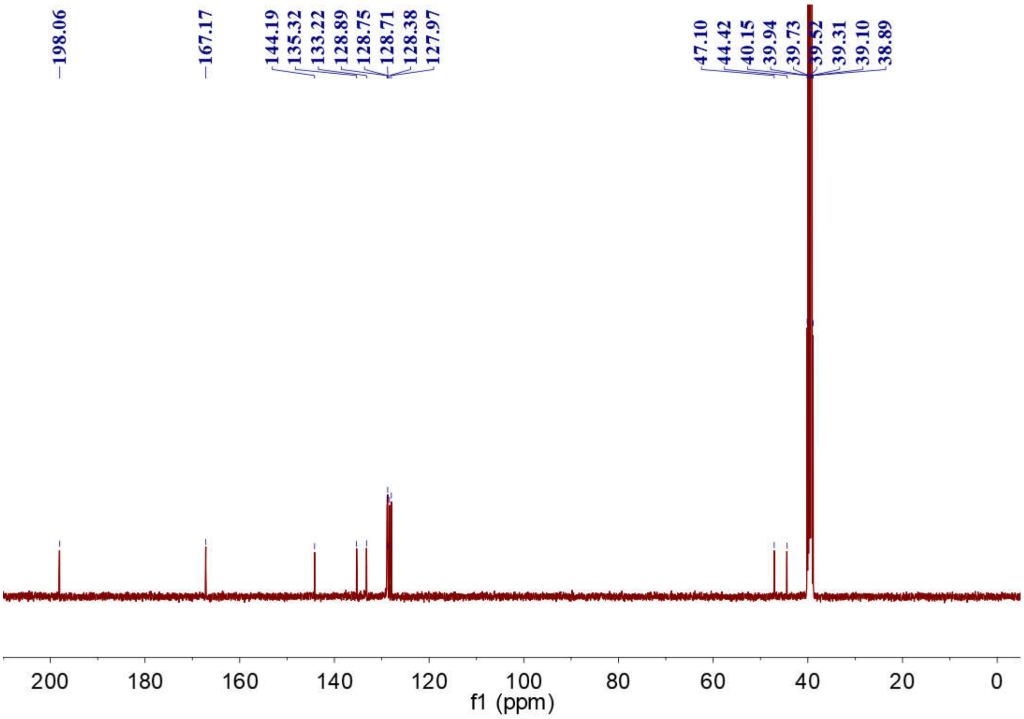

NMR spectra of product 9

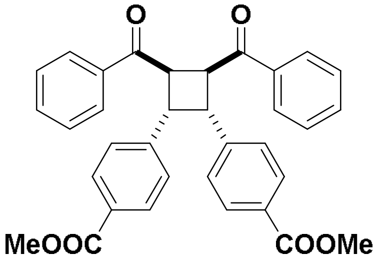

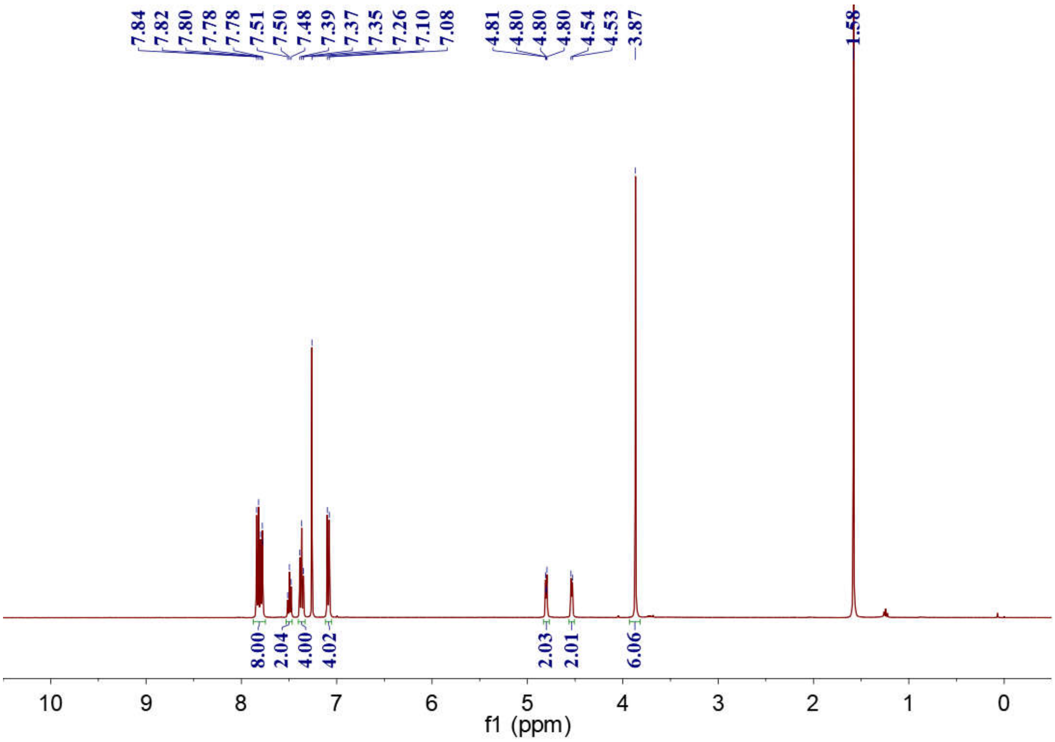

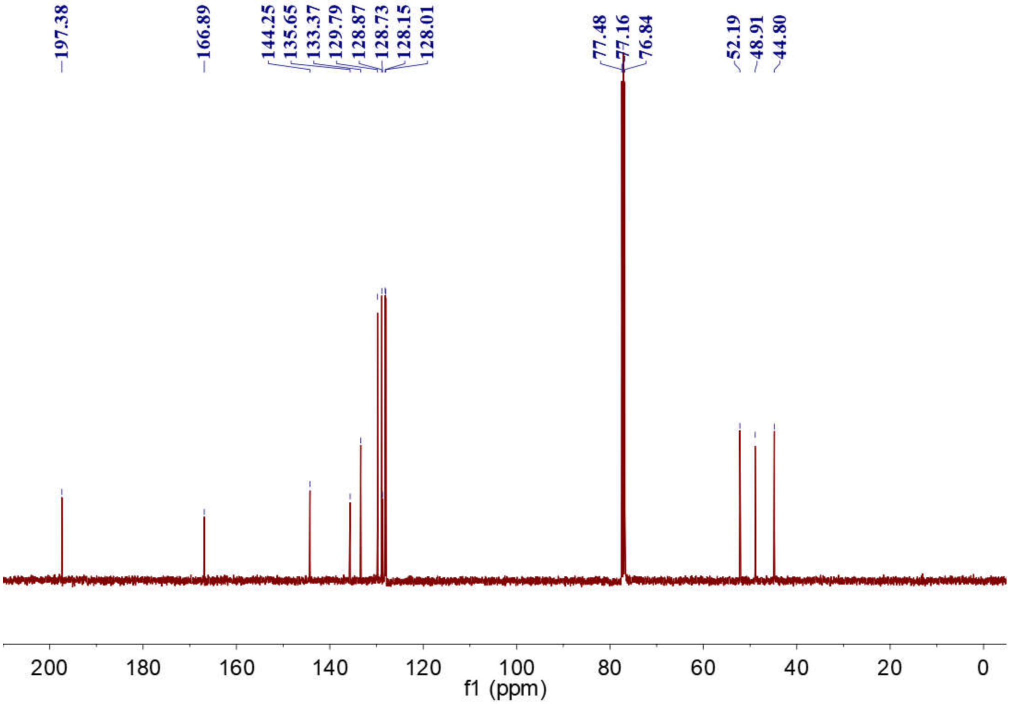

NMR spectra of product 10

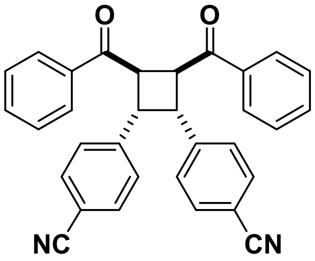

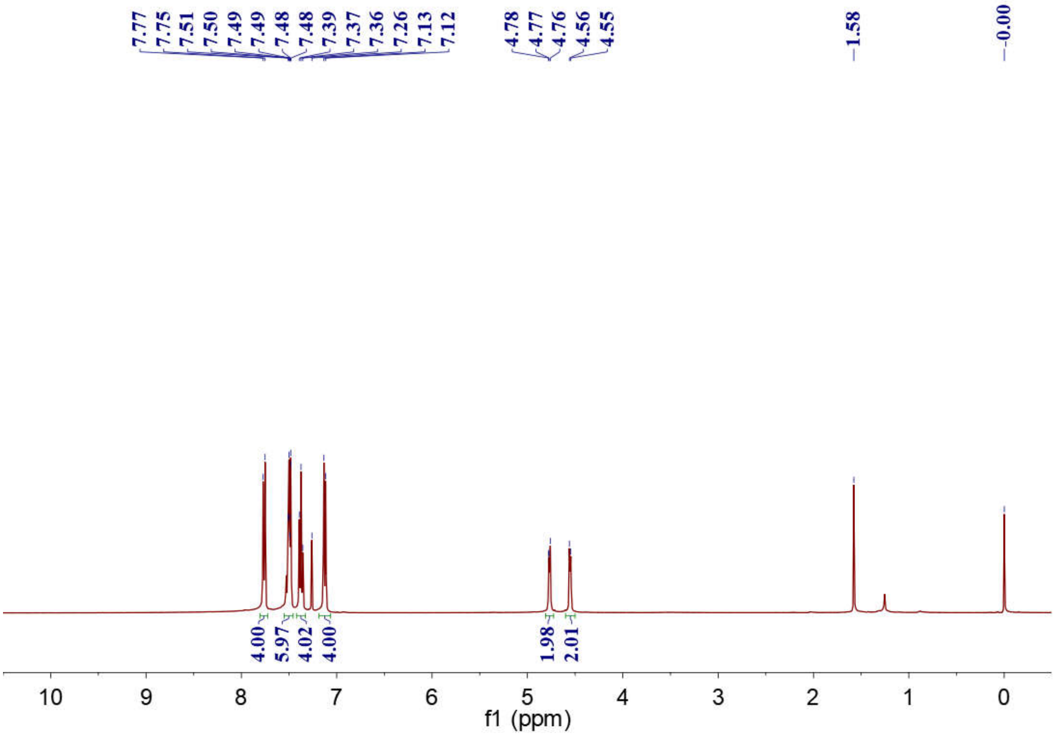

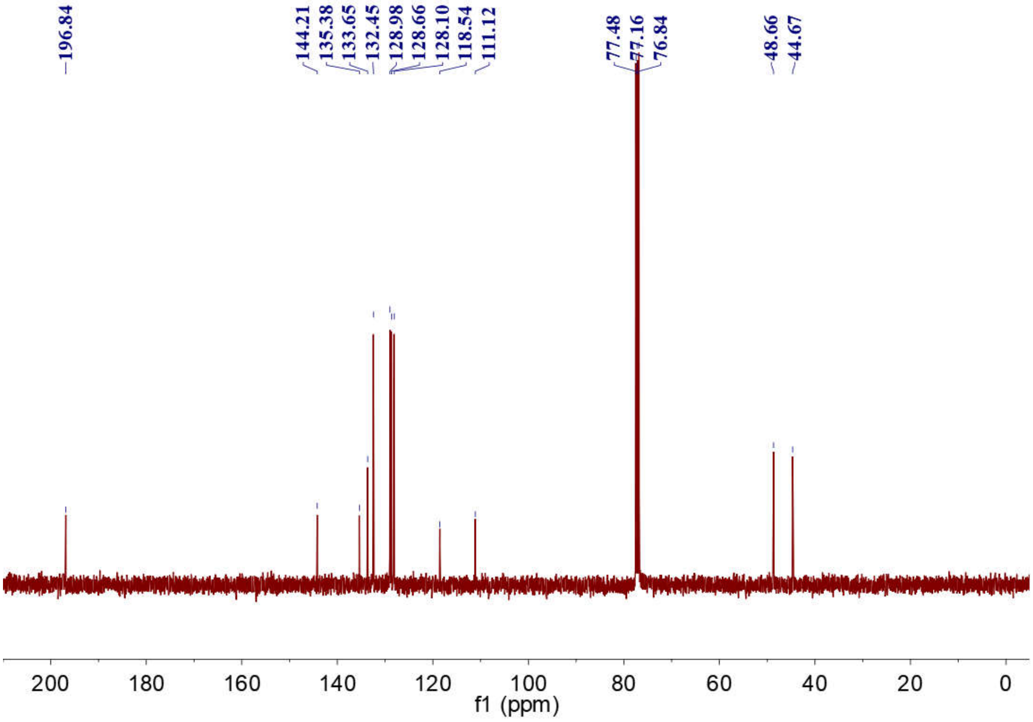

NMR spectra of product 11

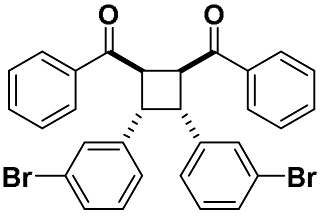

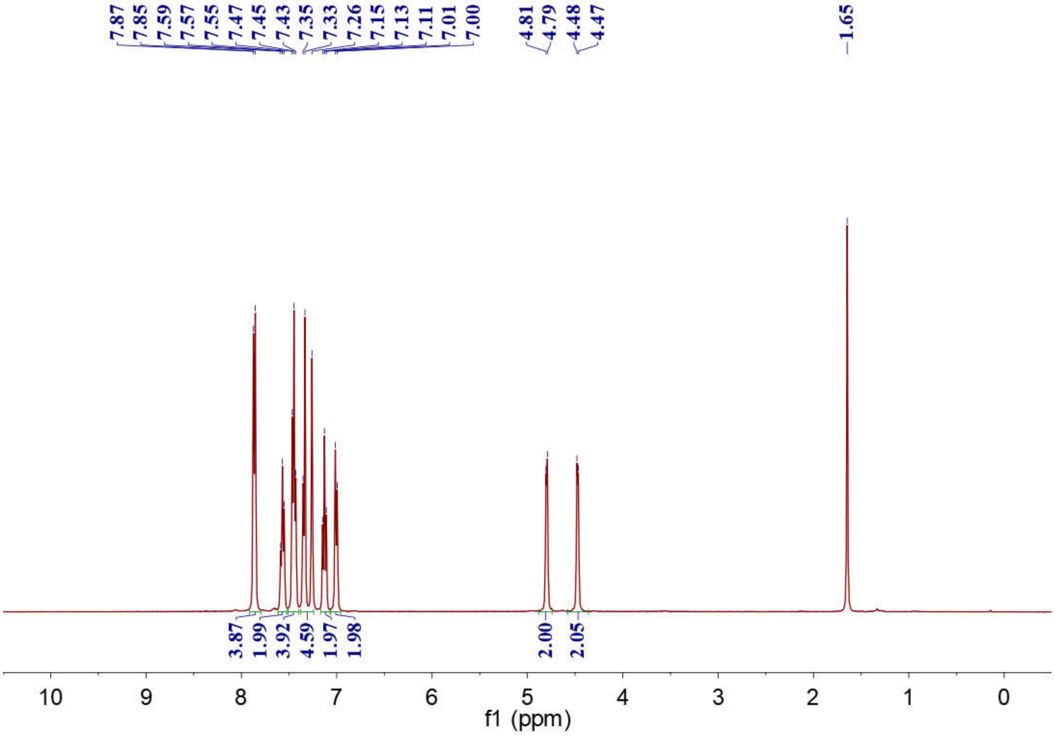

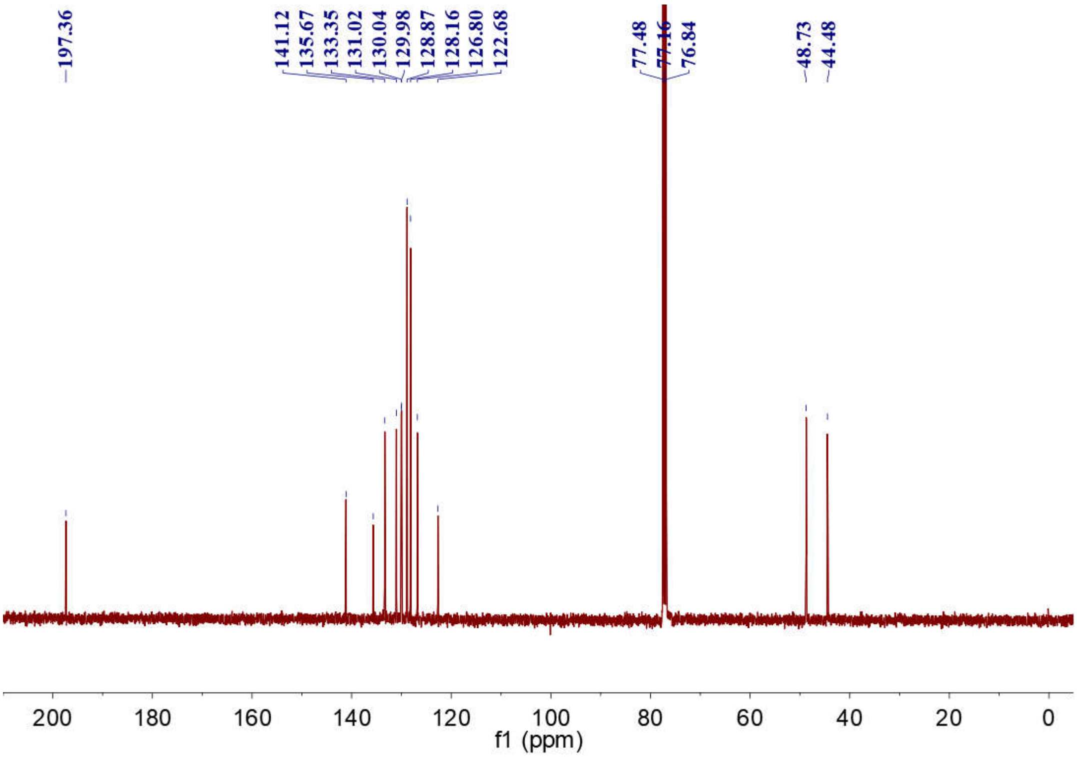

NMR spectra of product 12

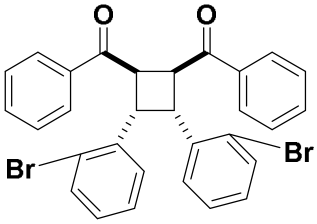

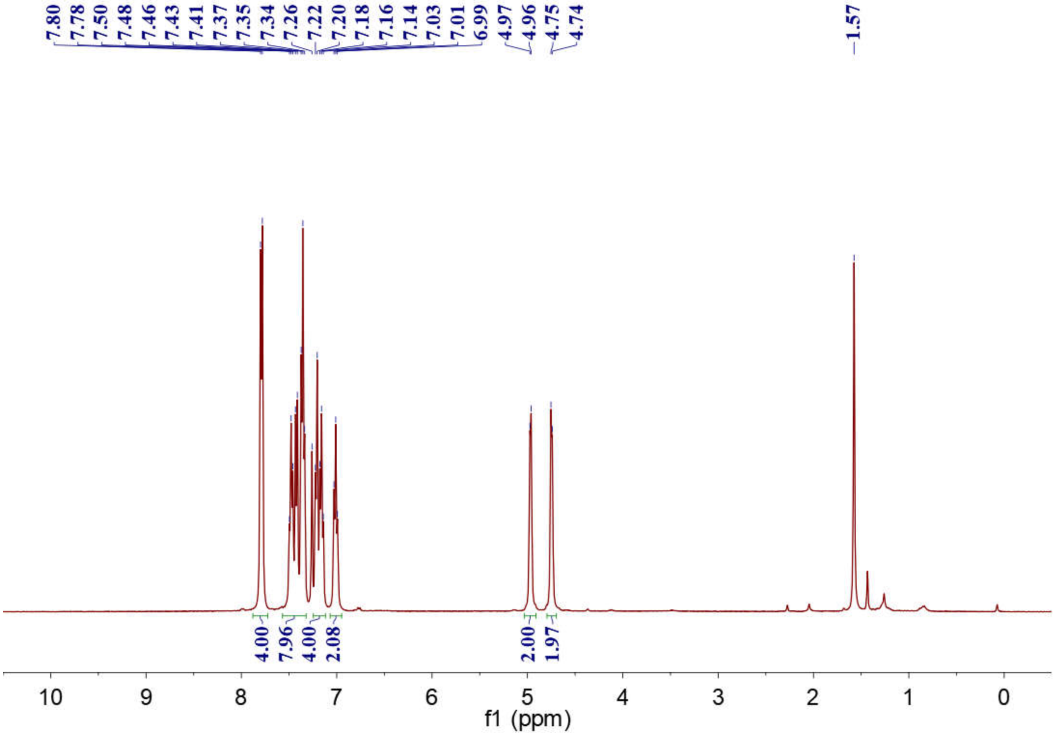

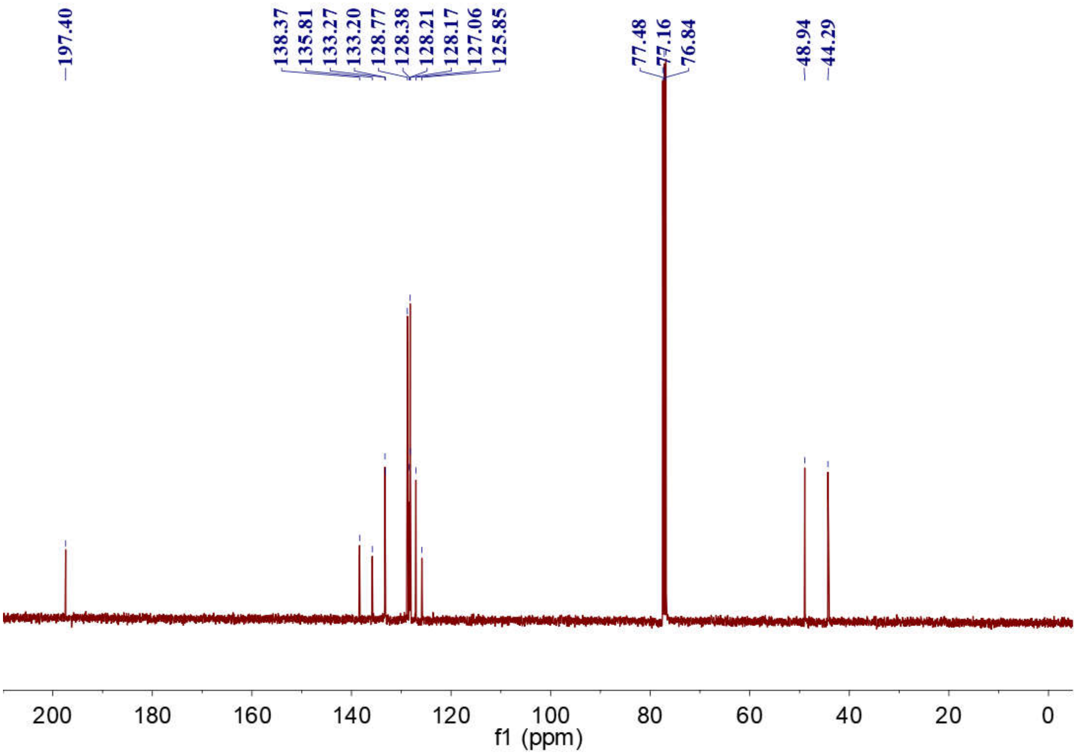

NMR spectra of product 13

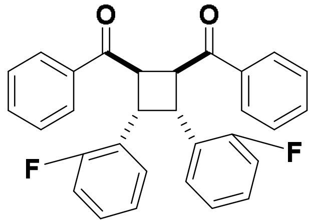

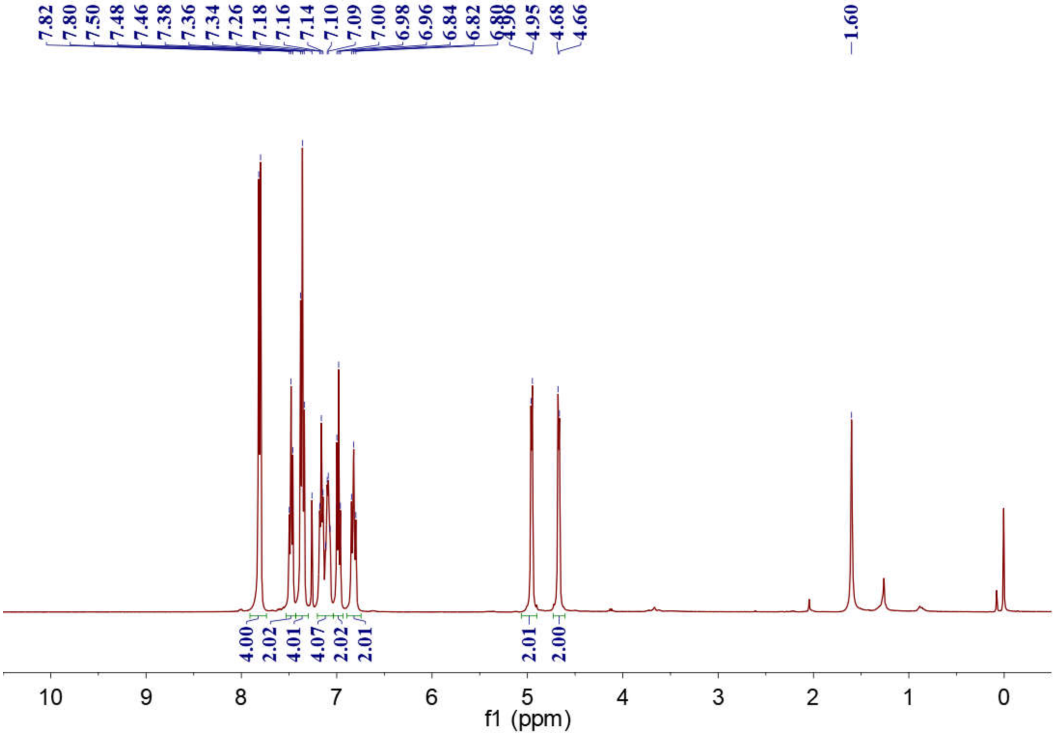

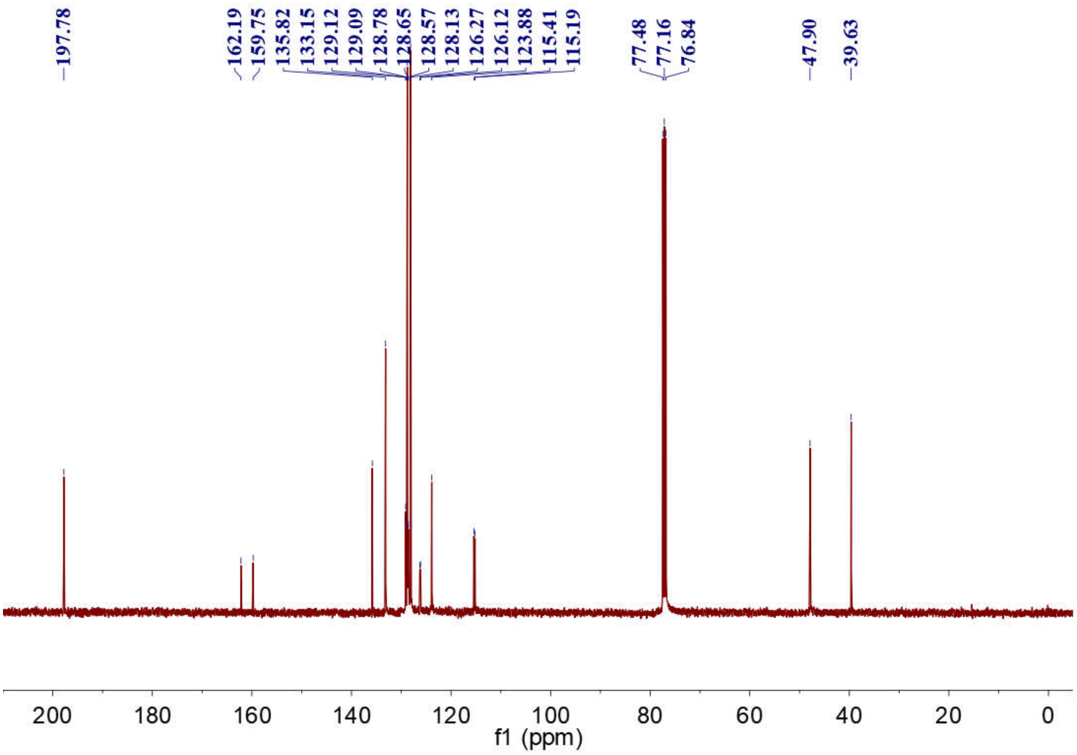

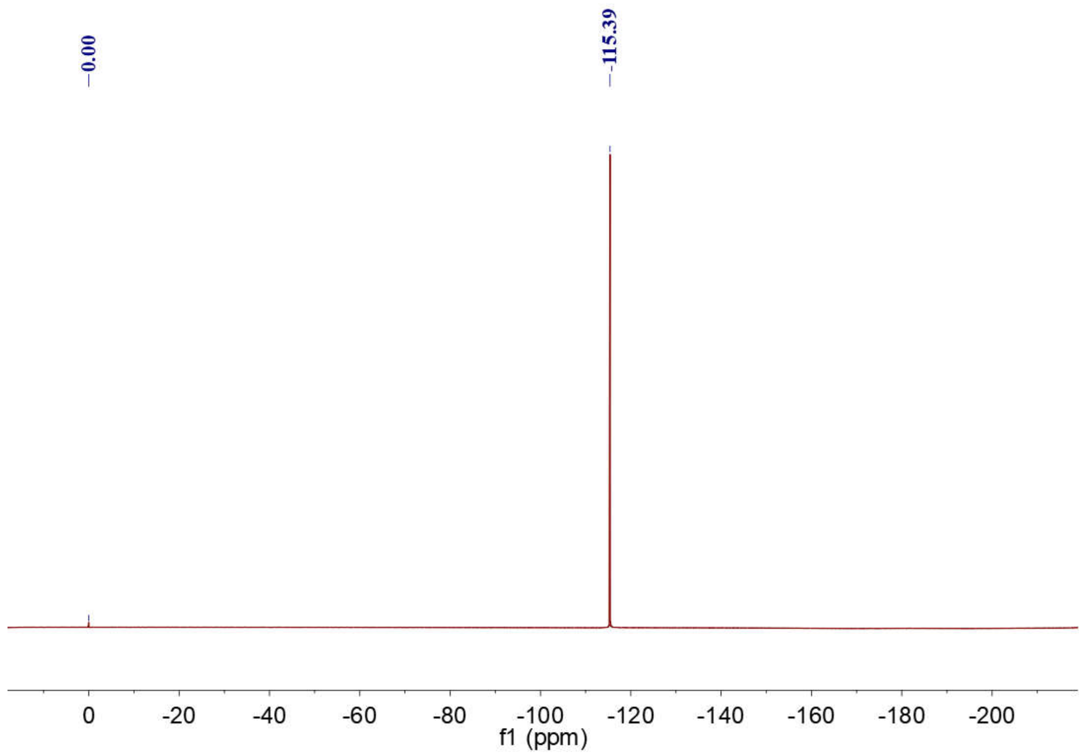

-0.00
--115.39
0
-20
-40
-60
-80
-100
-120
-140
-160
-180
-200
f1 (ppm)

NMR spectra of product 14

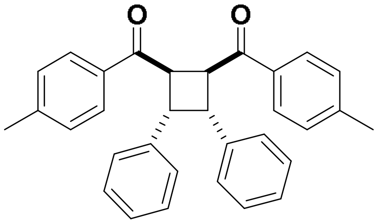

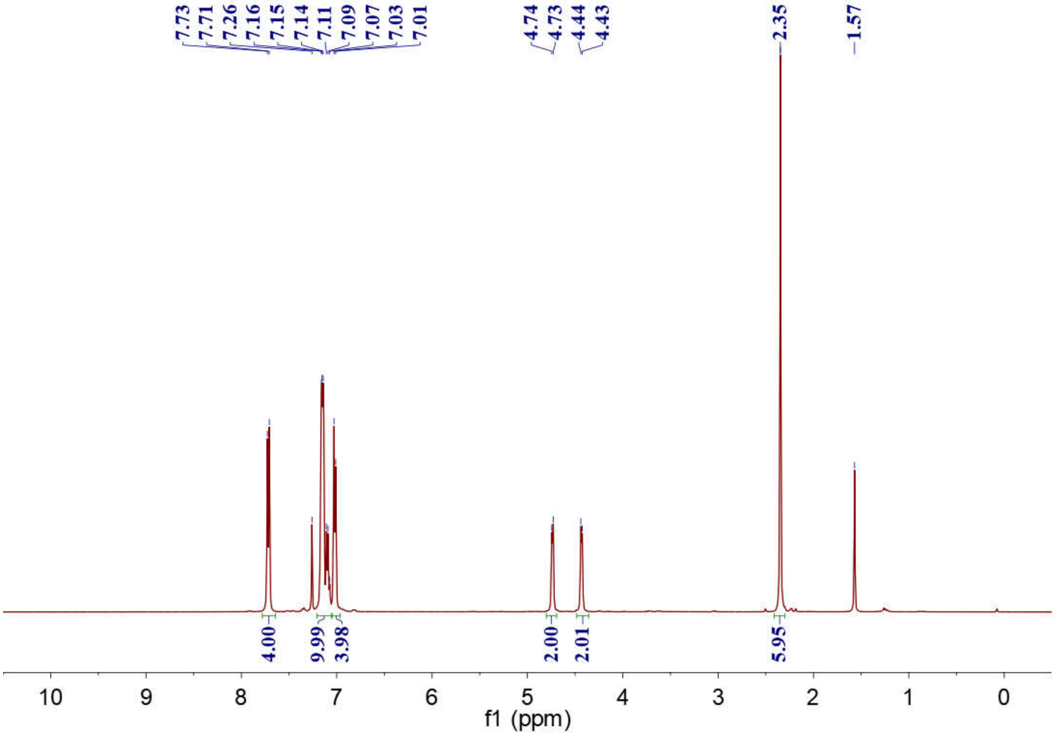

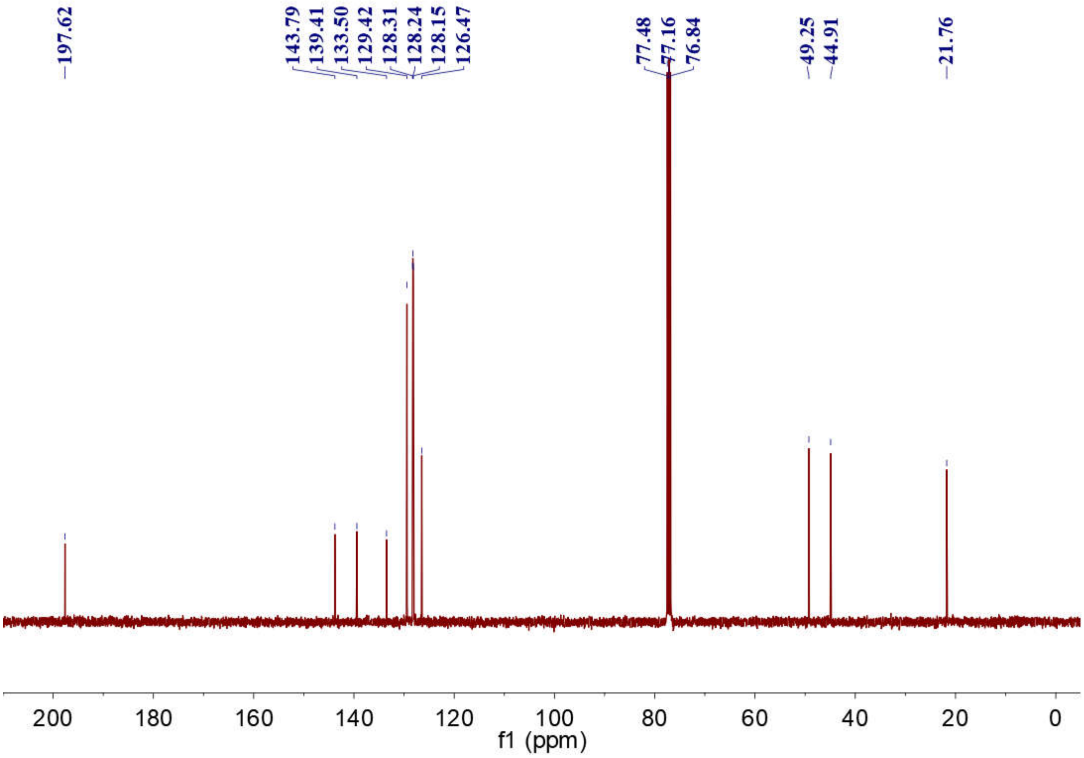

NMR spectra of product 15

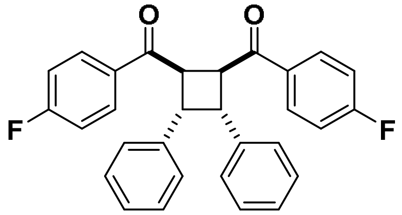

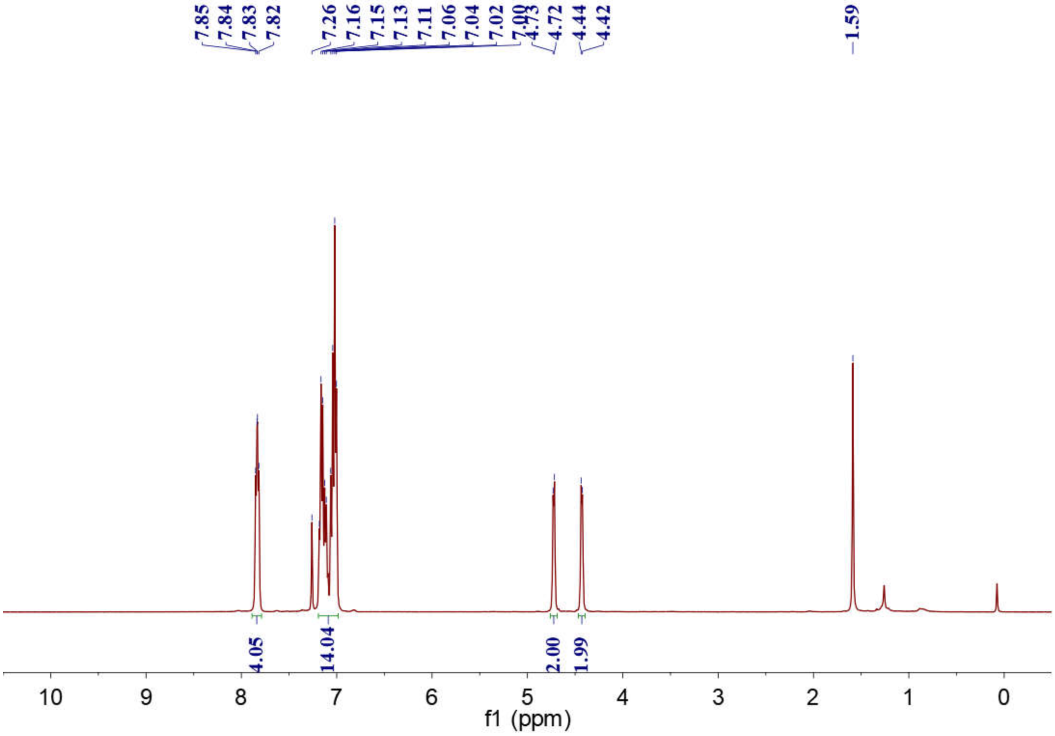

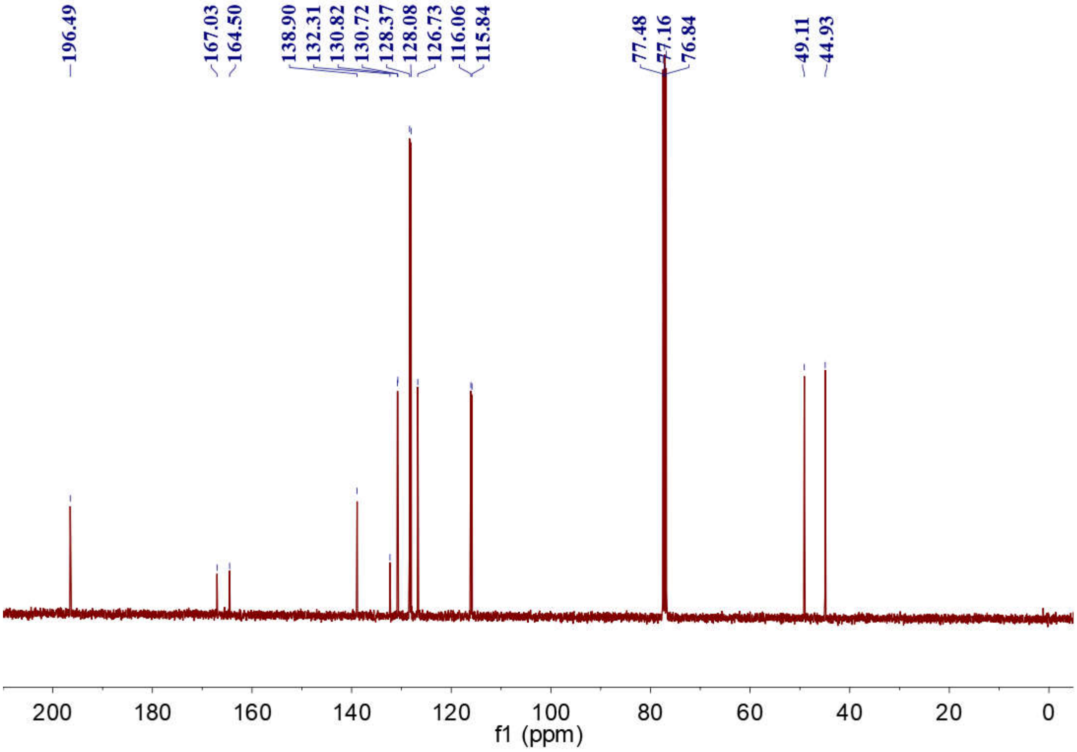

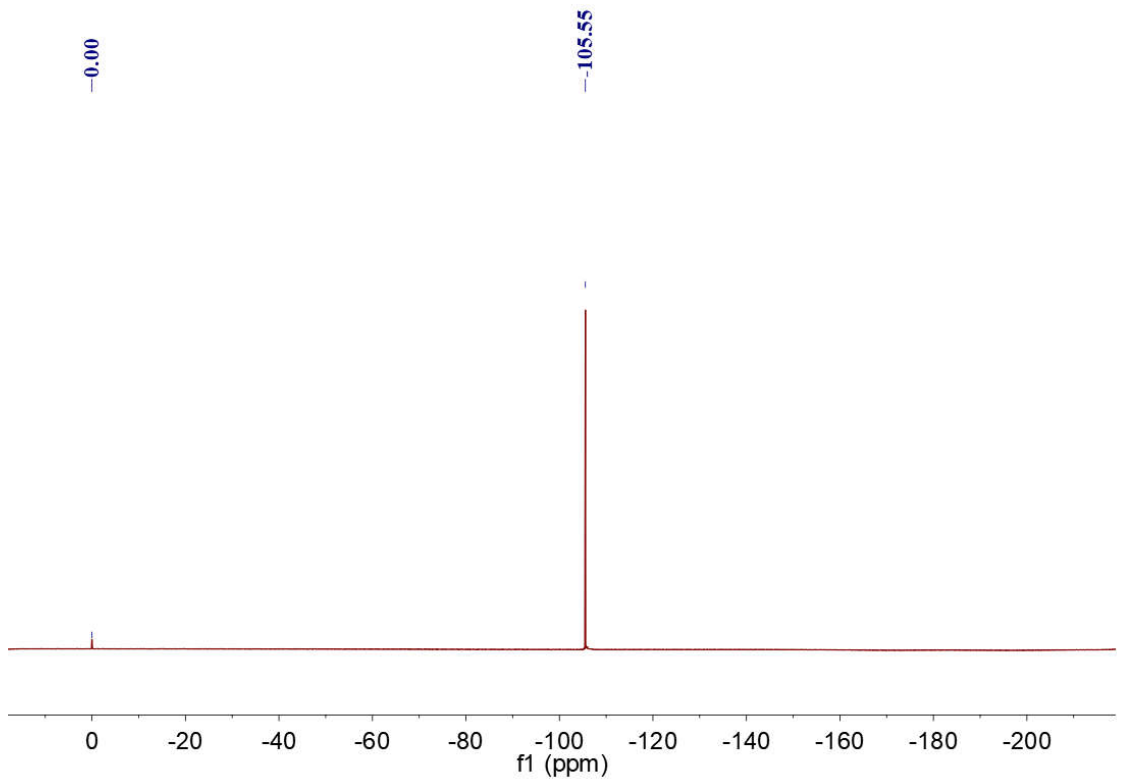
0.00
-105.55
0
-20
-40
-60
-80
-100
-120
-140
-160
-180
-200
f1 (ppm)

NMR spectra of product 16

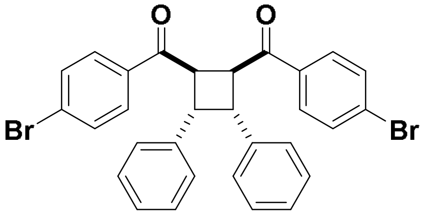

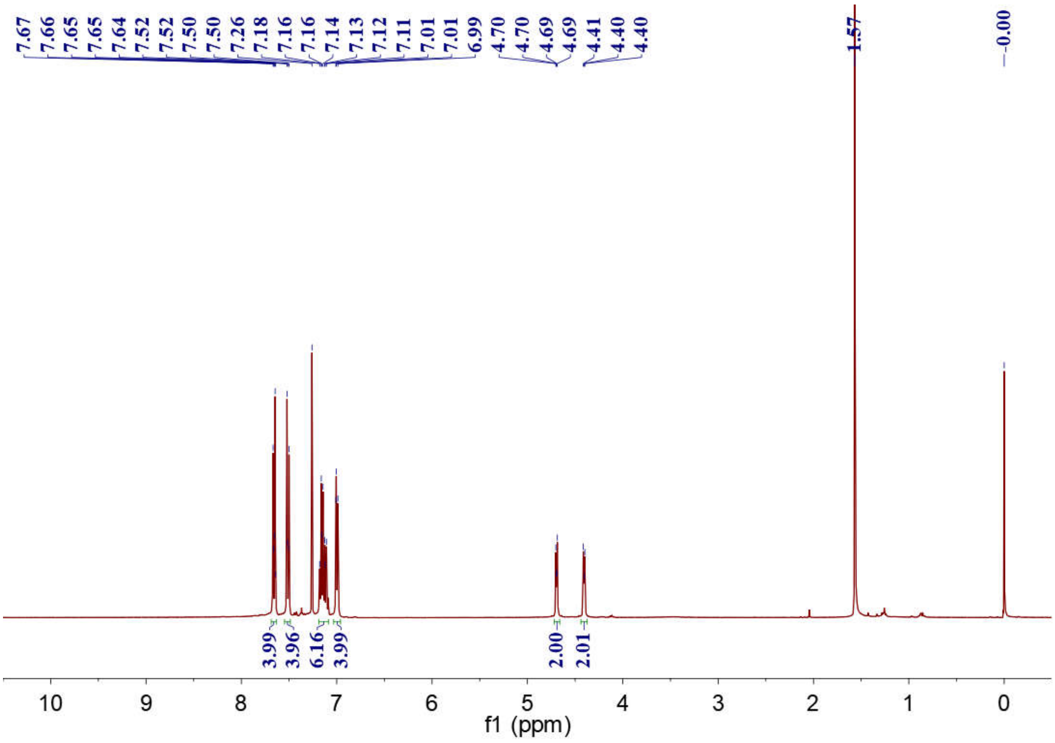

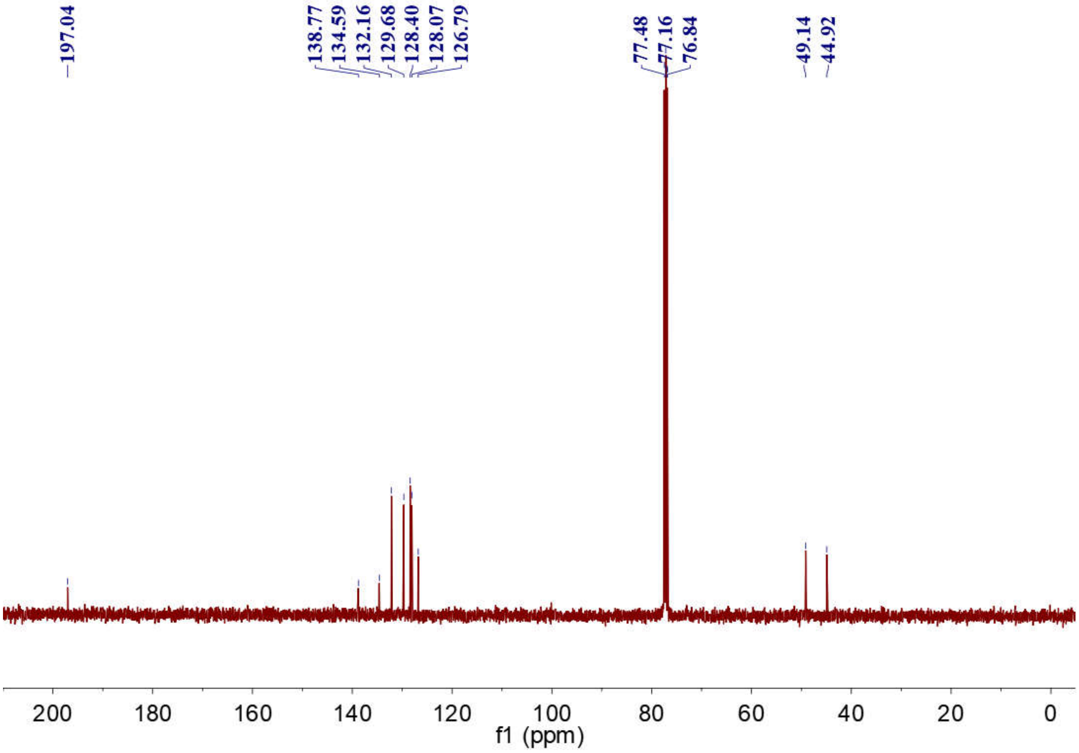

NMR spectra of product 17

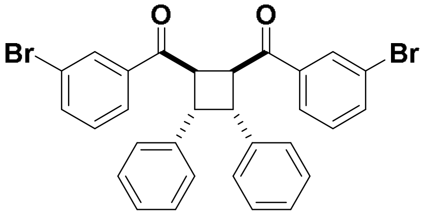

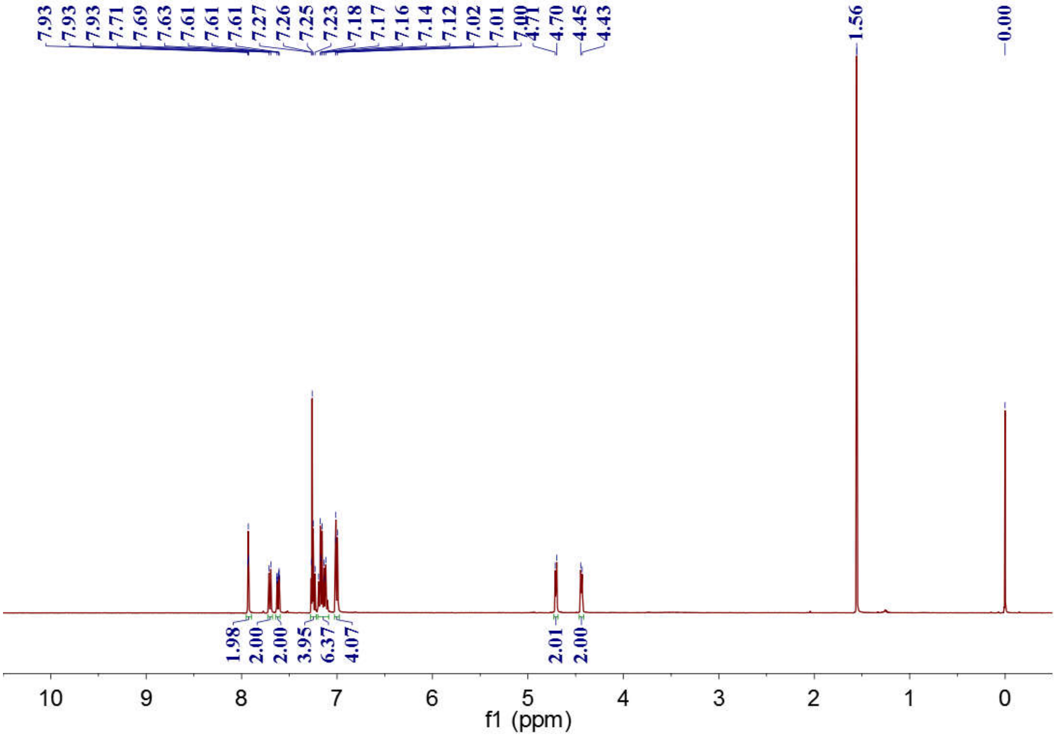

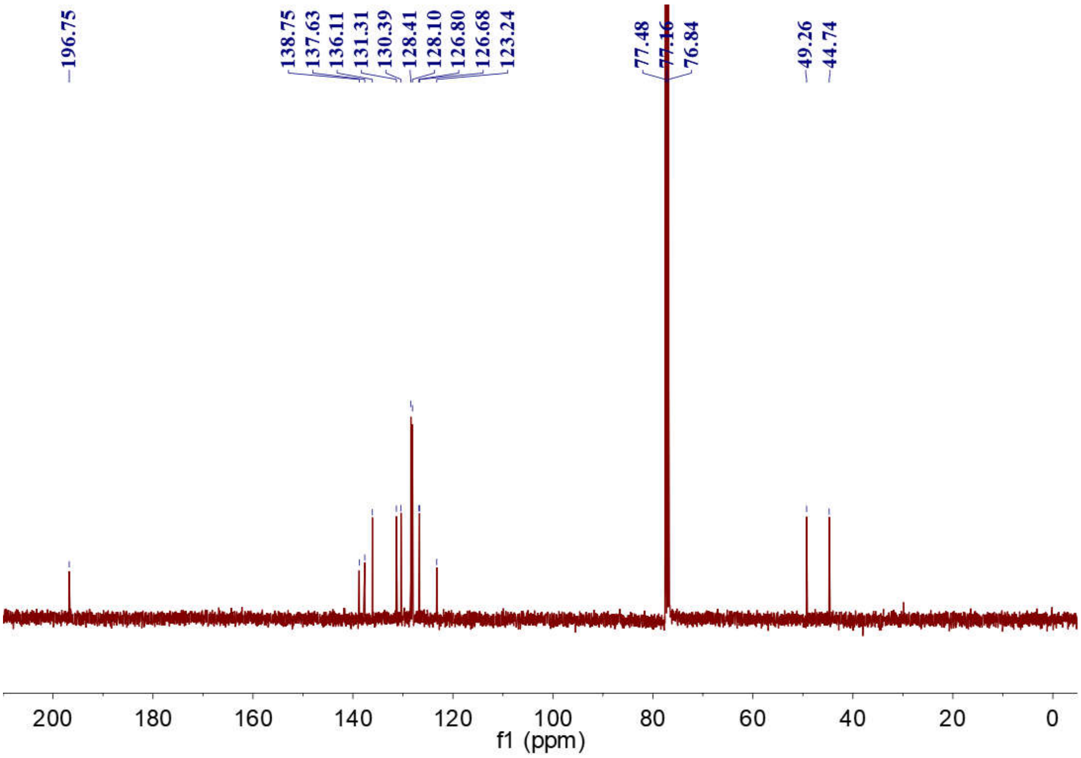

NMR spectra of product 18

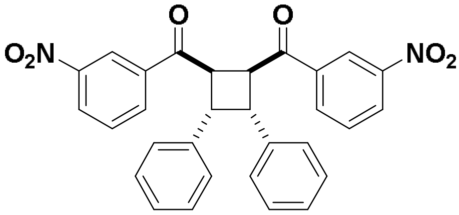

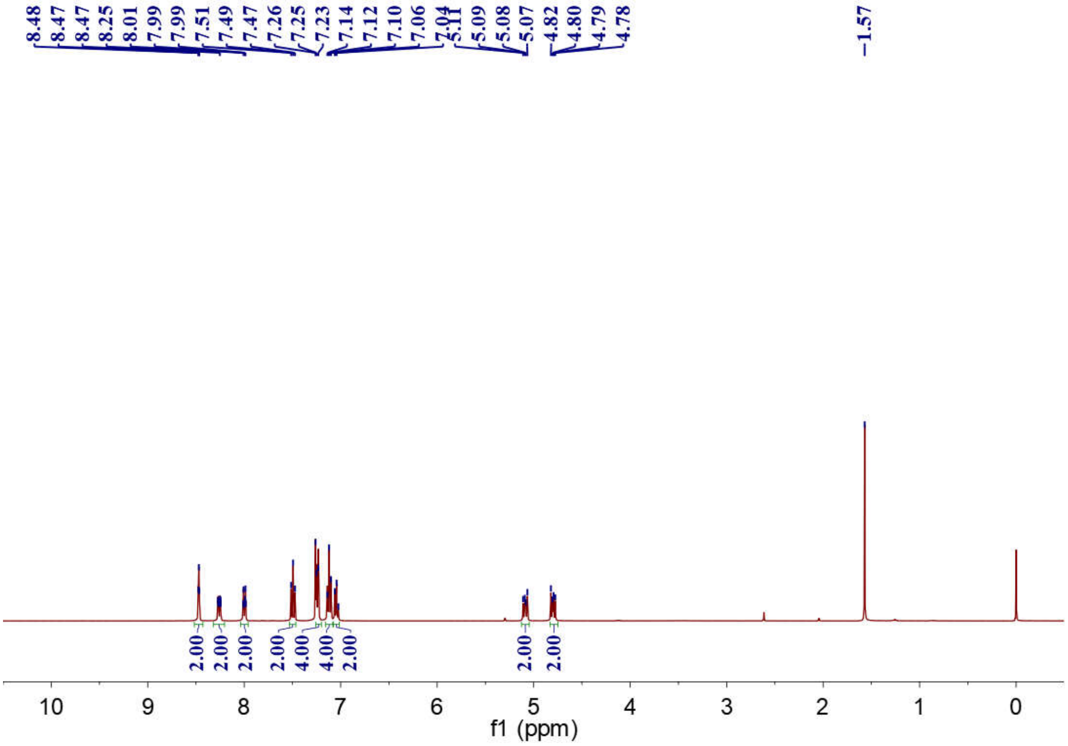

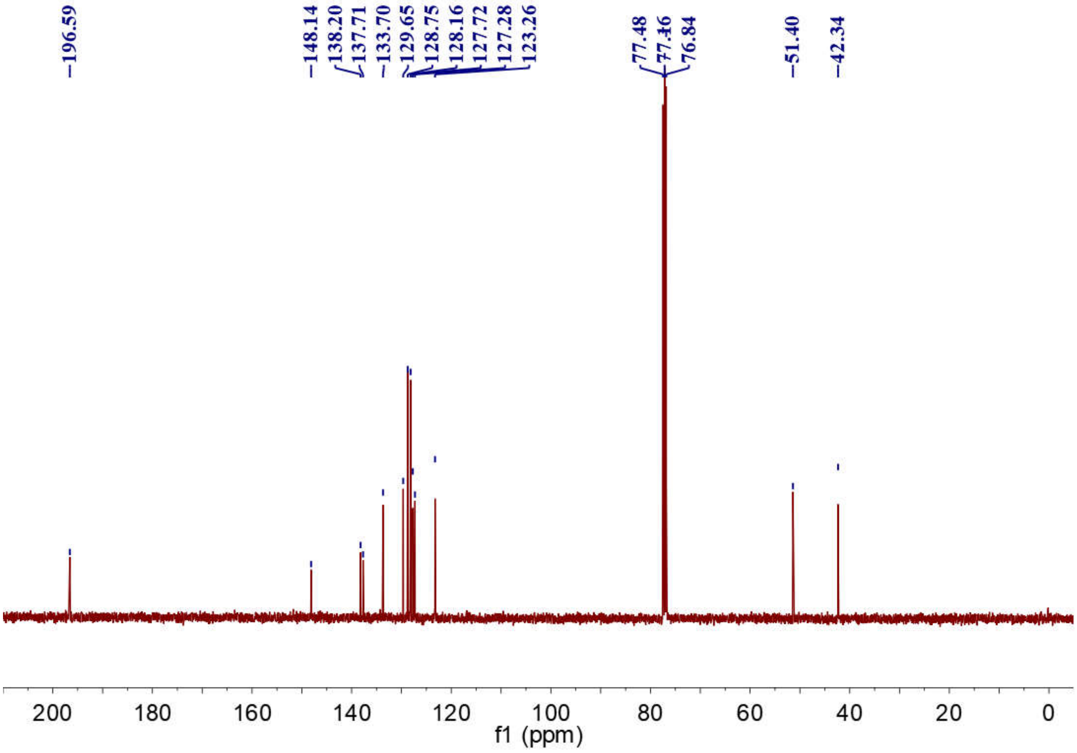

NMR spectra of product 19

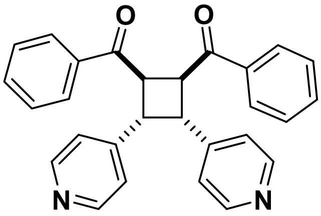

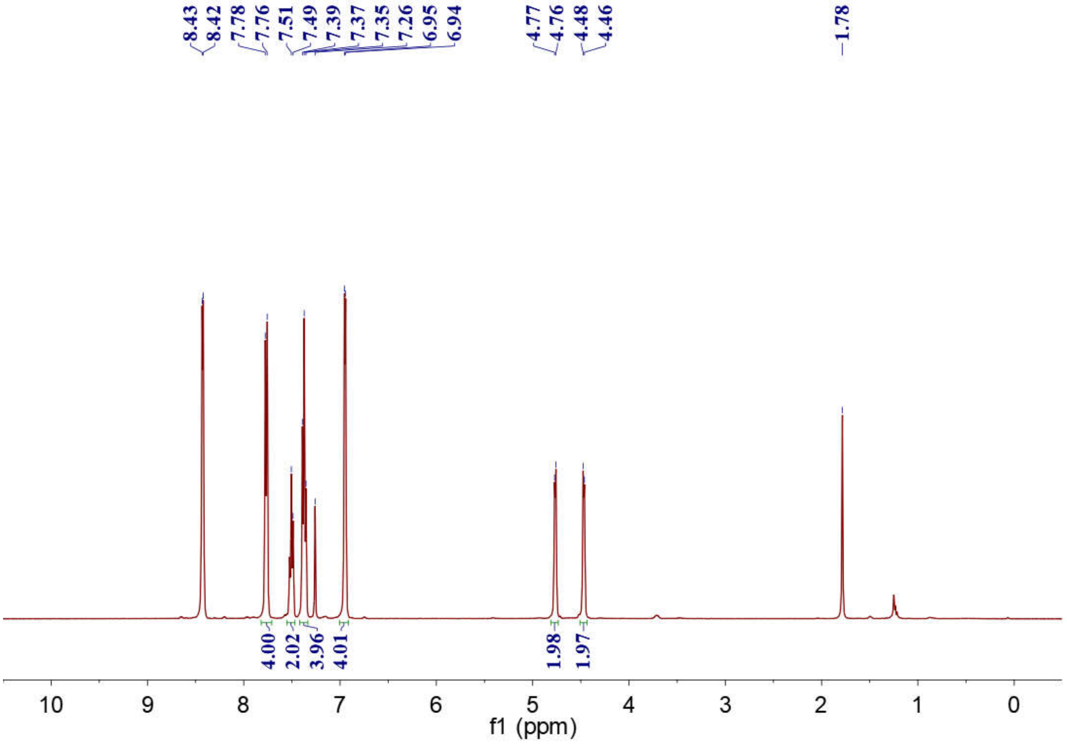

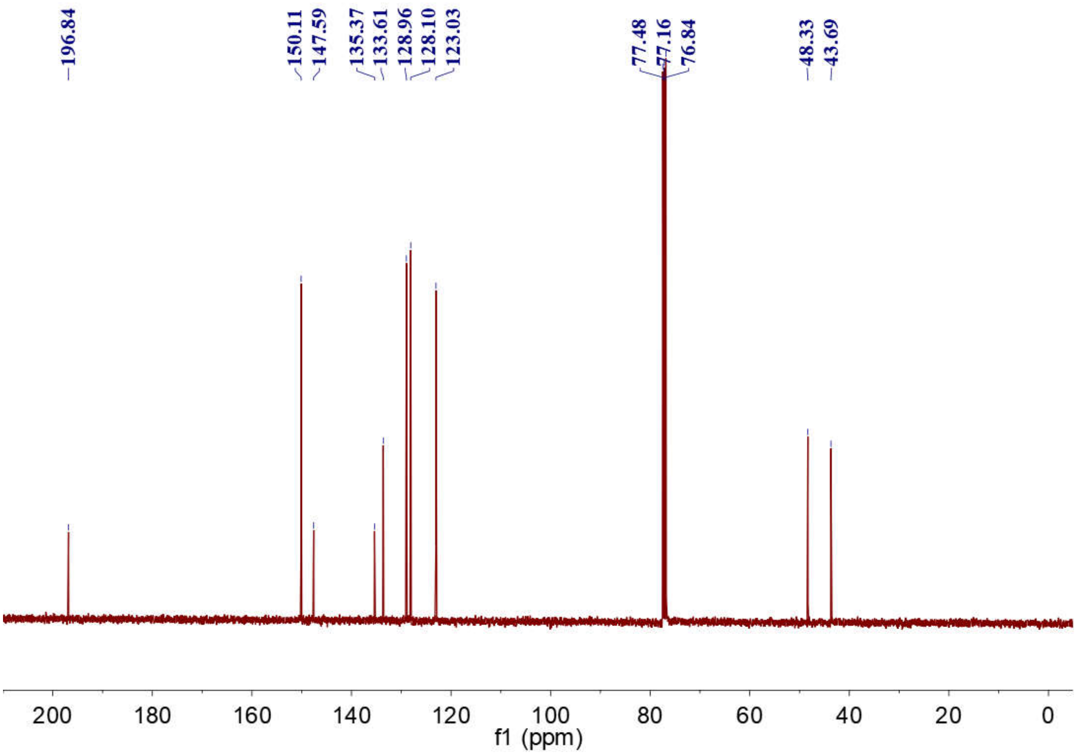

NMR spectra of product 20

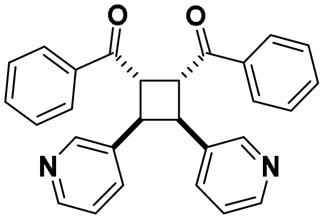

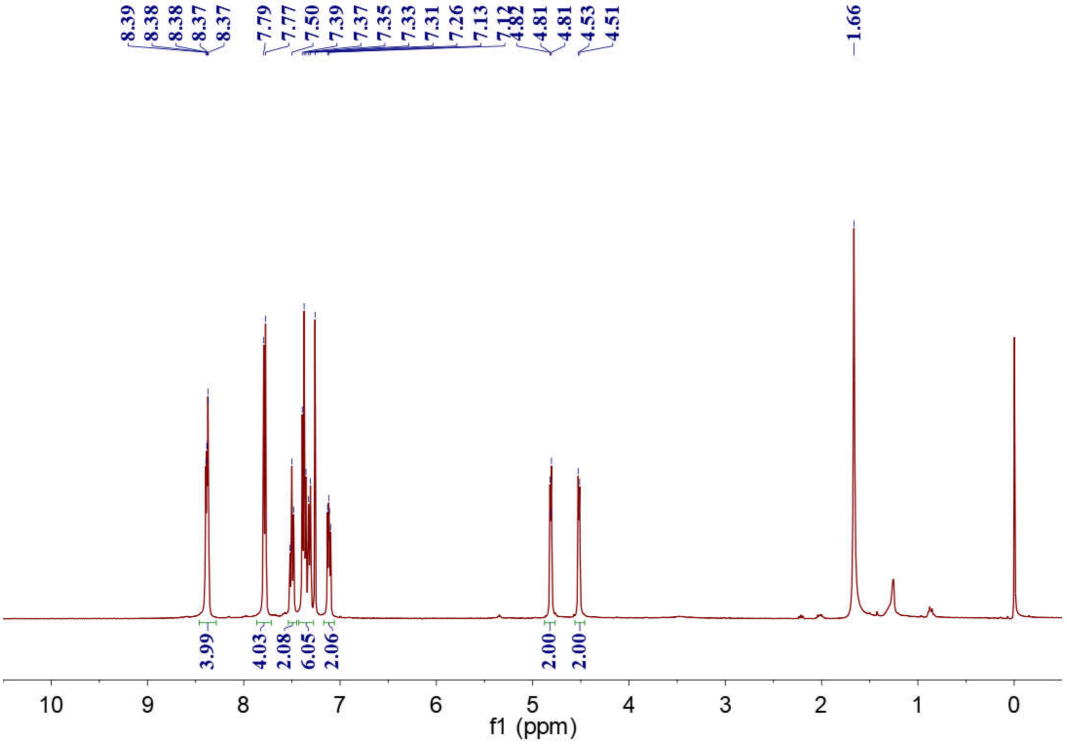

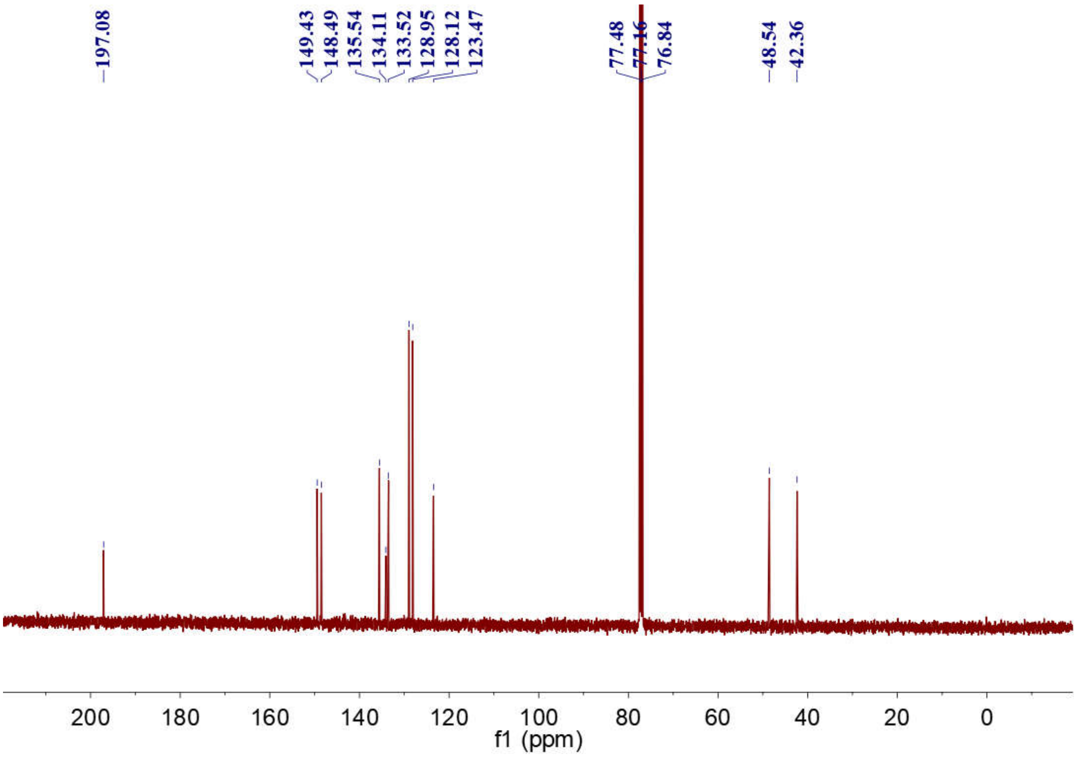

NMR spectra of product 21

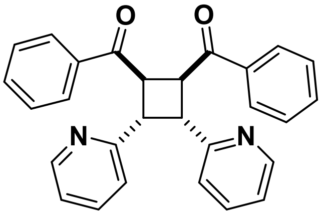

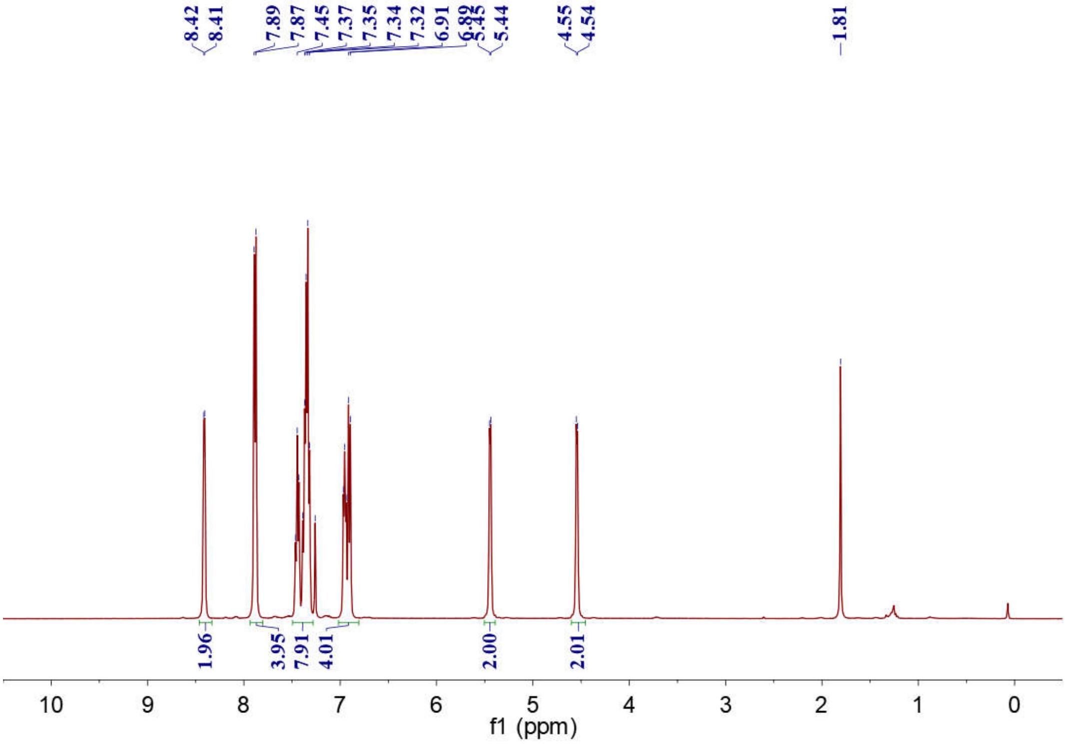

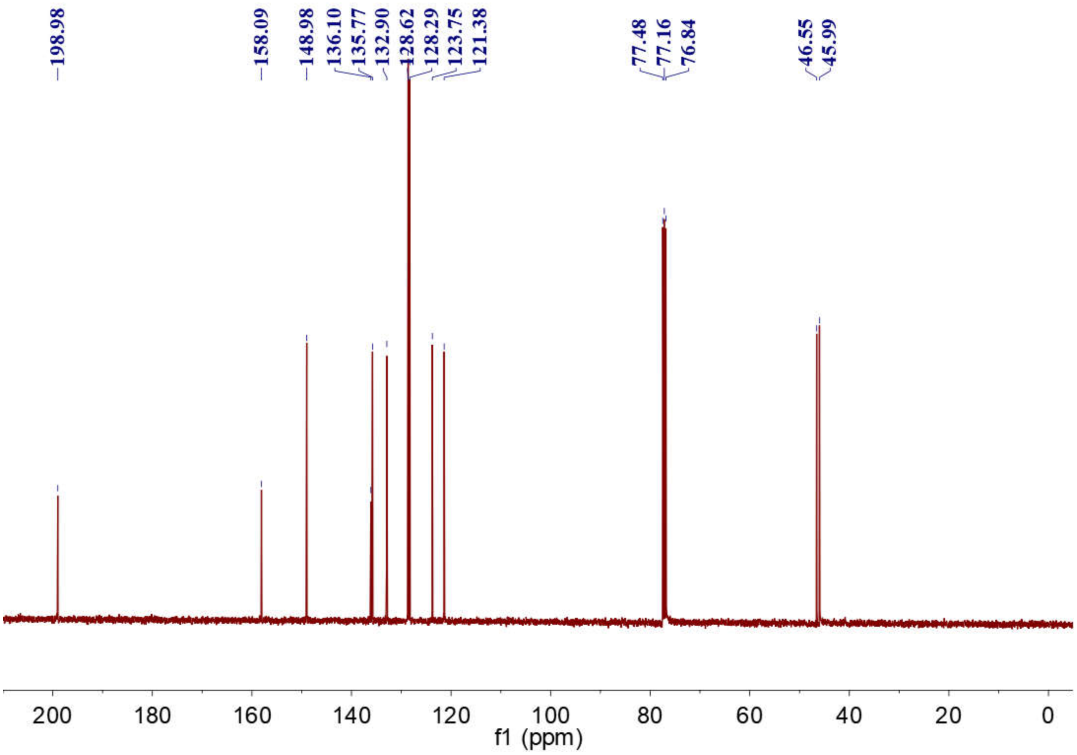

NMR spectra of product 22

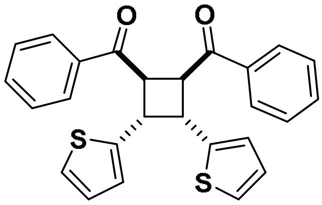

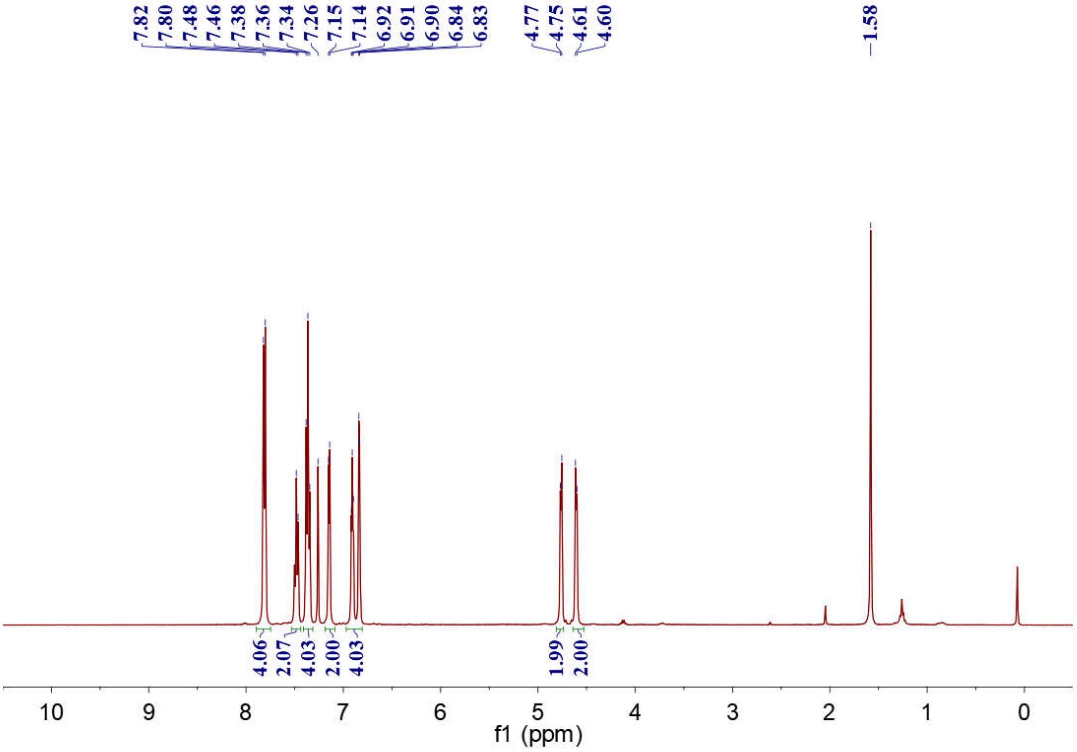

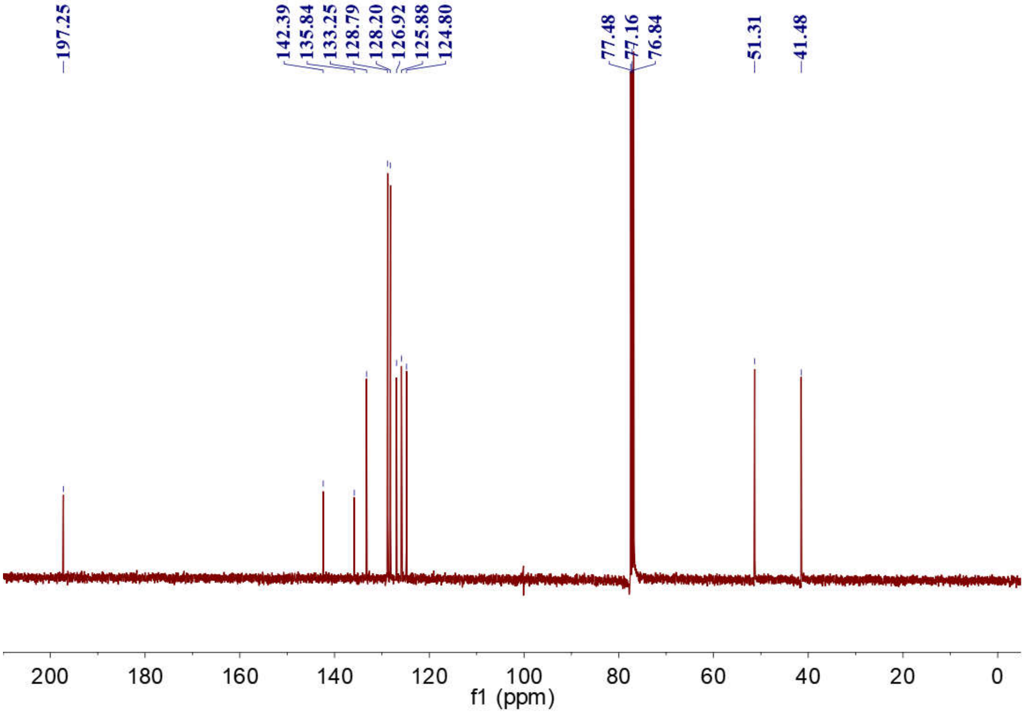

NMR spectra of product 23

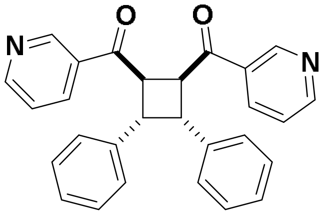

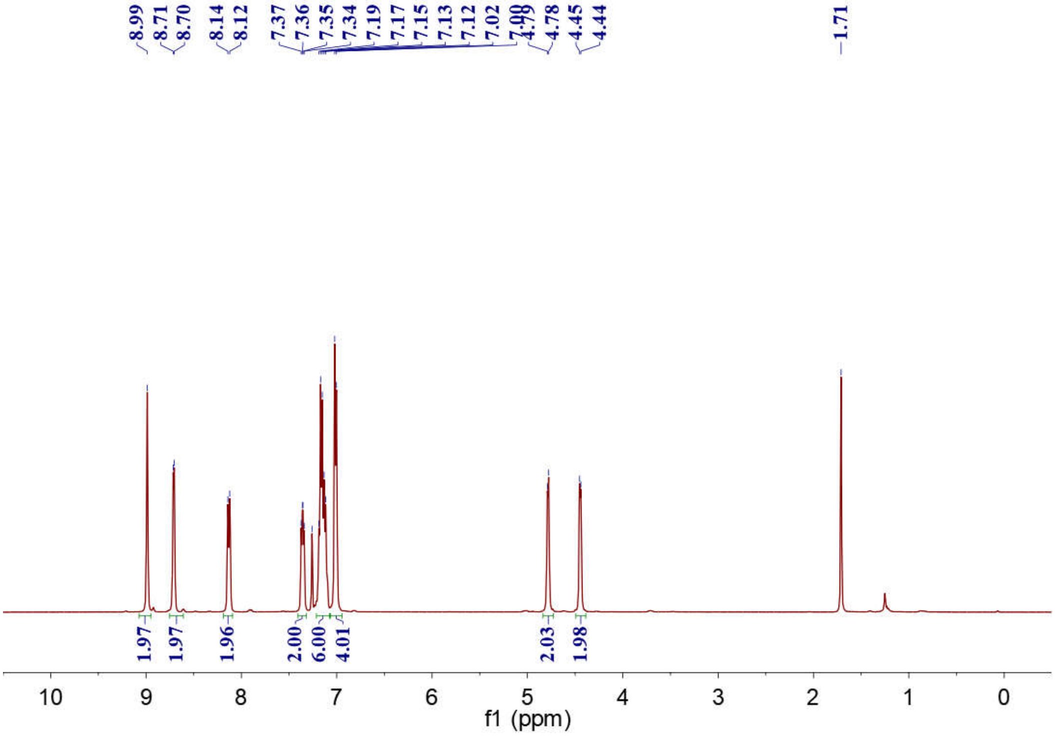

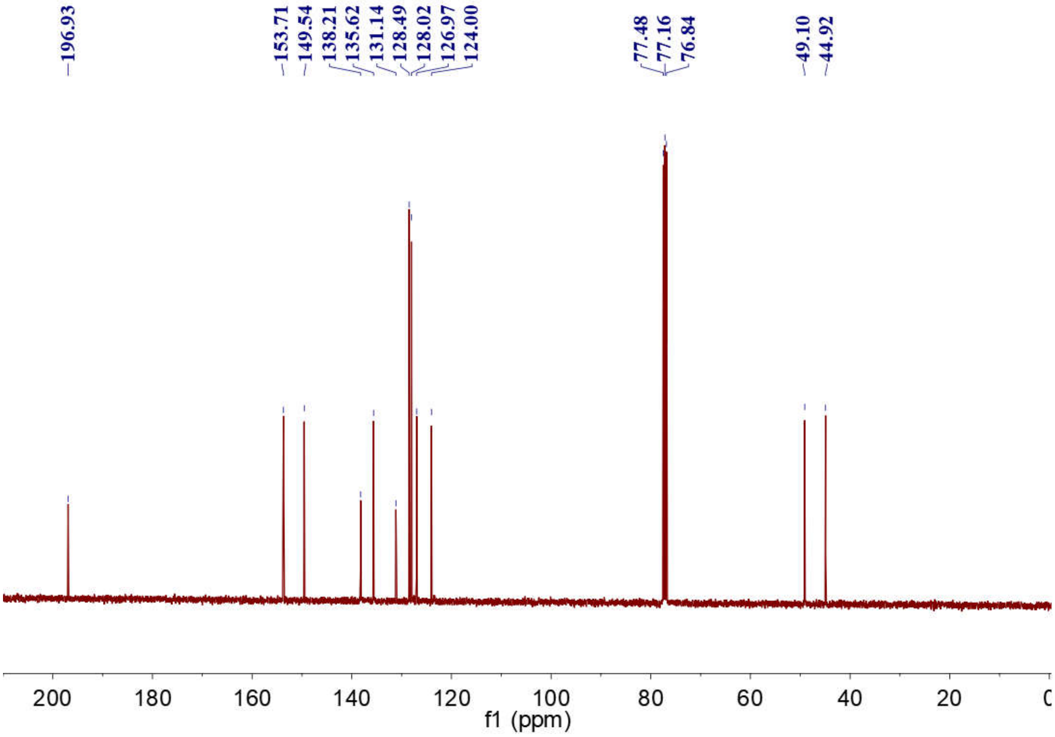

NMR spectra of product 24

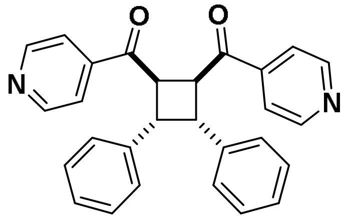

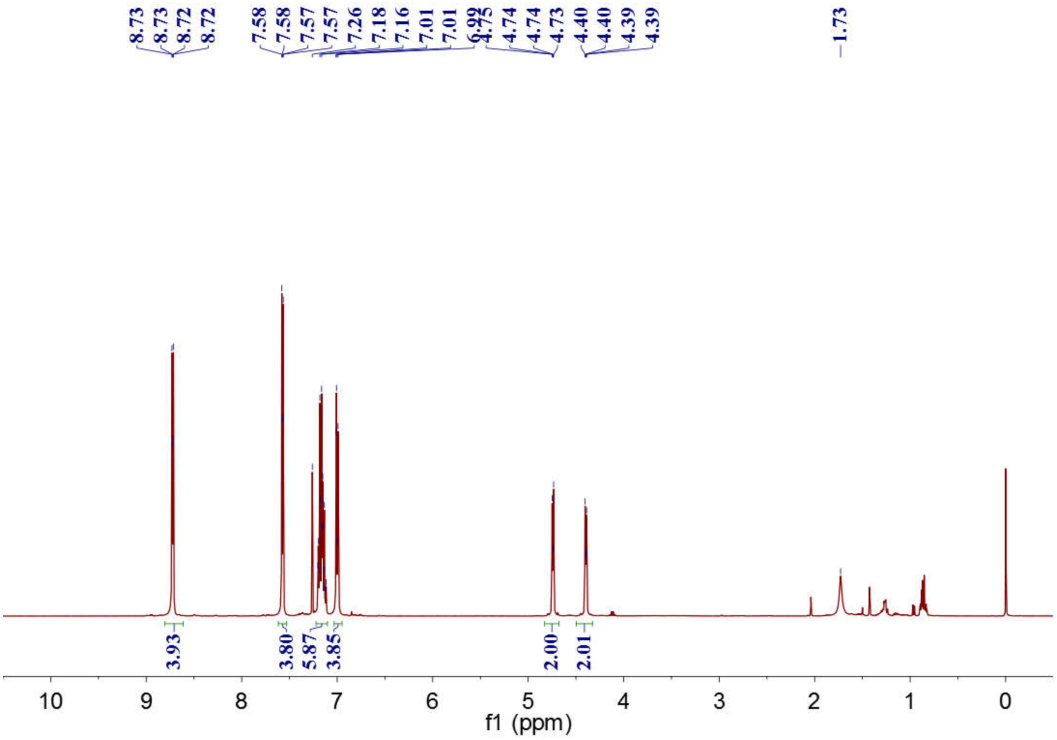

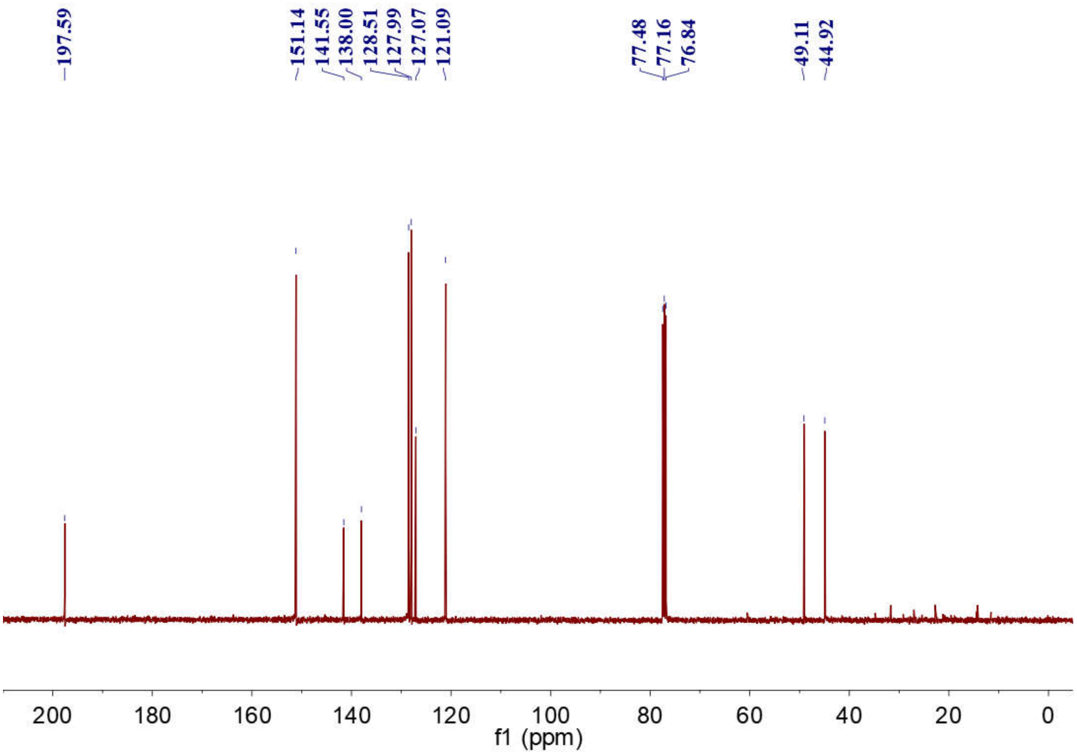

NMR spectra of product 25

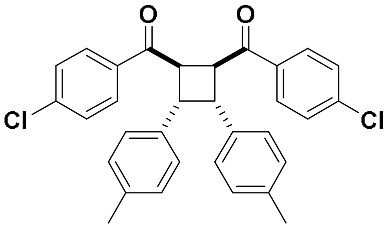

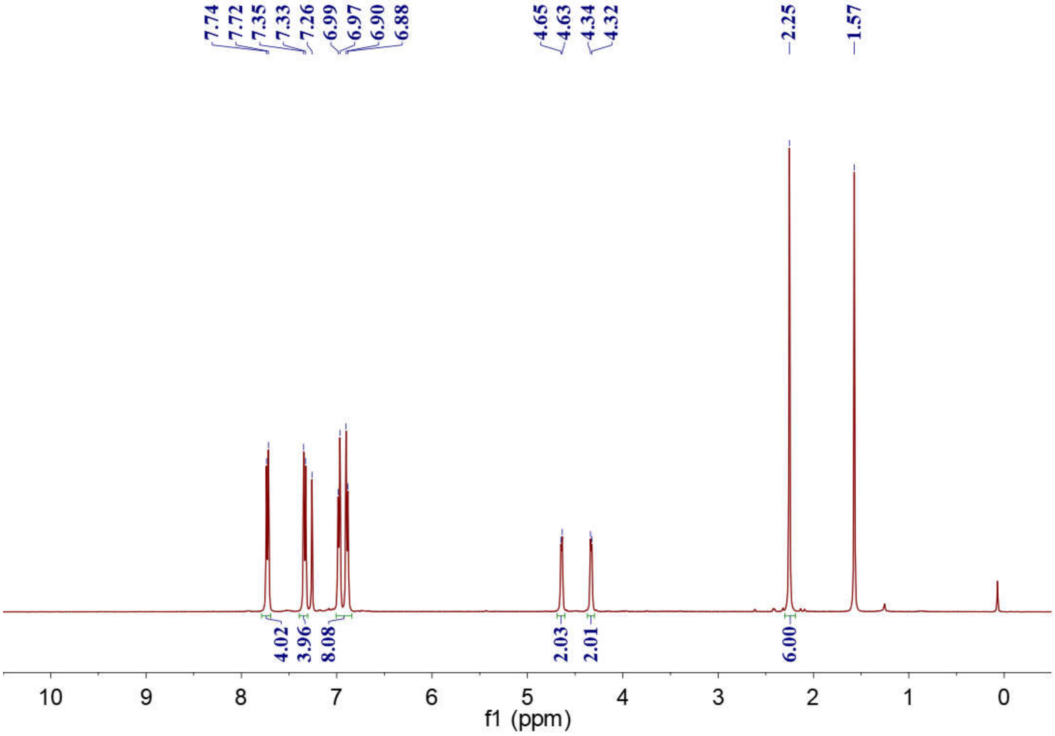

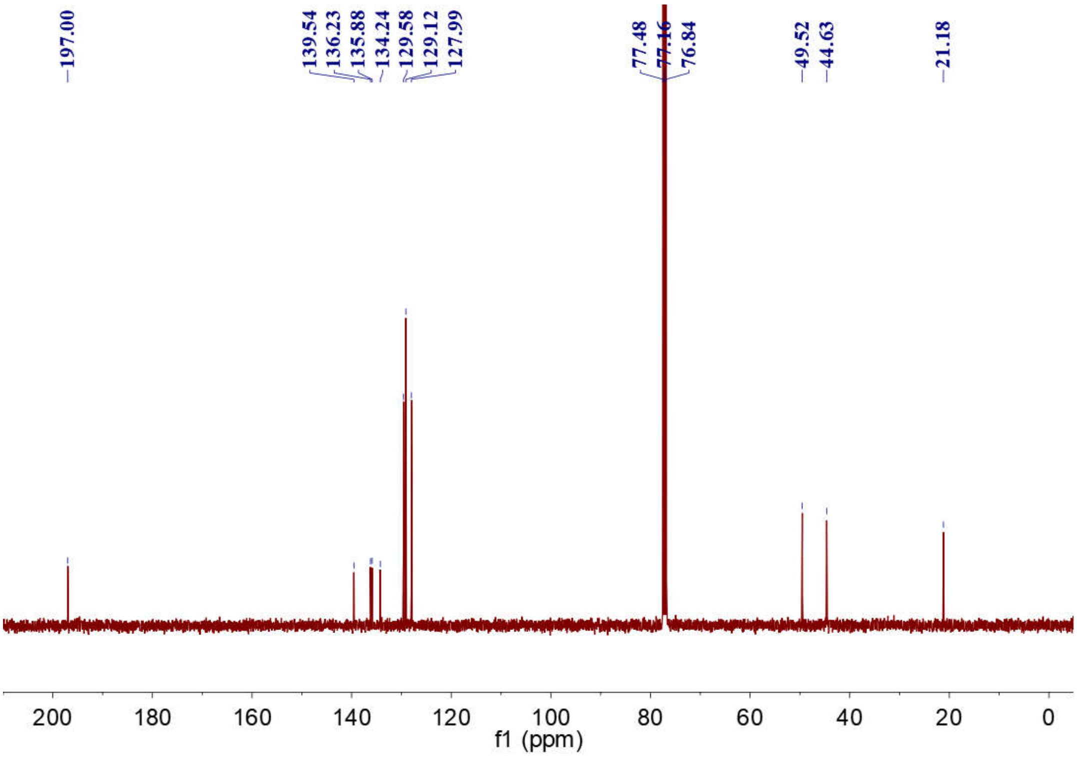

NMR spectra of product 26

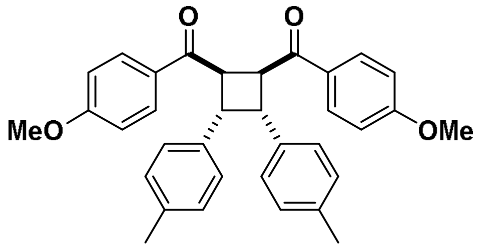

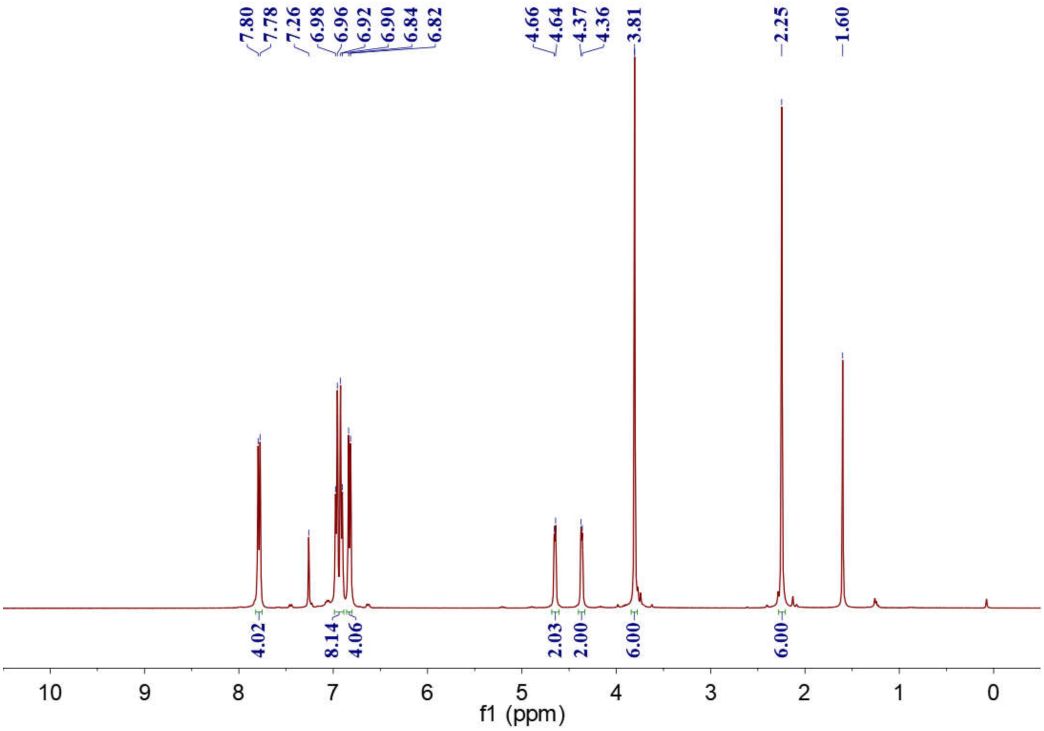

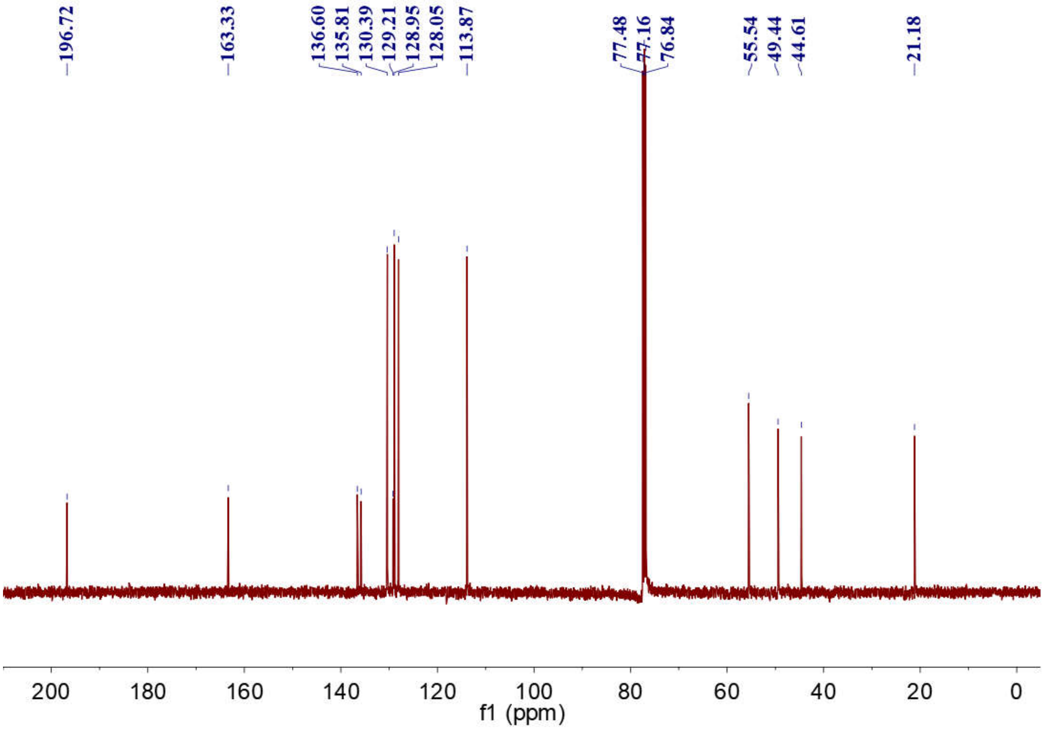

NMR spectra of product 27

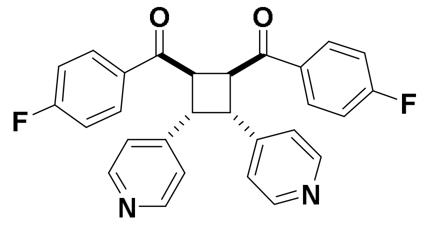

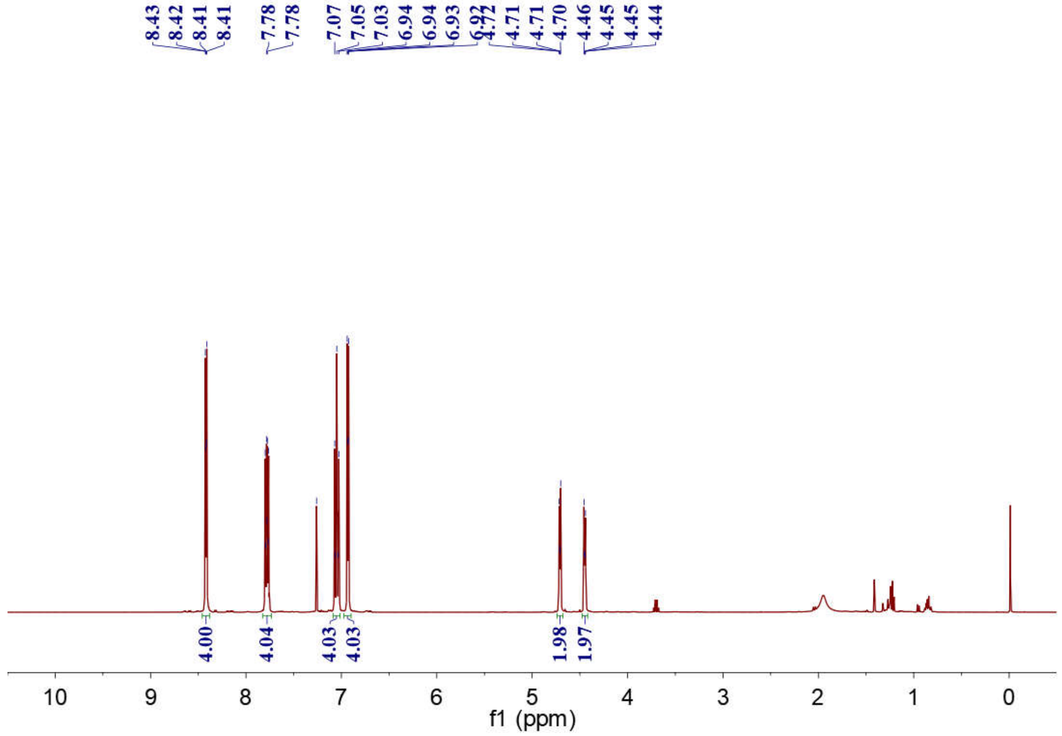

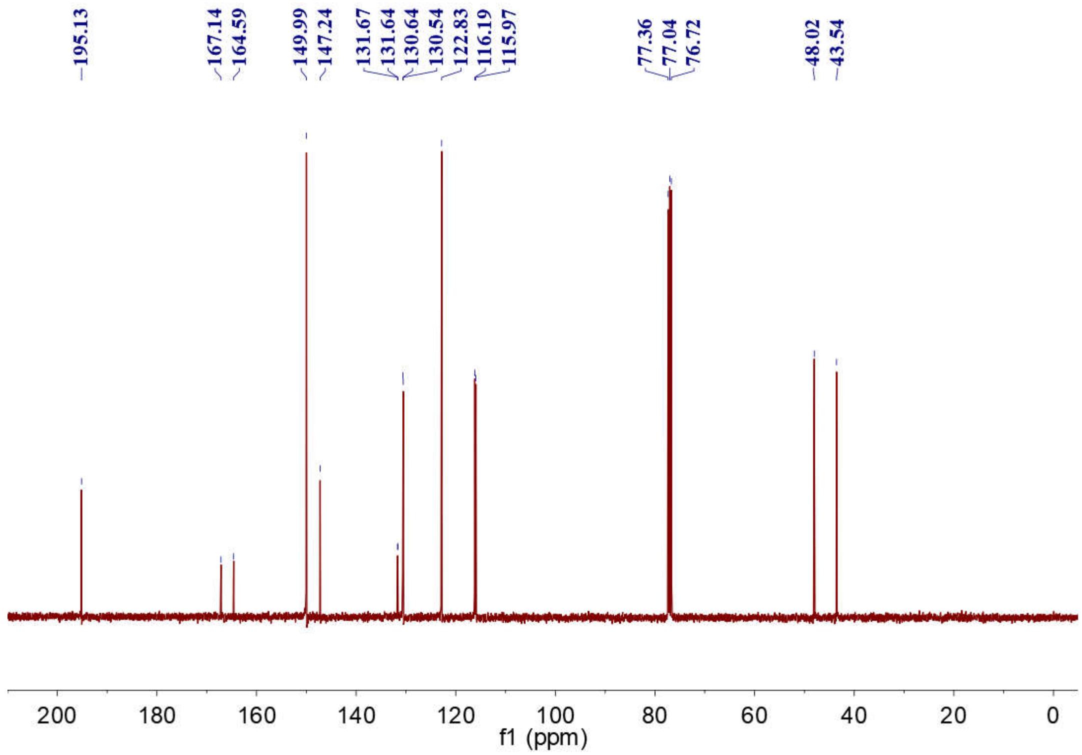

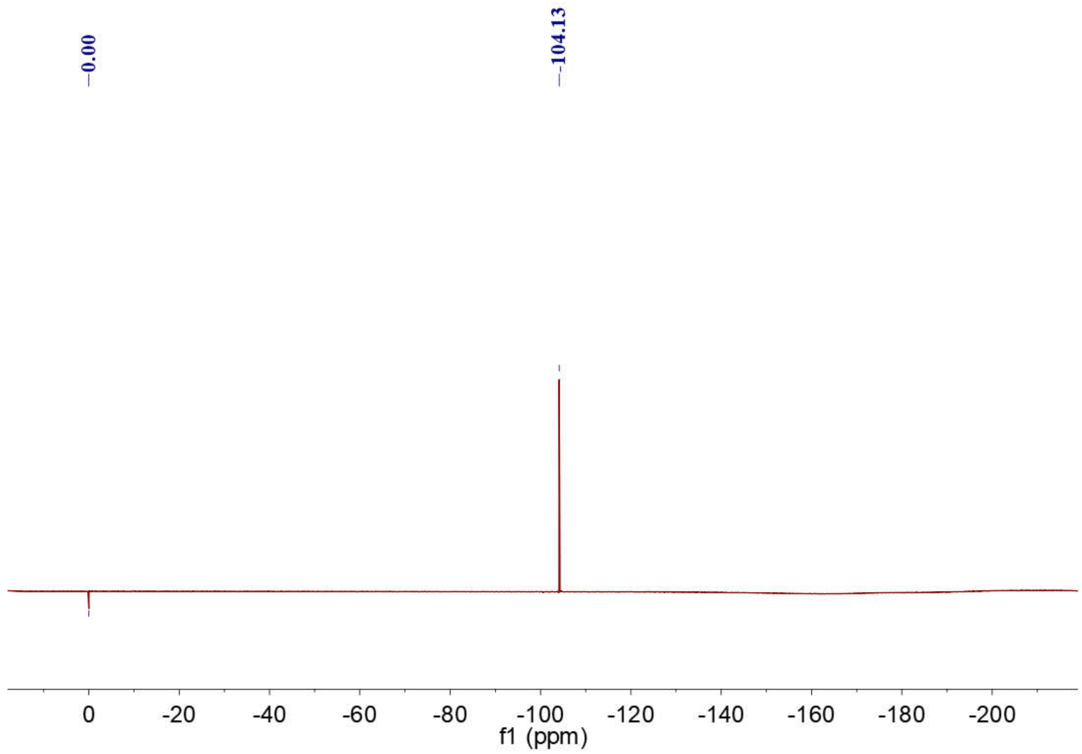

-0.00
-104.13
0
-20
-40
-60
-80
-100
-120
-140
-160
-180
-200
f1 (ppm)

NMR spectra of product 28

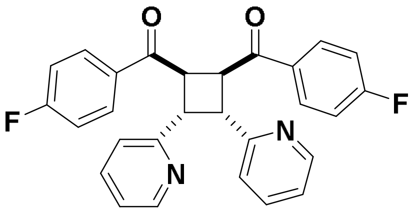

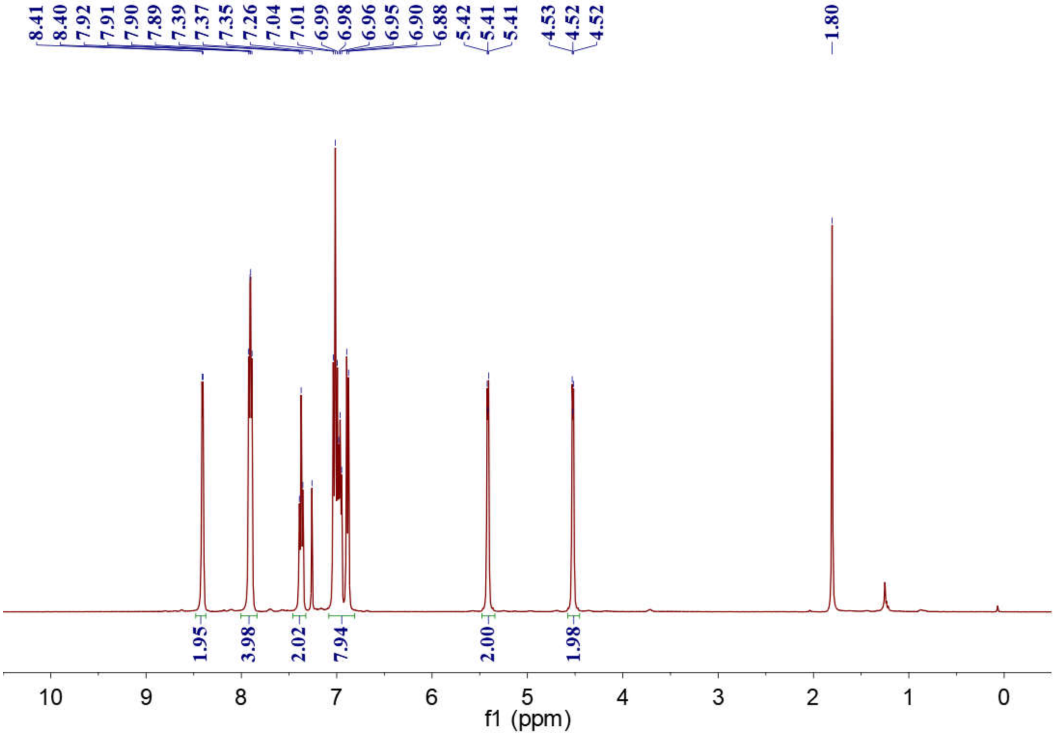

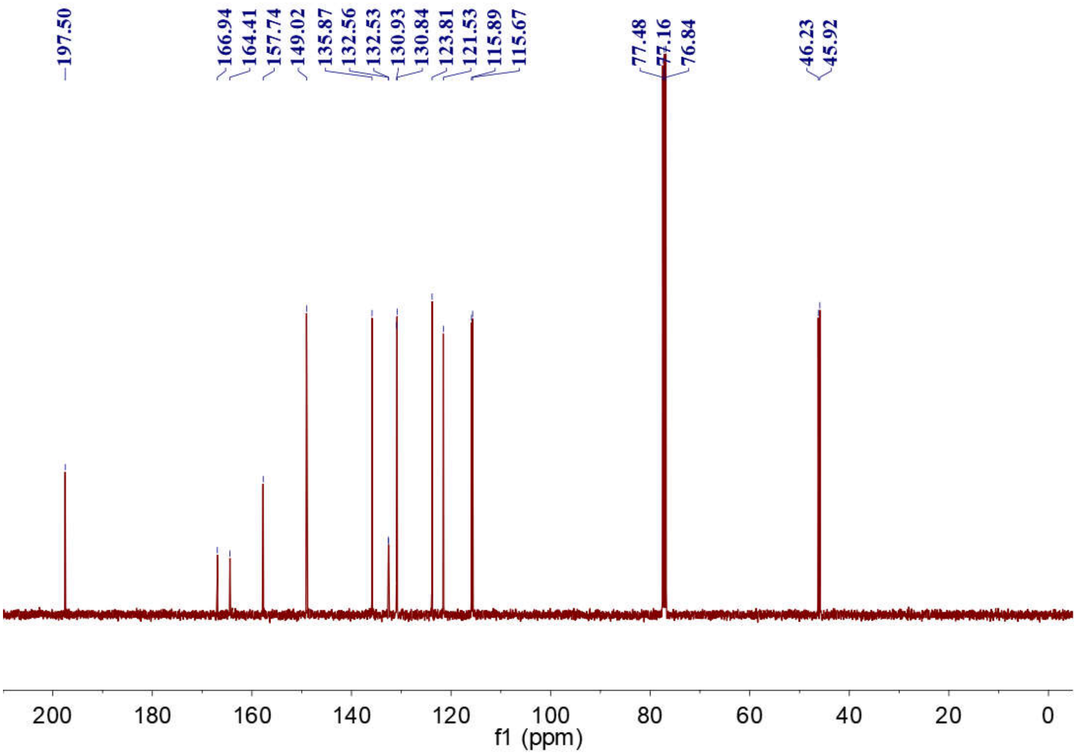

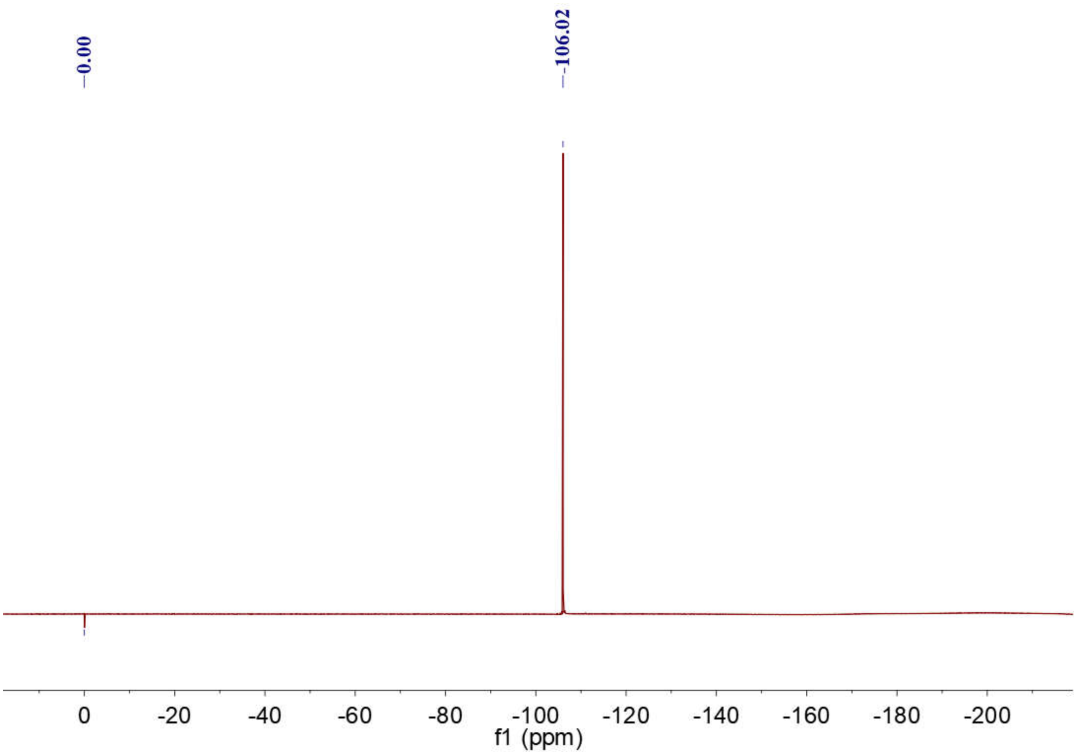

0.00
-106.02
0
-20
-40
-60
-80
-100
-120
-140
-160
-180
-200
f1 (ppm)

NMR spectra of product 29

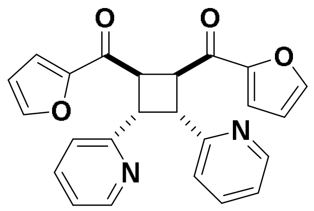

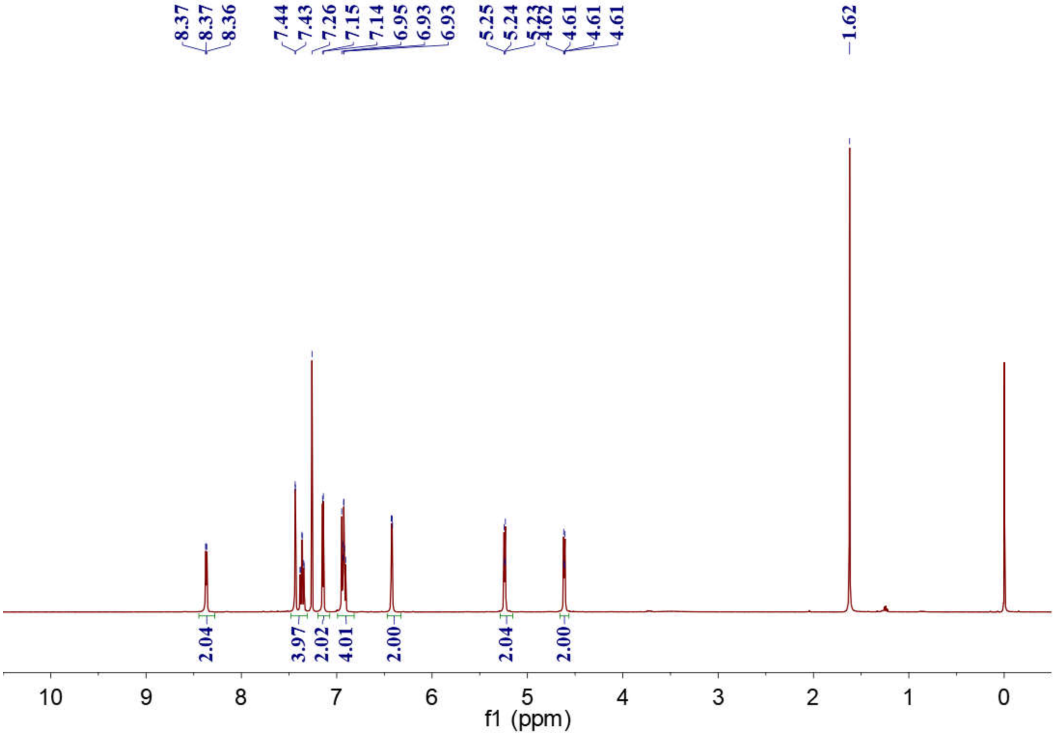

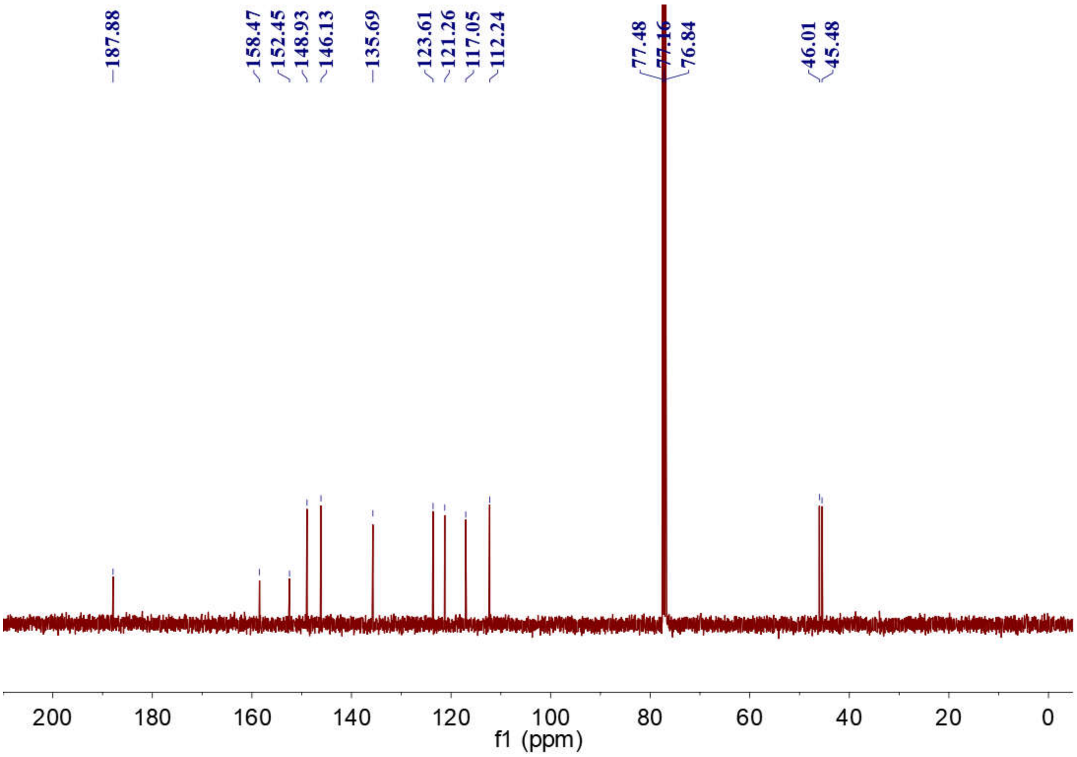

NMR spectra of product 30

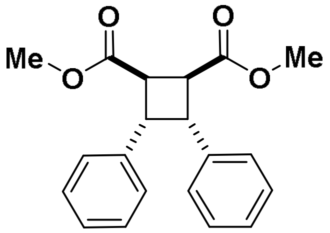

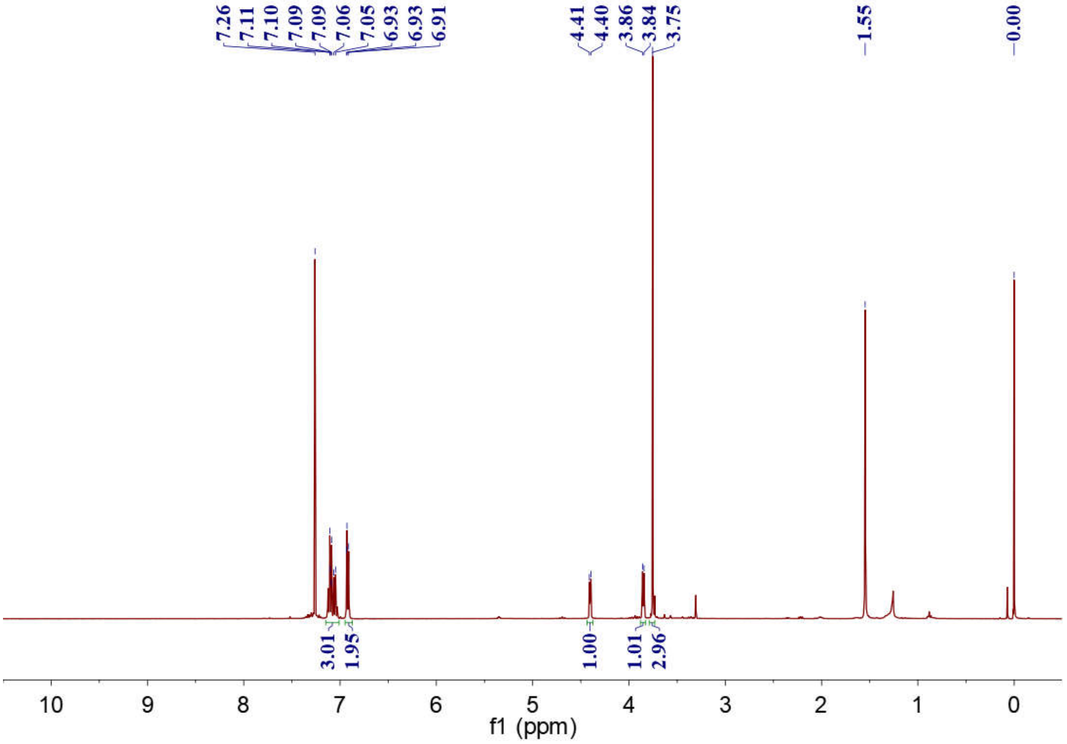

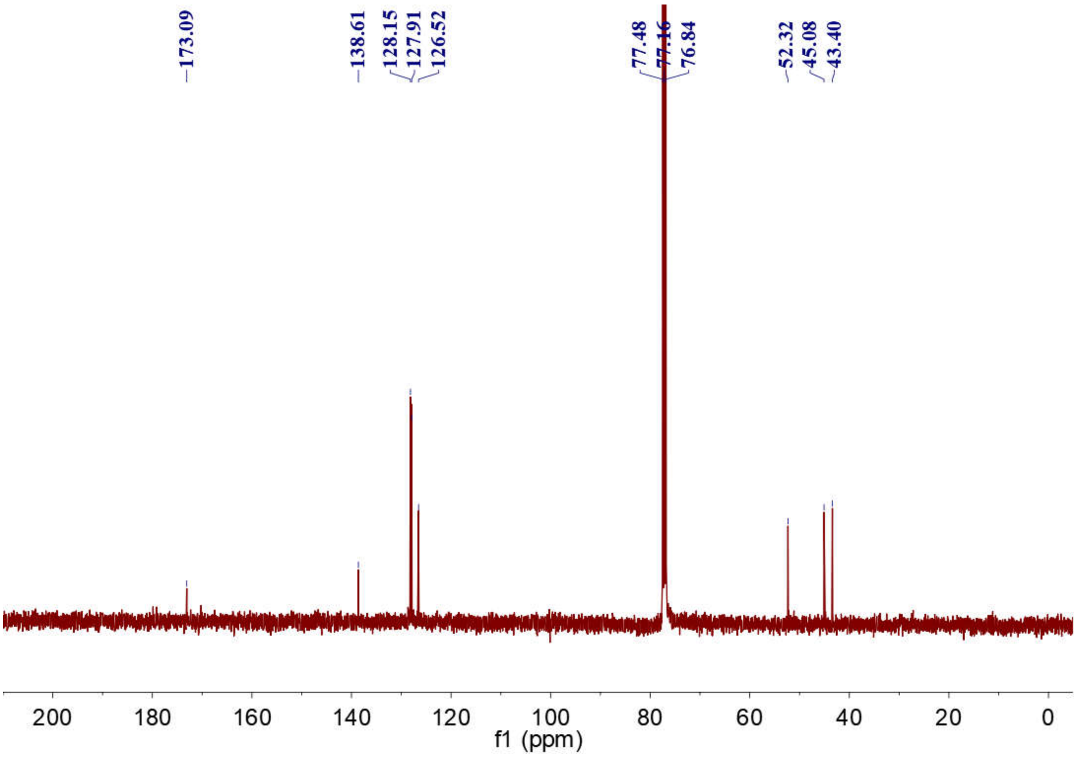

NMR spectra of product 31

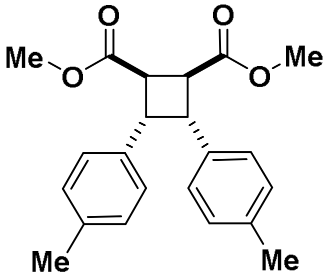

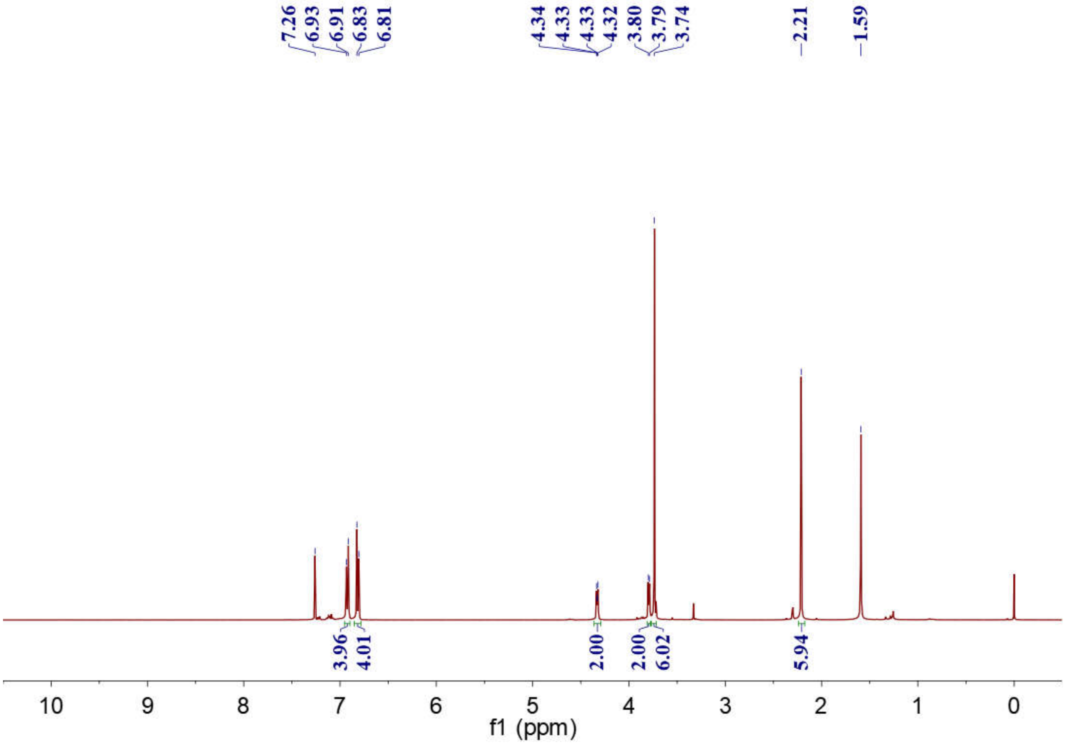

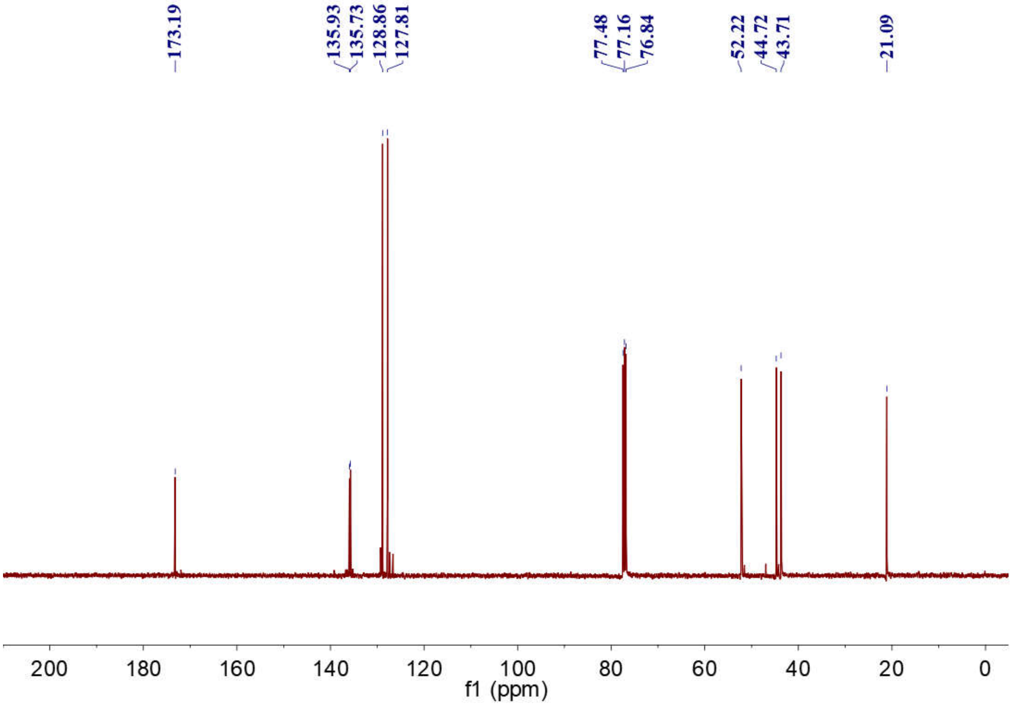

NMR spectra of product 32

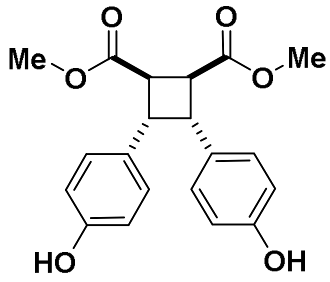

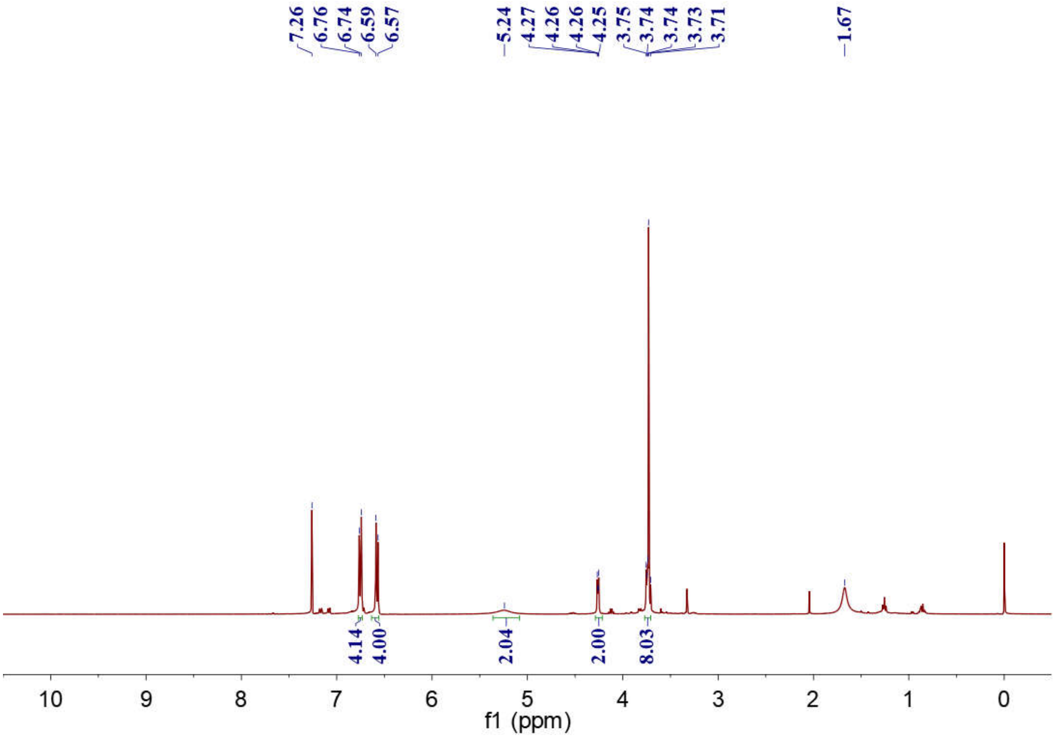

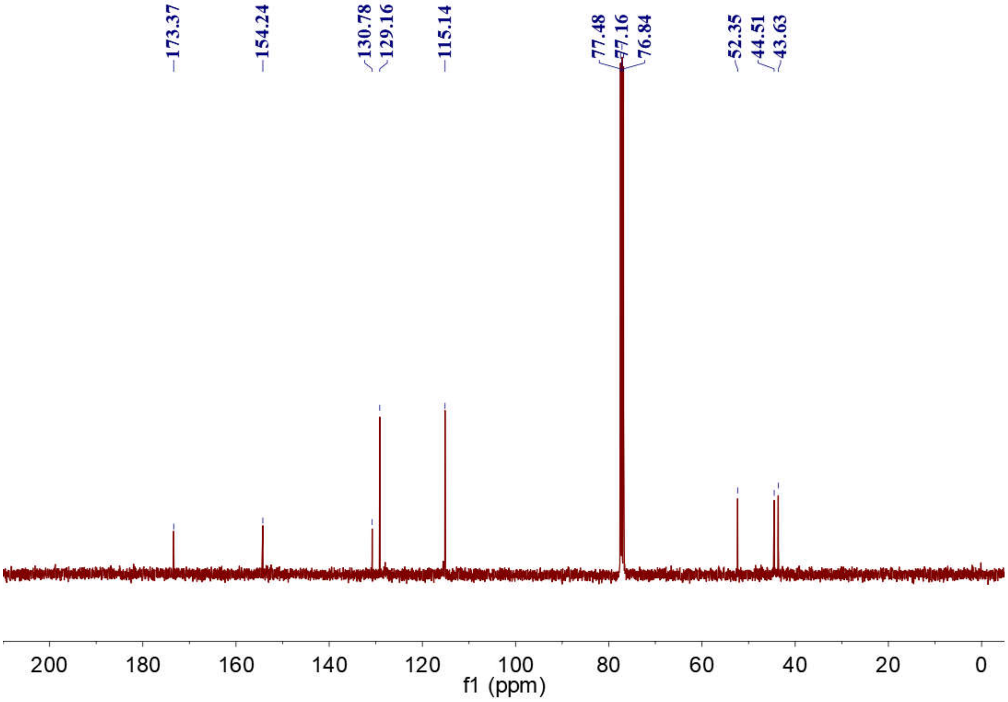

NMR spectra of product 33

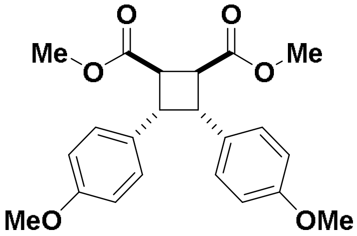

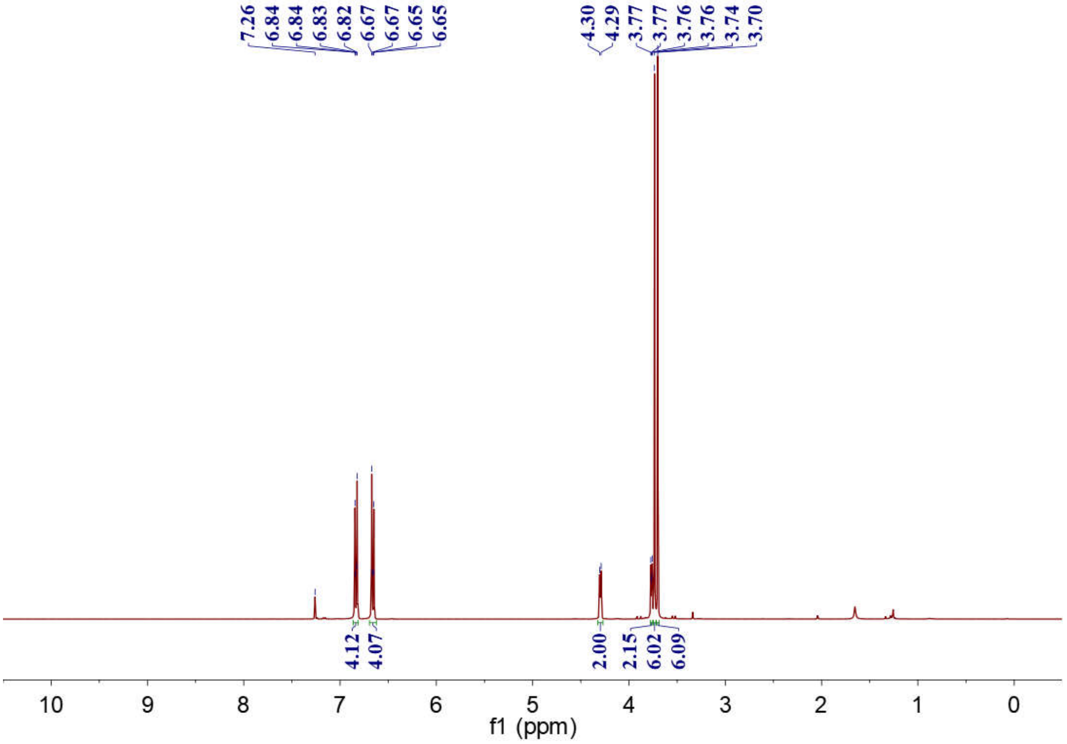

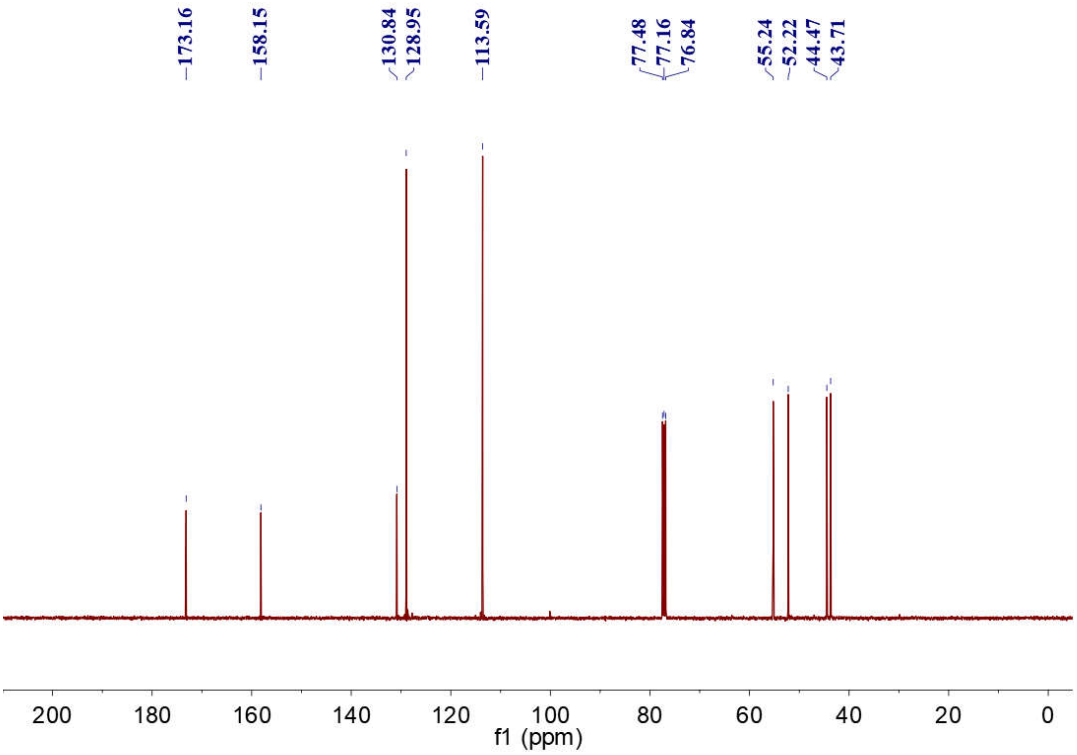

NMR spectra of product 34

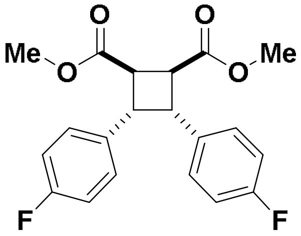

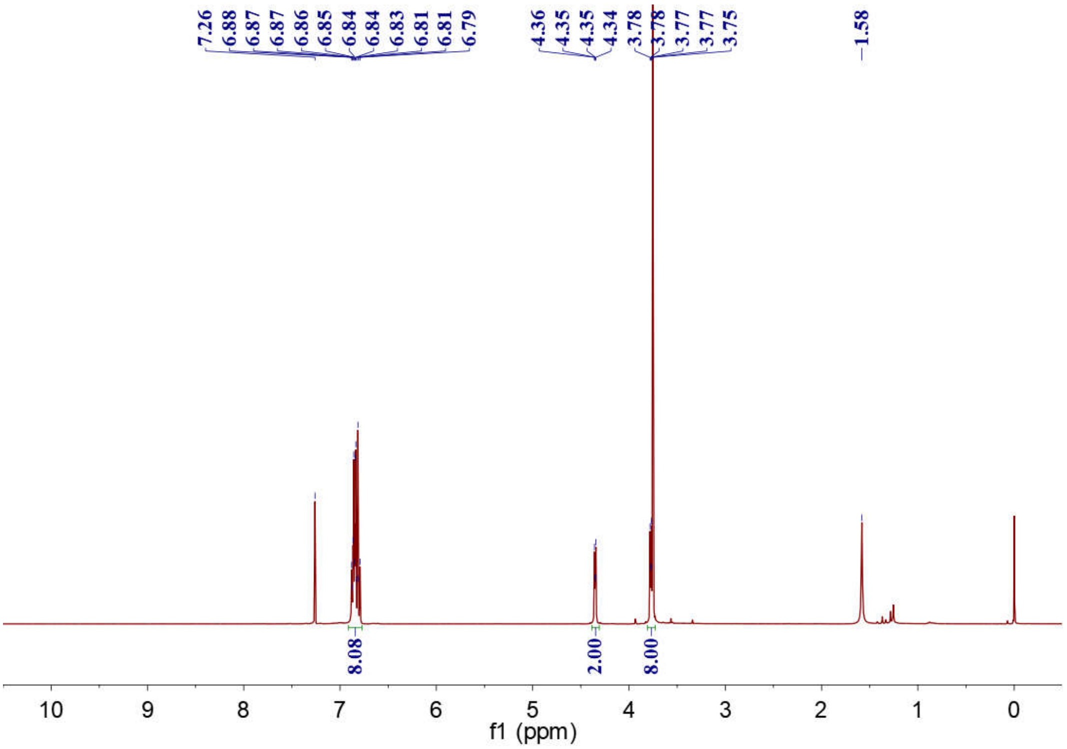

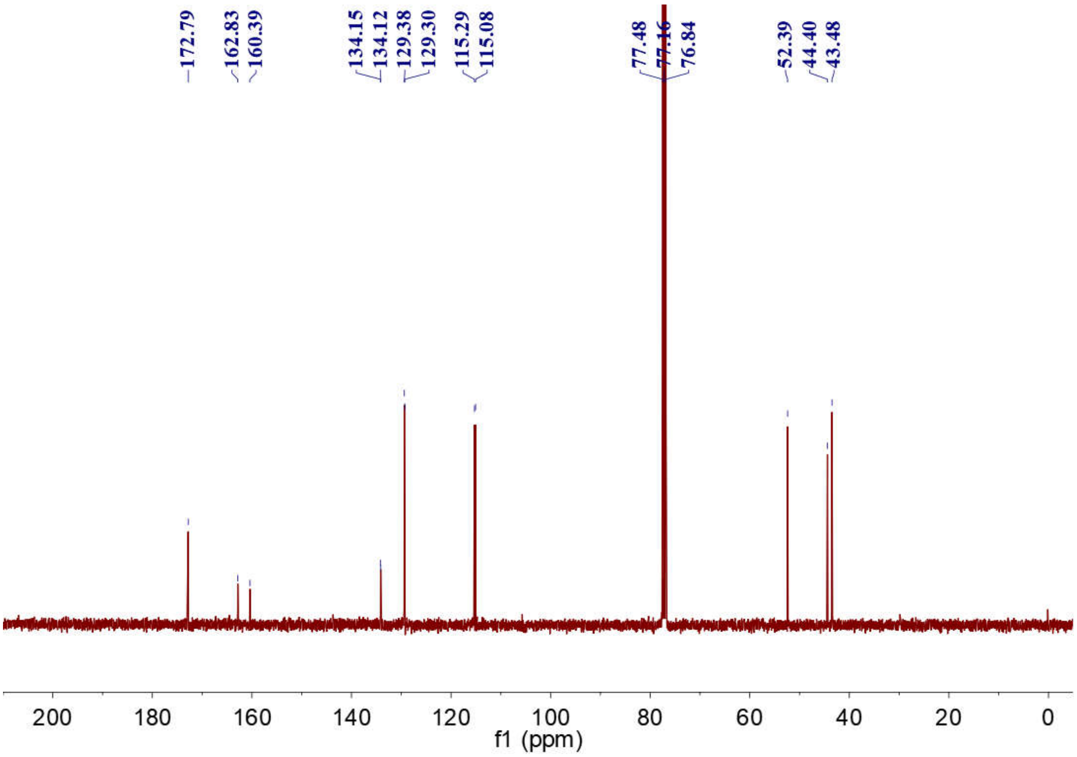

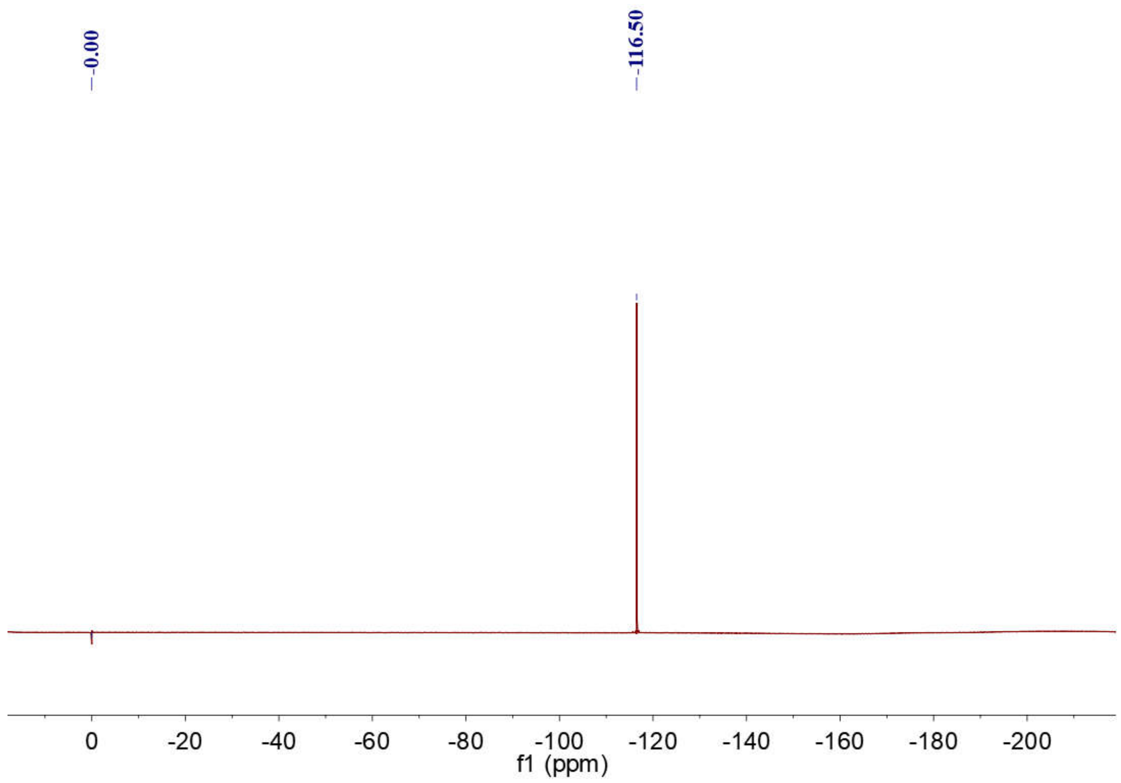

-0.00
-116.50
0
-20
-40
-60
-80
-100
-120
-140
-160
-180
-200
f1 (ppm)

NMR spectra of product 35

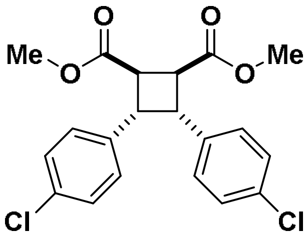

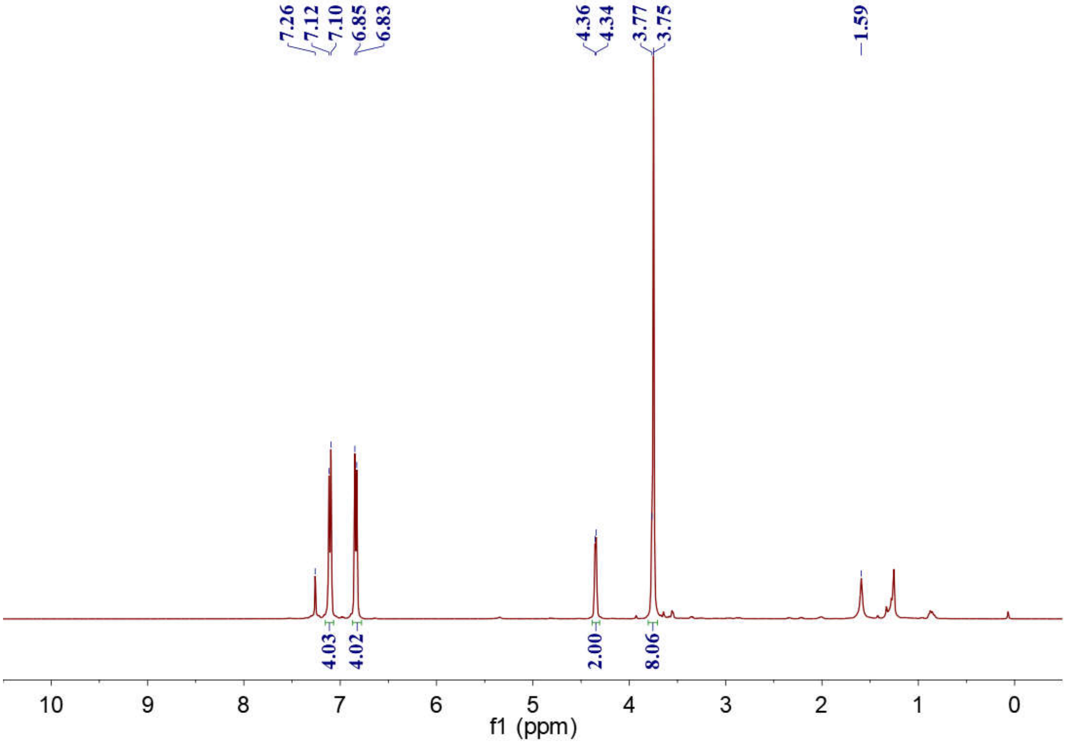

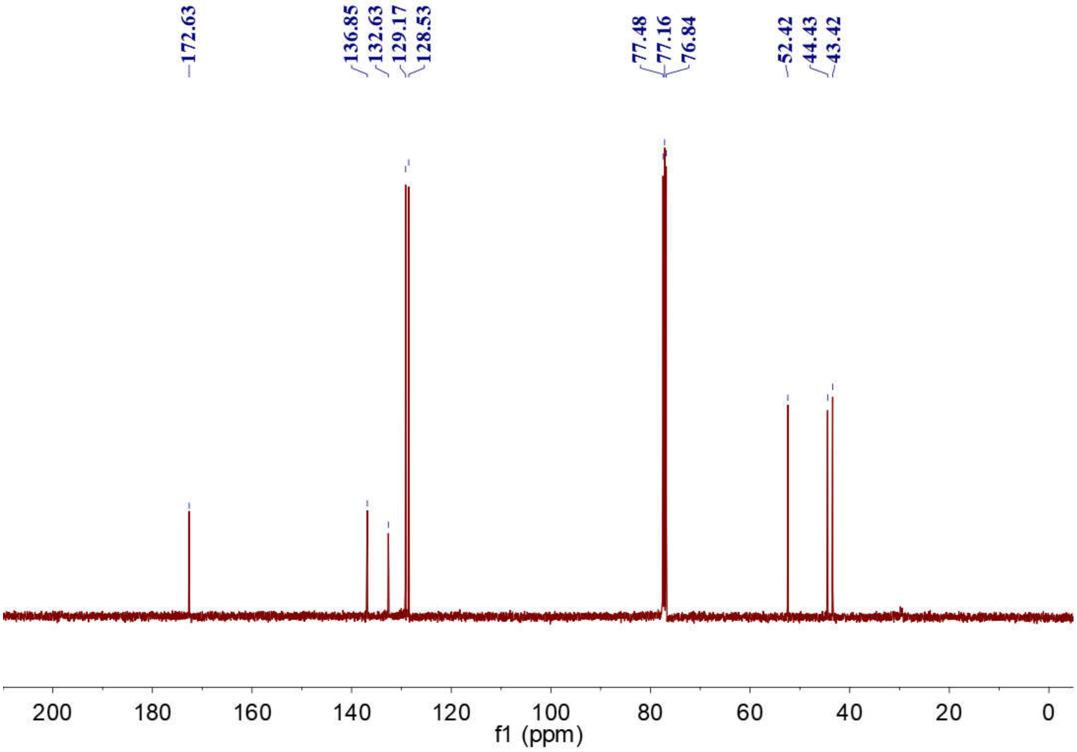

NMR spectra of product 36

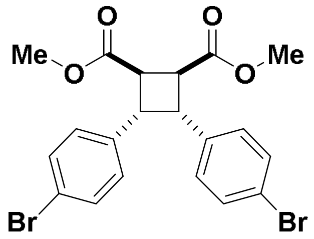

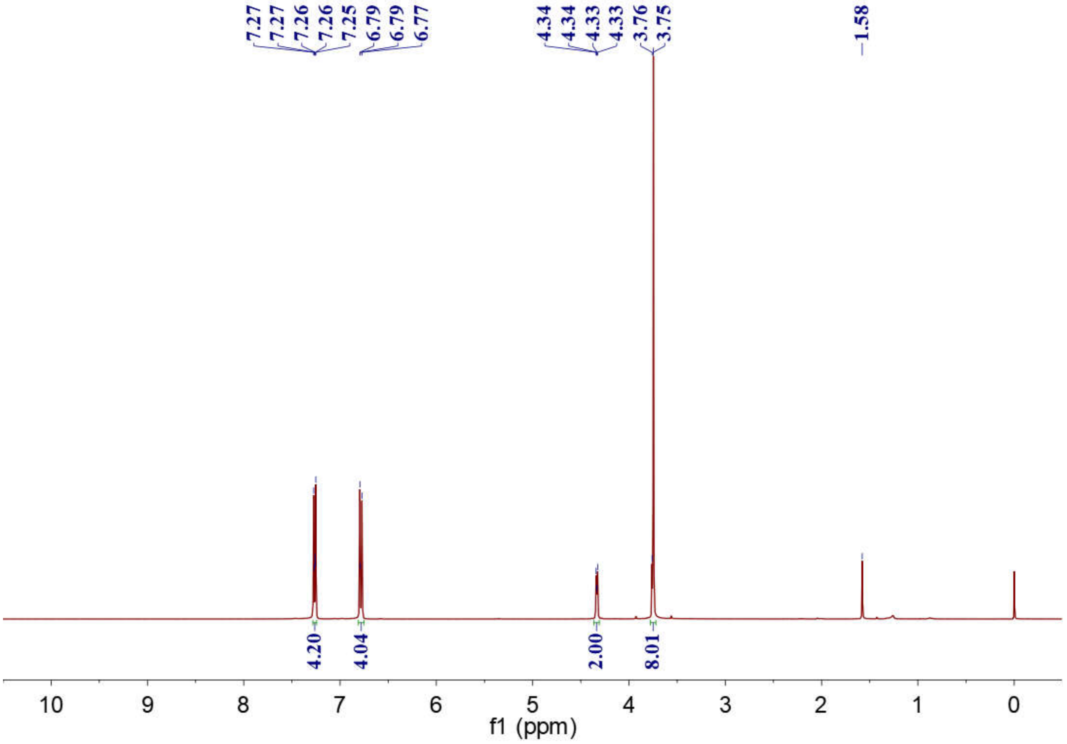

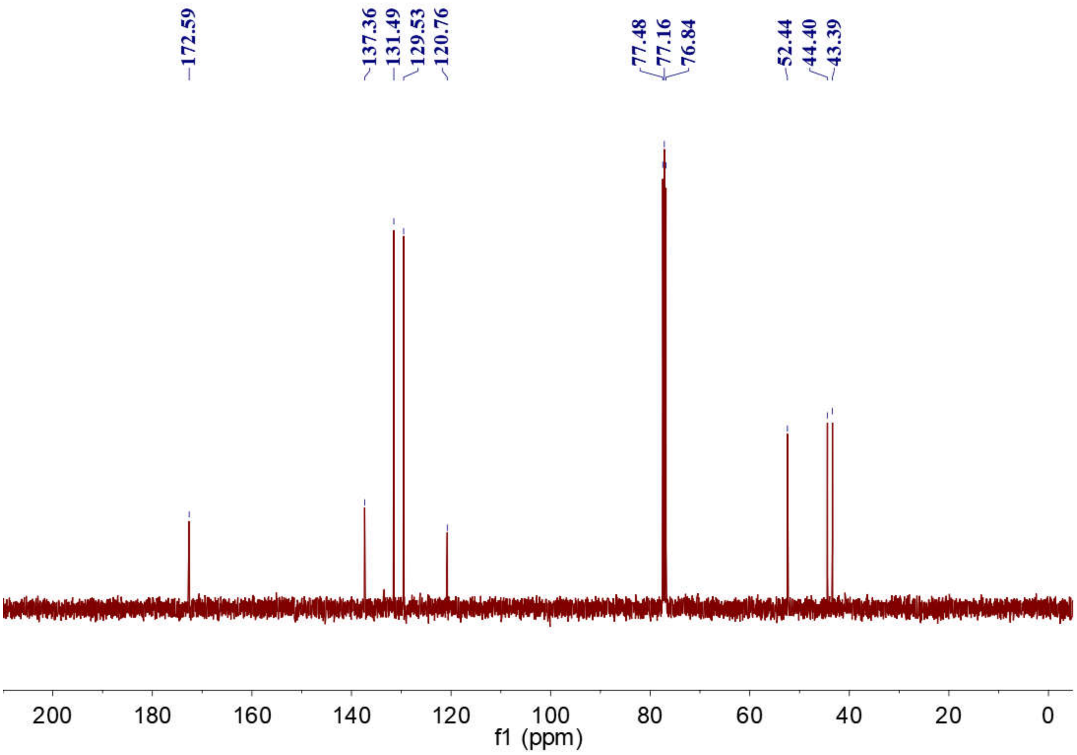

NMR spectra of product 37

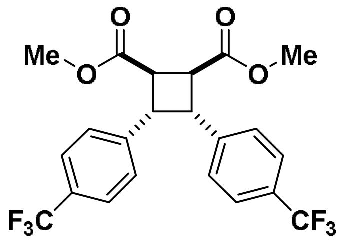

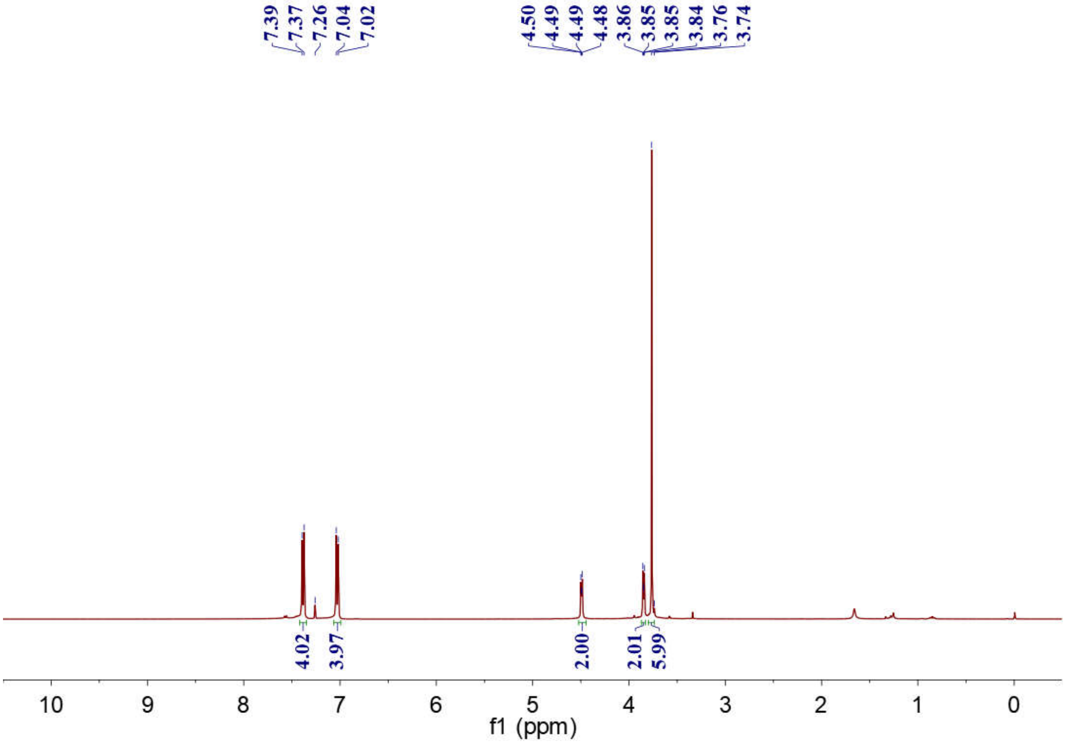

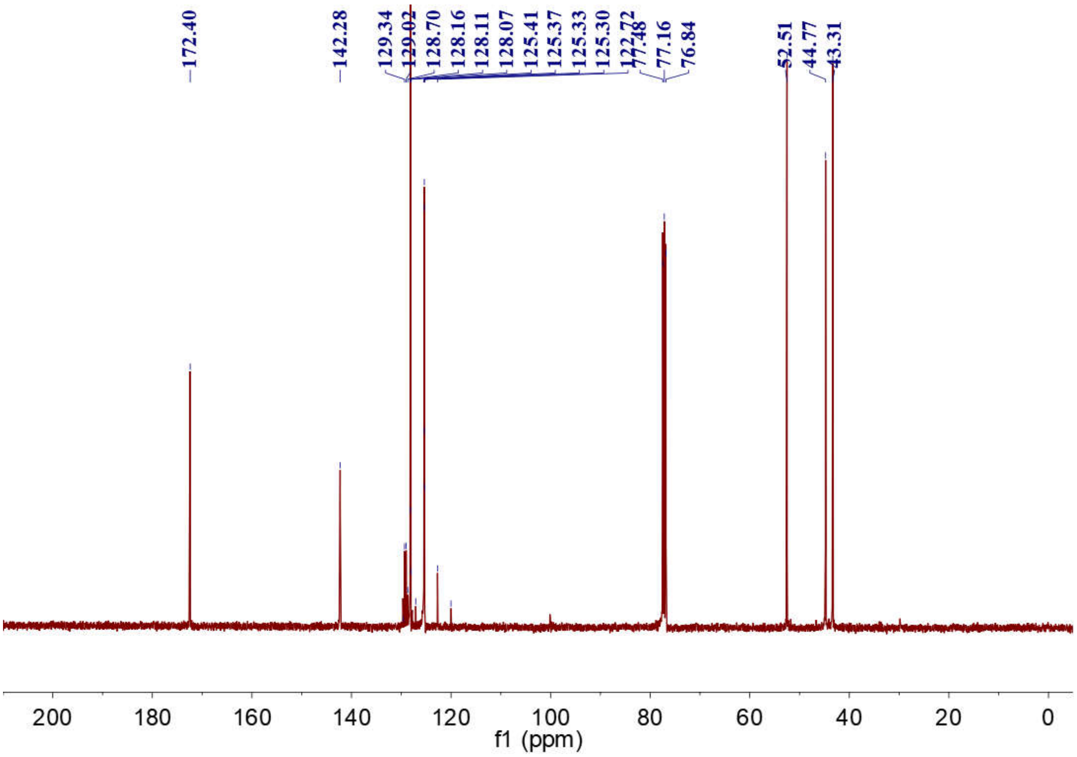

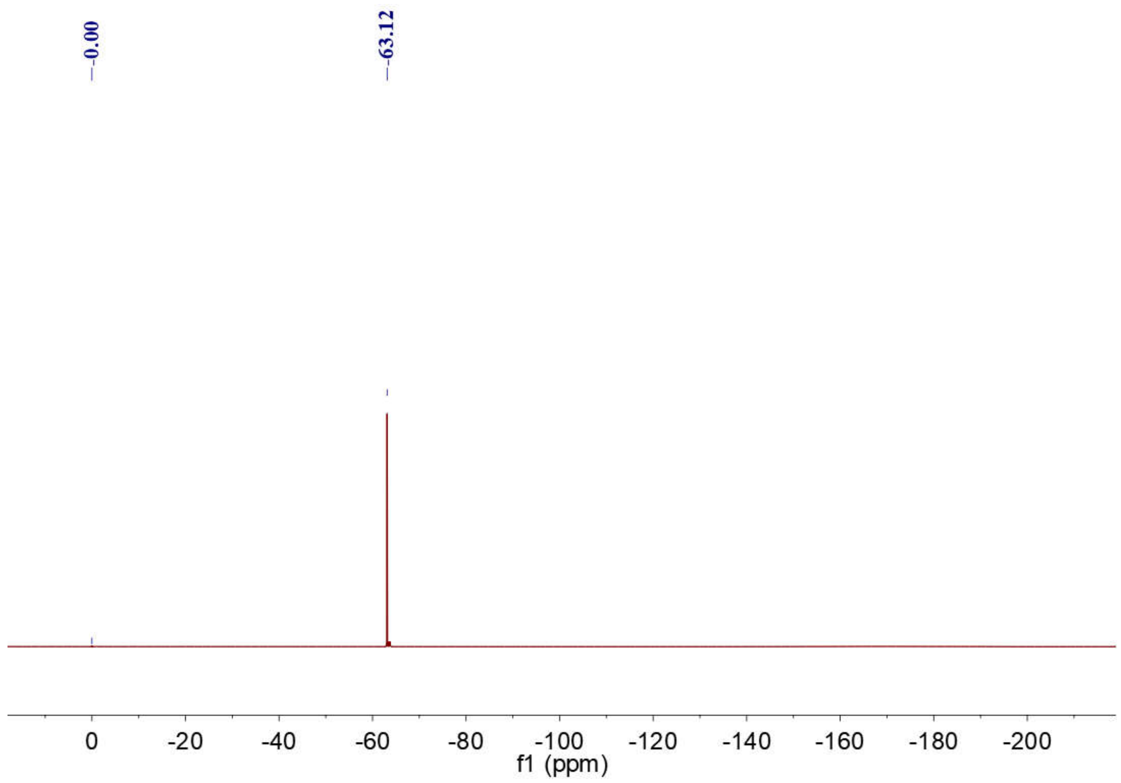
-0.00
-63.12
0
-20
-40
-60
-80
-100
-120
-140
-160
-180
-200
f1 (ppm)

NMR spectra of product 38

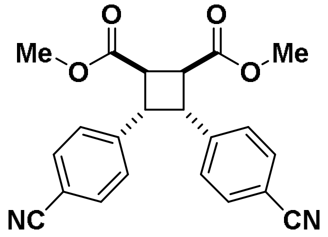

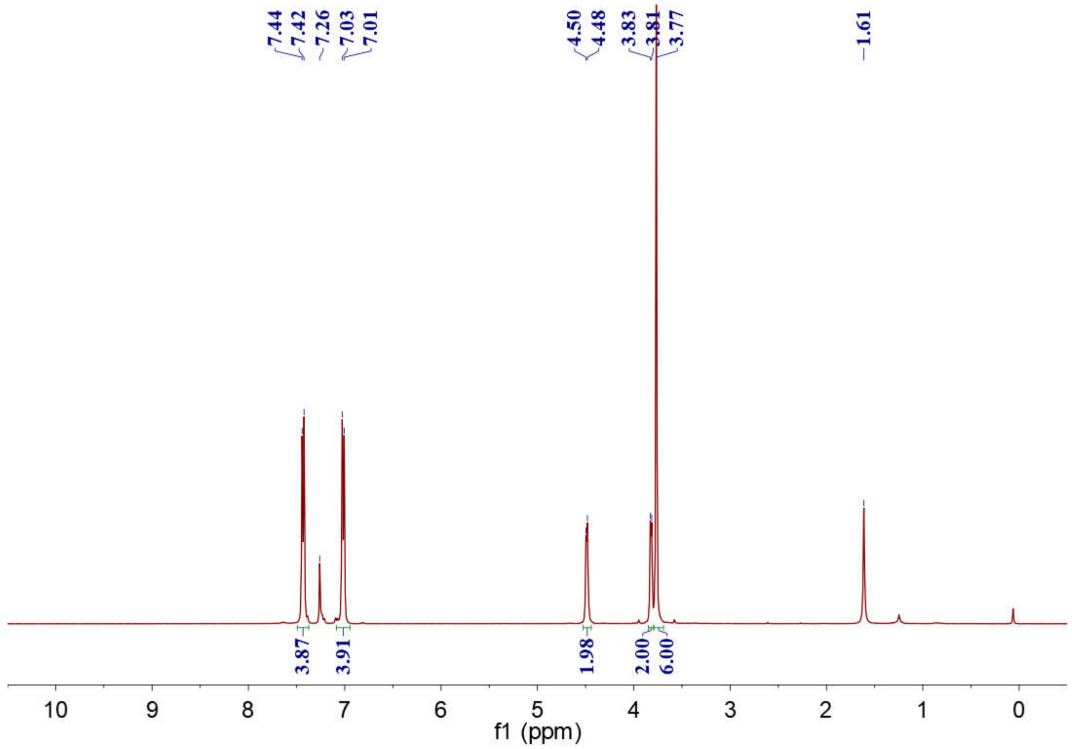

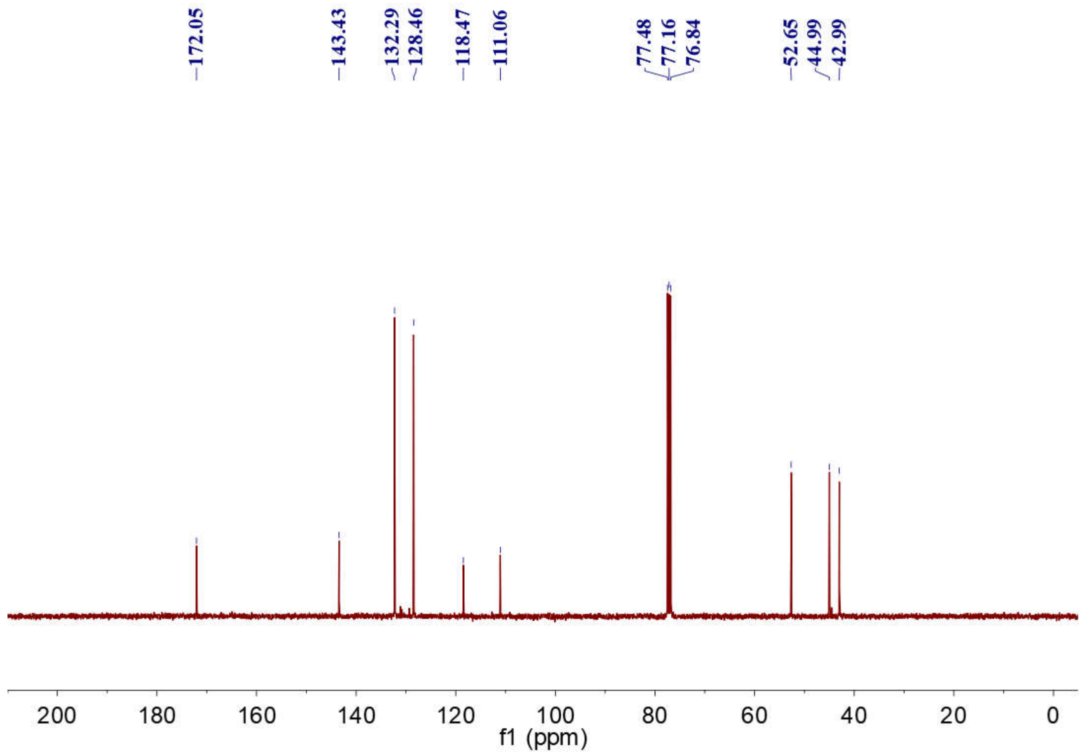

NMR spectra of product 39

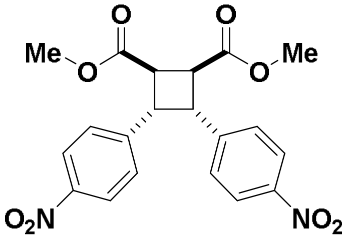

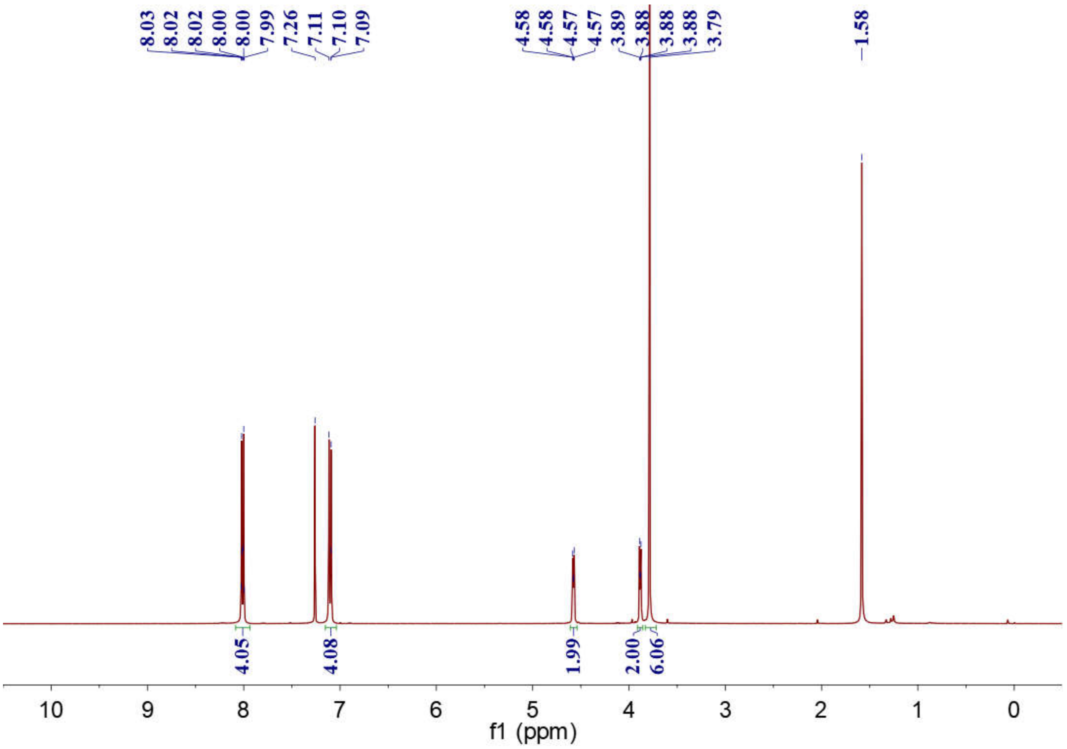

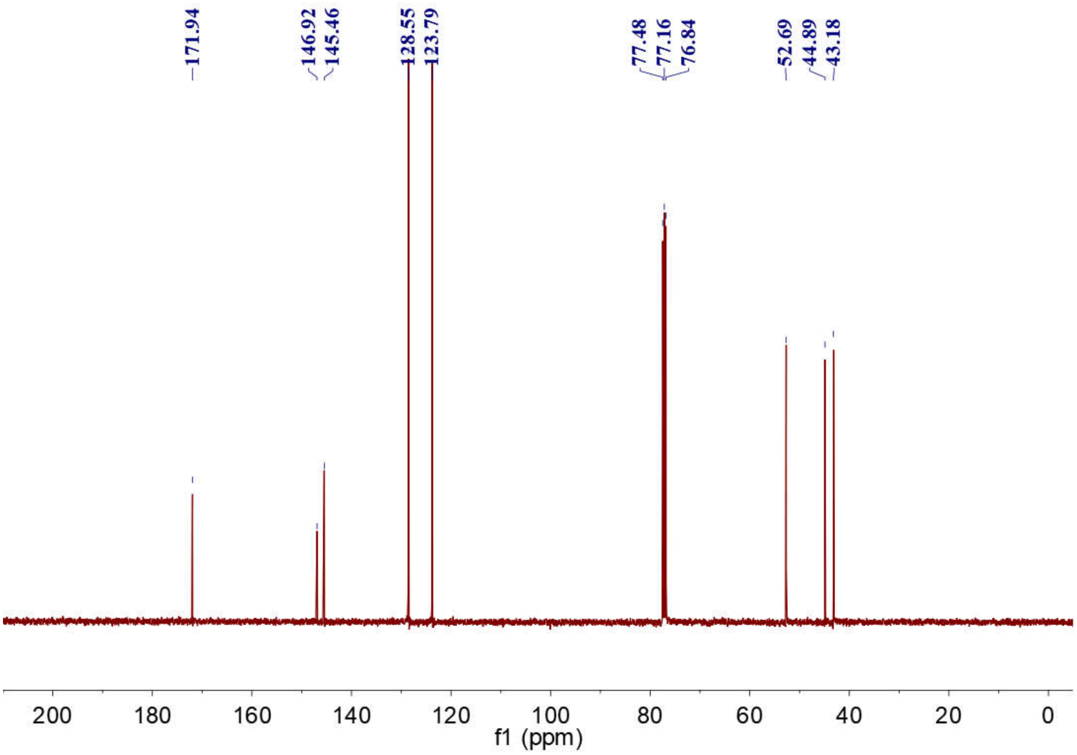

NMR spectra of product 40

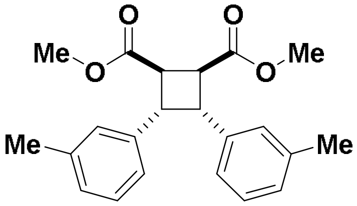

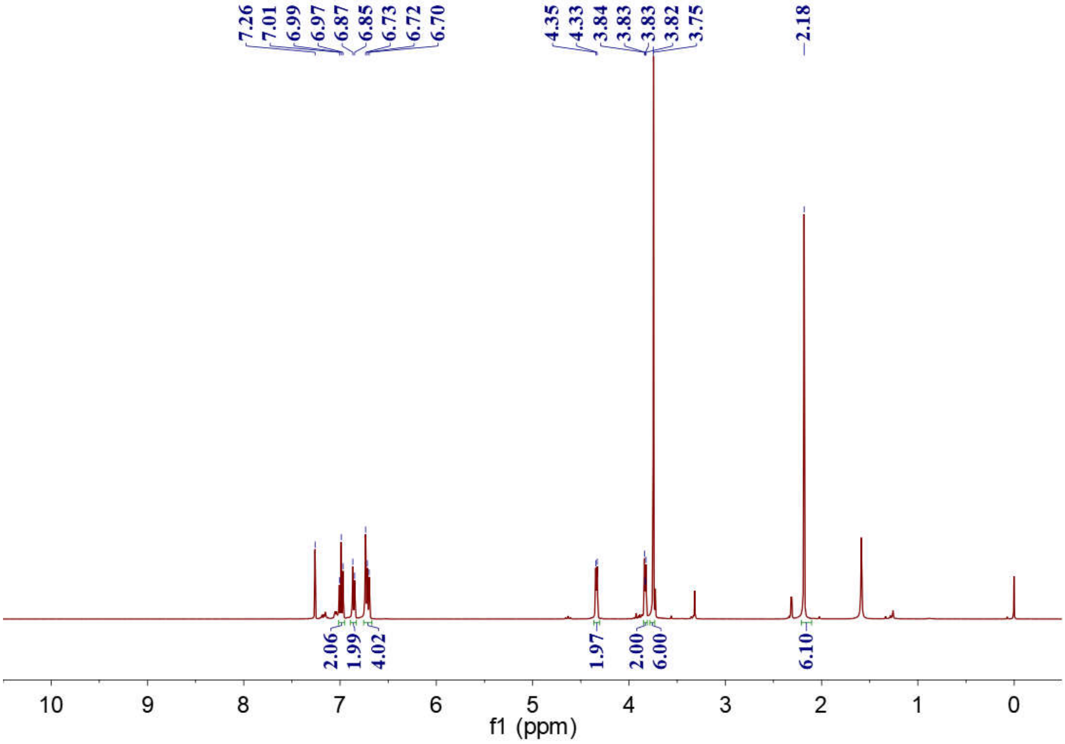

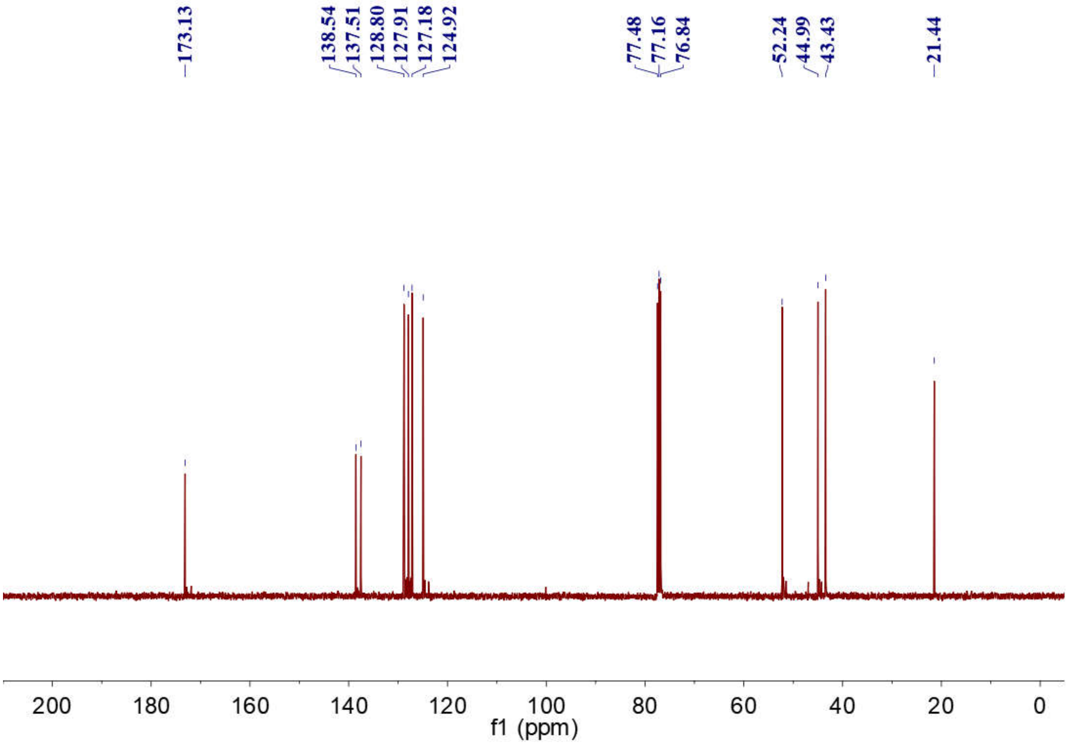

NMR spectra of product 41

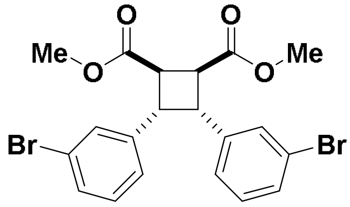

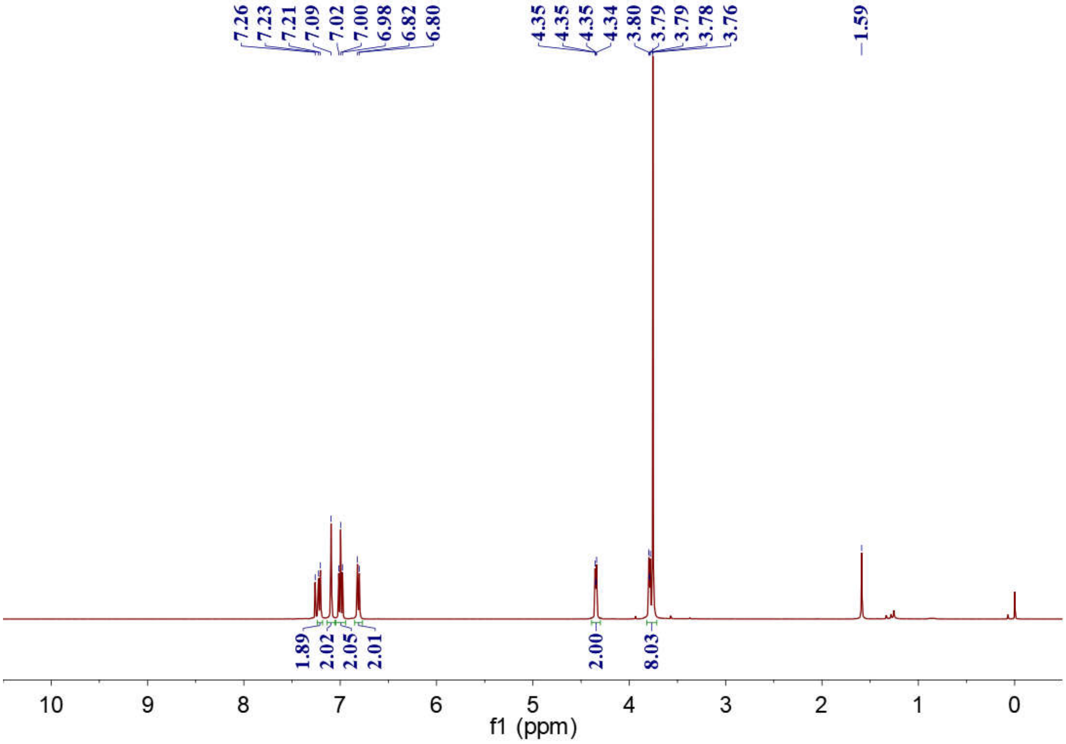

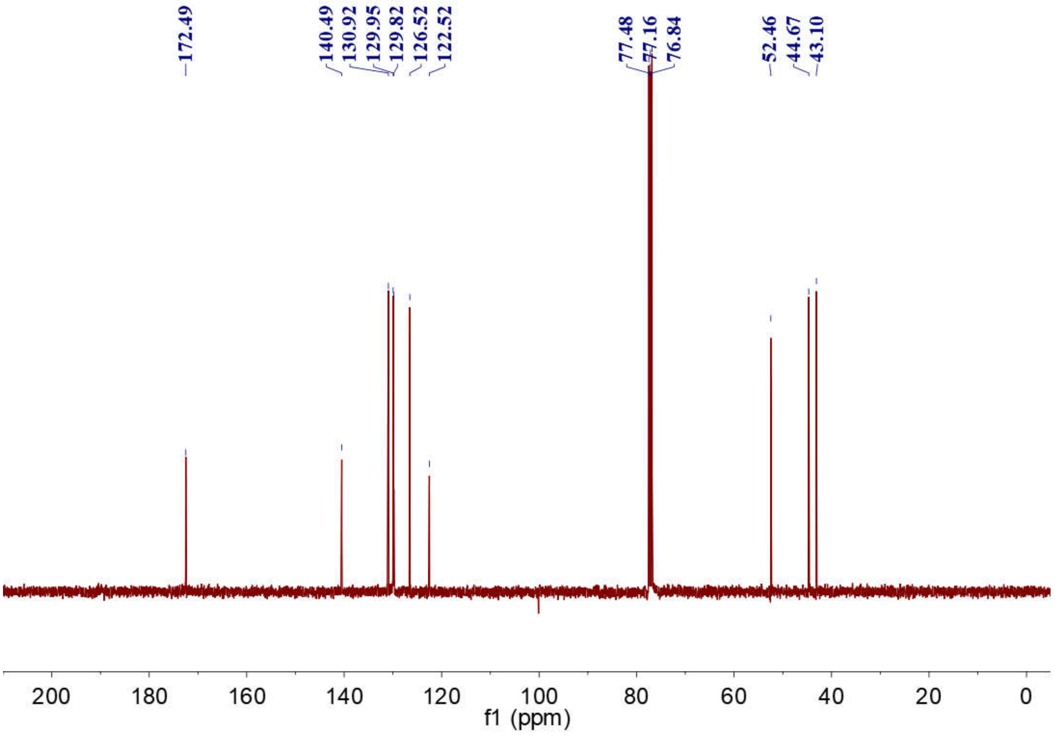

NMR spectra of product 42

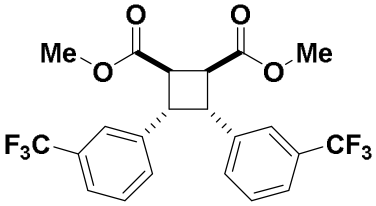

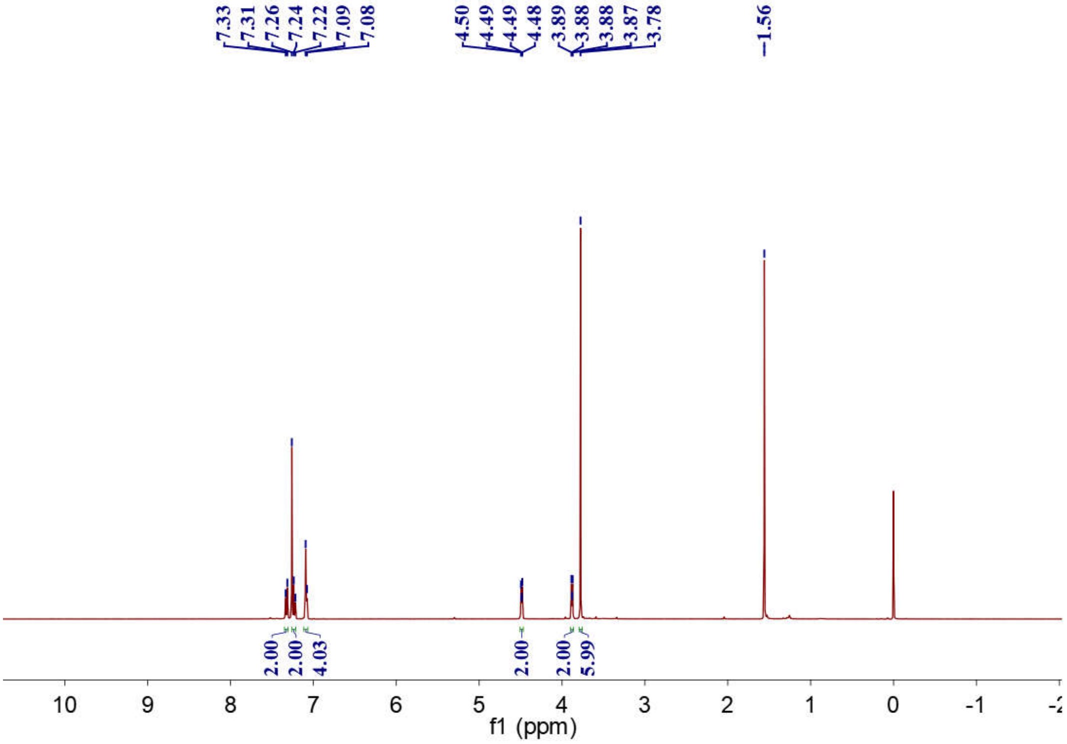

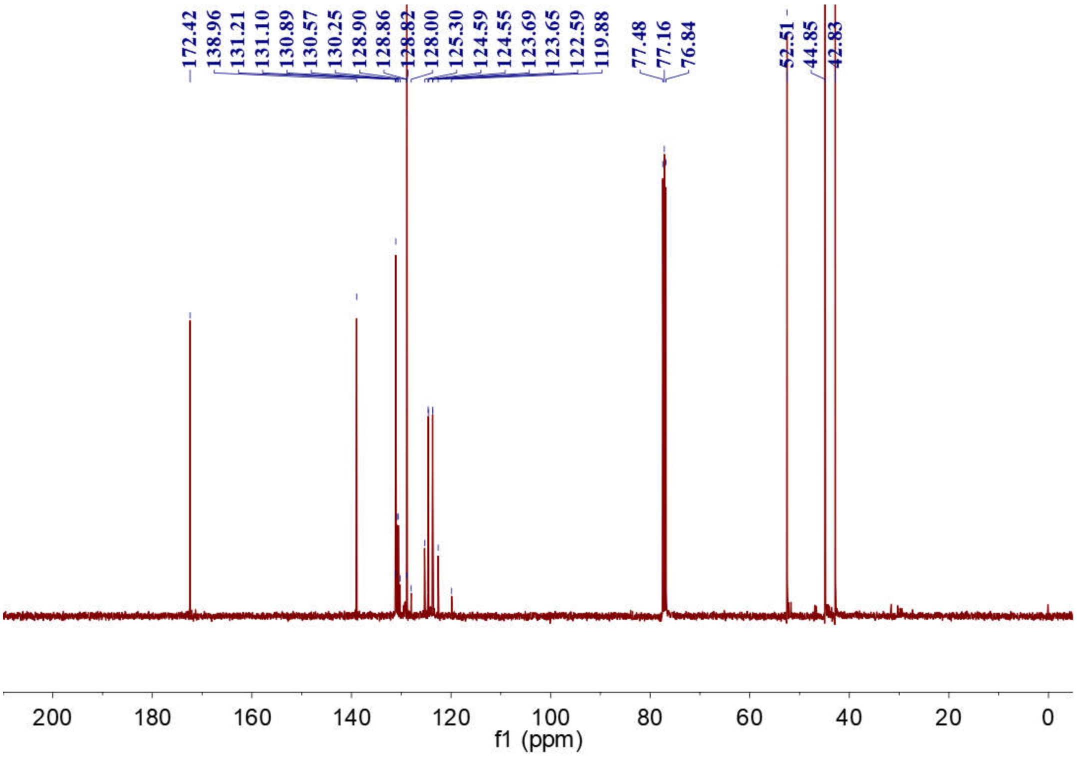

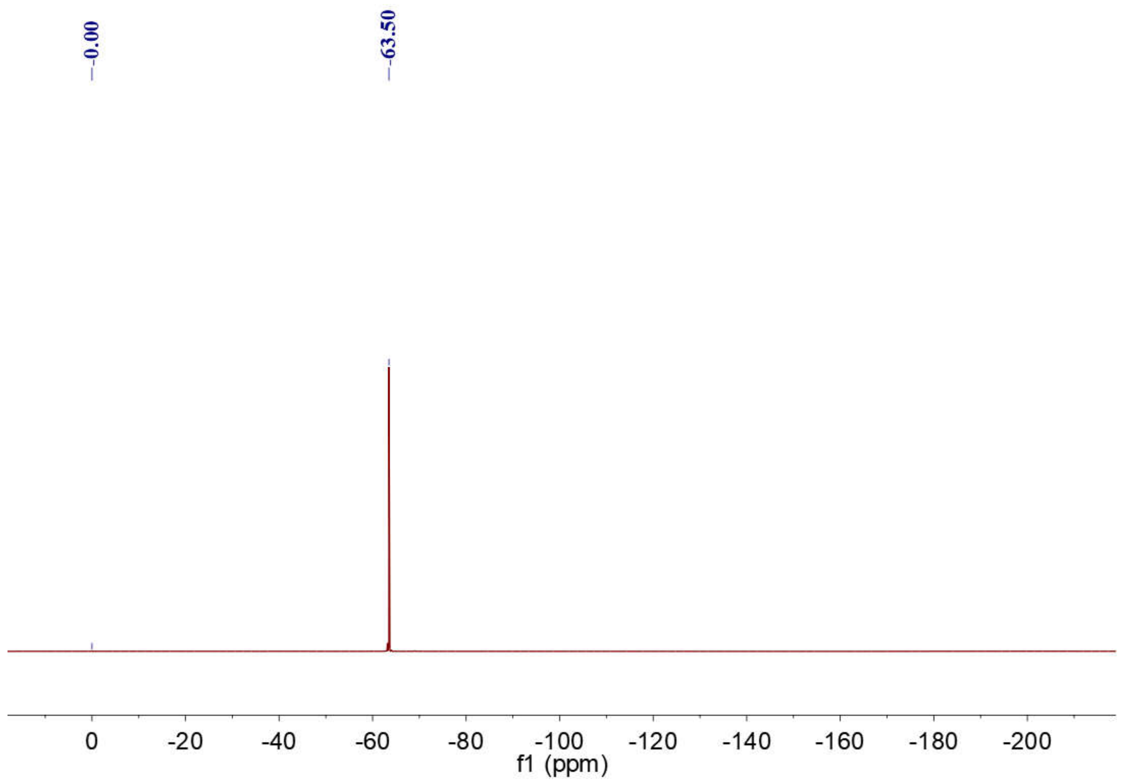

-0.00
-63.50
0
-20
-40
-60
-80
-100
-120
-140
-160
-180
-200
f1 (ppm)

NMR spectra of product 43

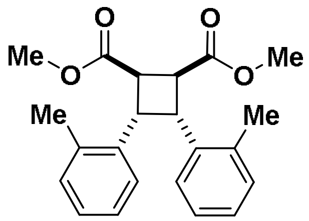

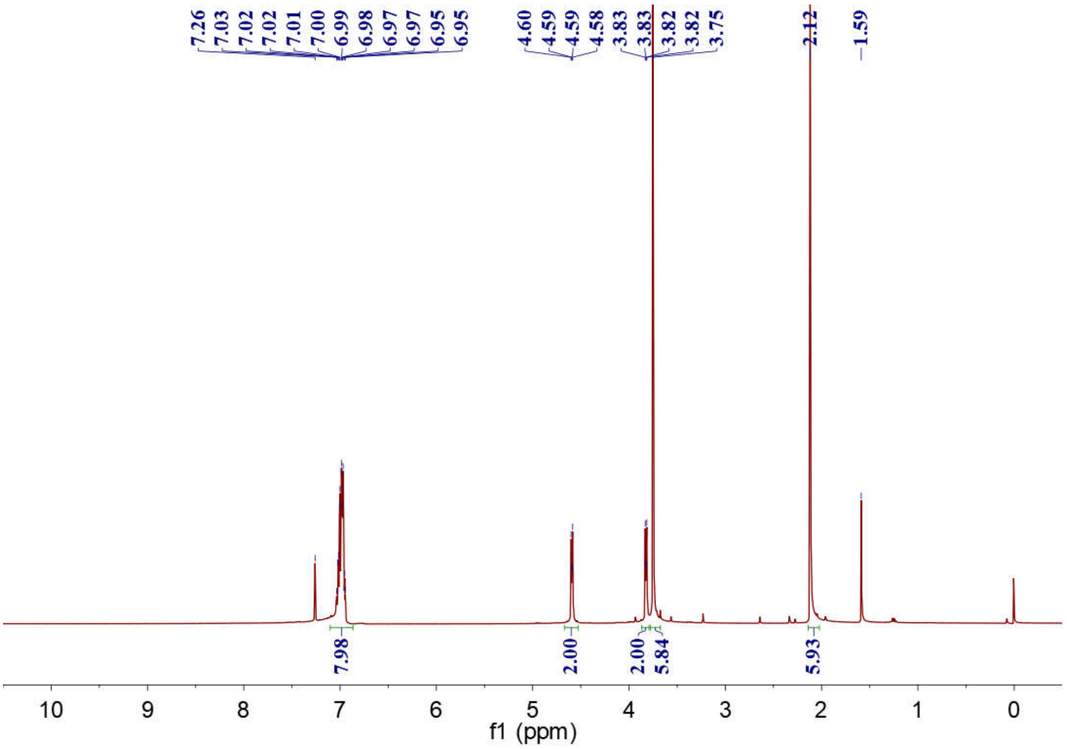

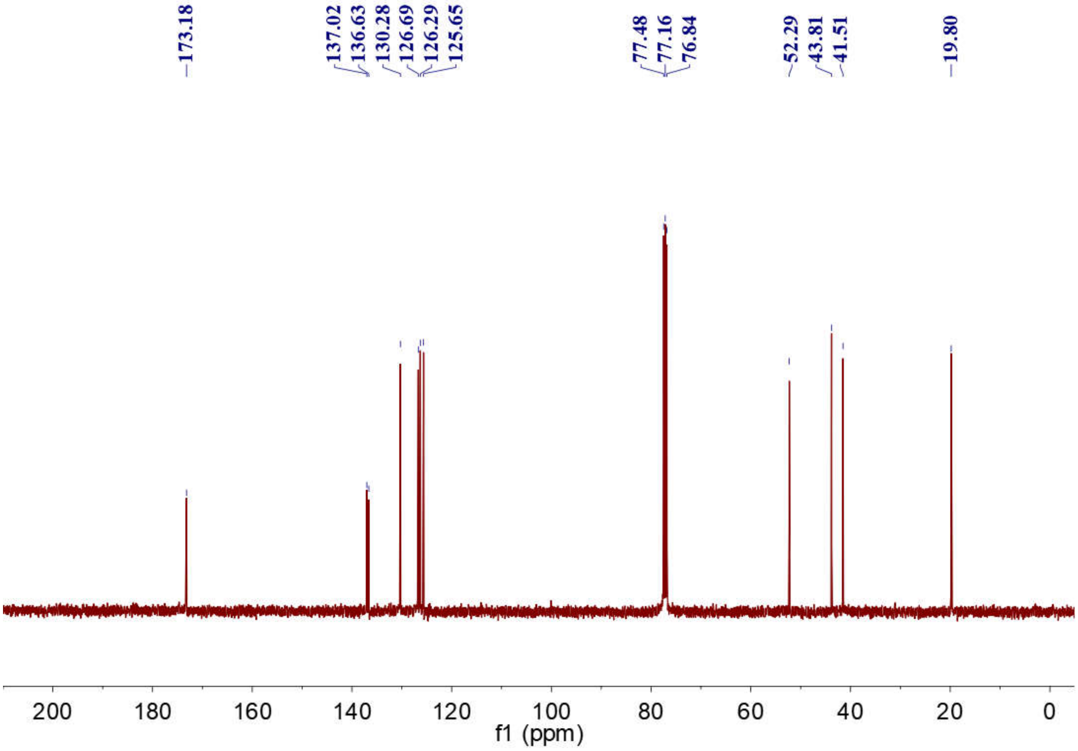

NMR spectra of product 44

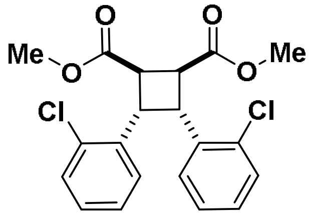

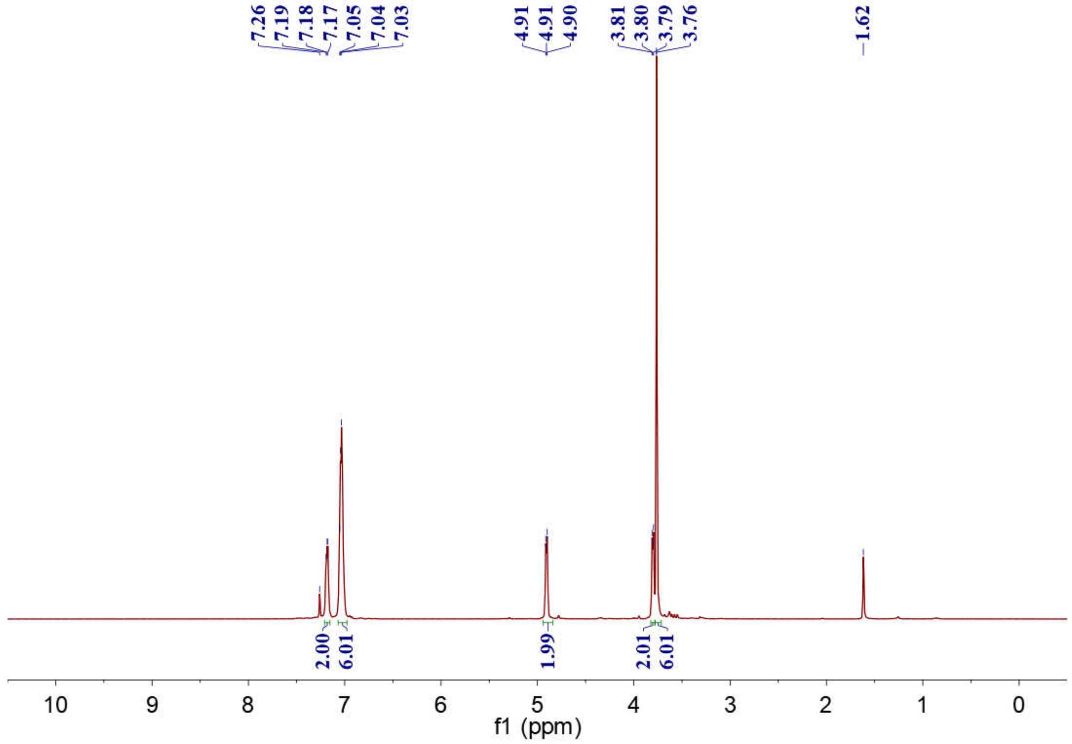

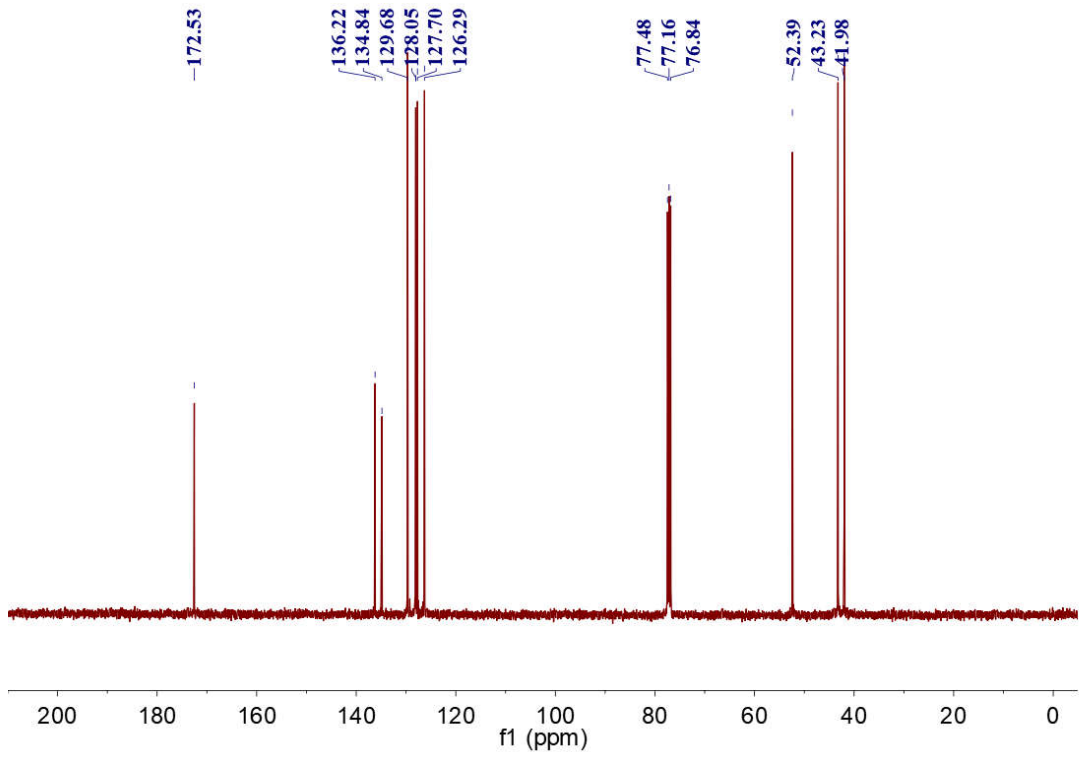

NMR spectra of product 45

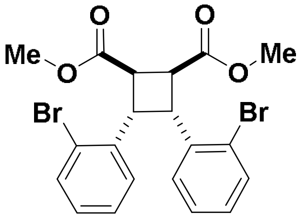

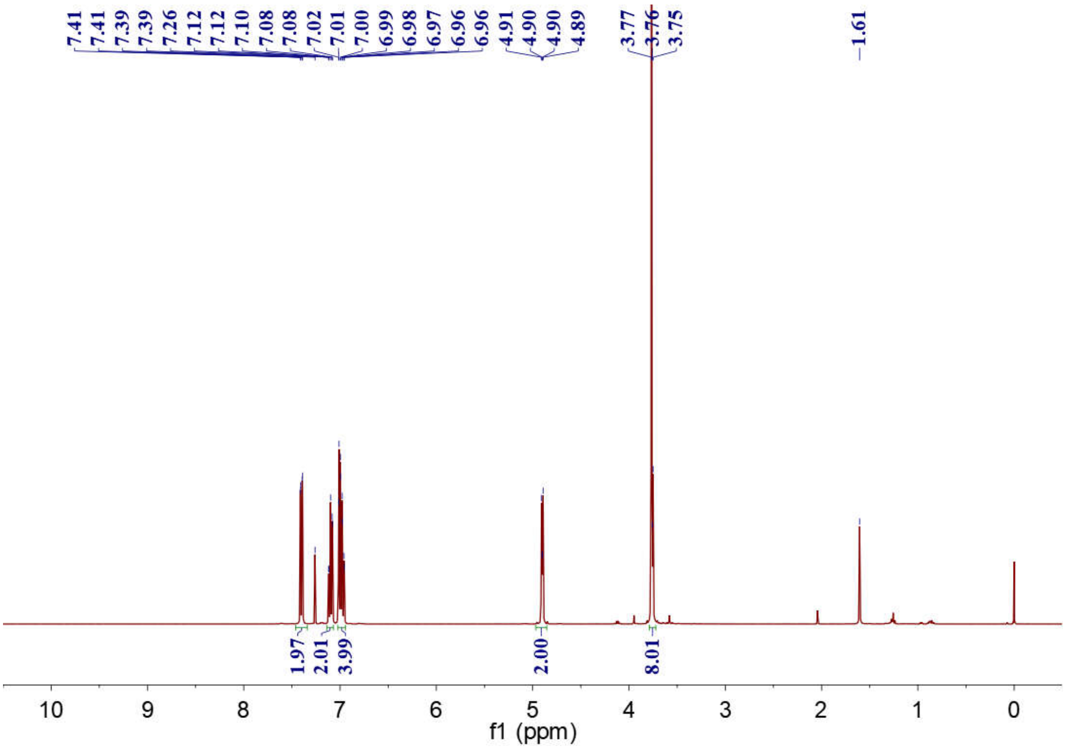

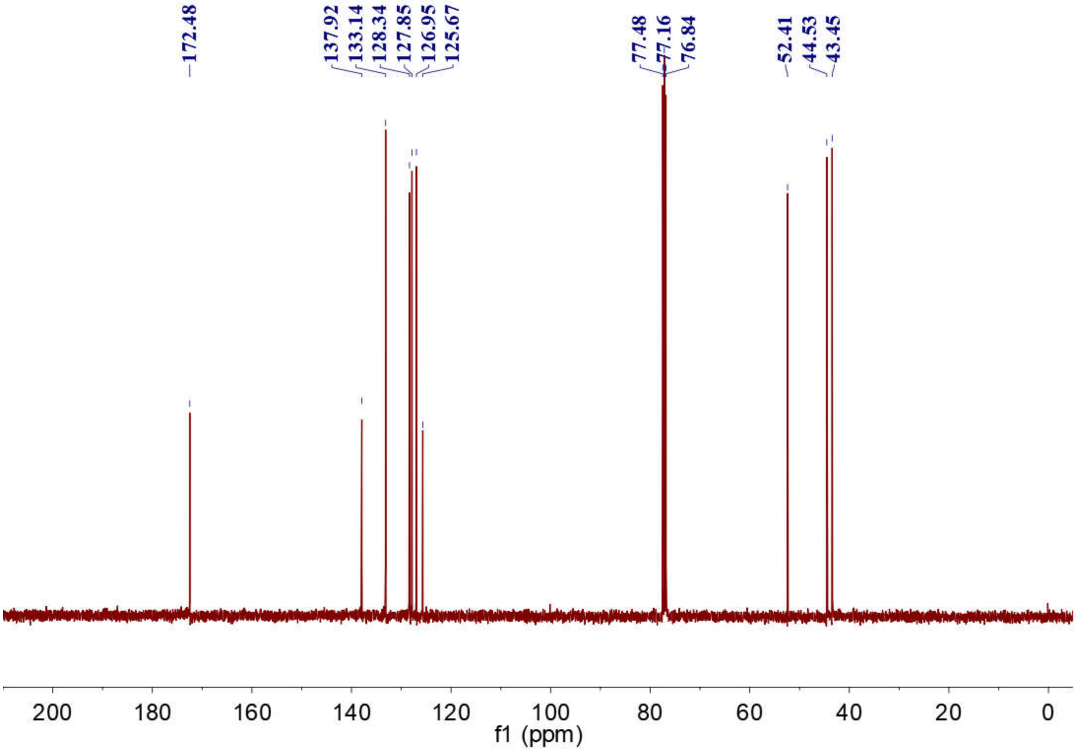

NMR spectra of product 46

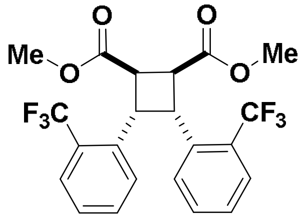

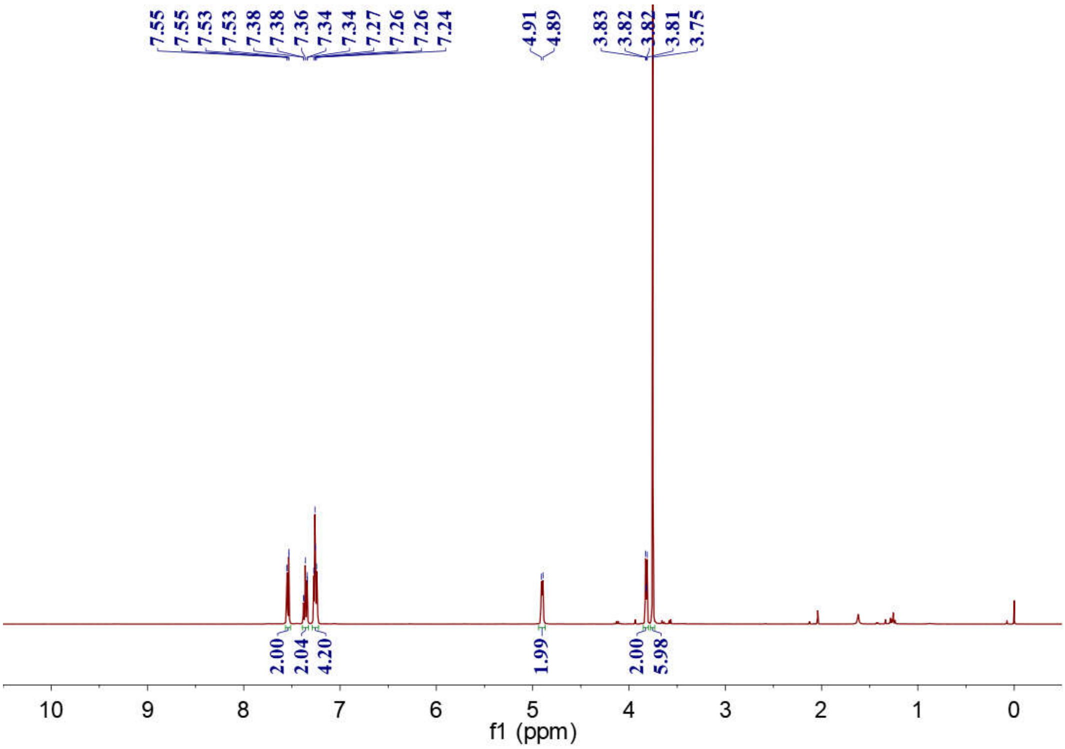

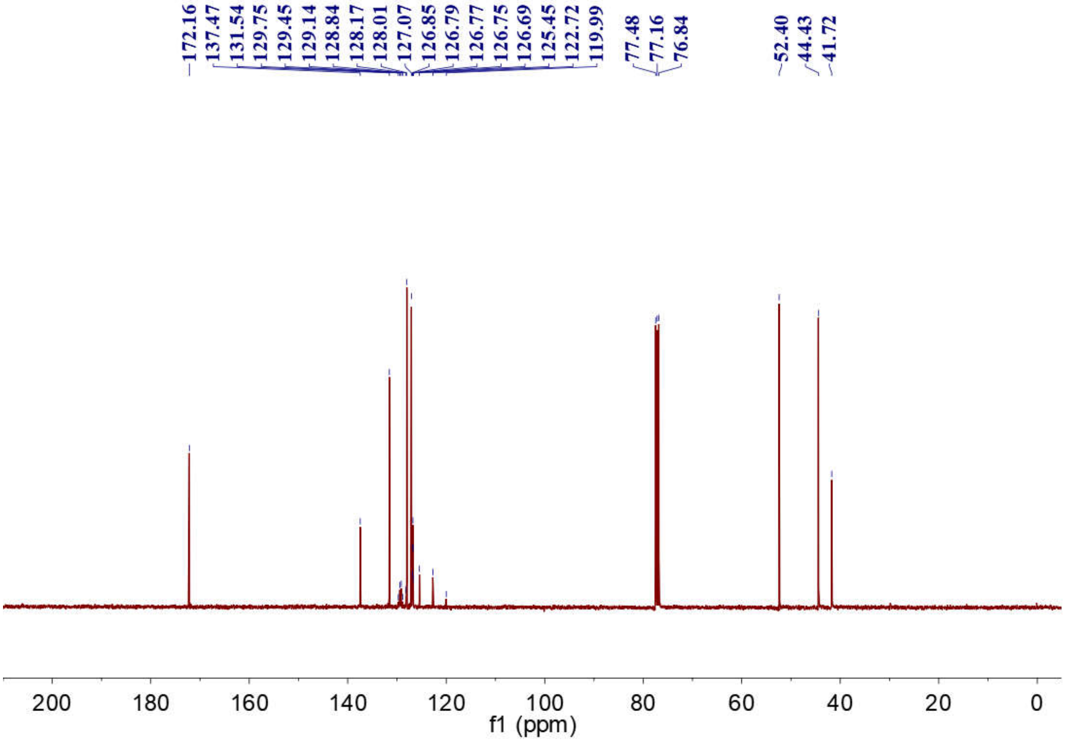

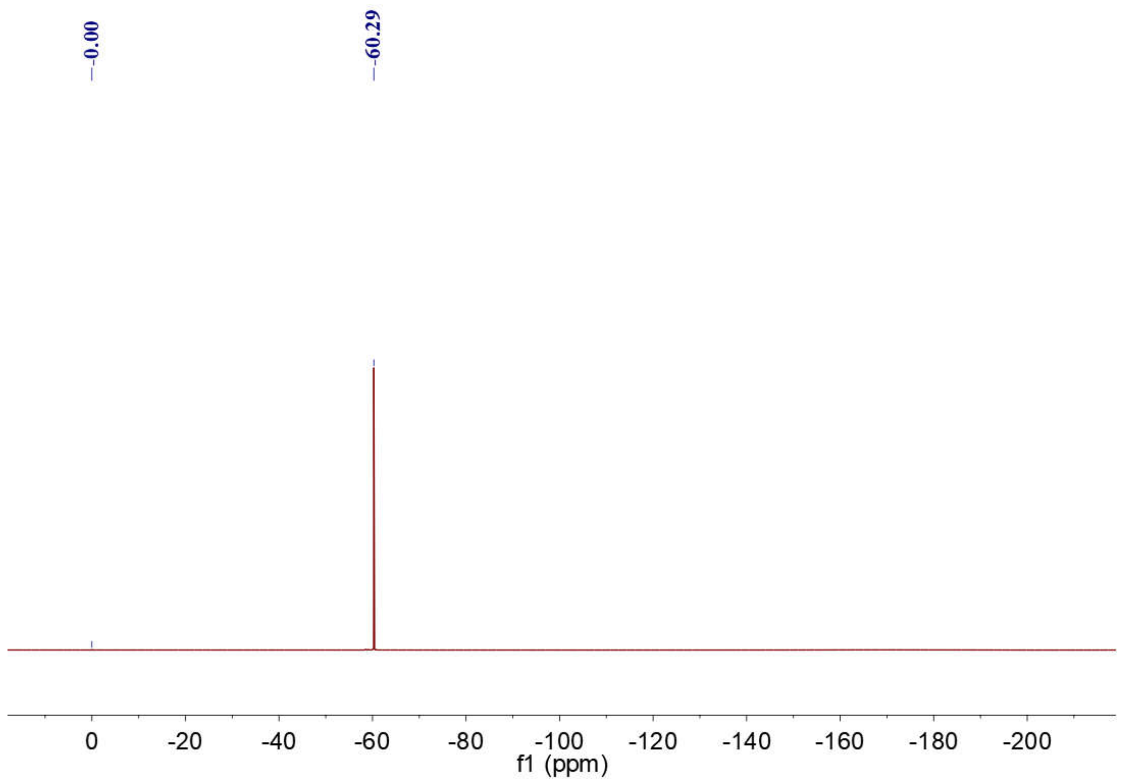

-0.00
-60.29
0
-20
-40
-60
-80
-100
-120
-140
-160
-180
-200
f1 (ppm)

NMR spectra of product 47

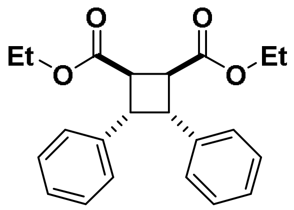

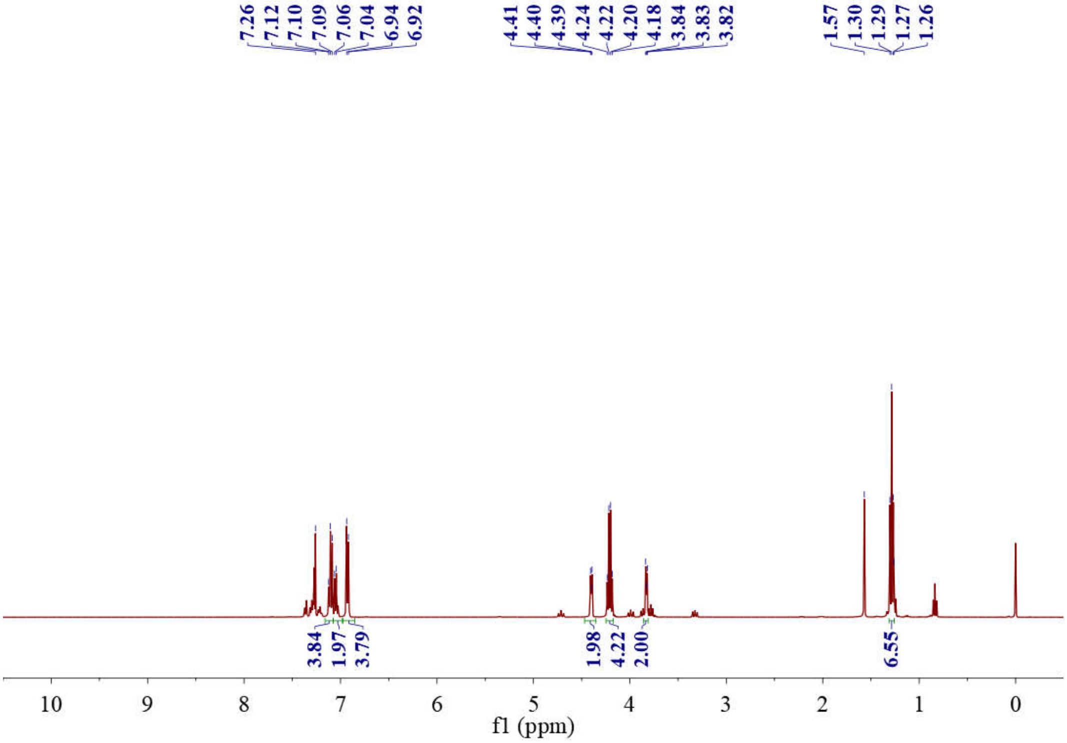

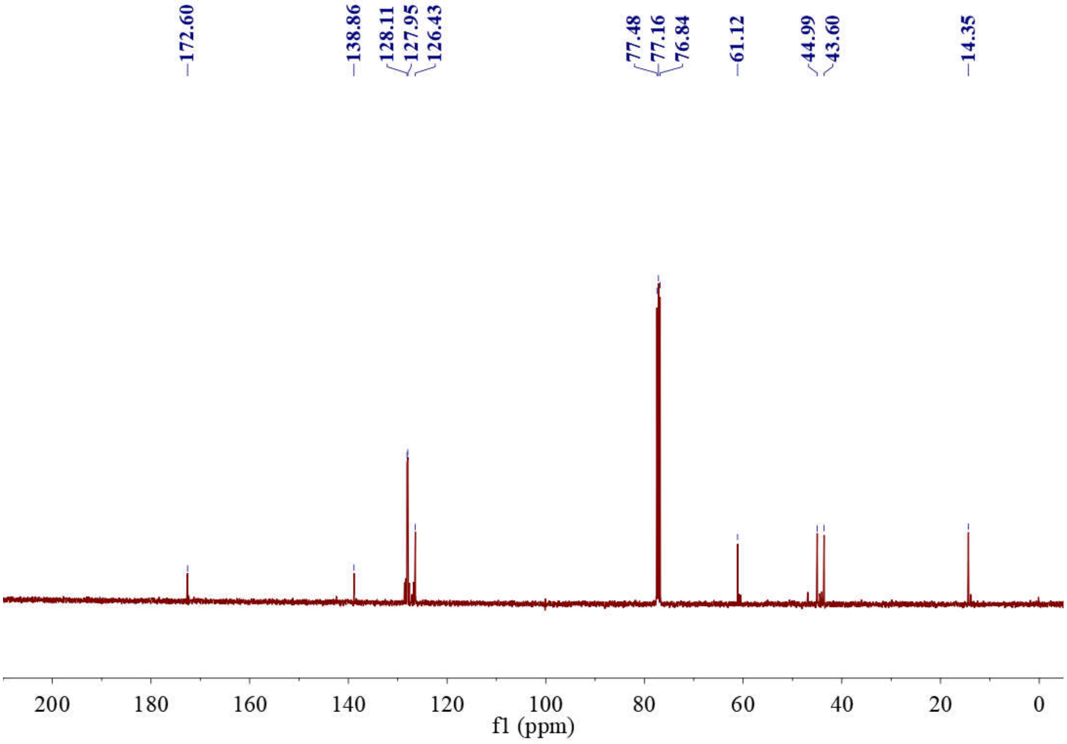

NMR spectra of product 48

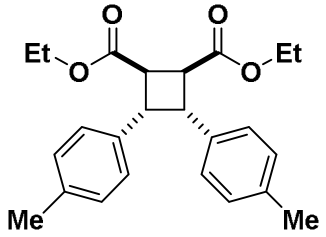

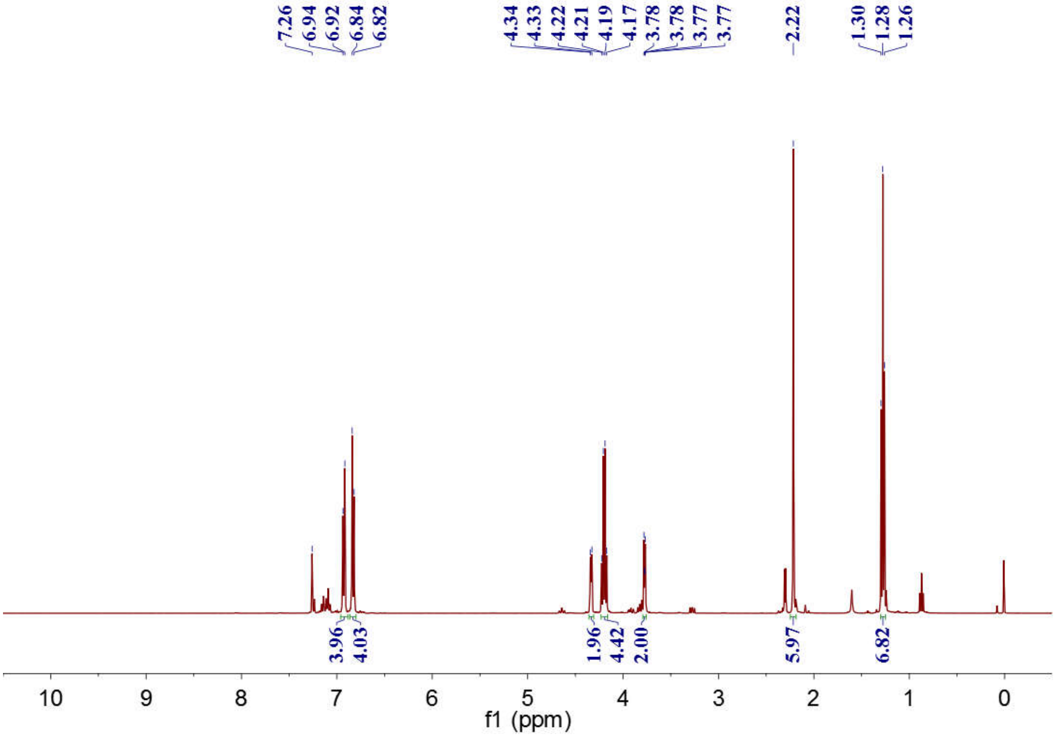

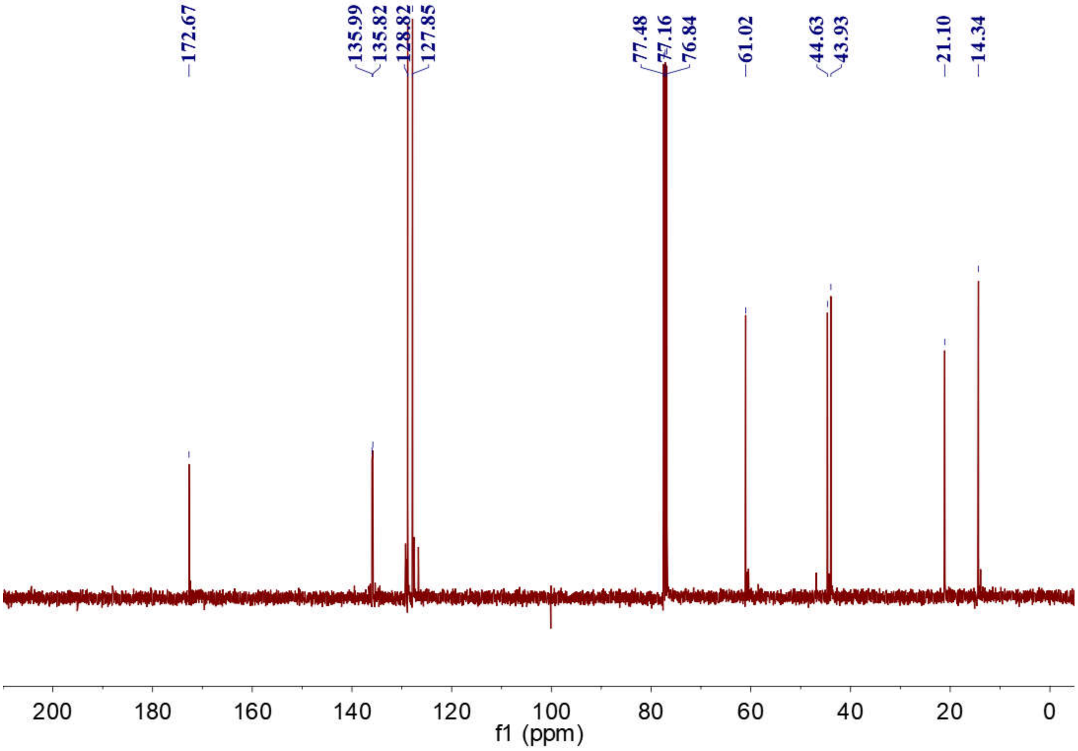

NMR spectra of product 49

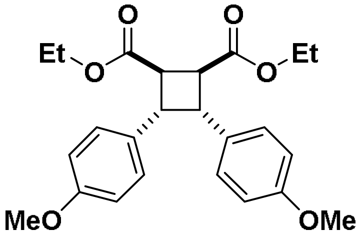

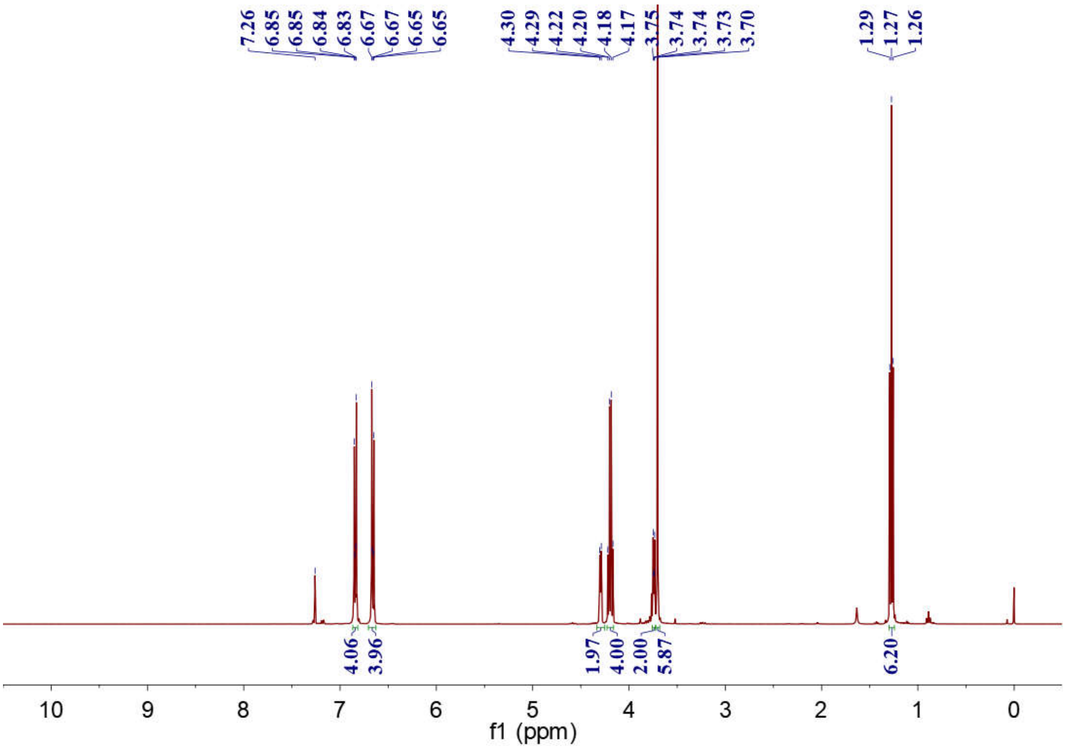

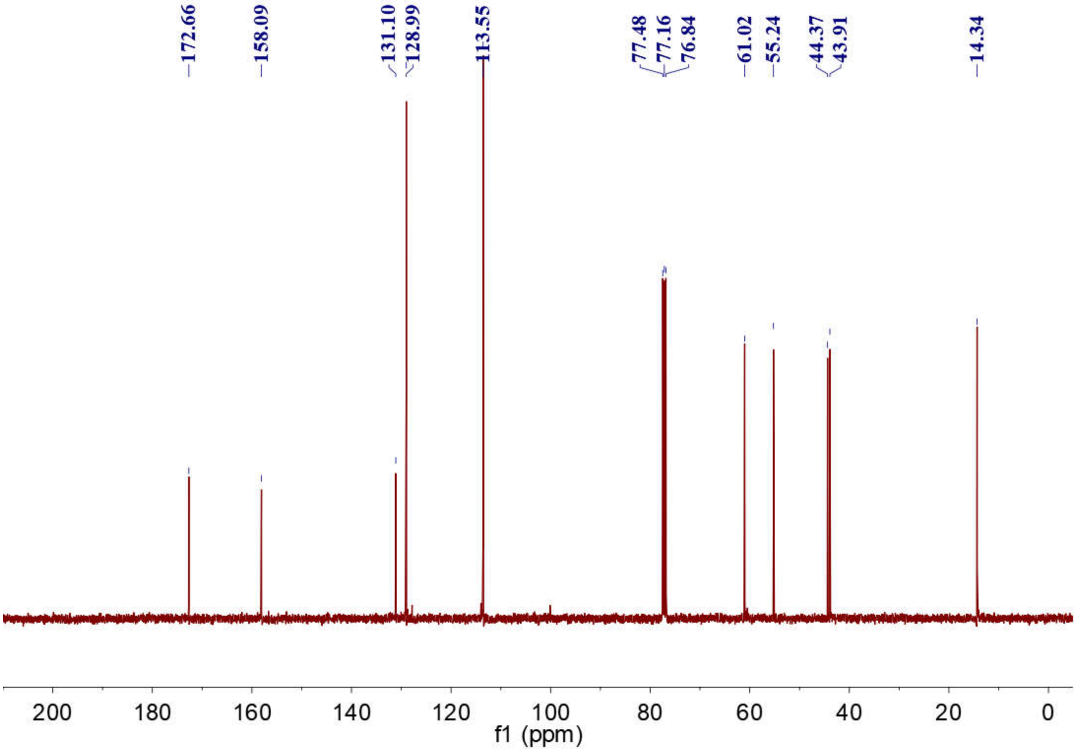

NMR spectra of product 50

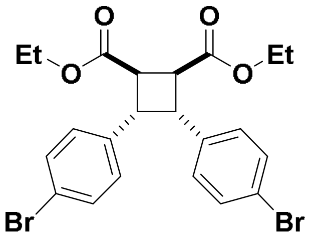

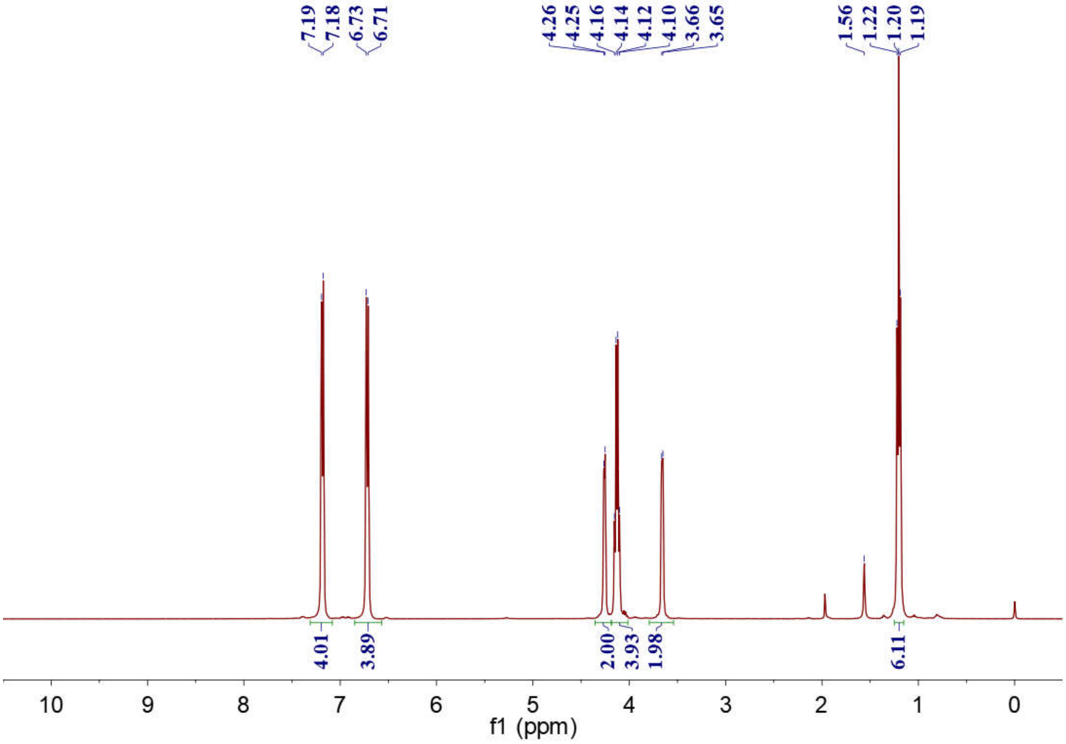

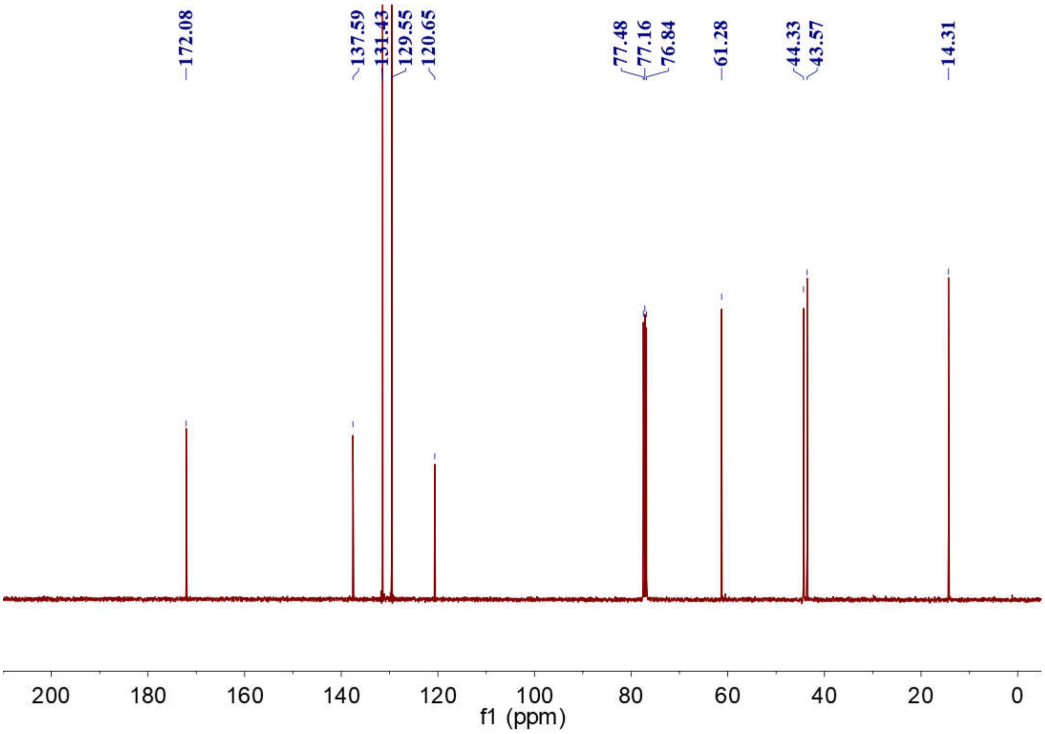

NMR spectra of product 51

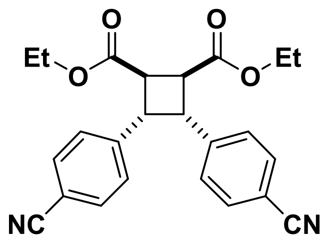

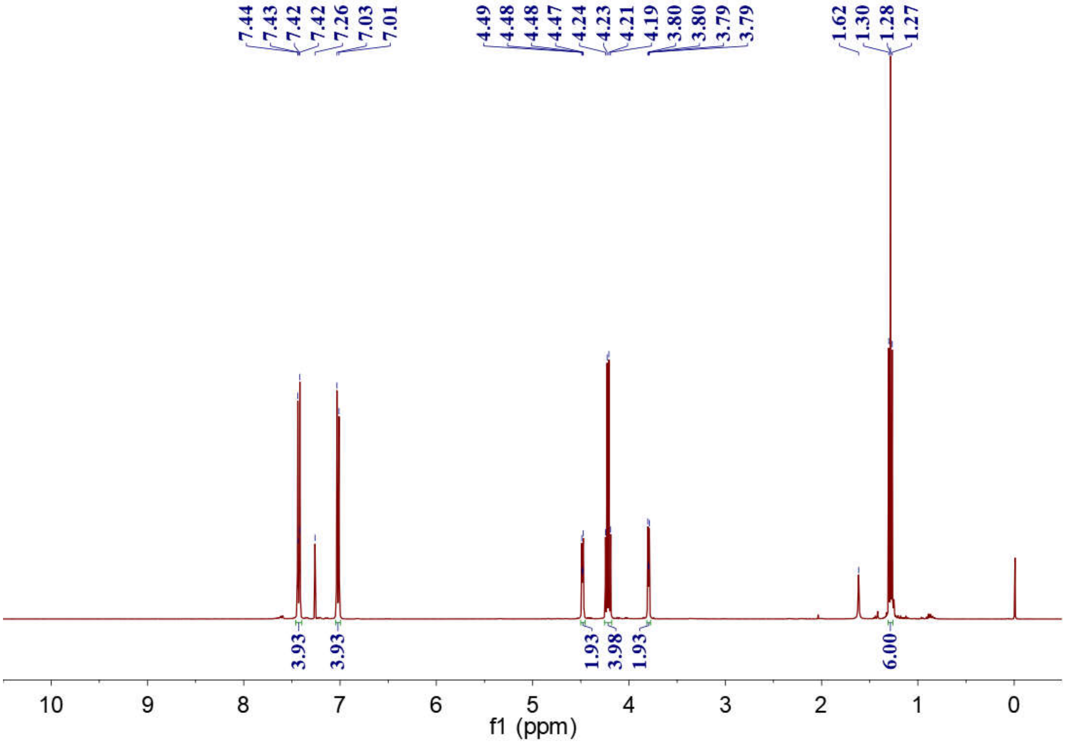

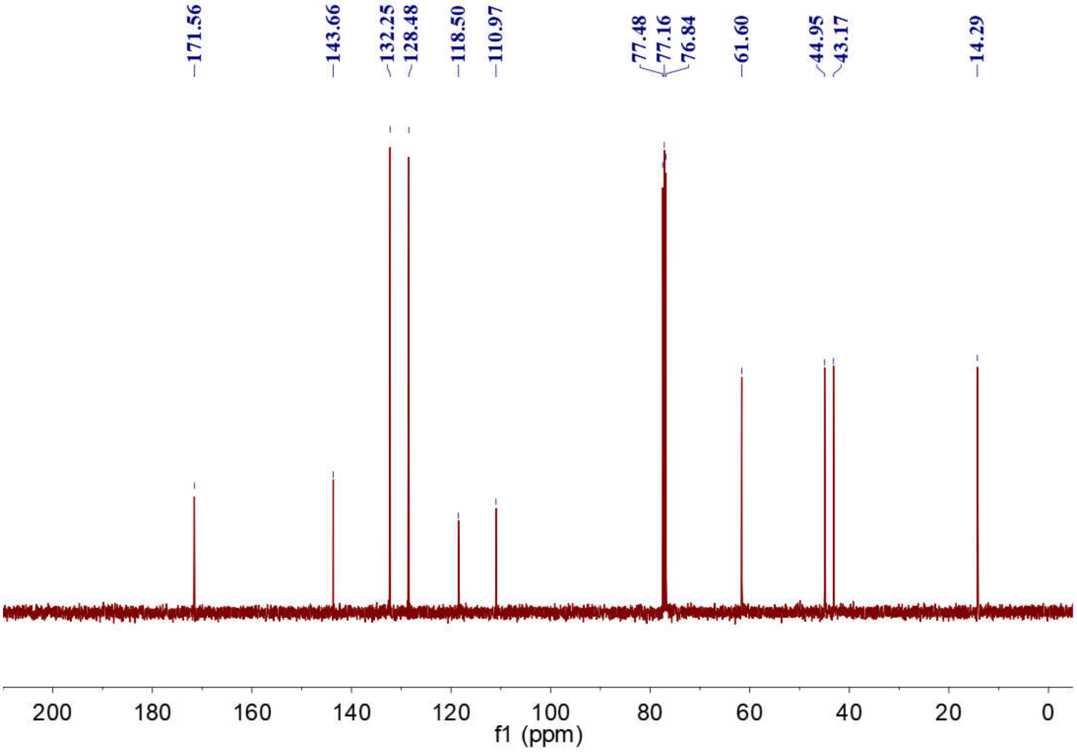

NMR spectra of product 52

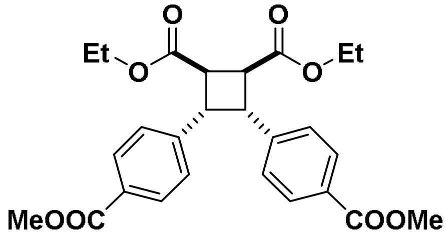

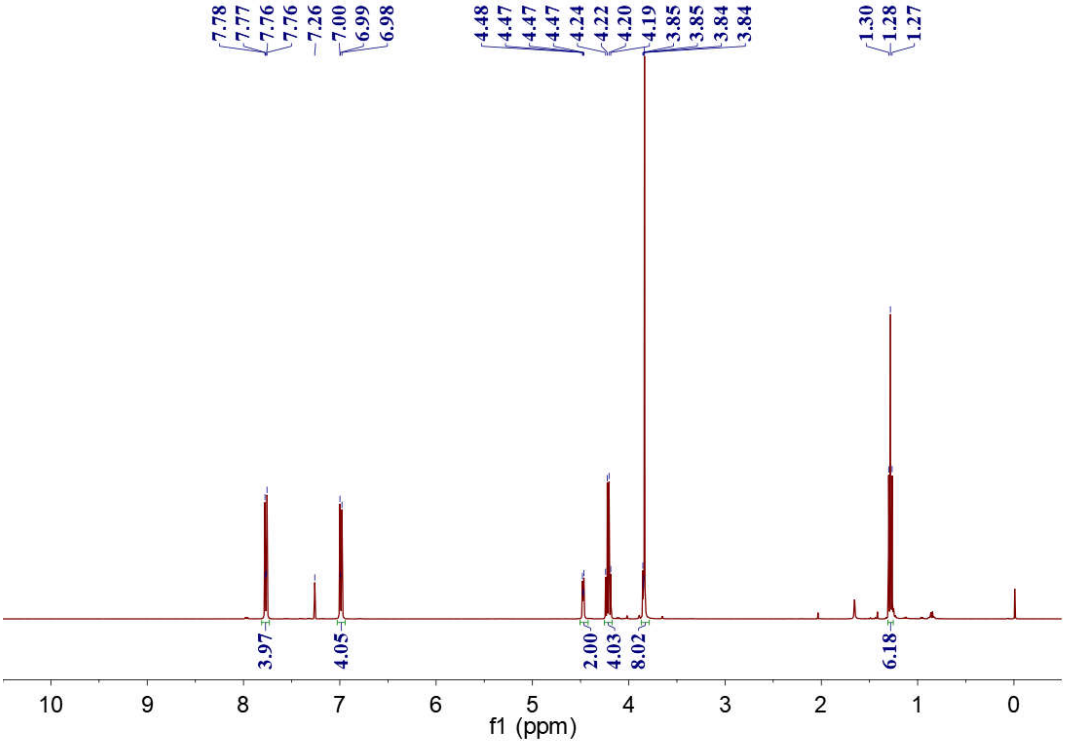

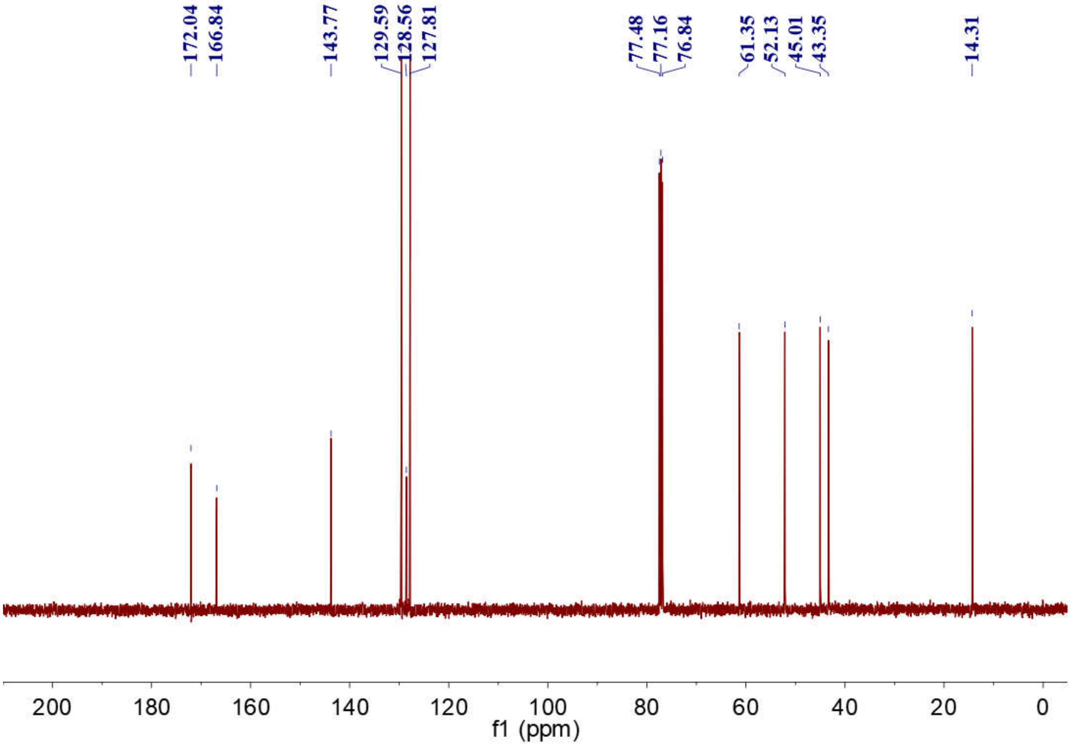

NMR spectra of product 53

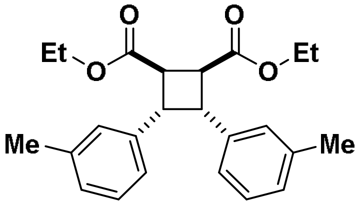

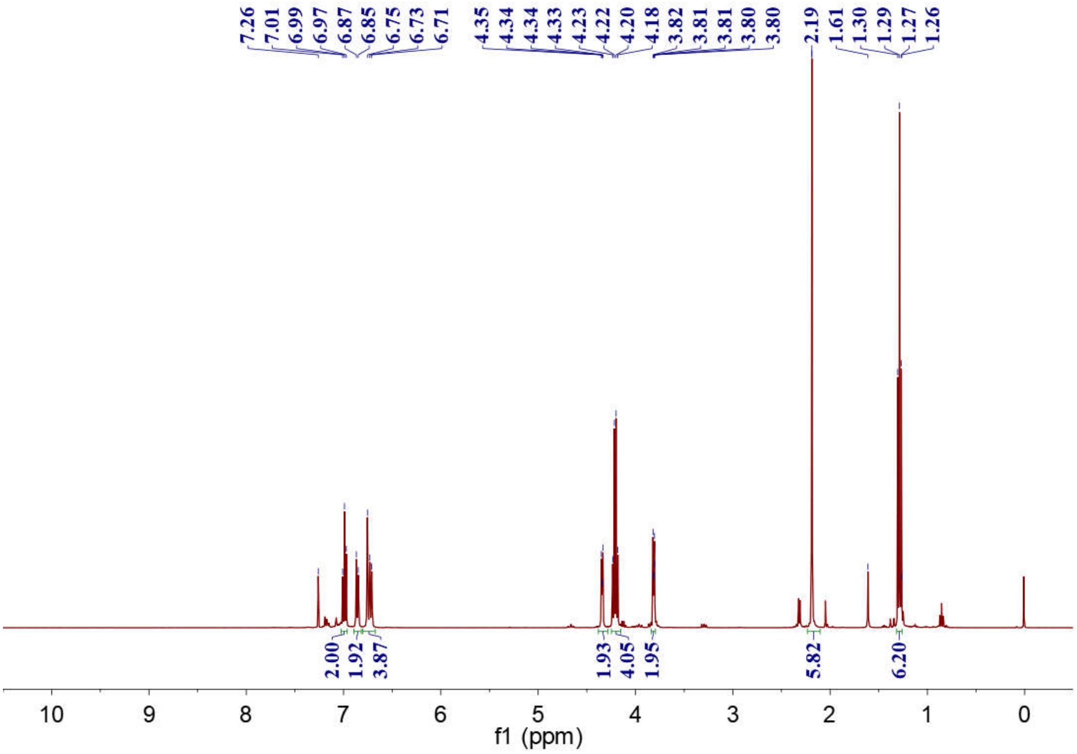

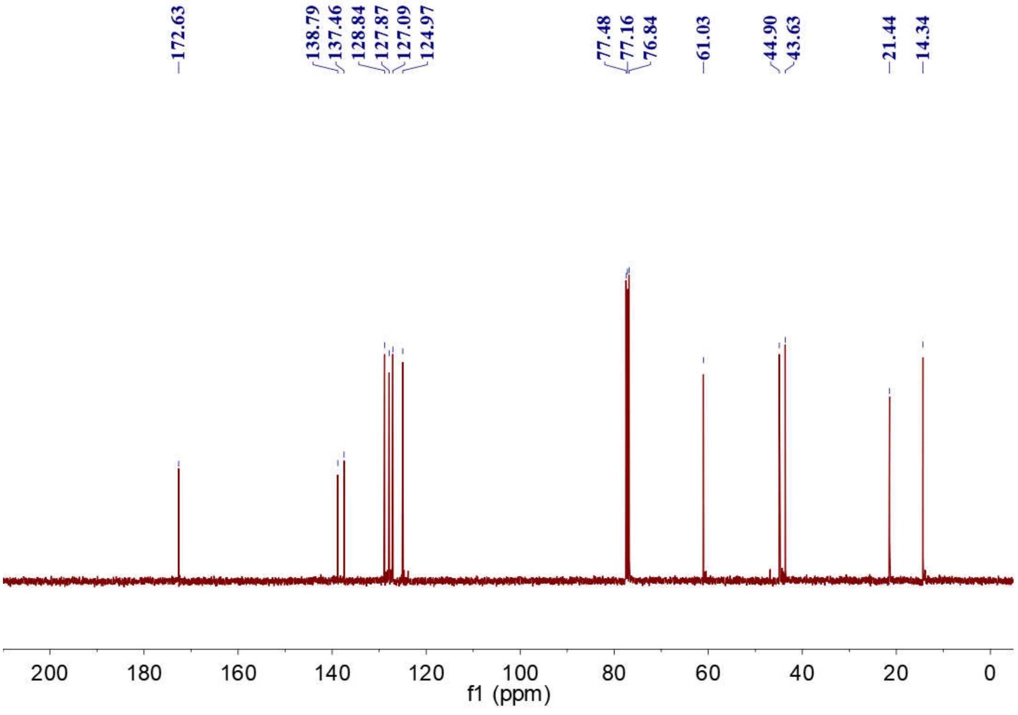

NMR spectra of product 54

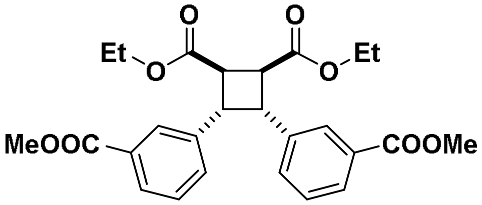

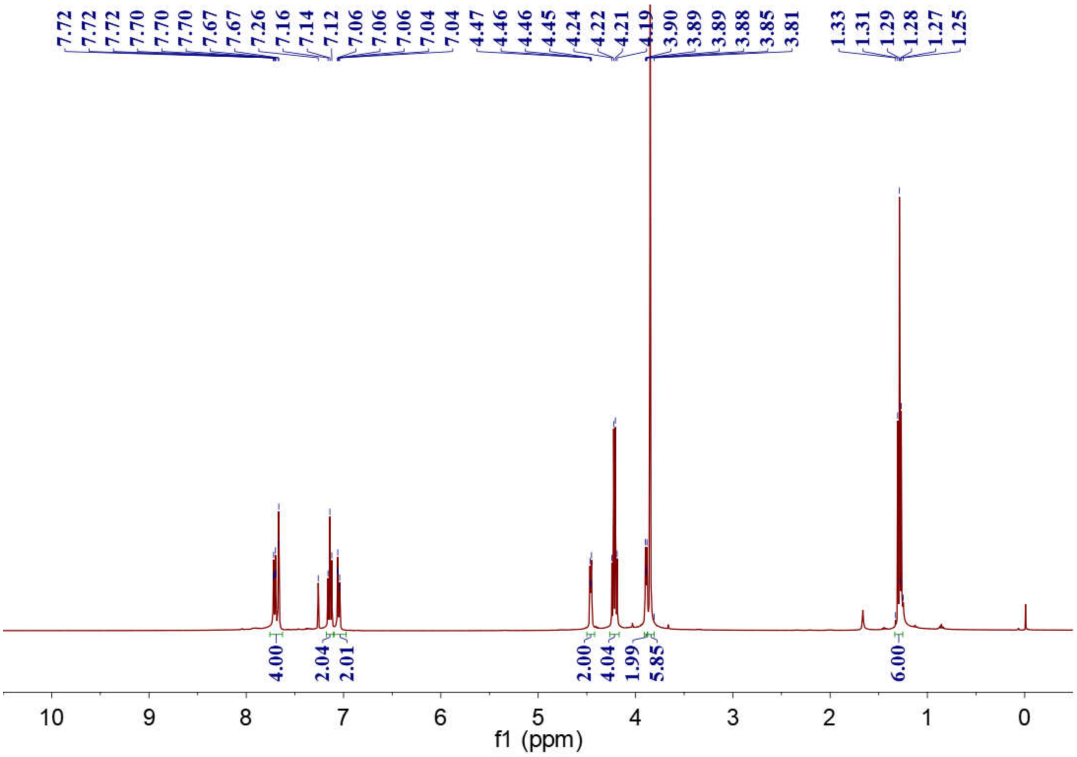

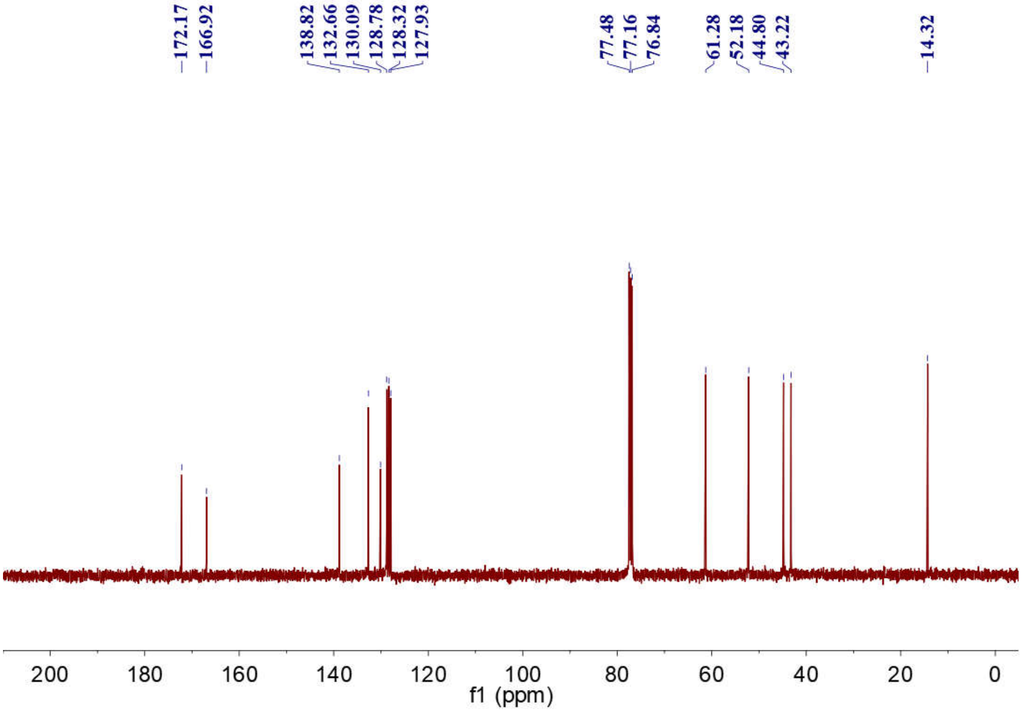

NMR spectra of product 55

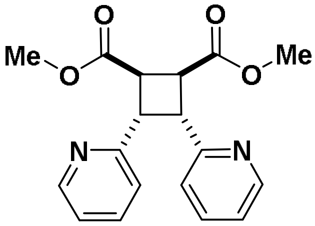

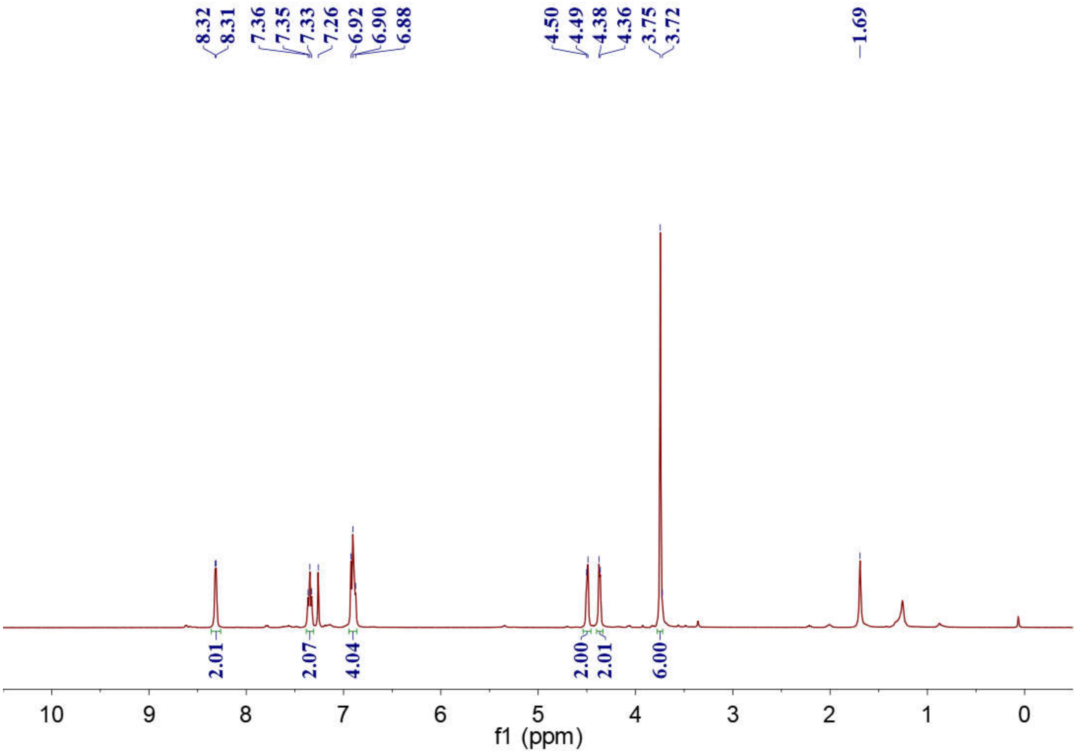

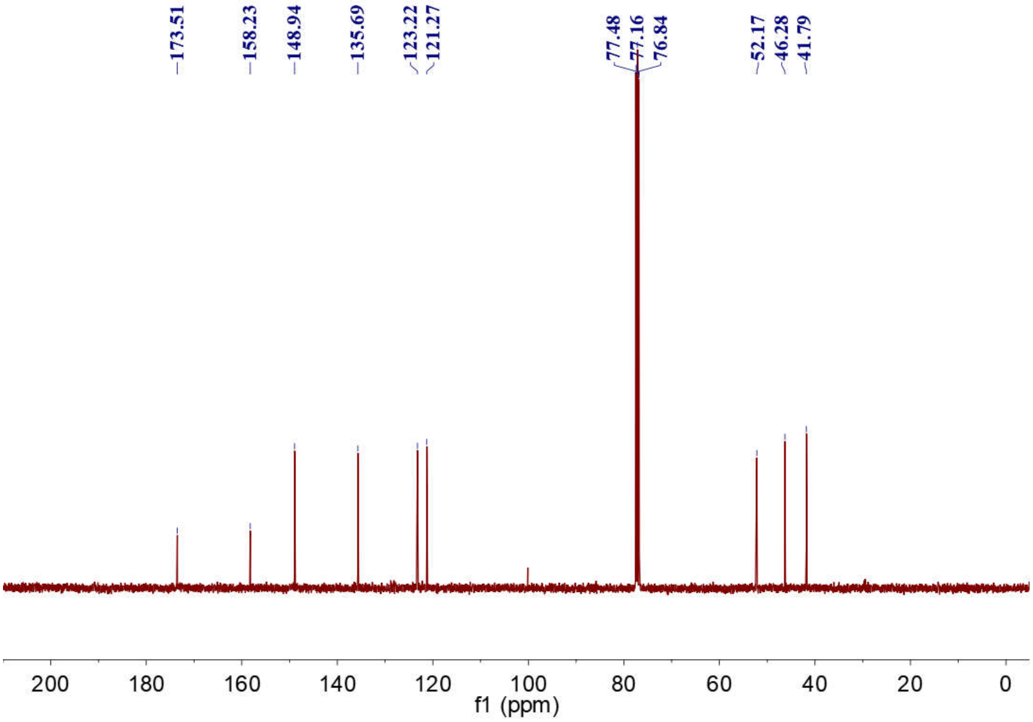

NMR spectra of product 56

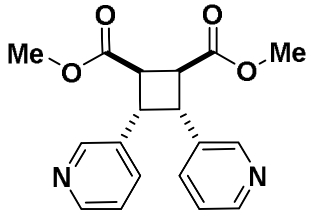

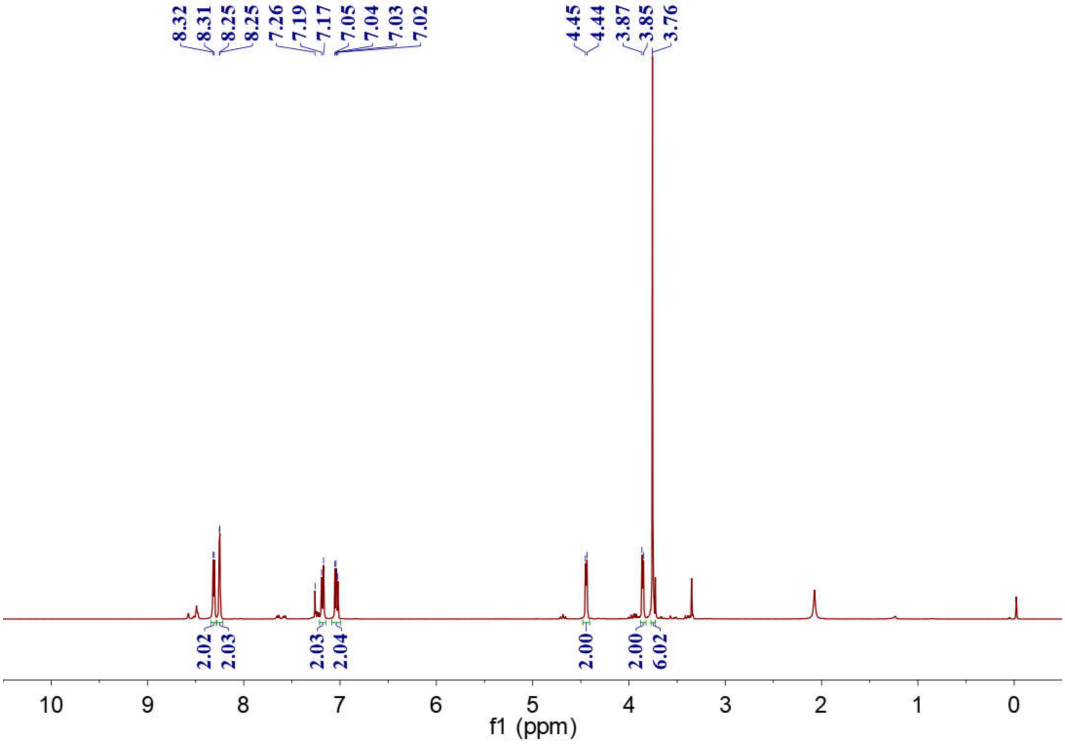

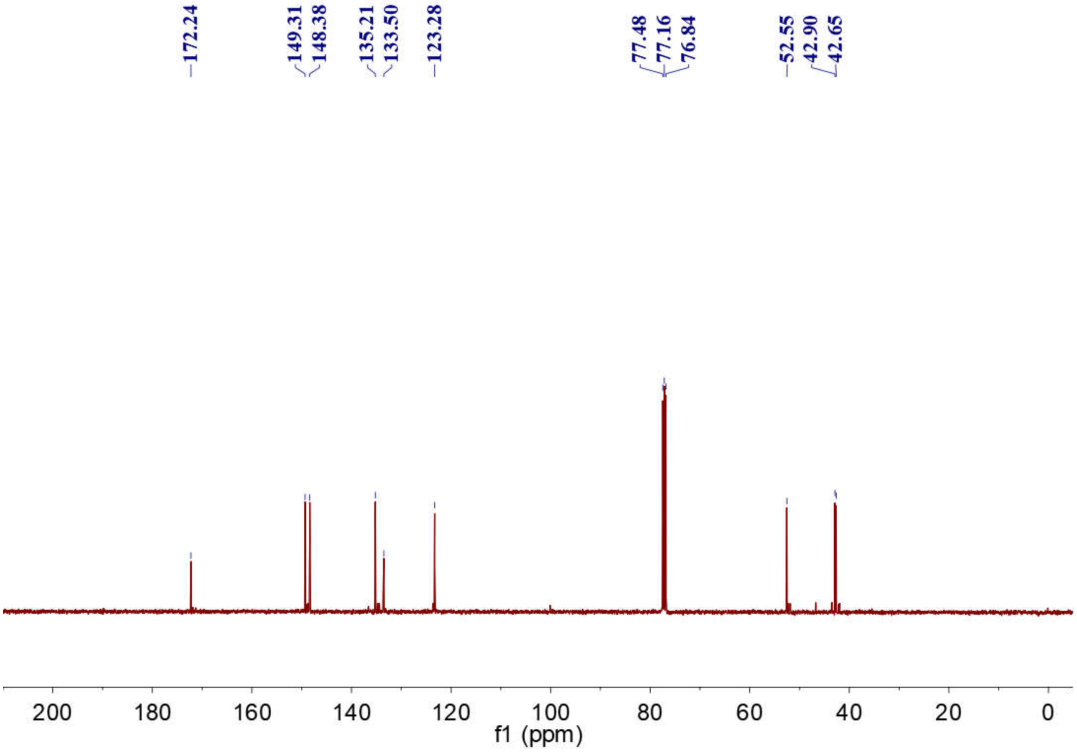

NMR spectra of product 57

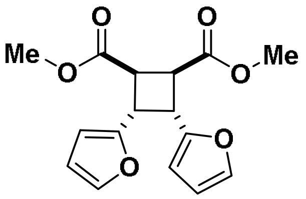

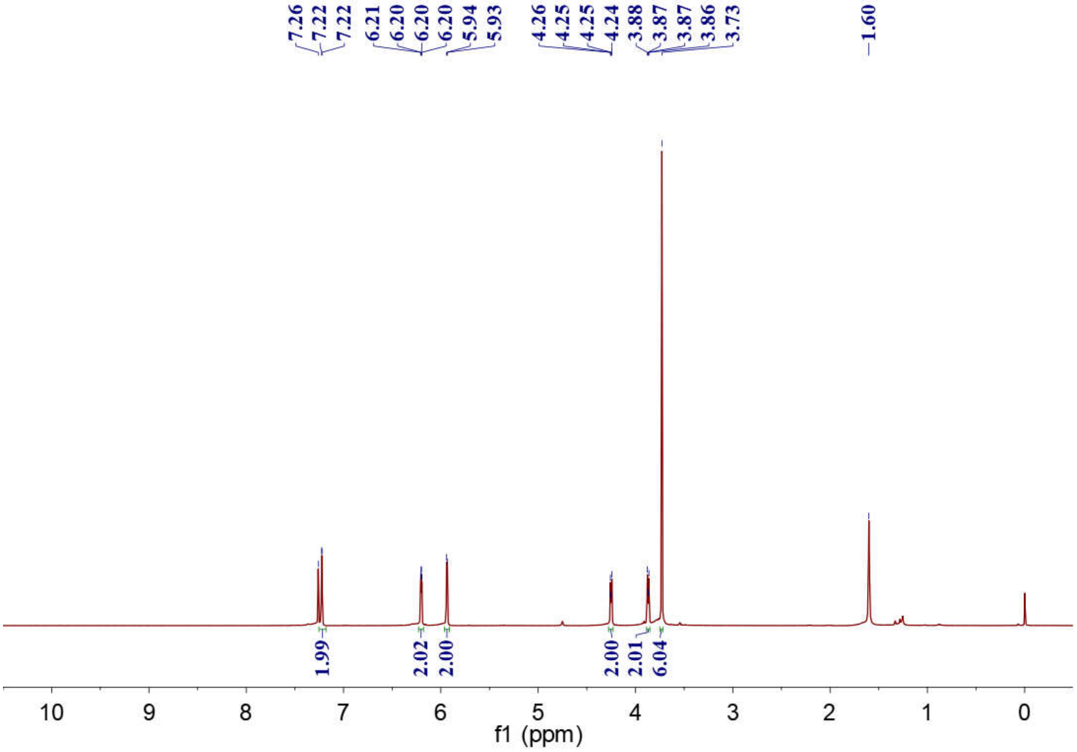

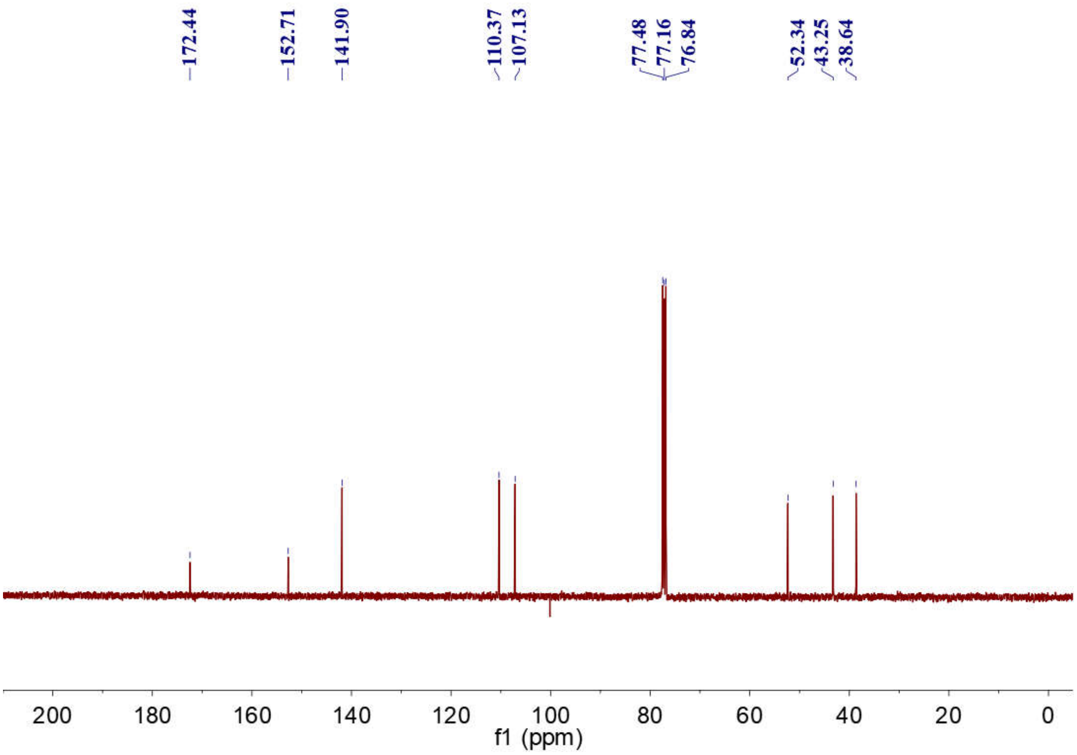

NMR spectra of product 58

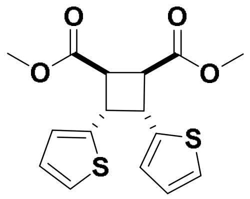

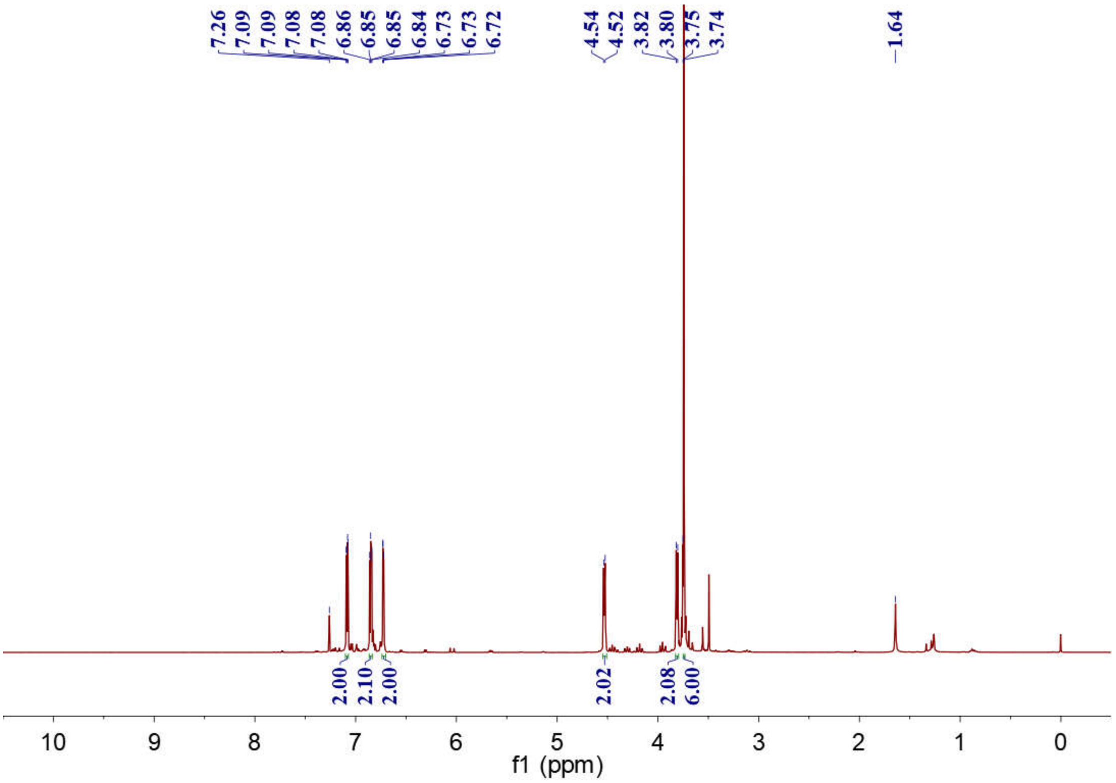

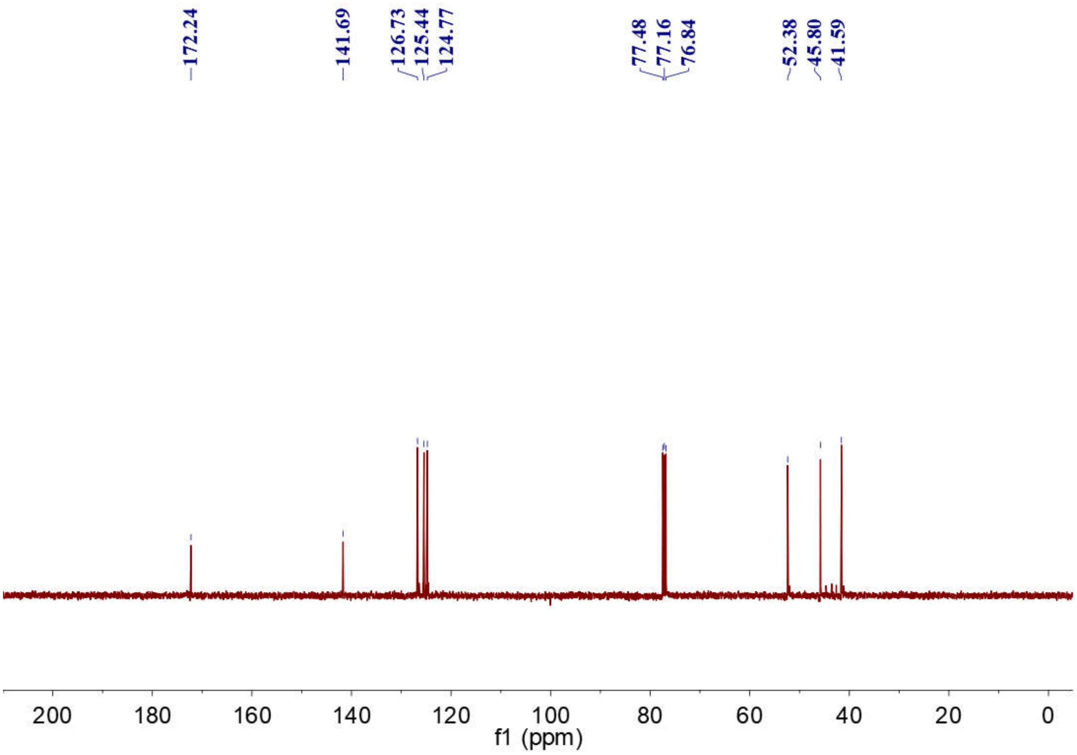

NMR spectra of product 59

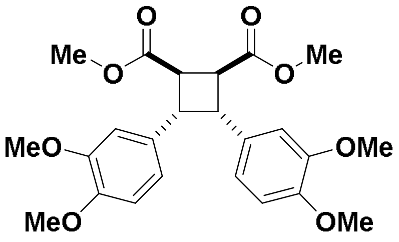

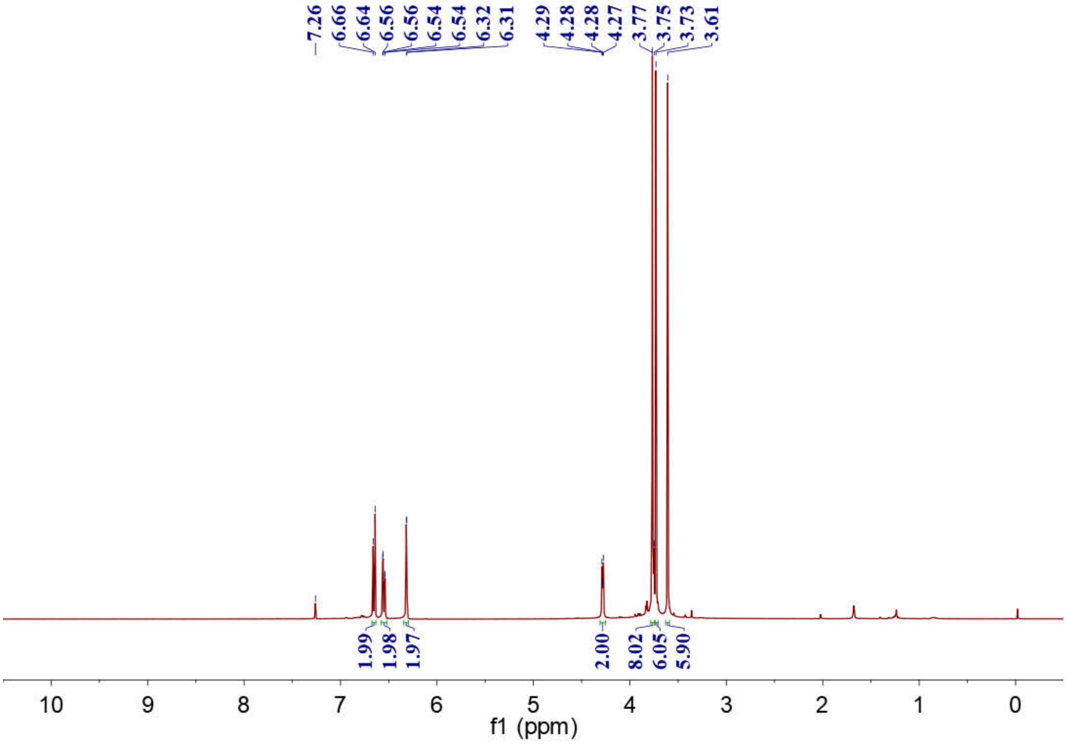

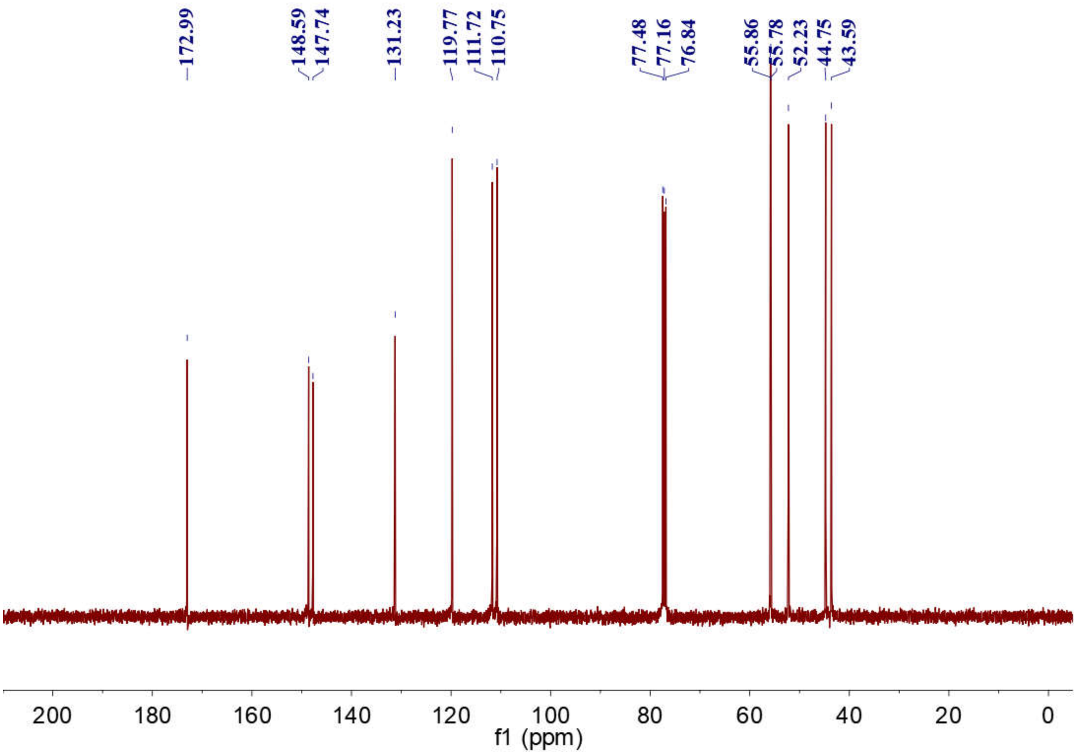

NMR spectra of product 60

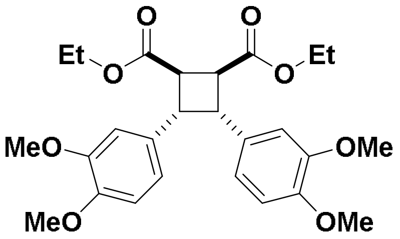

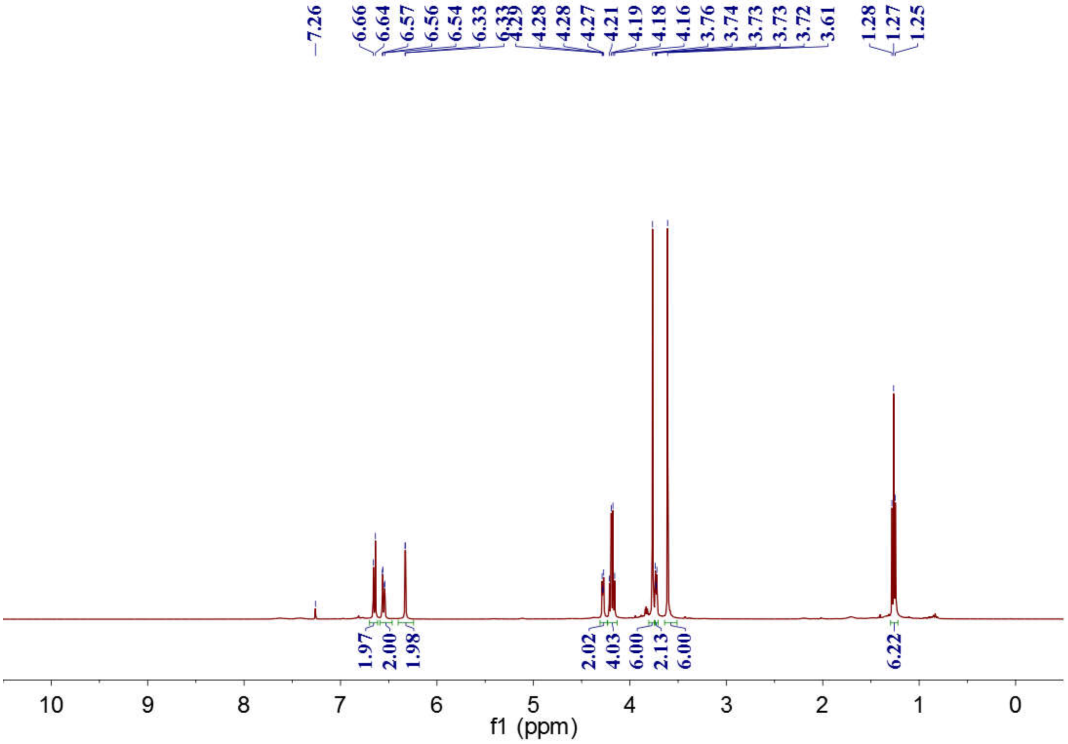

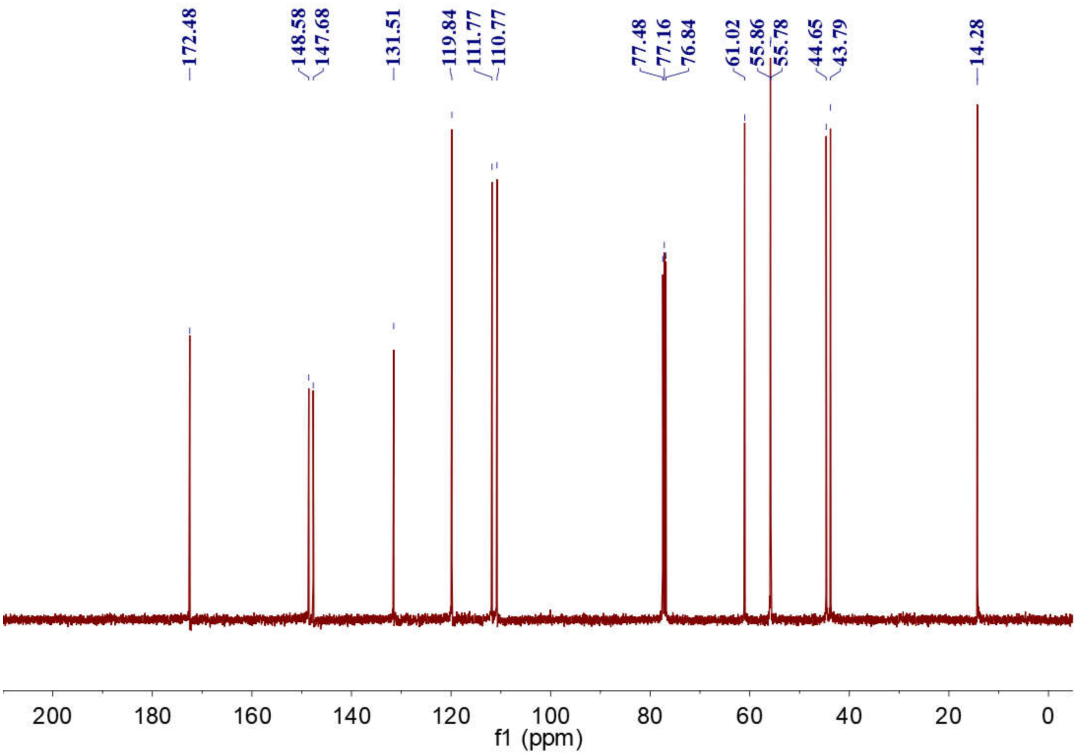

NMR spectra of product 61

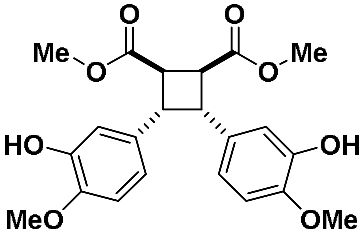

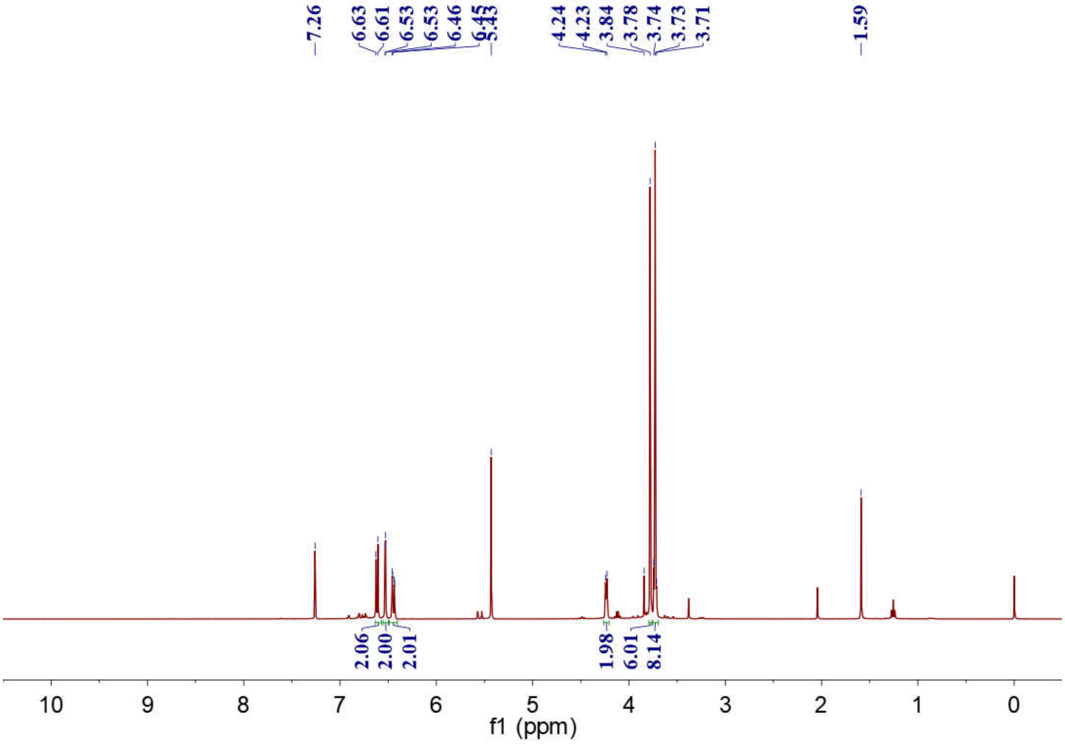

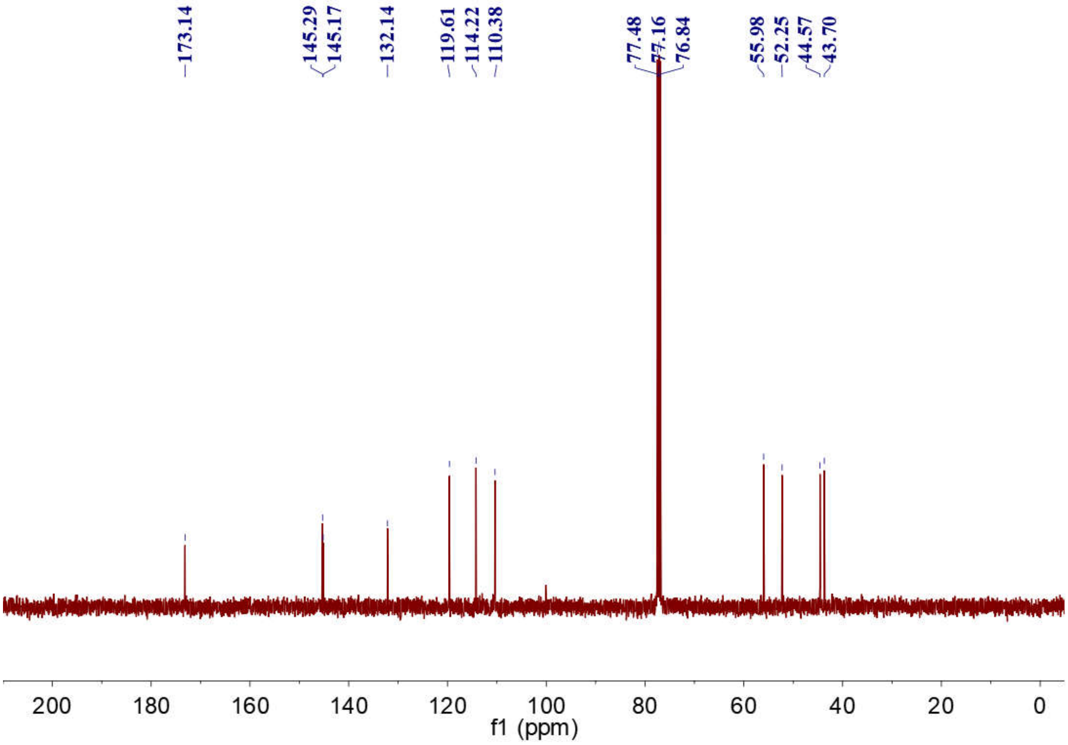

NMR spectra of product 62

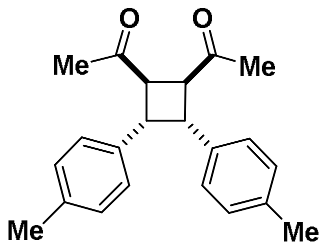

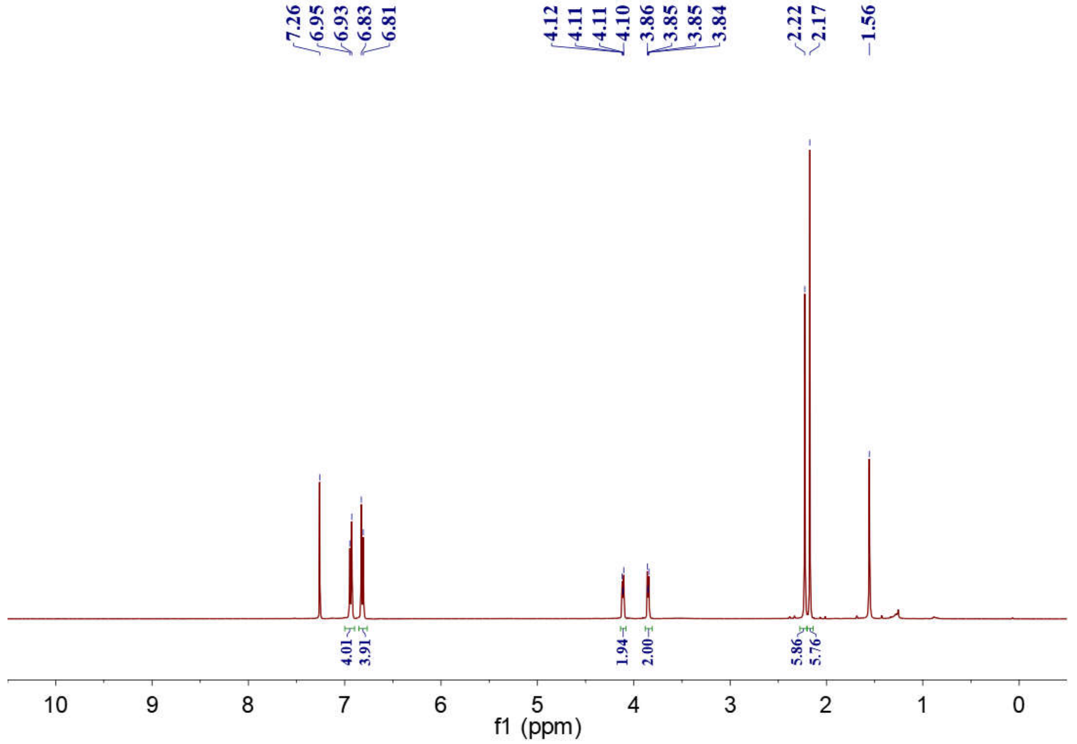

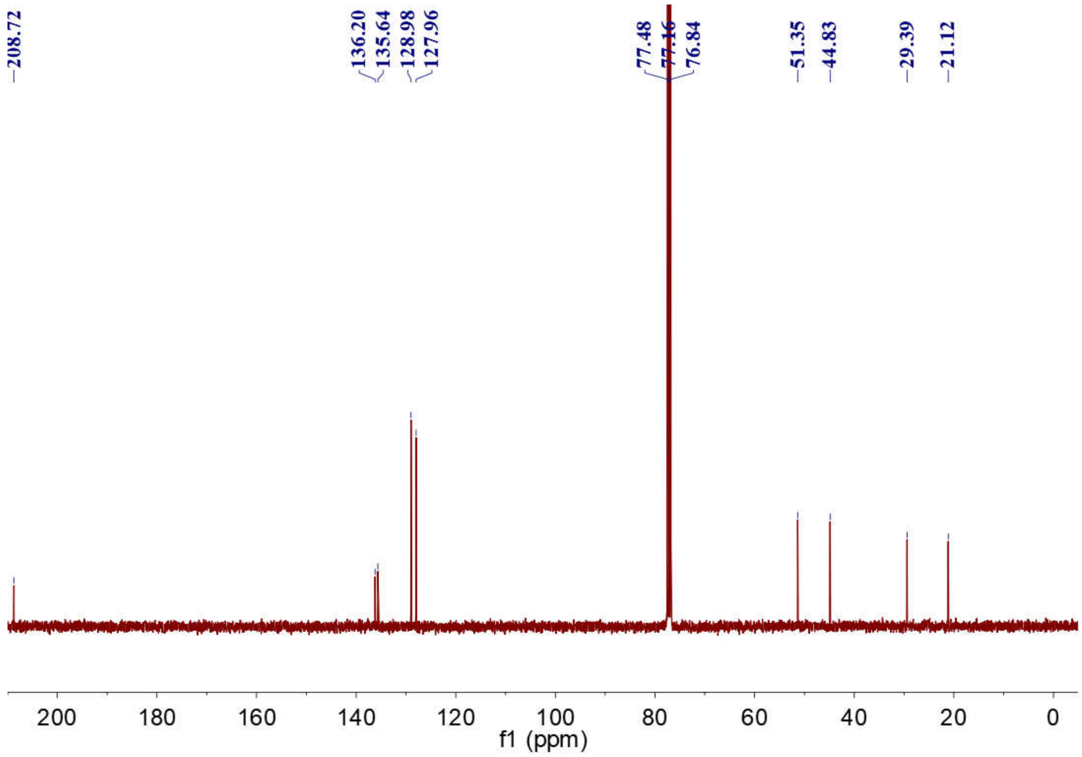

NMR spectra of product 63

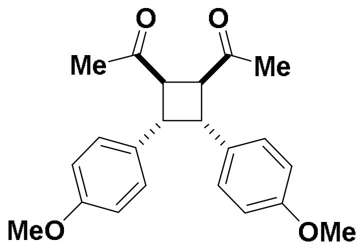

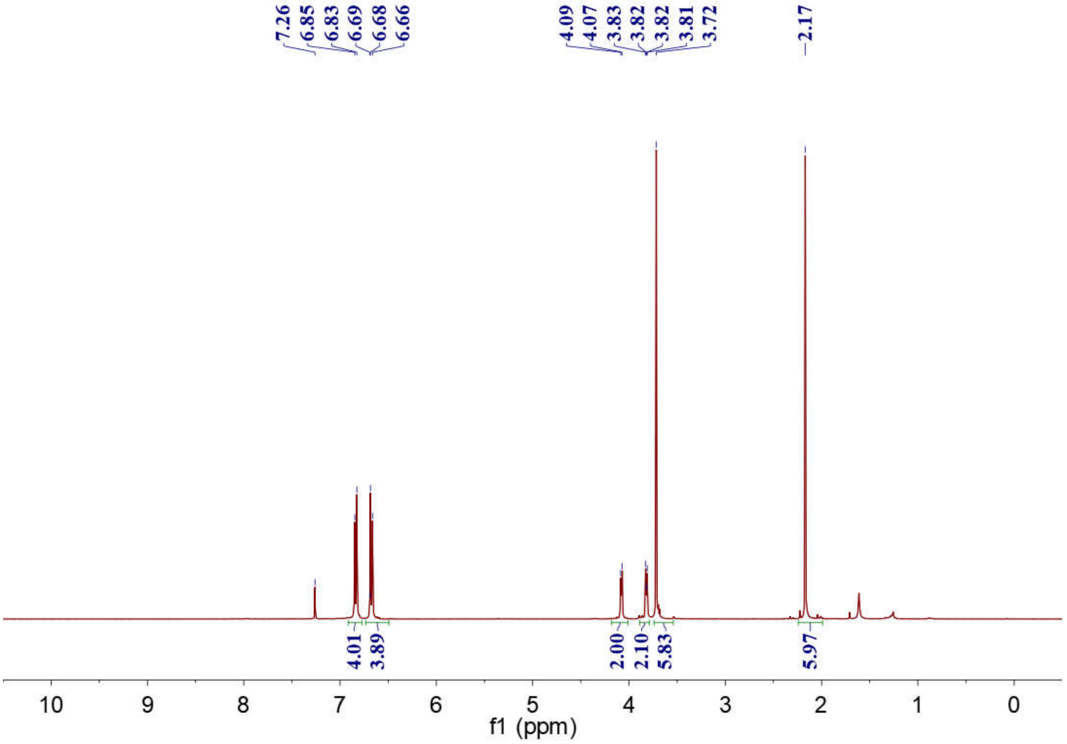

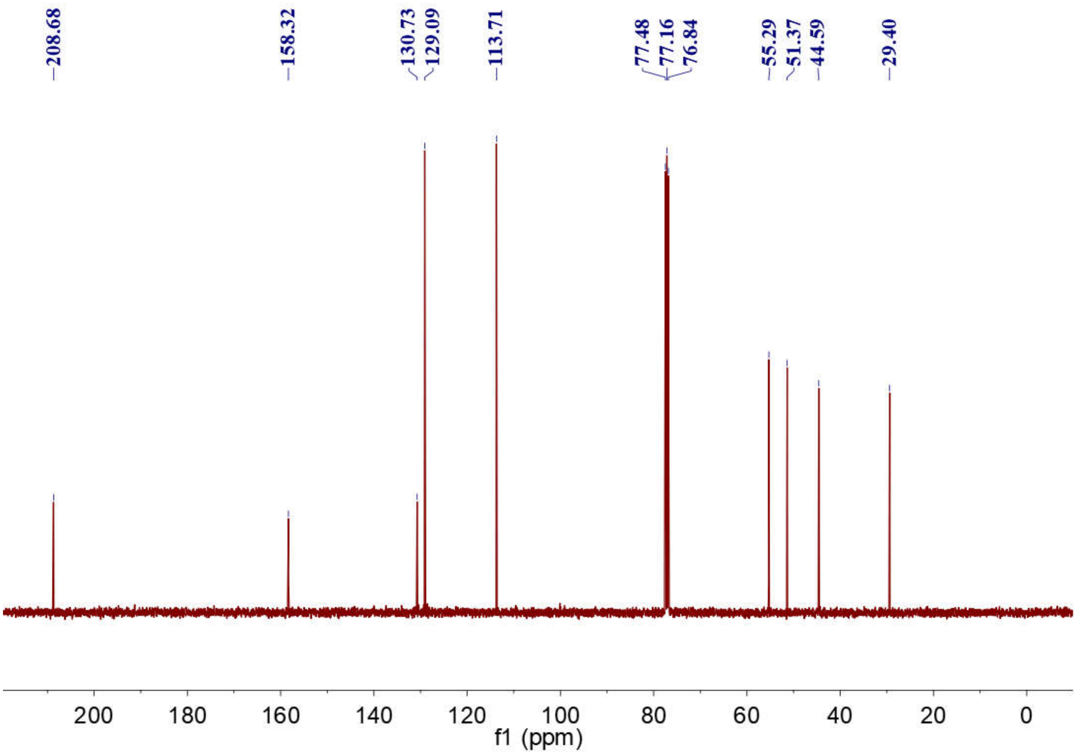

NMR spectra of product 64

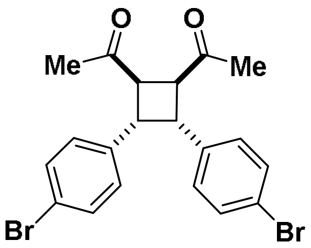

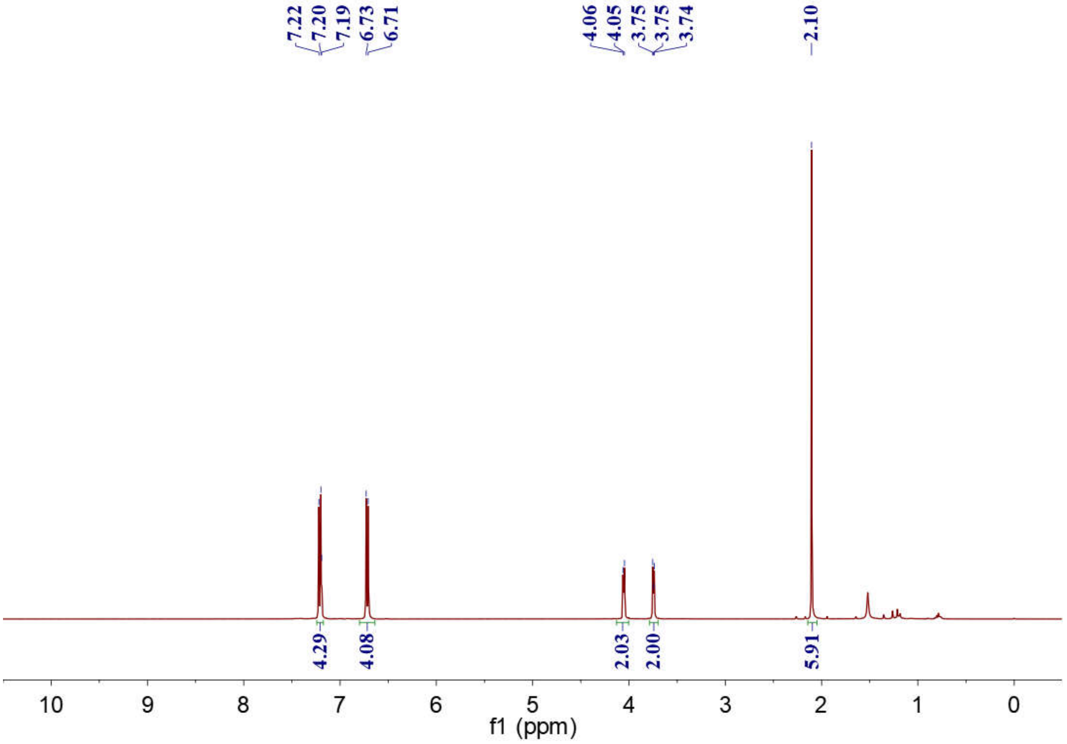

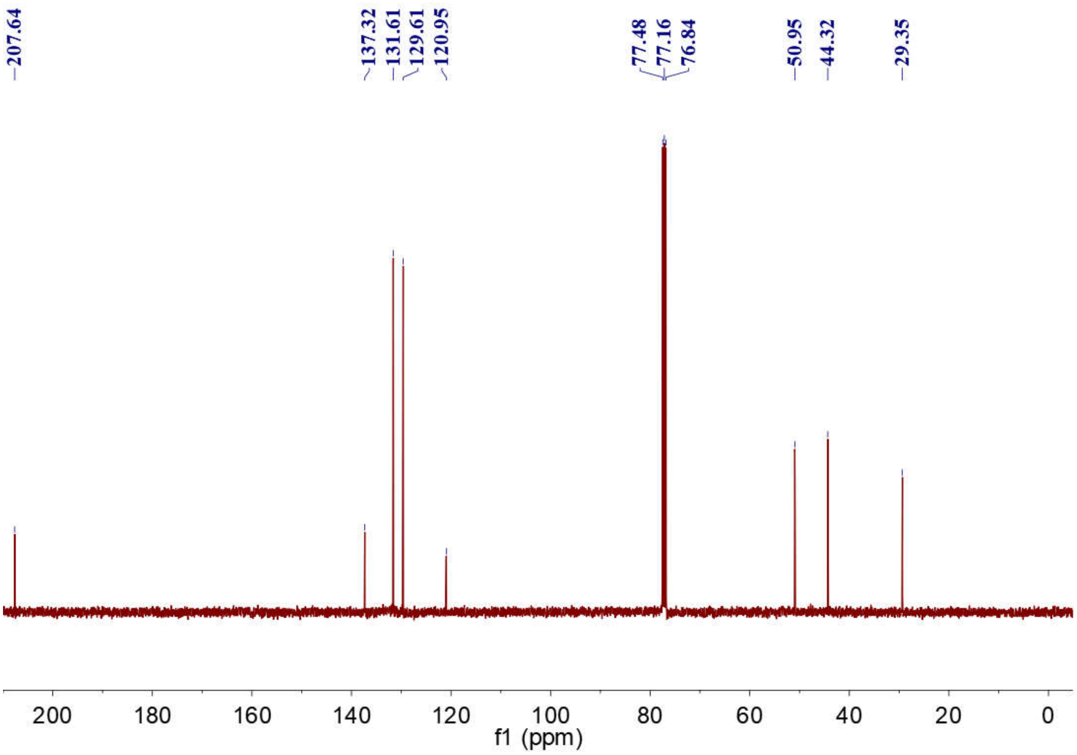

NMR spectra of product 65

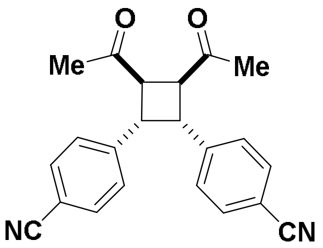

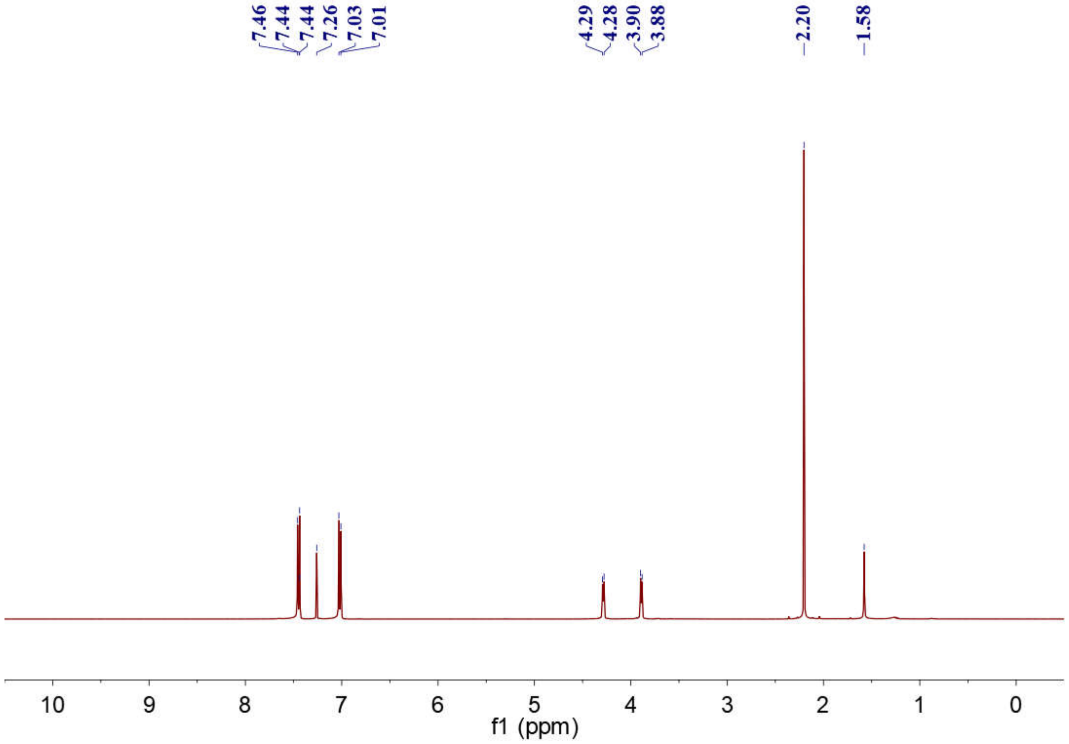

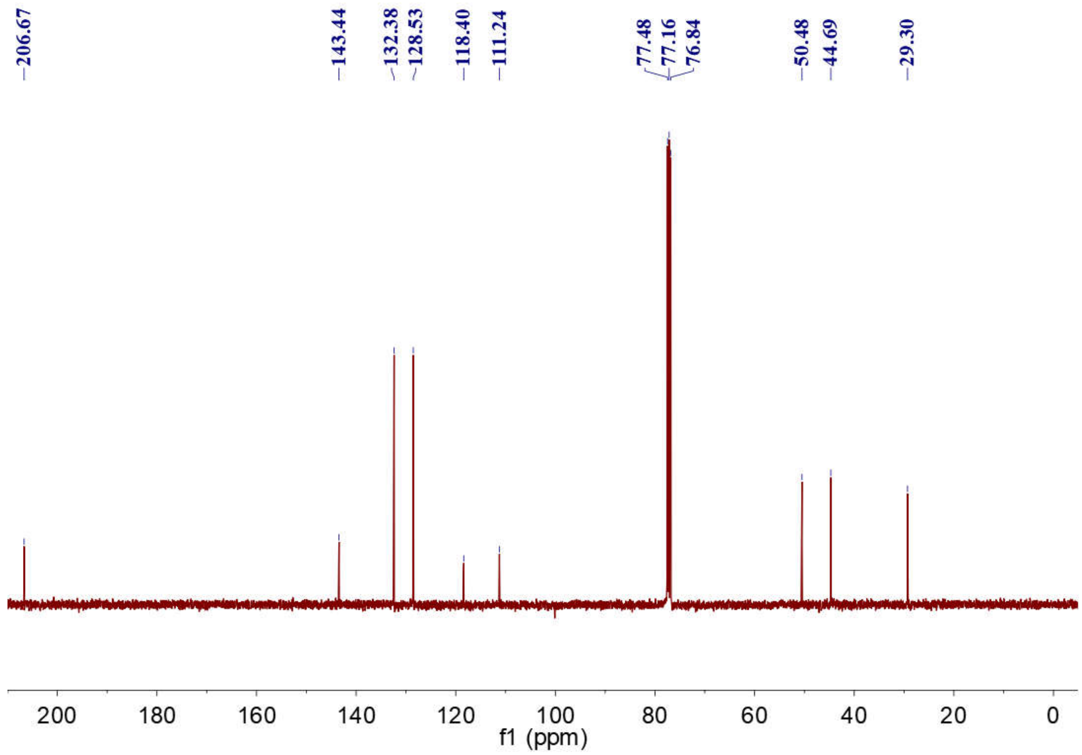

NMR spectra of product 66

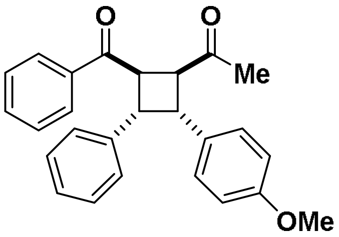

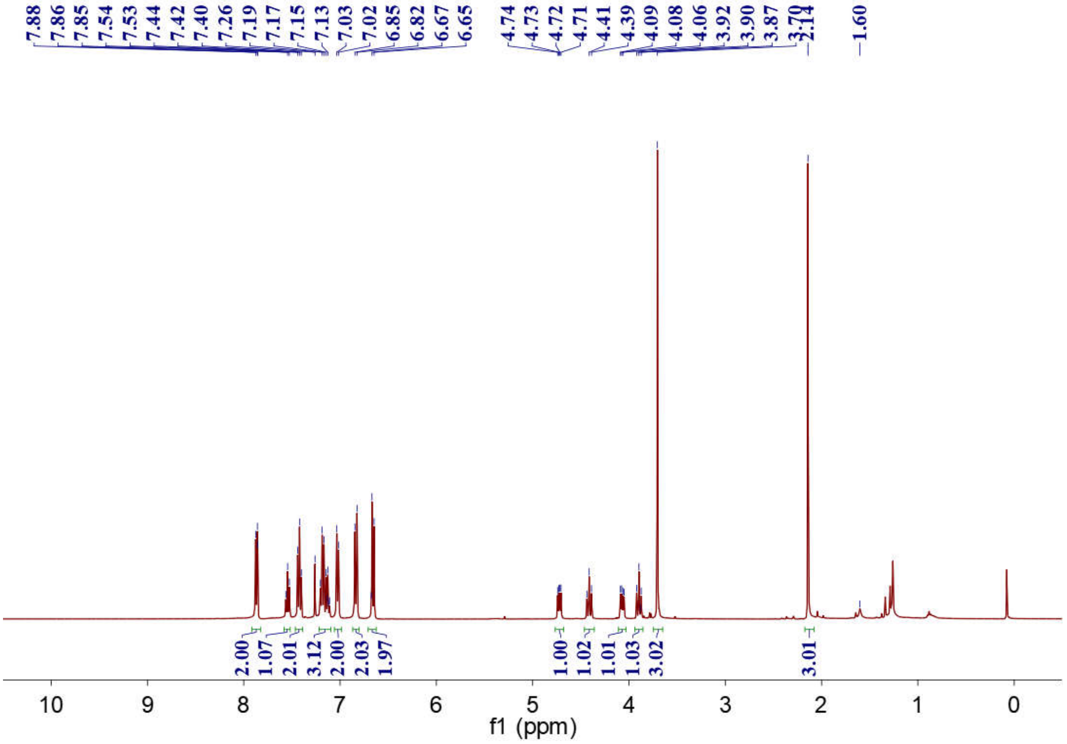

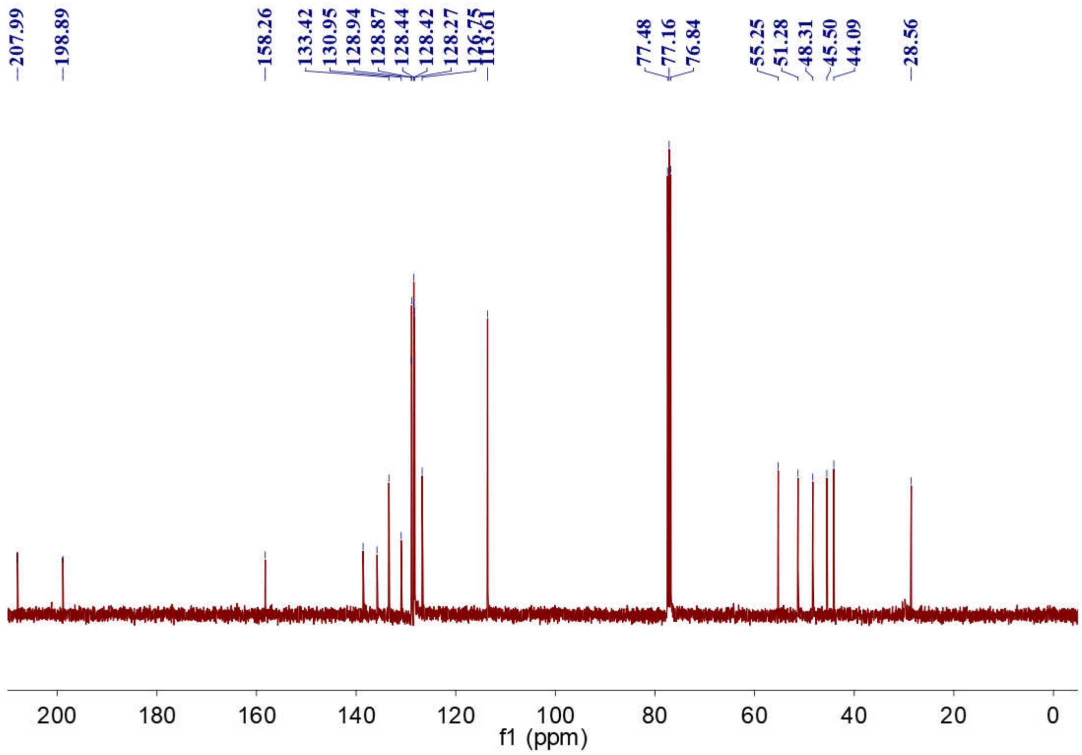

NMR spectra of product 67

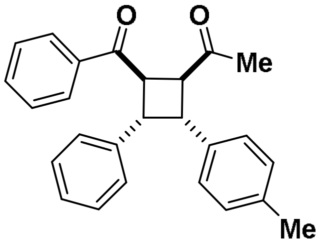

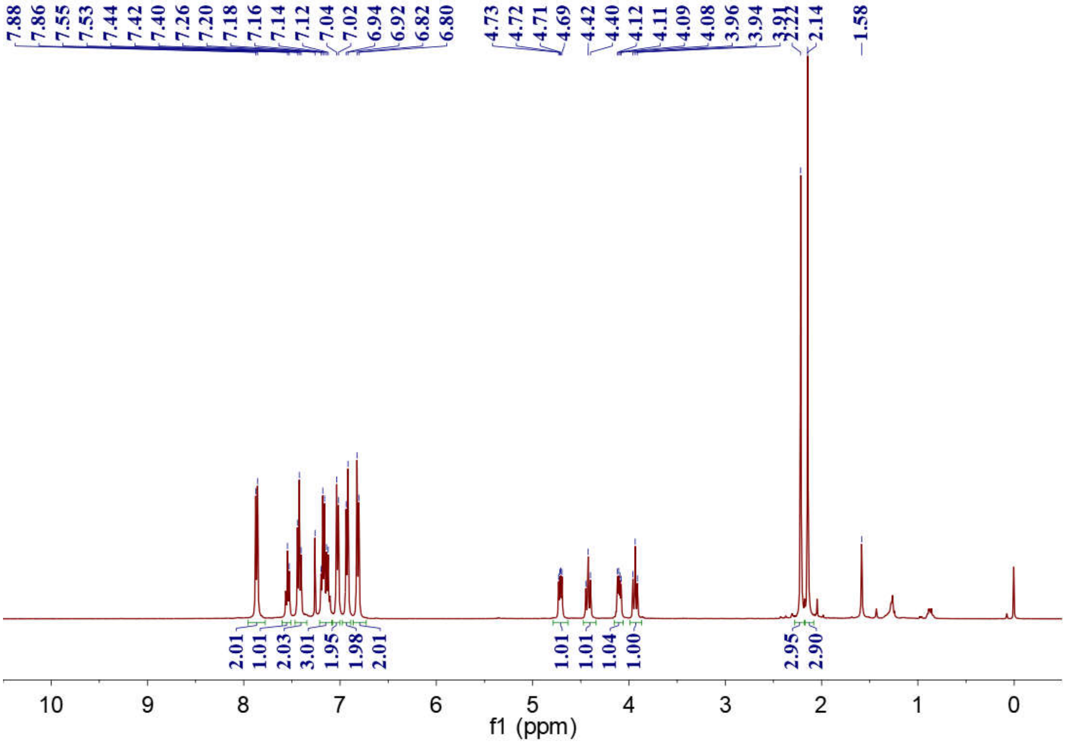

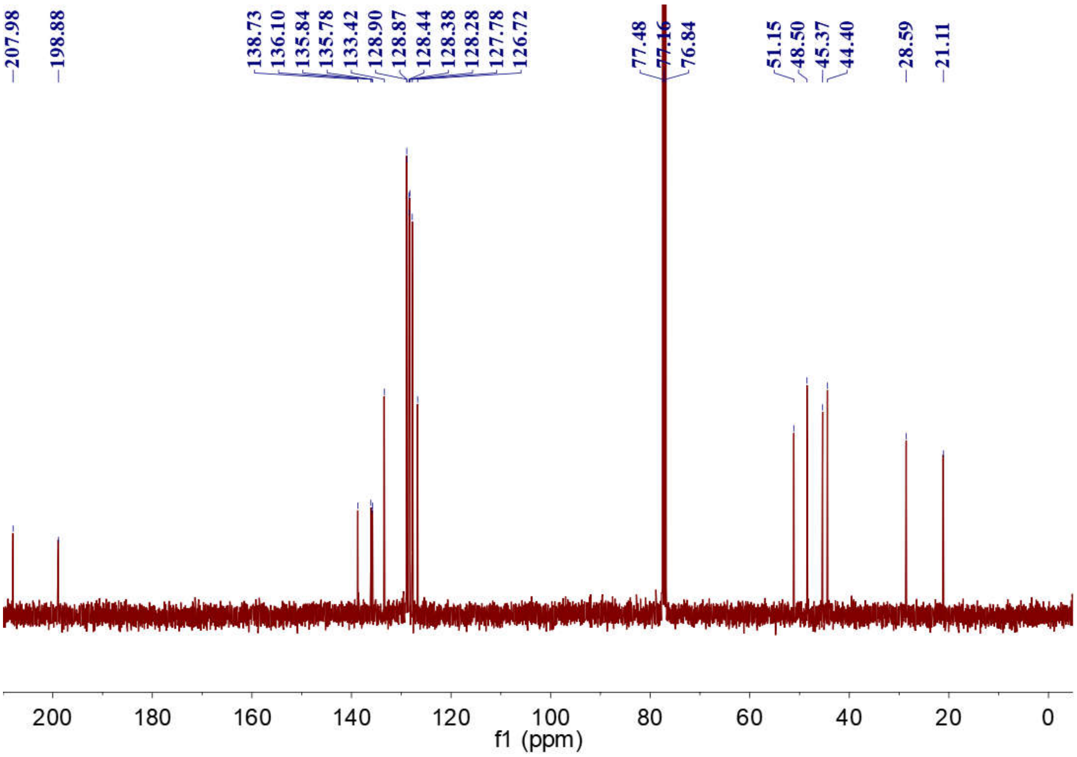

NMR spectra of product 68

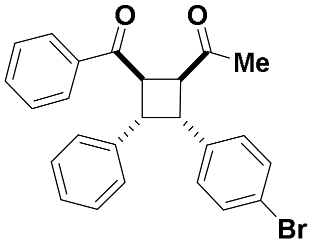

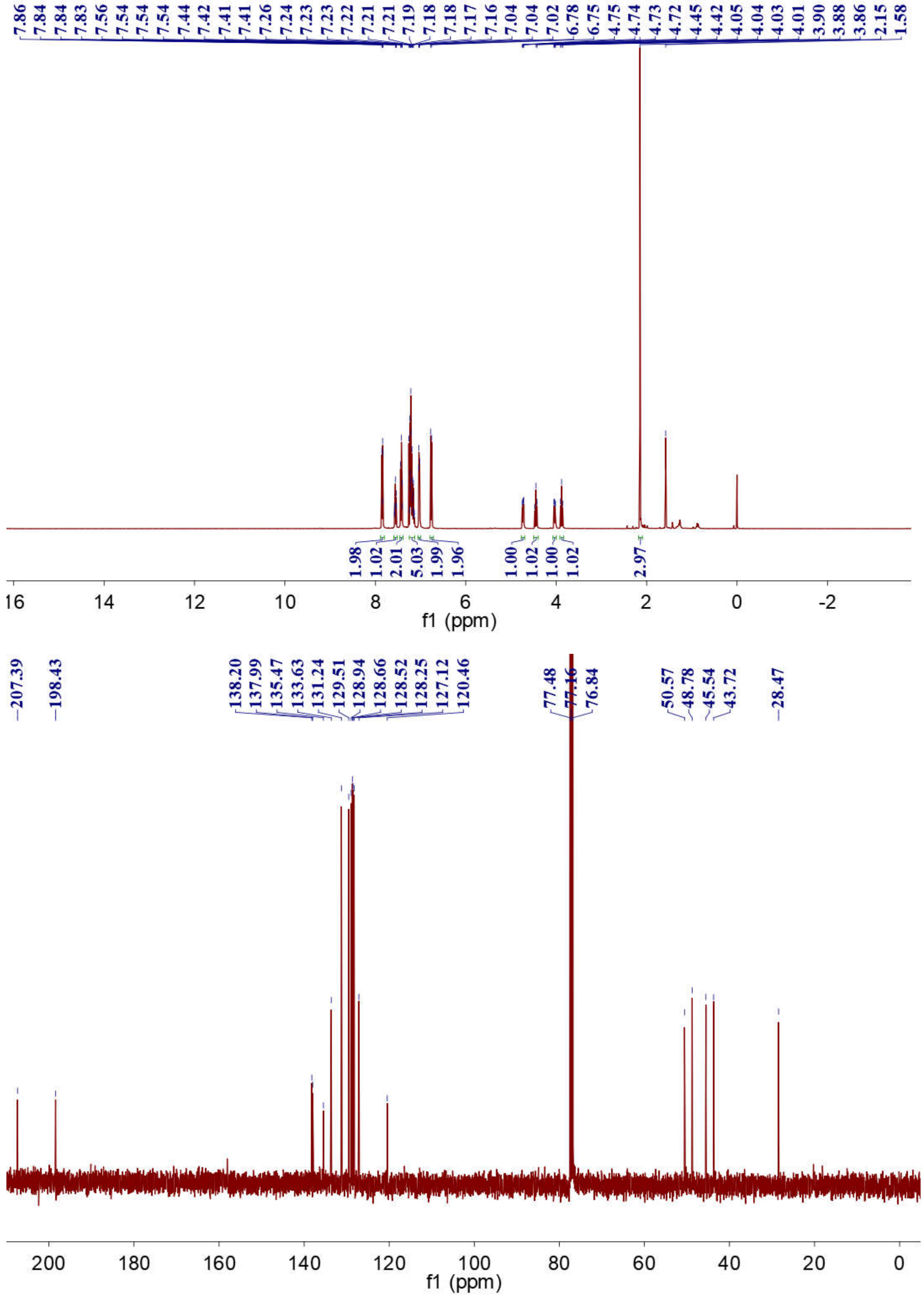

NMR spectra of product 69

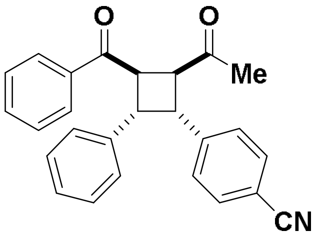

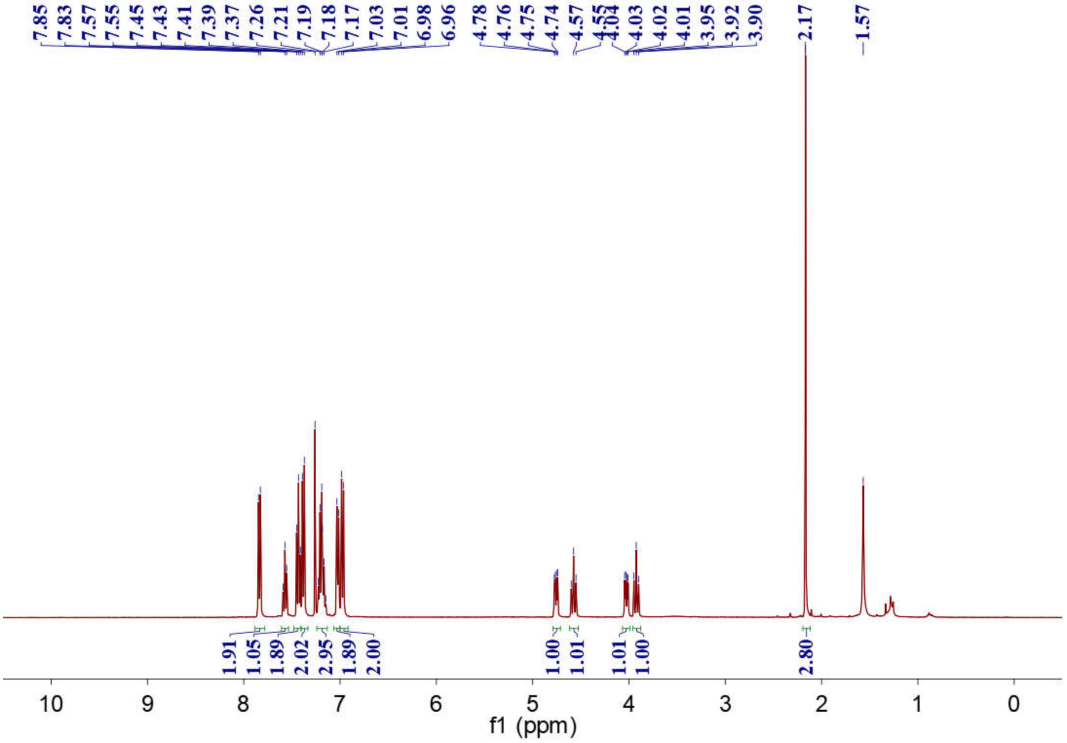

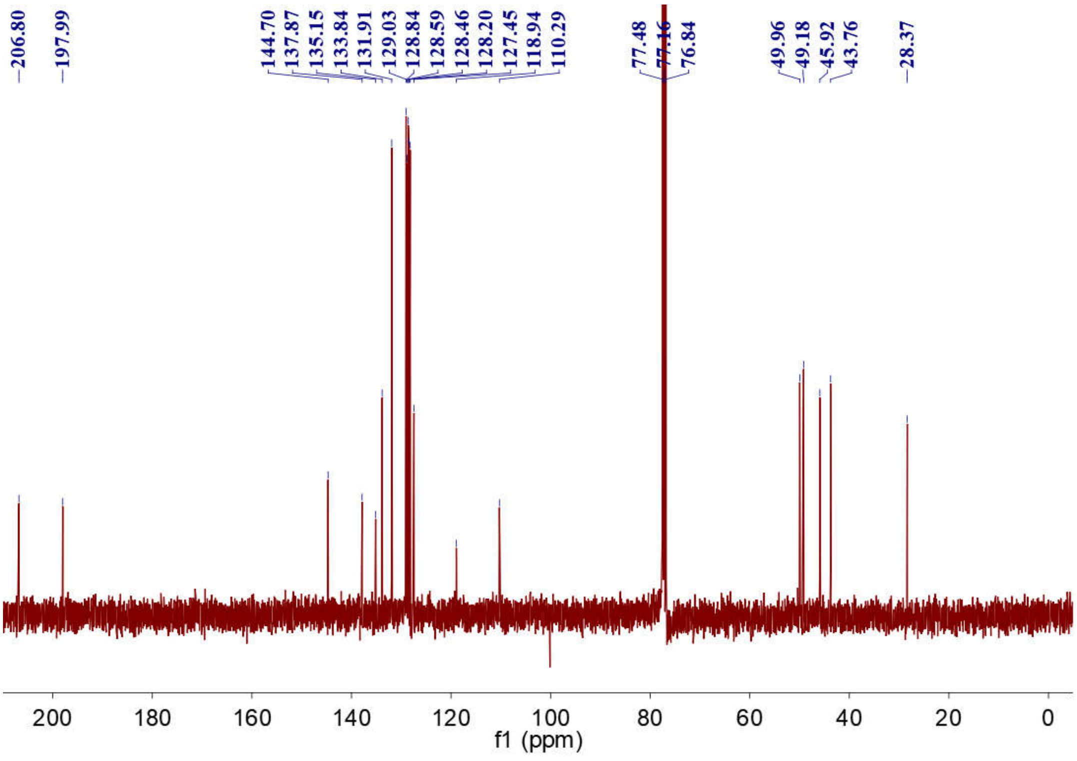

NMR spectra of product 70

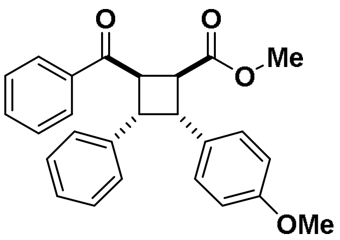

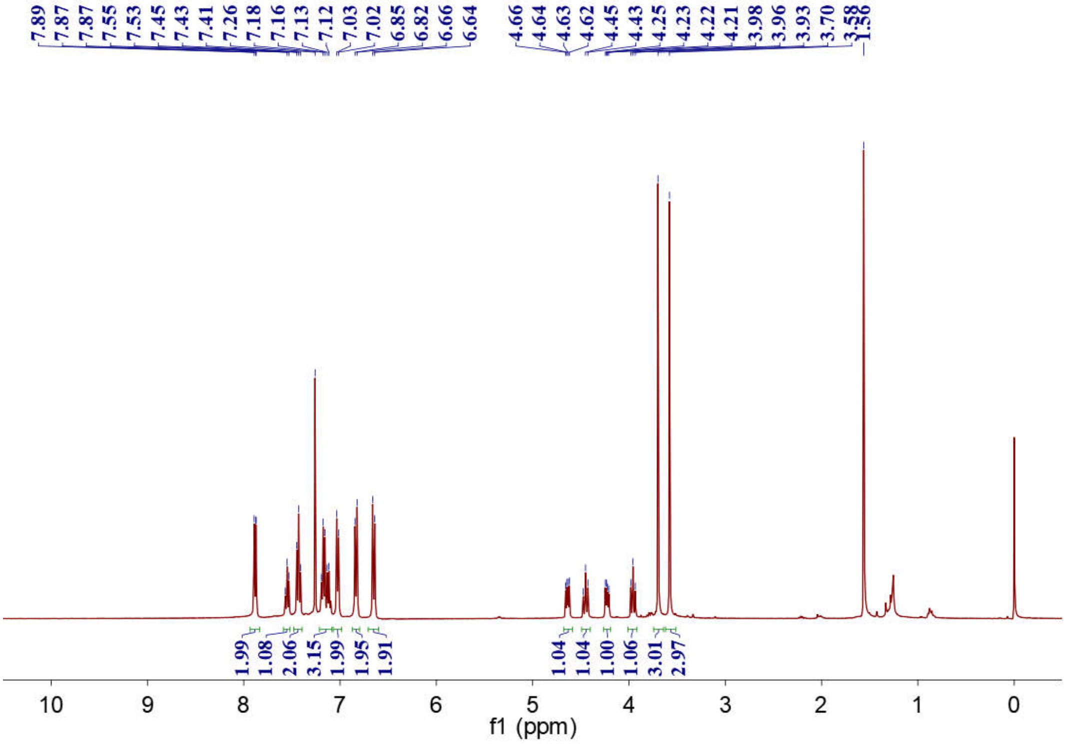

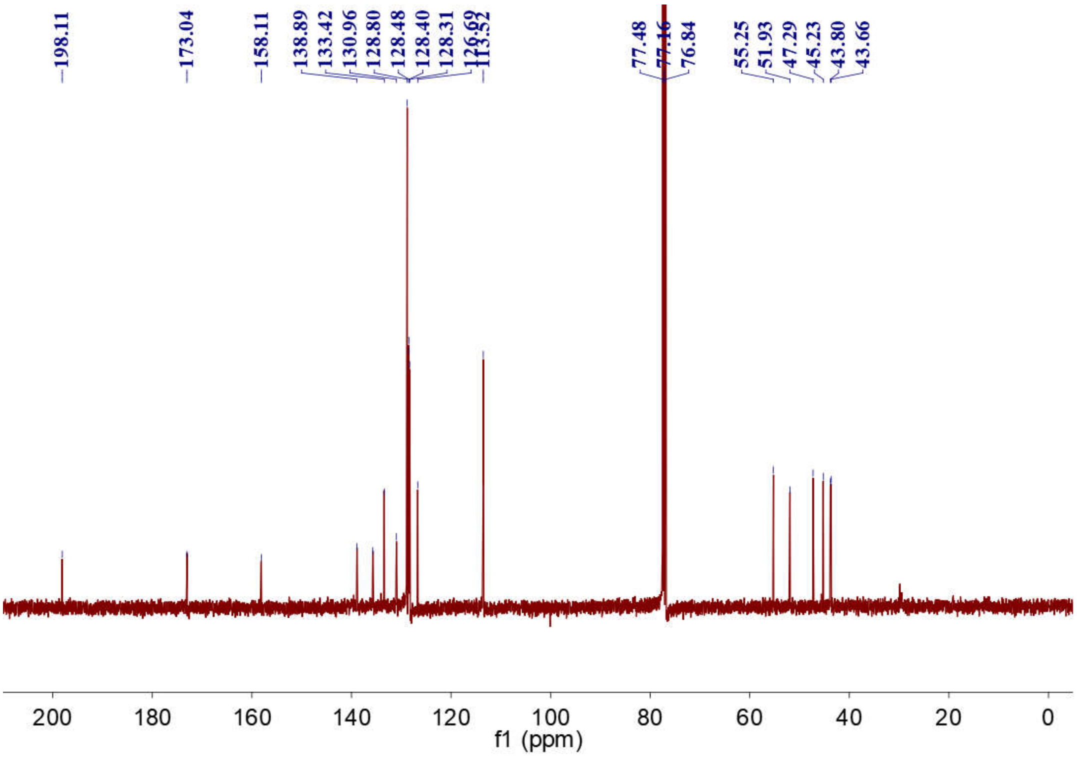

NMR spectra of product 71

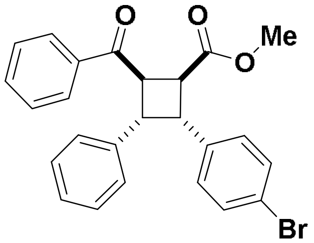

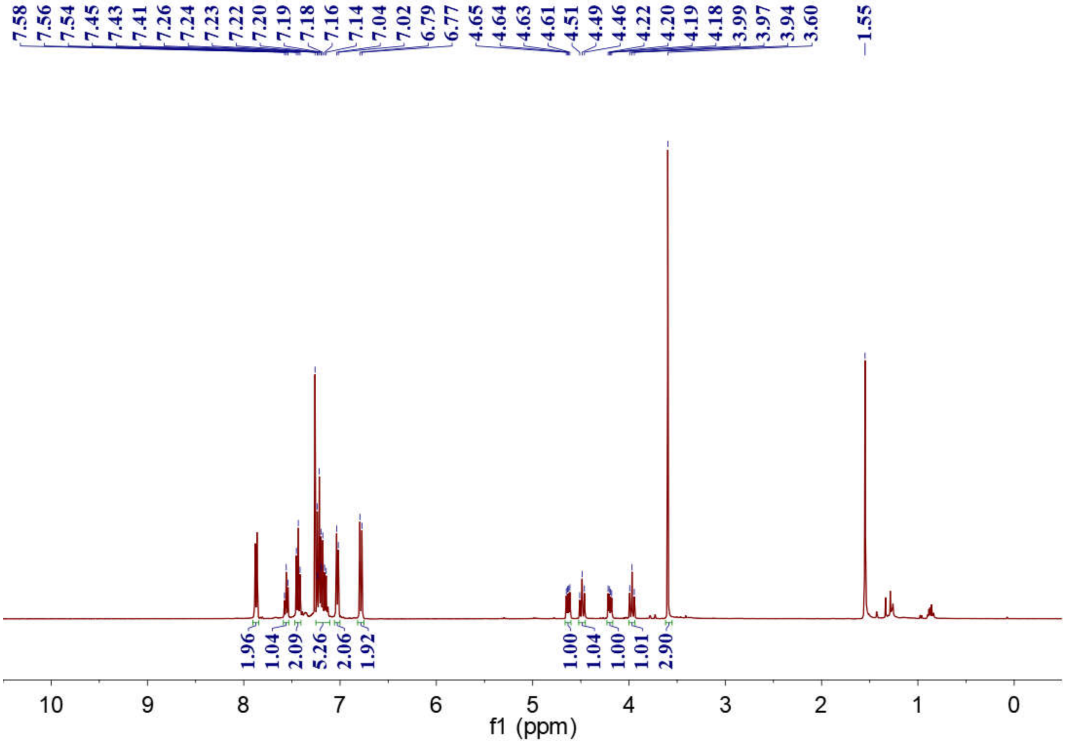

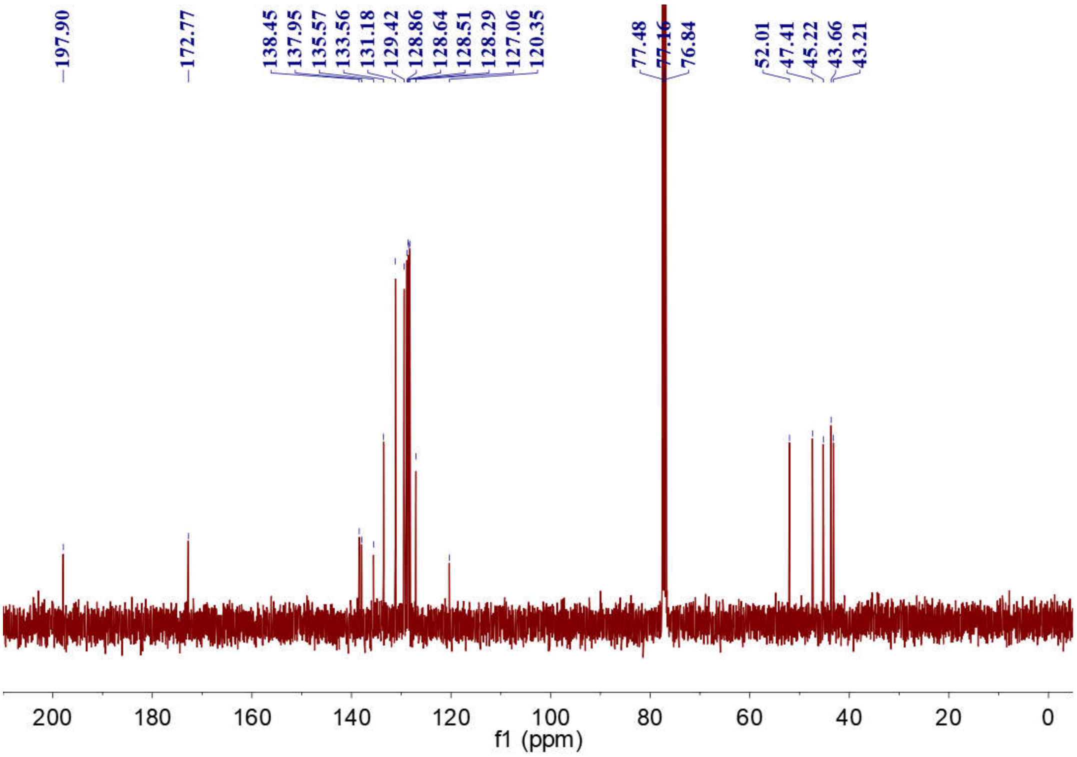

NMR spectra of product 72

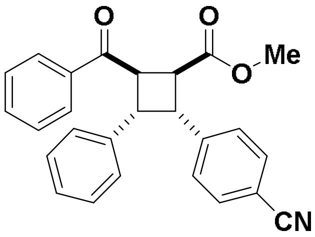

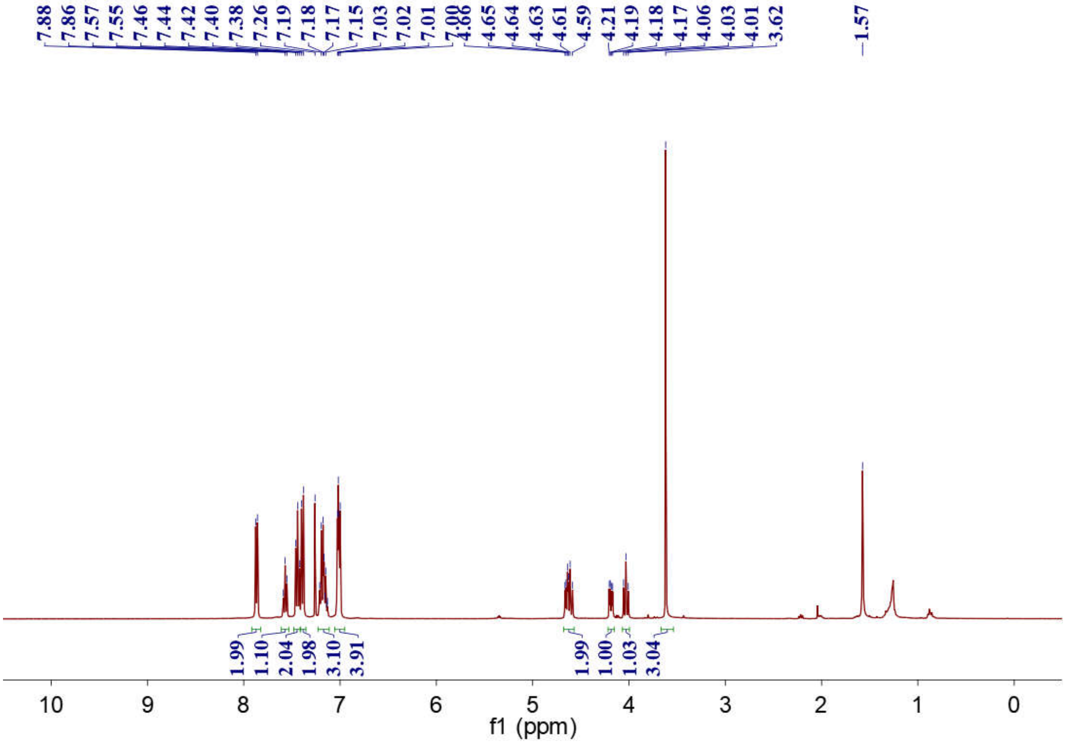

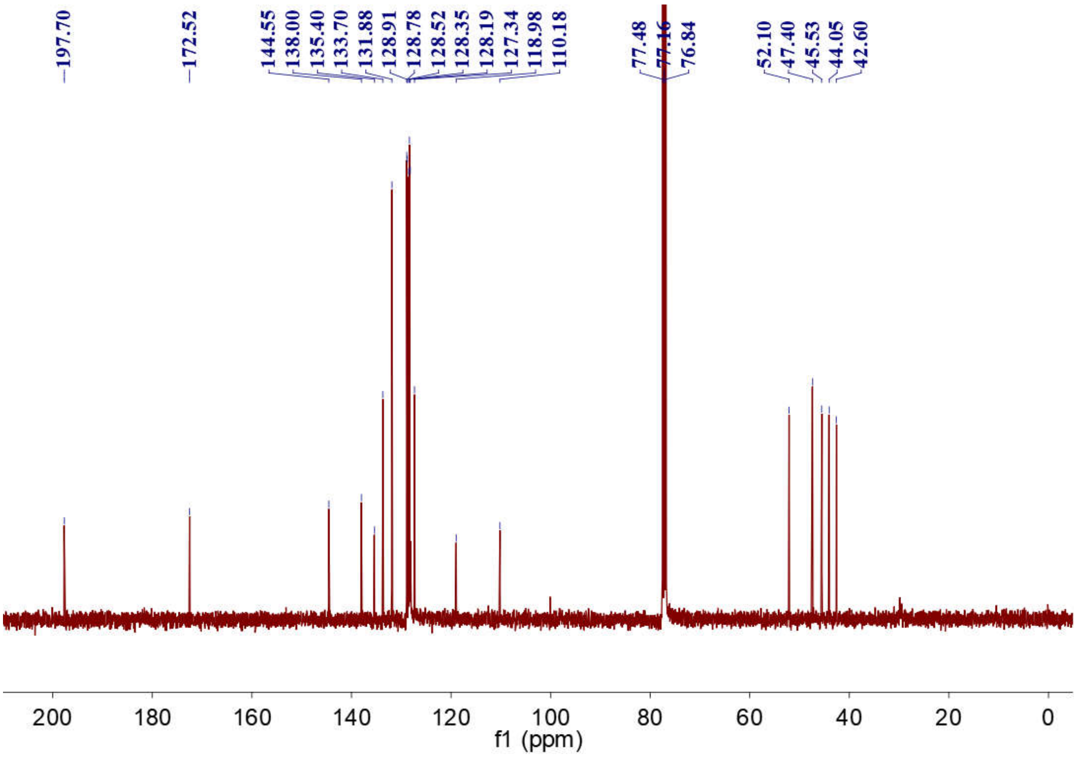

NMR spectra of product 73

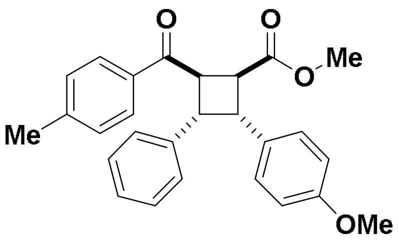

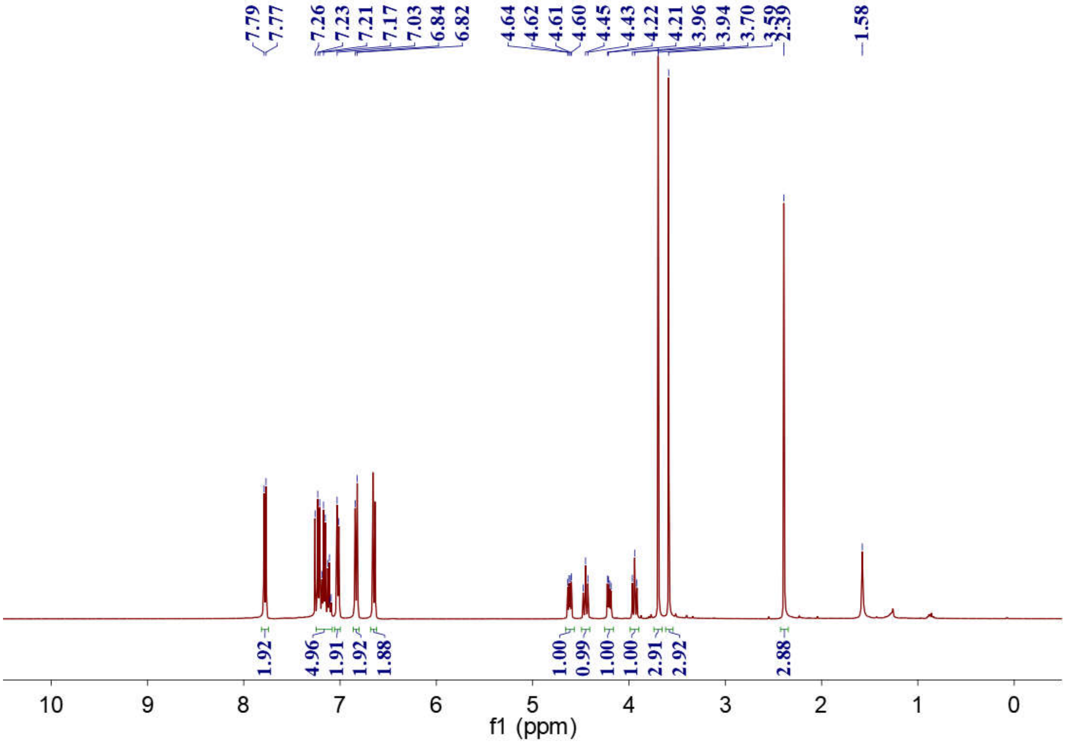

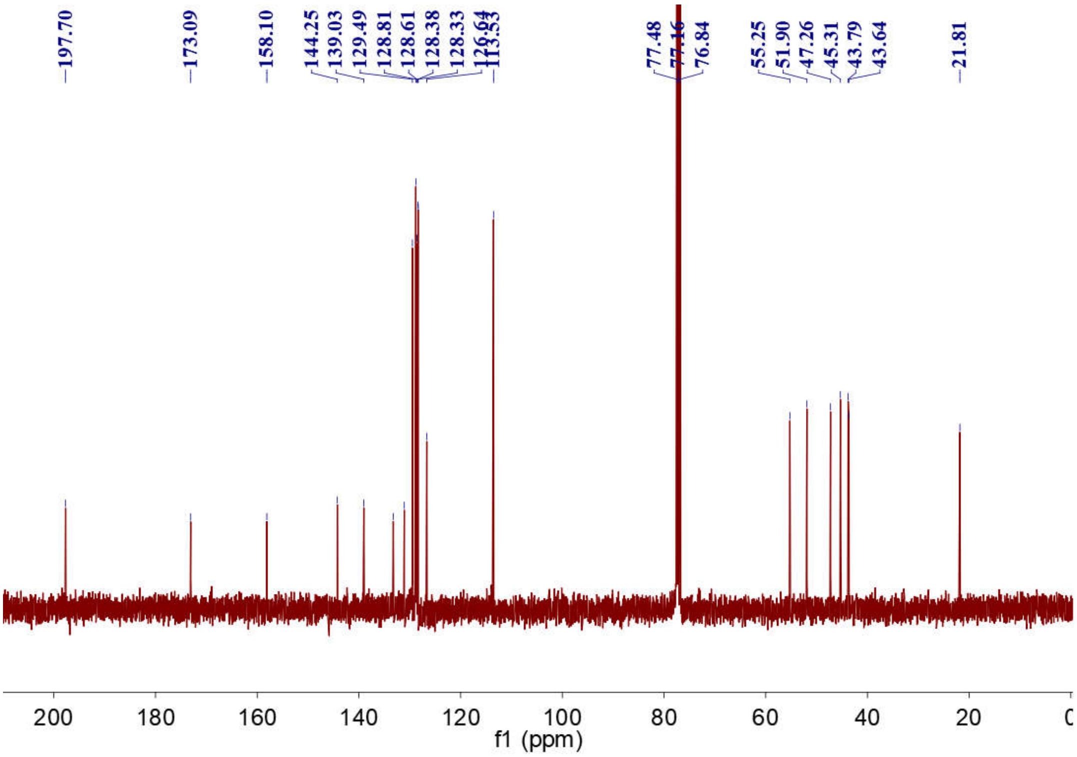

NMR spectra of product 74

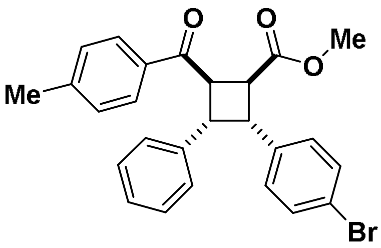

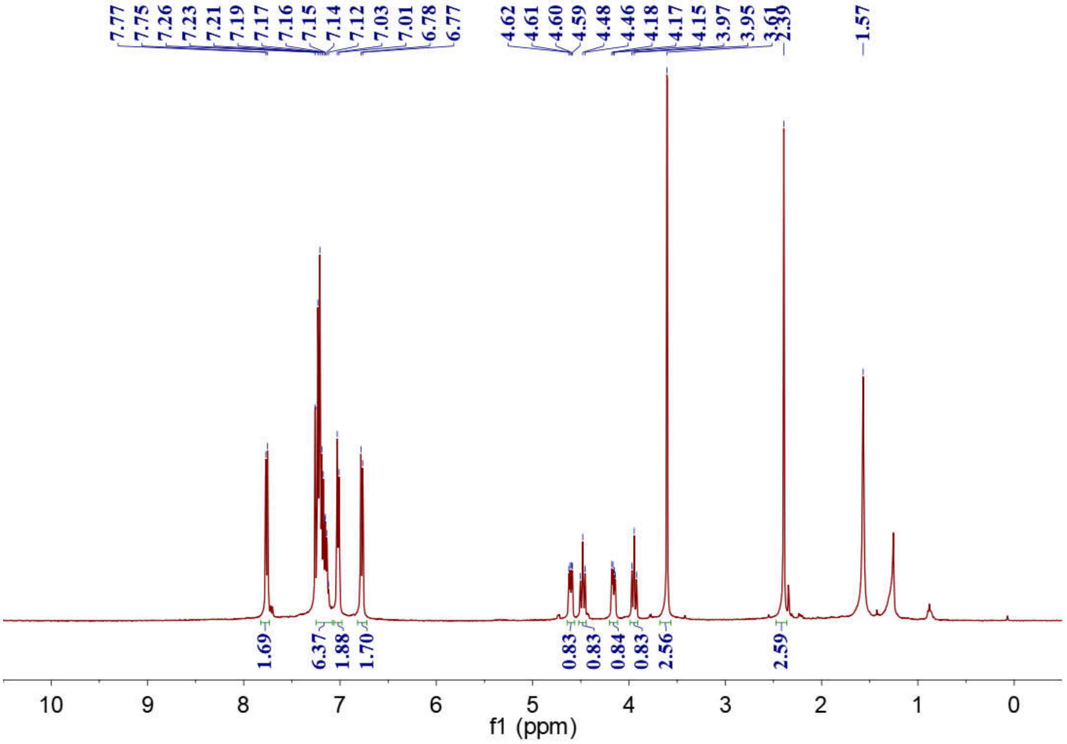

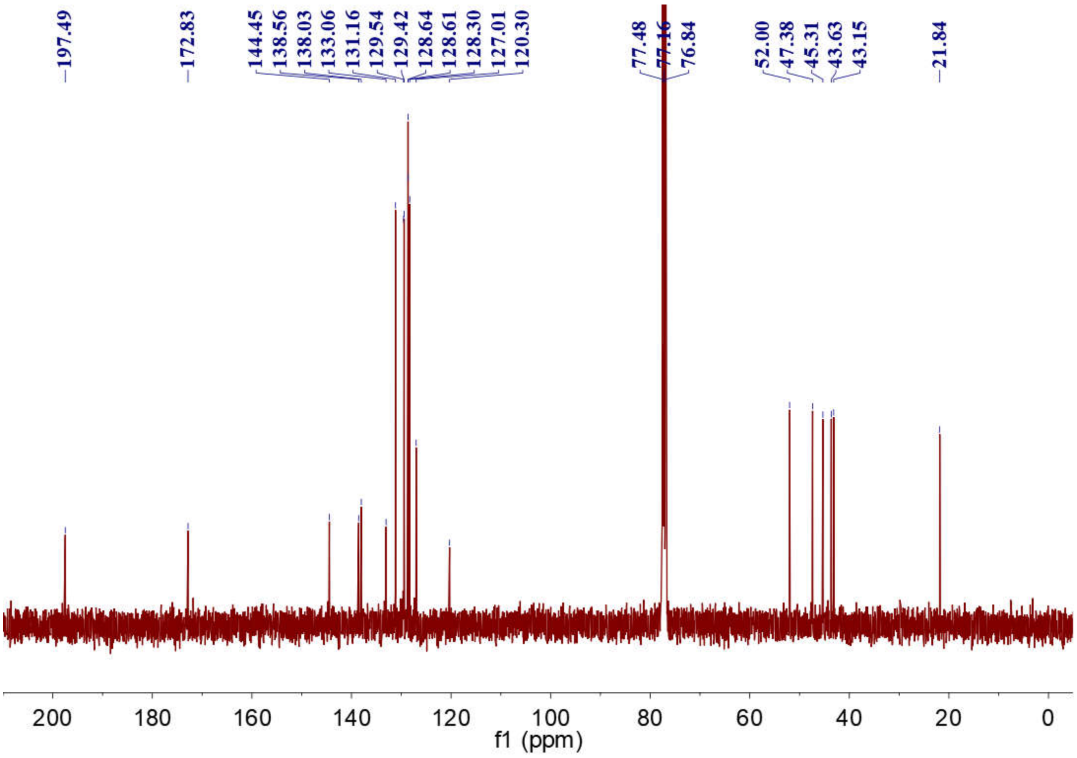

NMR spectra of product 75

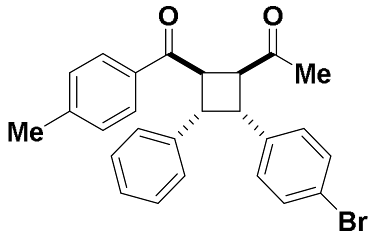

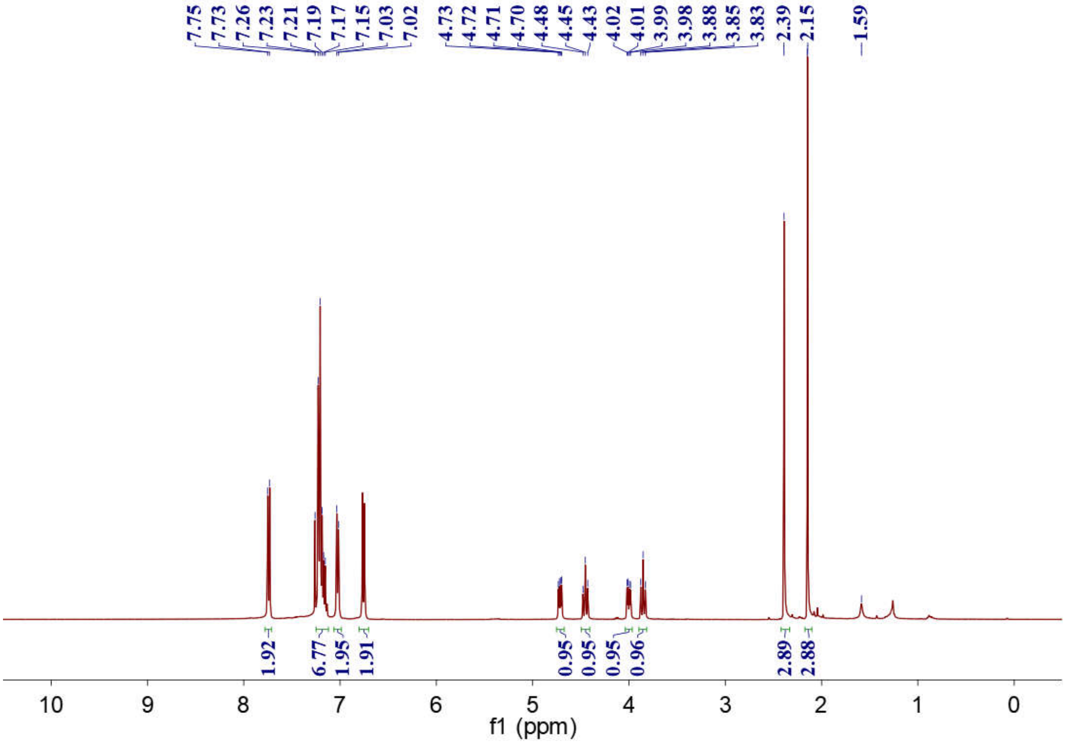

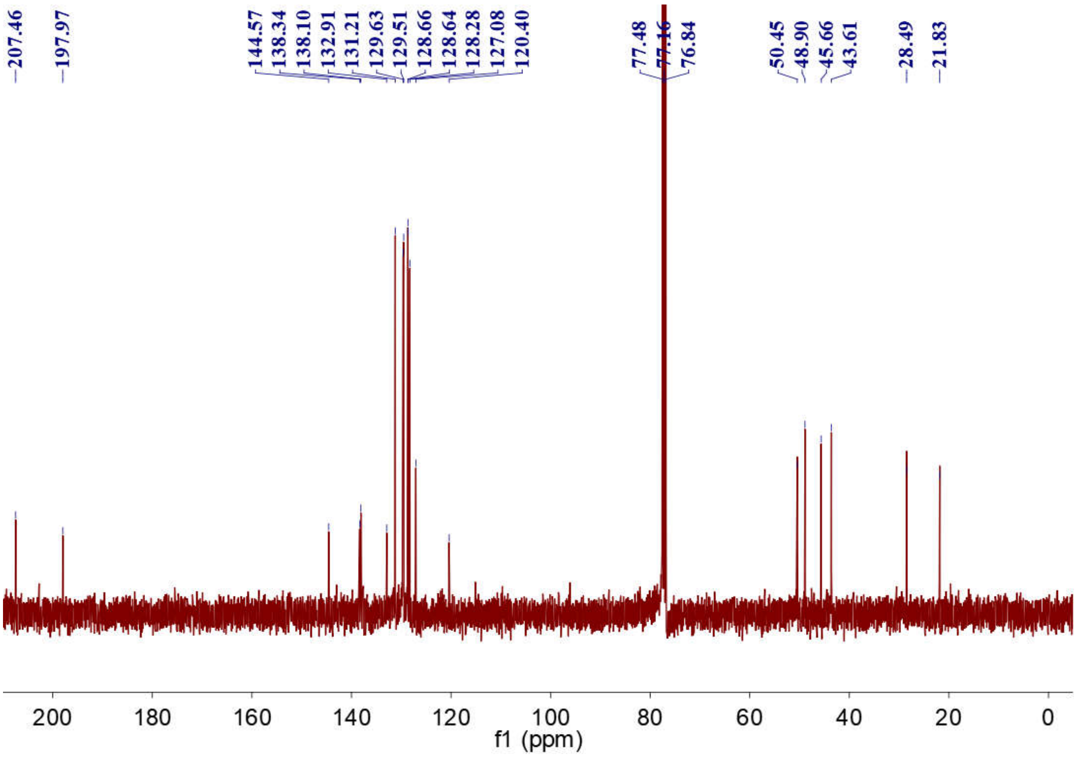

NMR spectra of product 76

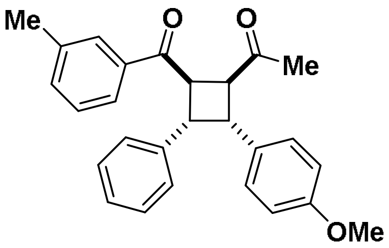

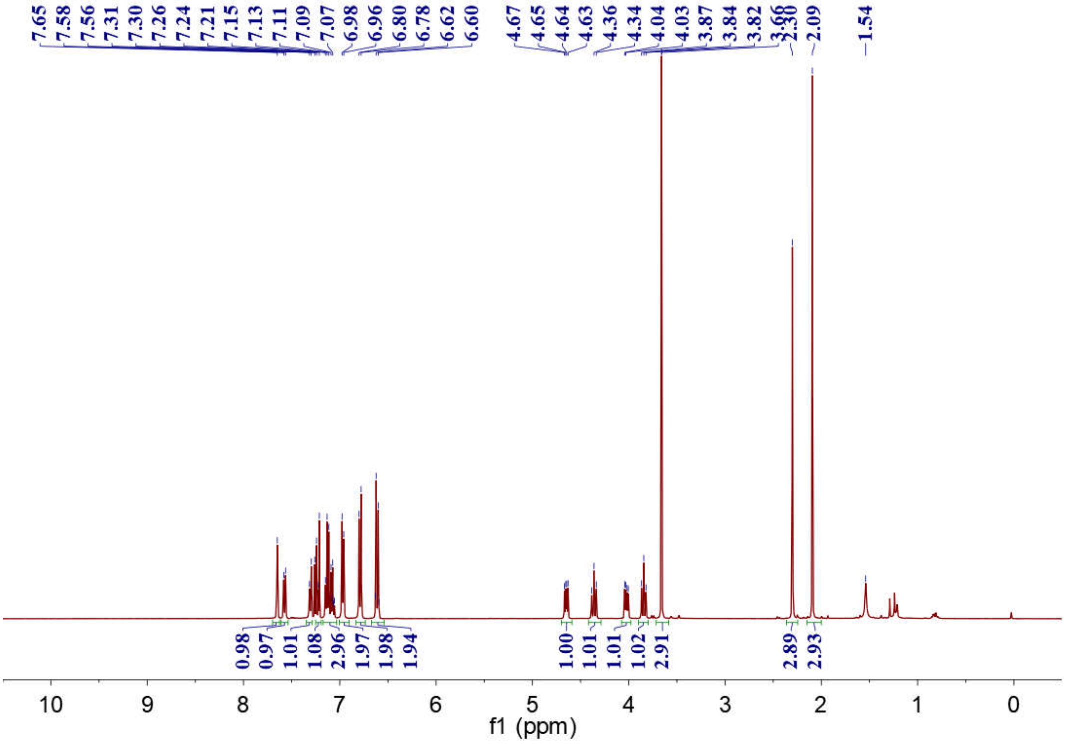

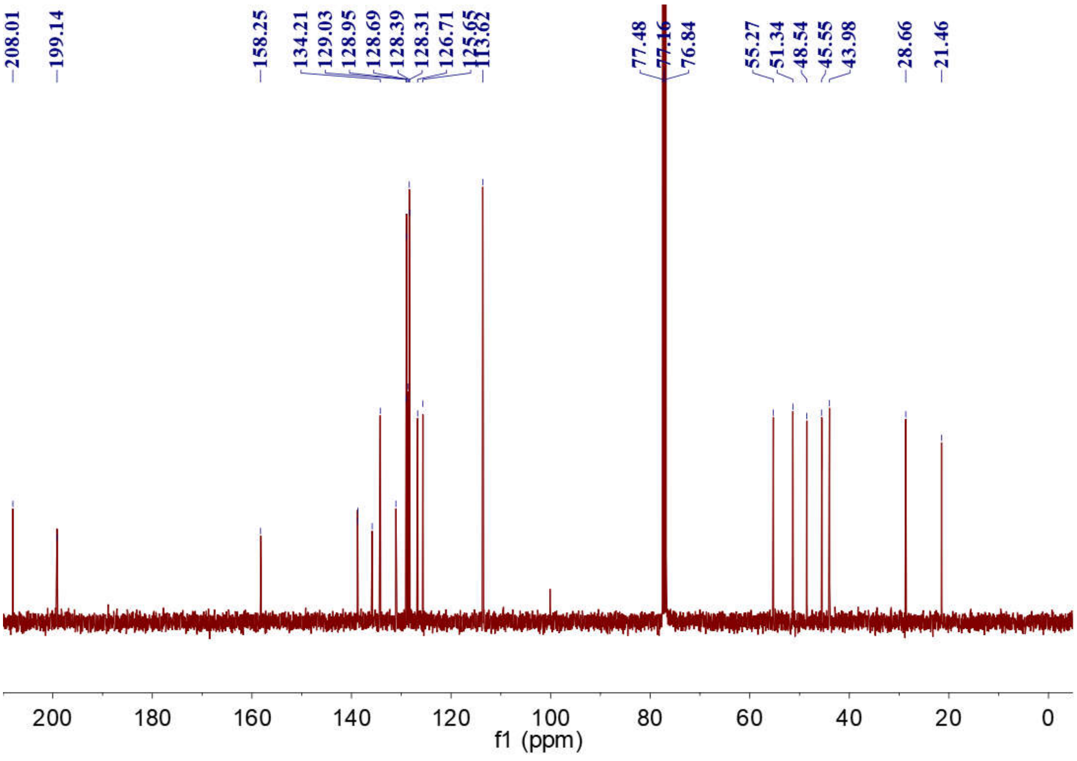

NMR spectra of product 77

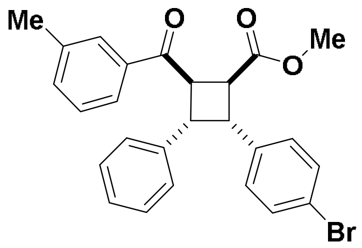

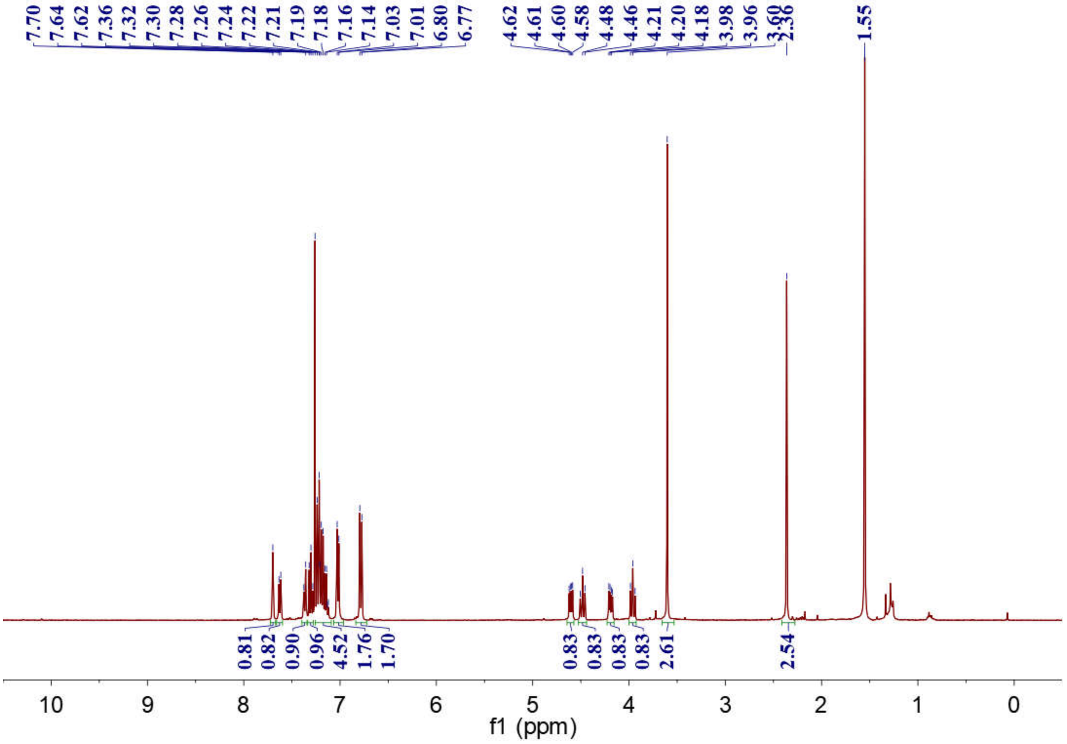

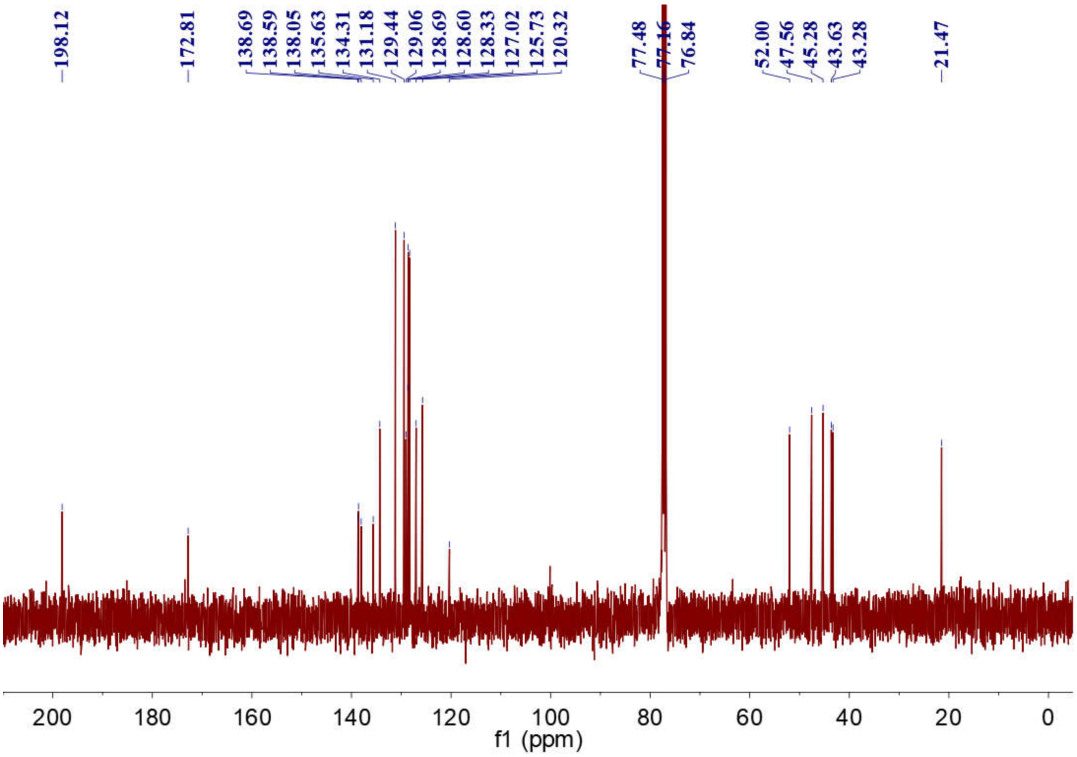

NMR spectra of product 78

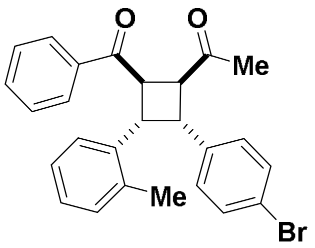

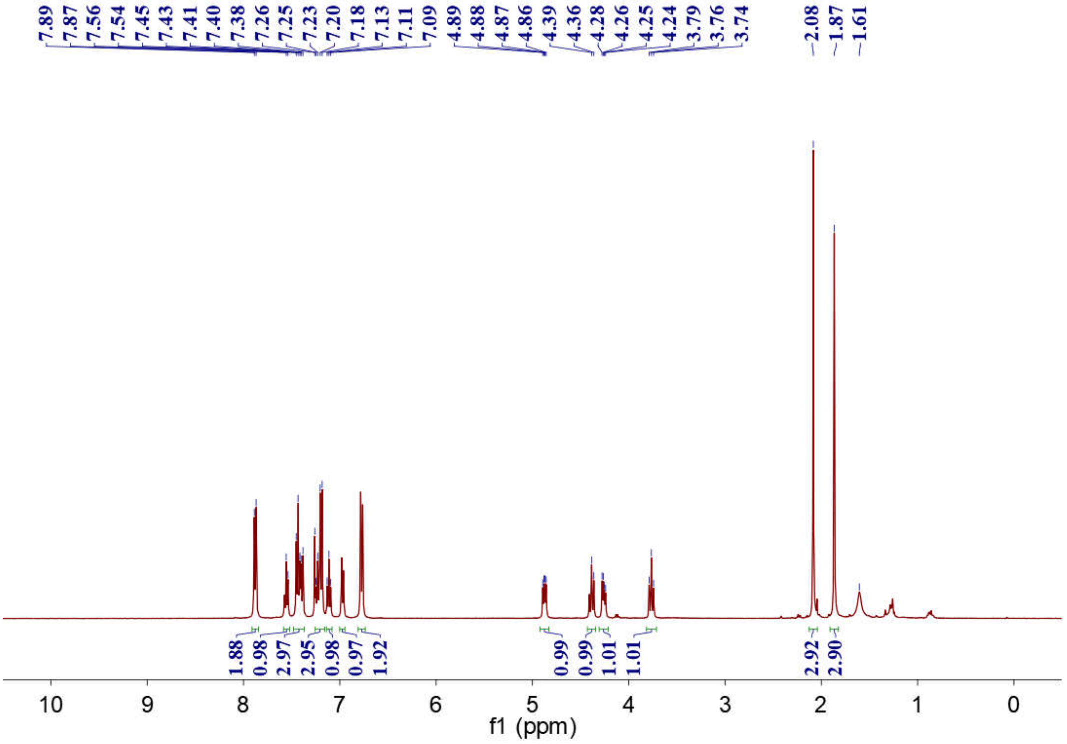

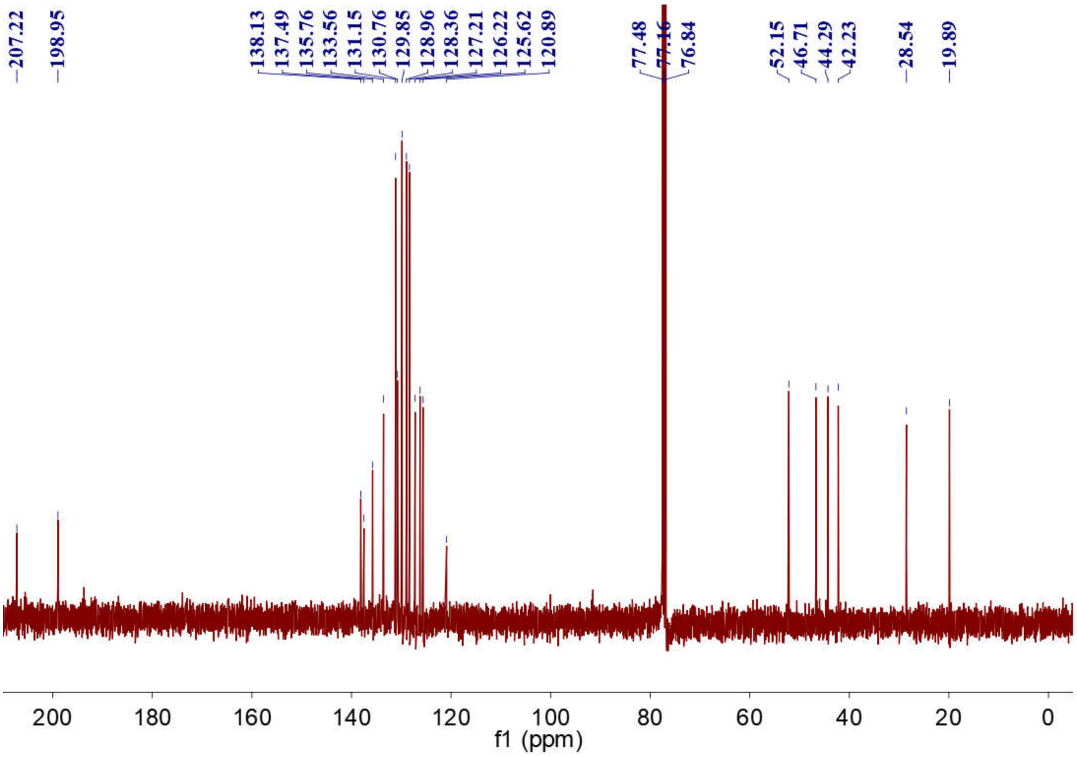

NMR spectra of substrate

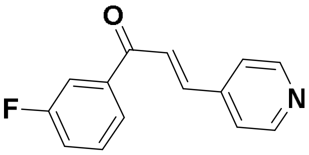

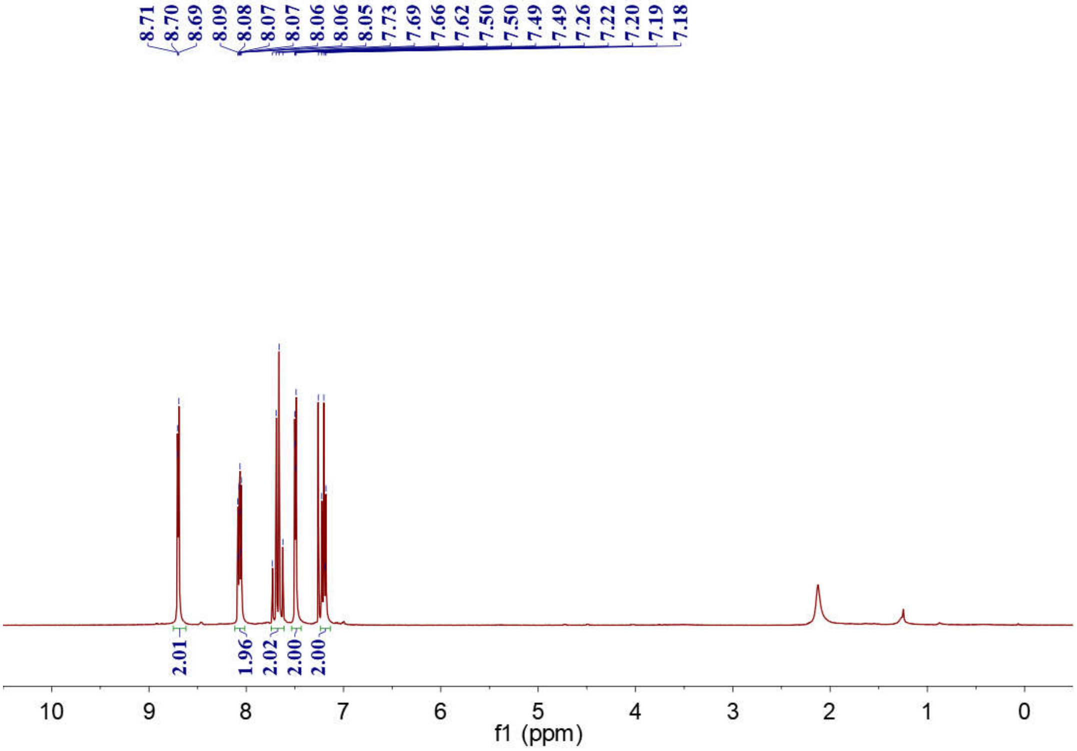

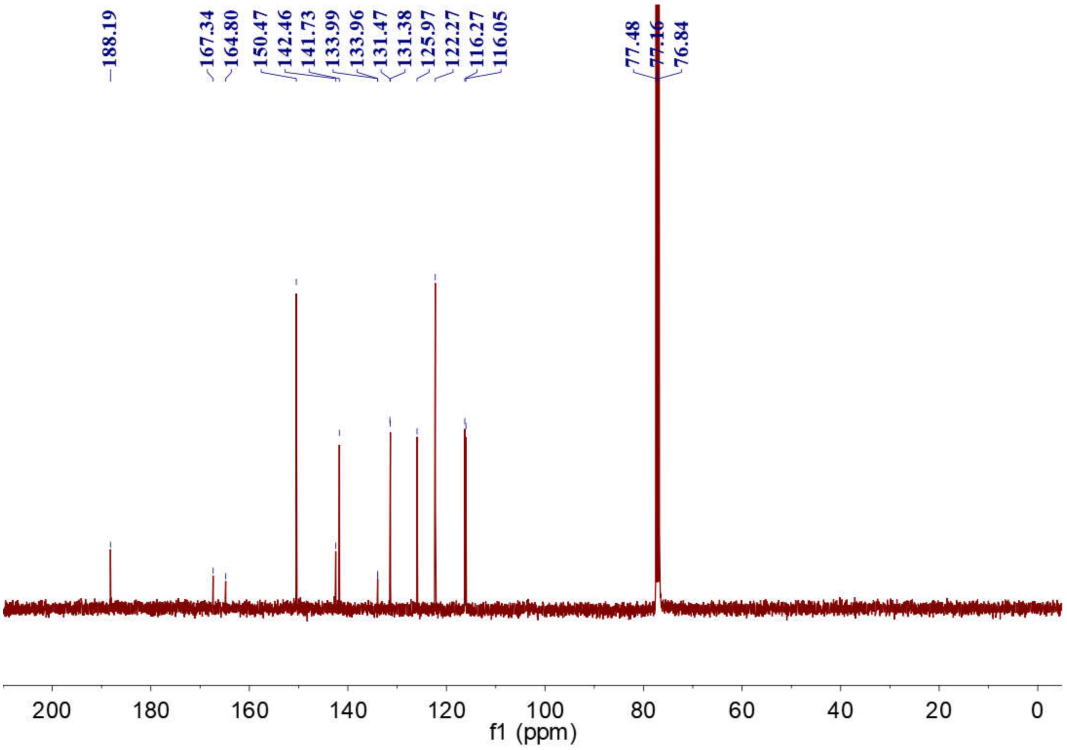

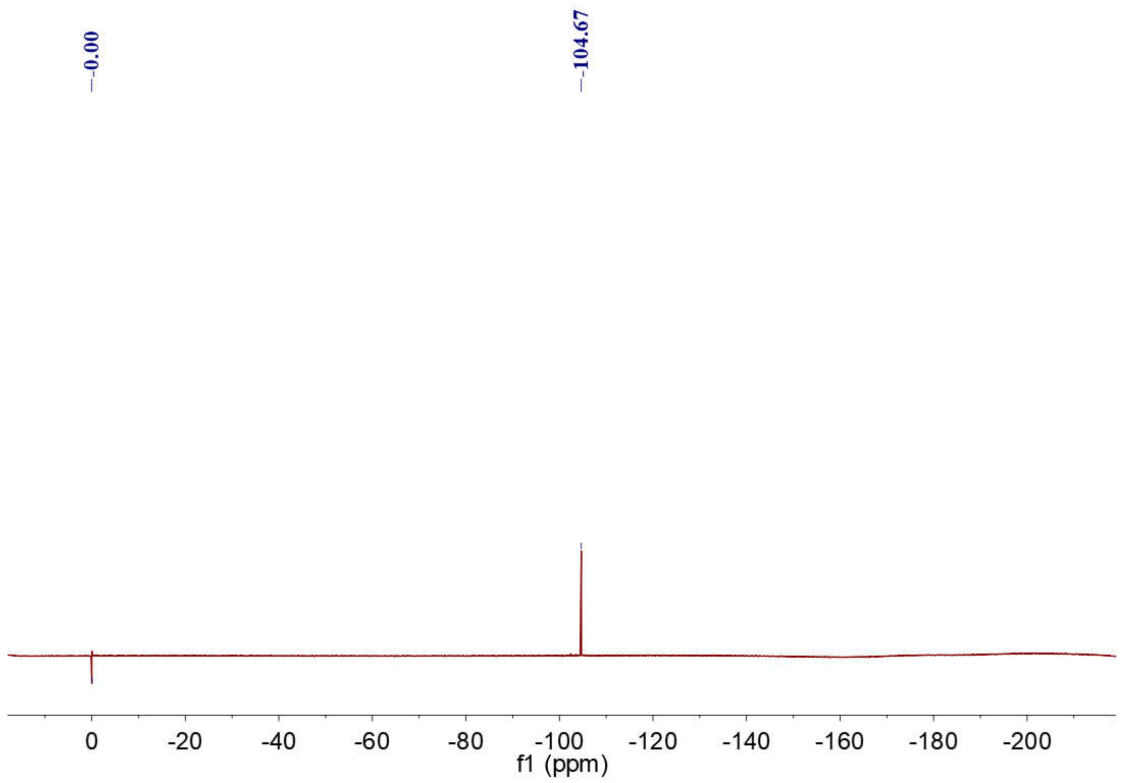

-0.00
-104.67
0
-20
-40
-60
-80
-100
-120
-140
-160
-180
-200
f1 (ppm)

Supplementary References

1. Li, K. *et al.* Stepwise assembly of $Pd_6(RuL_3)_8$ nanoscale rhombododecahedral metal-organic cages via metalloligand strategy for guest trapping and protection. *J. Am. Chem. Soc.* **136**, 4456-4459 (2014).
2. Sheldrick, G. A short history of SHELX. *Acta Crystallogr. A* **64**, 112-122 (2008).
3. Spek, L. PLATON SQUEEZE: a tool for the calculation of the disordered solvent contribution to the calculated structure factors. *Acta Crystallogra. C* **71**, 9-18 (2015).
4. Lei, T. *et al.* General and efficient intermolecular [2+2] photodimerization of chalcones and cinnamic acid derivatives in solution through visible-light catalysis. *Angew. Chem. Int. Ed.* **56**, 15407-15410 (2017).
5. Pagire, S. K., Hossain, A., Traub, L., Kerres, S., Reiser, O. Photosensitised regioselective [2+2]-cycloaddition of cinnamates and related alkenes. *Chem. Commun.* **53**, 12072-12075 (2017).
6. Gao, P.-S. *et al.* A robust multifunctional ligand-controlled palladium-catalyzed carbonylation reaction in water. *Chem. Commun.* **54**, 5074-5077 (2018).
7. Gomez-Rivera, A., Aguilar-Mariscal, H., Romero-Ceronio, N., Roa-de la Fuente, L. F., Lobato-Garcia, C. E. Synthesis and anti-inflammatory activity of three nitro chalcones. *Bioorg. Med. Chem. Lett.* **23**, 5519-5522 (2013).
8. Smit, F. J., van Biljon, R. A., Birkholtz, L.-M., N'Da, D. D. Synthesis and in vitro biological evaluation of dihydroartemisinyl-chalcone esters. *Eur. J. Med. Chem.* **90**, 33-44 (2015).
9. Kazi, I., Guha, S., Sekar, G. CBr_4 as a halogen bond donor catalyst for the selective activation of benzaldehydes to synthesize α,β-unsaturated ketones. *Org. Lett.* **19**, 1244-1247 (2017).
10. Yoshii, R., Nagai, A., Chujo, Y. Highly near‐infrared photoluminescence from aza‐borondipyrromethene‐based conjugated polymers. *J. Polym. Sci., Part A: Polym. Chem.* **48**, 5348-5356 (2010).
11. Jeong, E. J., Lee, I.-S. H. Studies of NMR chemical shifts of chalcone derivatives of five-membered monoheterocycles and determination of aromaticity indices. *Bull. Korean Chem. Soc.* **40**, 668-673 (2019).
12. Yang, X.-Y. *et al.* Mechanistic insights into the role of PC- and PCP-type palladium catalysts in asymmetric hydrophosphination of activated alkenes incorporating potential coordinating heteroatoms. *Dalton Trans.* **45**, 13449-13455 (2016).
13. Rosas-Sanchez, A., Toscano, R. A., Lopez-Cortes, J. G., Carmen Ortega-Alfaro, M. An expedient approach to synthesize fluorescent 3-substituted 4H-quinolizin-4-ones via (eta(4)-vinylketene)-Fe(CO)(3) complexes. *Dalton Trans.* **44**, 578-590 (2015).
14. James, J. P., Bhat, K. I., More, U. A., Joshi, S. D. Design, synthesis, molecular modeling, and ADMET studies of some pyrazoline derivatives as shikimate kinase inhibitors. *Med. Chem. Res.* **27**, 546-559 (2018).
15. Sheshenev, A. E., Boltukhina, E. V., White, A. J. P., Hii, K. K. Methylene-bridged bis(imidazoline)-derived 2-oxopyrimidinium salts as catalysts for asymmetric michael reactions. *Angew. Chem., Int. Ed.* **52**, 6988-6991 (2013).
16. Thiemann, T., Watanabe, M., Tanaka, Y., Mataka, S. Solvent-free Wittig olefination with stabilized phosphoranes-scope and limitations. *New. J. Chem.* **28**, 578–584 (2004).
17. Ambler, B. R., Altman, R. A. Copper-catalyzed decarboxylative trifluoromethylation of allylic bromodifluoroacetates. *Org.Lett.* **15**, 5578-5581(2013).
18. Cao, W., Liu, X., Peng, R., He, P., Feng X. Catalytic asymmetric cross-dehydrogenative coupling: activation of C-H bonds by a cooperative bimetallic catalyst system. *Chem. Commun.* **49**, 3470-3472

(2013).
19. Zhang, P., Brozek, L. A., Morken, J. P. Pd-catalyzed enantioselective allyl-allyl cross coupling. *J. Am. Chem. Soc.* **132**, 10686-10688 (2010).
20. Konrádová, D., Kozubíková, H., Doležal, K., Pospíšil, J. Microwave‐assisted synthesis of phenylpropanoids and coumarins: Total synthesis of osthol. *Eur. J. Org. Chem.* **35**, 5204–5213 (2017).
21. El-Batta, A. *et al.* Wittig reactions in water media employing stabilized ylides with aldehydes. Synthesis of α,β-unsaturated esters from mixing aldehydes, α-bromoesters, and Ph_3P in aqueous $NaHCO_3$. *J. Org. Chem.* **72**, 5244-5259 (2007).
22. Tchilibon, S. *et al.* Exploring distal regions of the A_3 adenosine receptor binding site: sterically constrained N^6-(2-phenylethyl) adenosine derivatives as potent ligands. *Bioorg. Med. Chem.* **12**, 2021–2034 (2004).
23. Yamada, Y. M. A., Takeda, K., Takahashi, H., Ikegami, S. Assembled catalyst of palladium and non-cross-linked amphiphilic polymer ligand for the efficient heterogeneous Heck reaction. *Tetrahedron* **60**, 4097-4105 (2004).
24. Lu, W. *et al.* Search for novel histone deacetylase inhibitors. Part II: Design and synthesis of novel isoferulic acid derivatives. *Bioorg. Med. Chem.* **9**, 2707-2713 (2014).
25. Yi, C., Hua, R. An efficient palladium-catalyzed Heck coupling of aryl chlorides with alkenes. *Tetrahedron Lett.* **40**, 2573-2576 (2006).
26. Ohtaka, A. *et al.* Linear polystyrene-stabilized Rh(III) nanoparticles for oxidative coupling of arylboronic acids with alkenes in water. *J. Organomet. Chem.* **873**, 1-7 (2018).
27. Liu, Y. C., Wu, Z. L. Switchable asymmetric bio-epoxidation of α, β-unsaturated ketones. *Chem. Commun.* **52**, 1158-1161 (2016).
28. Dong, J. *et al.* Design, synthesis, and biological evaluation of 2-(5-methyl-1H-pyrazol-1-yl) acetamide derivatives as androgen receptor antagonists. *Med. Chem. Res.* **28**, 380-386 (2019).
29. Leung P. S., Teng Y., Toy P. H. Chromatography-free Wittig reactions using a bifunctional polymeric reagent. *Org Lett.* **12**, 4996‐4999 (2010).
30. Cristau H. J., Taillefer M. Reactivity of substituted and unsubstituted diphenylphosphonium diylides towards carbonic acids derivatives. *Tetrahedron* **54**, 1507-1522 (1998).
31. Chen, S. et al. A metal-organic cage incorporating multiple light harvesting and catalytic centres for photochemical hydrogen production. *Nat. Commun.* **7,** 13169 (2016).
